# Global, regional, and national burden of chronic kidney disease and its associated anemia, 1990 to 2021 and predictions to 2050: an analysis of the global burden of disease study 2021

**DOI:** 10.1186/s12882-025-04398-4

**Published:** 2025-08-27

**Authors:** Qiao Qi, Yongtao Hu, Qiqi Shen, Kun Tang, Jie Yu, Yuexian Xu, Qingfeng Huang, Bingbing Hou, Zongyao Hao

**Affiliations:** 1 Department of Urology, The First Affiliated Hospital of Anhui Medical University, 218th Jixi Road, Hefei, China; 2 Institute of Urology, Anhui Medical University, Hefei, China; 3 Anhui Province Key Laboratory of Urological and Andrological Diseases Research and Medical Transformation, Anhui Medical University, Hefei, China; 4 Department of Infectious Disease, The First Affiliated Hospital of Anhui Medical University, Hefei, China

**Keywords:** GBD, Chronic kidney disease, Socio-demographic index, Age-standardized rates, Anemia, Disability-adjusted life years

## Abstract

**Background:**

Our objective was to conduct a thorough evaluation of the burden of CKD and its associated anemia by age and sex at the global, regional, and national levels, with projections extending to 2050.

**Methods:**

The data from the Global Burden of Diseases (GBD) 2021 were used to describe relevant indicators of CKD and its associated anemia. At different geographic levels, subgroup analysis was carried out by sex, age, and Socio-Demographic Index (SDI). The time trend was examined using the joinpoint regression and decomposition analyses, and predictive analysis was utilized to further estimate the disease burden to 2050.

**Results:**

The incidence, prevalence, mortality, and Disability-Adjusted Life Years (DALYs) of CKD, along with the prevalence and Years Lived with Disability (YLDs) of CKD-associated anemia, maintained a steady increase and would continue until 2050. In addition, the ASRs of mortality and DALYs attributable to CKD in 2021 were highest in low SDI regions. Regionally, CKD exhibited the greatest ASRs of mortality and DALYs in Central Latin America in 2021. Meanwhile, the disease burden of CKD and its associated anemia also showed significant differences at different national levels probably mainly due to population growth and aging. Moreover, the prediction analysis showed that the ASR of incidence attributable to CKD continued to increase.

**Conclusions:**

With the global population growth and aging, the disease burden of CKD and its associated anemia is still high and varies significantly at the global, regional, and national levels, which requires healthcare professionals to refine targeted interventions.

**Supplementary Information:**

The online version contains supplementary material available at 10.1186/s12882-025-04398-4.

## Introduction

Globally, chronic kidney disease (CKD) is a public health problem of great concern. In recent years, the incidence and mortality owing to CKD have significantly increased due to population aging [1–4]. In the global ranking of causes of death, CKD rose from 17th in 1990 to 12th in 2017 [5, 6], which is expected to rise to 5th by 2040 [7]. Meanwhile, the treatment of CKD is costly, especially in the case of kidney replacement therapy (e.g., kidney transplantation). In the United States, the medical cost per CKD patient ranged from $28627 (G2) to $42902 (G5) [8]. In China, according to the Chinese Kidney Disease Network’s 2015 annual data report, the medical cost resulting from CKD hospitalizations was about $3430 million, accounting for 6.30% of the total expenses [9]. Moreover, CKD has a poor prognosis, affecting the daily lives of patients, which brings inestimable losses to individuals, families, and even the entire social economy.

Diabetes, obesity, hypertension, and other factors are common risk factors for CKD, and economic income is also an important aspect [10, 11]. Absolutely, the major causes and the disease burden of CKD are not uniformly distributed in different regions. For example, diabetes and hypertension are the primary causes of CKD in developed regions (e.g., Europe and North America), while glomerulonephritis is more prevalent in some developing countries in Africa and Asia [12]. CKD can also cause a series of serious complications, among which its associated anemia is caused by decreased erythropoietin (EPO) production due to decreased kidney function. CKD-associated anemia may increase the burden on the heart and lower patients’ quality of life. A meta-analysis indicated that about three-fifths of CKD patients in sub-Saharan Africa suffered from anemia [13]. It is worth noting that anemia plays a significant role in exacerbating cardiovascular dysfunction, especially in the context of cardiorenal syndrome (CRS). Anemia directly impairs cardiac function through mechanisms such as reducing the oxygen-carrying capacity of blood, increasing the cardiac load, and promoting pathological ventricular remodeling, and further aggravates renal injury, thereby significantly accelerating the progressive deterioration of cardiac and renal functions characteristic of CRS [14]. In addition, Sofue et al. found that 40.10% of patients with stage 4 CKD and 60.30% of those with stage 5 CKD were affected by anemia [15]. The annual direct medical costs of CKD patients with anemia are significantly higher than those of non-anemia patients, which indicates that active treatment of anemia and prevention of related complications is one of the keys to reduce the economic burden of CKD patients [16].

The Global Burden of Diseases (GBD) database offers a thorough scientific evaluation of the global impact of diseases, injuries, and associated risk factors. Based on the most recent information, the study, which evaluated the impact of CKD and its associated anemia at different geographic levels, aimed to provide new estimates for all previous years and superseded previous results. In recent years, although there have been epidemiological investigations related to CKD, some of them have been limited to CKD caused by certain etiologies (such as diabetes, lead exposure or high-sodium diet, etc.) or in certain special populations, lacking macroscopic control over the epidemiology of CKD [17–19]. Secondly, some studies only report the epidemiological studies of CKD in a certain country or region, lacking an understanding of the epidemiology in all regions worldwide [20]. The research time of some literatures is relatively early and lacks certain timeliness [21]. Of course, the global burden of CKD-related anemia in this study has been rarely reported before. In addition, we predicted the future disease burden of CKD and its associated anemia, which is conducive to rational medical resource allocation and scientific health policy formulation for policymakers and implementers in the future.

## Methods

### Overview

In this study, the disease burden data for CKD and its associated anemia were developed by GBD collaborators using the Global Health Data Exchange Outcome Tool, with the aim of systematically assessing age - and sex-specific mortality rates for 288 causes of death, prevalence, and disability survival years for 371 diseases and injuries, and relative risk for 88 risk factors in 204 countries and regions between 1 January 1990 and 31 December 2021 [22, 23]. To estimate the disease burden attributable to diverse etiologies, we integrated multiple data sources—including vital registration systems, population-based health surveys, and clinical disease registries. These heterogeneous data were synthesized using DisMod-MR 2.1, a Bayesian meta-regression framework that employs hierarchical modeling to ensure internal consistency across epidemiological parameters (incidence, prevalence, remission, and mortality) for each location, age stratum, sex, and year. This approach generated globally comparable estimates while explicitly quantifying model uncertainty through 1,000 posterior draws. The comprehensive data processing protocol is publicly accessible via the Institute for Health Metrics and Evaluation’s Global Burden of Disease methodology portal (http://www.healthdata.org/gbd/). This standardized framework systematically addresses data gaps and inconsistencies through spatial-temporal smoothing and cross-validation techniques. However, in regions with limited primary data (particularly low-income settings), estimates rely on extrapolation from epidemiologically similar populations, which may introduce directional bias in burden quantification.

In this study, age standardization was performed using the direct method, with the proportion of each age group in the GBD standard population applied as a fixed weight. The age-specific rates were multiplied by these standard weights and then summed to derive the age-standardized rate (ASR). This approach effectively removes the influence of differences in population age structure across countries, regions, or time periods, enabling fair and comparable assessments of health indicators such as incidence and mortality rates. By standardizing to a common reference population, the ASR facilitates meaningful comparisons of disease burden between populations and over time. The GBD 2021 protocol was made available on the website of the Institute for Health Metrics and Evaluation [24]. The GBD 2021 study was authorized by the Institutional Review Board at the University of Washington.

### Definitions

CKD is defined as kidney injury or impaired renal function that persists for three months or longer, regardless of the cause [25]. The study data was divided by sex, 5-year age group, year and region. In addition, we estimated the burden of CKD by incidence, prevalence, mortality, and Disability-Adjusted Life Years (DALYs), as well as the burden of its associated anemia by prevalence and years lived with disability (YLDs). YLDs are the years of healthy life lost as a result of disease-induced disability, which is utilized as a metric to estimate the impact of disease-induced disability on a person’s health status. YLDs are calculated as follows: YLD = (prevalence rate × duration of disease) × disability weight [4]. The disability weight indicates the severity of the disease and ranges from 0 (complete health) to 1 (death). DALYs are a comprehensive index that measures the health loss caused by diseases or health problems, including the loss of healthy life years resulting from early death and disability caused by diseases [26]. The higher the value of DALYs, the heavier the disease burden.

Based on GBD data, we obtained sample statistics such as sample size and population standard deviation, and then obtained the Z-value corresponding to the confidence level through Z-distribution. The formula for calculating the 95% confidence interval is: 95%CI=¯x ± Z * (σ/√n), where¯x is the sample mean, Z is the Z-value for the chosen confidence level (approximately 1.96 for 95%), σ is the population standard deviation, and n is the sample size. The width of CI reflects both the precision and uncertainty of the estimate: a narrower CI indicates higher precision and less uncertainty, while a wider CI suggests greater variability or a smaller sample size. In epidemiological trend analysis, if the lower limit of the 95% CI exceeds zero, it signifies a statistically significant upward trend; if the upper limit is below zero, it indicates a significant downward trend. If the 95% CI contains zero, it suggests that the observed change is not statistically significant. The Socio-Demographic Index (SDI) is a comprehensive indicator to reflect the degree of national and regional development, which consists of the following key socio-demographic factors: Total fertility rate for females under 25, education level of females aged 15 and over, and per capita lagged distribution income [4, 27, 28]. The value of SDI varies between 0 and 1. The greater the SDI, the higher the degree of socioeconomic development in a country or region [29]. According to the SDI quintiles, the 204 countries and regions were divided into five groups: low SDI, low-middle SDI, middle SDI, high-middle SDI, and high SDI. The GBD super-regions are a geographical grouping formed by combining multiple second level GBD regions with similarities in SDI, epidemiological pattern (disease burden characteristics), and cultural background. The countries and regions included in the grouping can be seen in Table S1.

### Decomposition analysis

Decomposition analysis can reveal the factors behind shifts in incidence, prevalence, mortality, and DALYs over specified timeframes at different geographic scales [30]. This study delved deeper into how these underlying factors affect the epidemiological patterns of these health metrics. By decomposing the variations in incidence, prevalence, mortality, and DALYs of CKD and the prevalence and YLDs of its associated anemia, the study presented the impact of population growth, aging, and epidemiological change, thereby measuring their overall influence on these health indicators [12].

### Predictive analysis

The analyses presented concentrated on the impact of CKD and its associated anemia across recent decades. To strengthen public health strategy development and healthcare resource allocation, projections of CKDs and its associated anemia’s impact over the forthcoming decades have been conducted. The Bayesian age-period-cohort (BAPC) model, enhanced with integrated nested Laplace approximation (INLA), has been employed to forecast the global CKD and its associated anemia burden through to 2050, showing improved accuracy and reliability compared to the traditional APC model [31]. The marginal posterior distribution can be estimated using the INLA and BAPC model, potentially avoiding the mixing and convergence problems that are frequently encountered with the Markov Chain Monte Carlo approach, which is commonly used in Bayesian analysis [32].

### Statistical analysis

Study variables are expressed as quantities, percentages, and ratios. Also, in order to make meaningful comparisons of prevalence rates across populations worldwide, the age-standardized rate (ASR) was used to adjust the prevalence rates of different risk factors in a country by using the same standard population. The disease burden of CKD and its associated anemia was evaluated after stratification by sex, age, location, and SDI. Based on previous studies, our research used both CIs and UIs simultaneously, and the combination of the two enhanced the credibility of the research conclusions [33]. UIs were calculated by taking the 2 × 5th and 97 × 5th percentiles of the distribution of 1000 model runs after convergence. The UI analysis was used in the GBD to address the possible heterogeneity from both sampling error and non-sampling variance. However, CIs were mainly used for statistical inference to help understand the reliability of estimated values. The epidemiological trends in the incidence, prevalence, mortality, and DALYs of CKD (including the prevalence and YLDs of its associated anemia) were compared using the joinpoint regression model. The average annual percent change (AAPC) was used to estimate the disease burden trends of CKD and its associated anemia. Both the 95% confidence interval (CI) and the AAPC value over 0 indicate a rising trend, whereas both below 0 indicate a declining trend. Lastly, by sex and year, we assessed the burden of CKD and its associated anemia in different SDI regions. R software (version 4.4.1) was used for all analyses and data visualization. *p* < 0.05 was considered statistically significant.

## Results

### Global trends

**Fig. 1:**
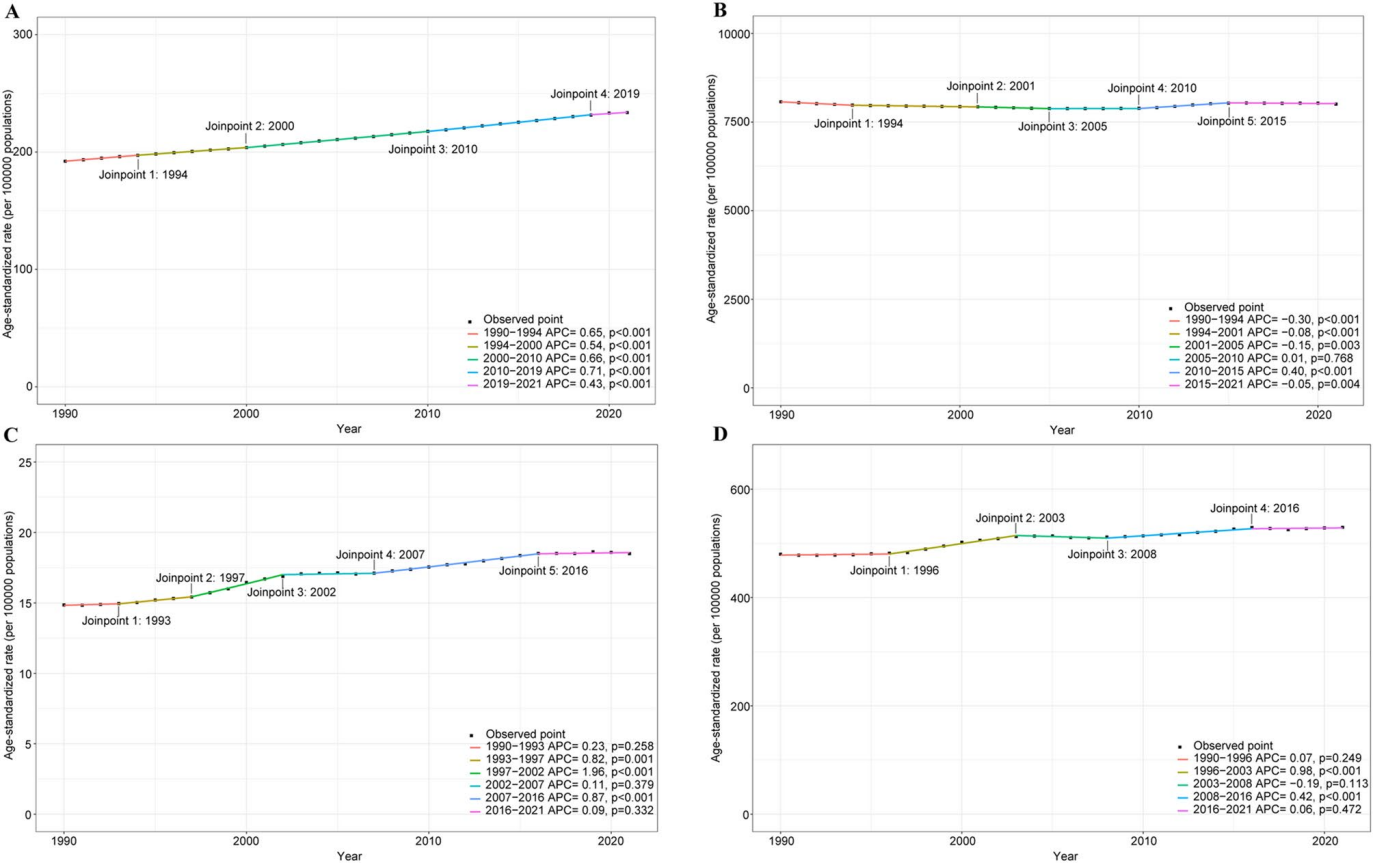
Joinpoint regression analysis of age-standardized rates of incidence, prevalence, mortality, and DALYs for CKD from 1990 to 2021. (**A**) Incidence; (**B**) Prevalence; (**C**) Mortality; (**D**) DALYs. DALYs, disability-adjusted life years; CKD, chronic kidney disease

The global incidence cases of CKD increased by nearly 156% from 7,790,705 (95%UI: 7226165–8402568) in 1990 to 19,935,038 (95%UI: 18702793–21170794) in 2021 (Table S2). The joinpoint regression analysis found that the ASIR of CKD changed significantly in 1994, 2000, 2010, and 2019 (Fig. 1). The age-standardized rate of prevalence (ASPR) of CKD showed a significant decline from 1990 to 1999 (AAPC: −0.18, 95%CI: −0.21 to −0.15) and increased significantly from 2010 to 2021 (AAPC: 0.16, 95%CI: 0.13 to 0.19) (Table S3, Table S6). The joinpoint regression analysis found that the ASPR of CKD during 1990–2021 changed significantly in 1994, 2001, 2005, 2010, and 2015 (Fig. 1). In addition, the age-standardized rate of mortality (ASMR) and age-standardized DALYs of CKD showed a steady upward trend in most periods (Tables S4, S5, Table S7). The ASMR increased most significantly from 1997 to 2002, while the age-standardized DALYs showed the highest increase from 1996 to 2003 (Fig. 1).

The prevalence cases of CKD-associated anemia in 2021 were 63,751,624 (95%UI: 59045051–68372650), which increased by 96% compared with 32,486,224 (95%UI: 30356876–35047084) in 1990 (Table S8). Meanwhile, the number of YLDs for CKD-associated anemia has also shown an increasing trend year by year (Table S9). Furthermore, the ASRs of prevalence and YLDs for CKD-associated anemia showed a decreasing trend between 1990 and 2021 (Table S10). The joinpoint regression analysis showed that the ASPR decreased most significantly between 1990 and 1993, while the age-standardized YLDs showed the largest decrease between 1990 and 1999 (Fig S1, Table S11).

### Global trends by sex and age groups

**Fig. 2:**
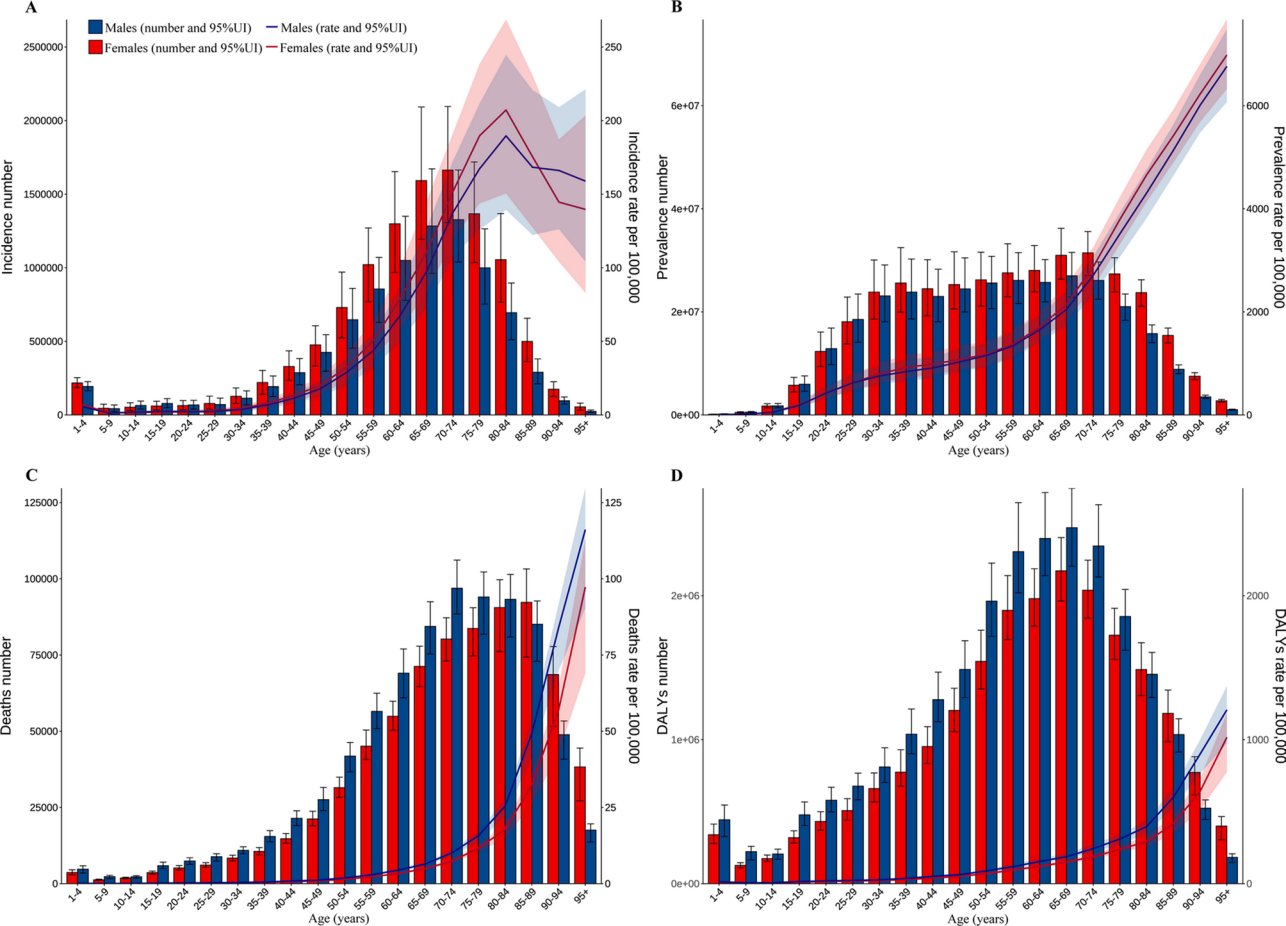
Numbers and age-specific rates of incidence, prevalence, mortality, and DALYs for CKD by age group and sex in 2021. (**A**) Incidence; (**B**) Prevalence; (**C**) Mortality; (**D**) DALYs; bar charts represent counts; lines represent crude rates. CKD, chronic kidney disease; DALYs, disability-adjusted life years

**Fig. 3:**
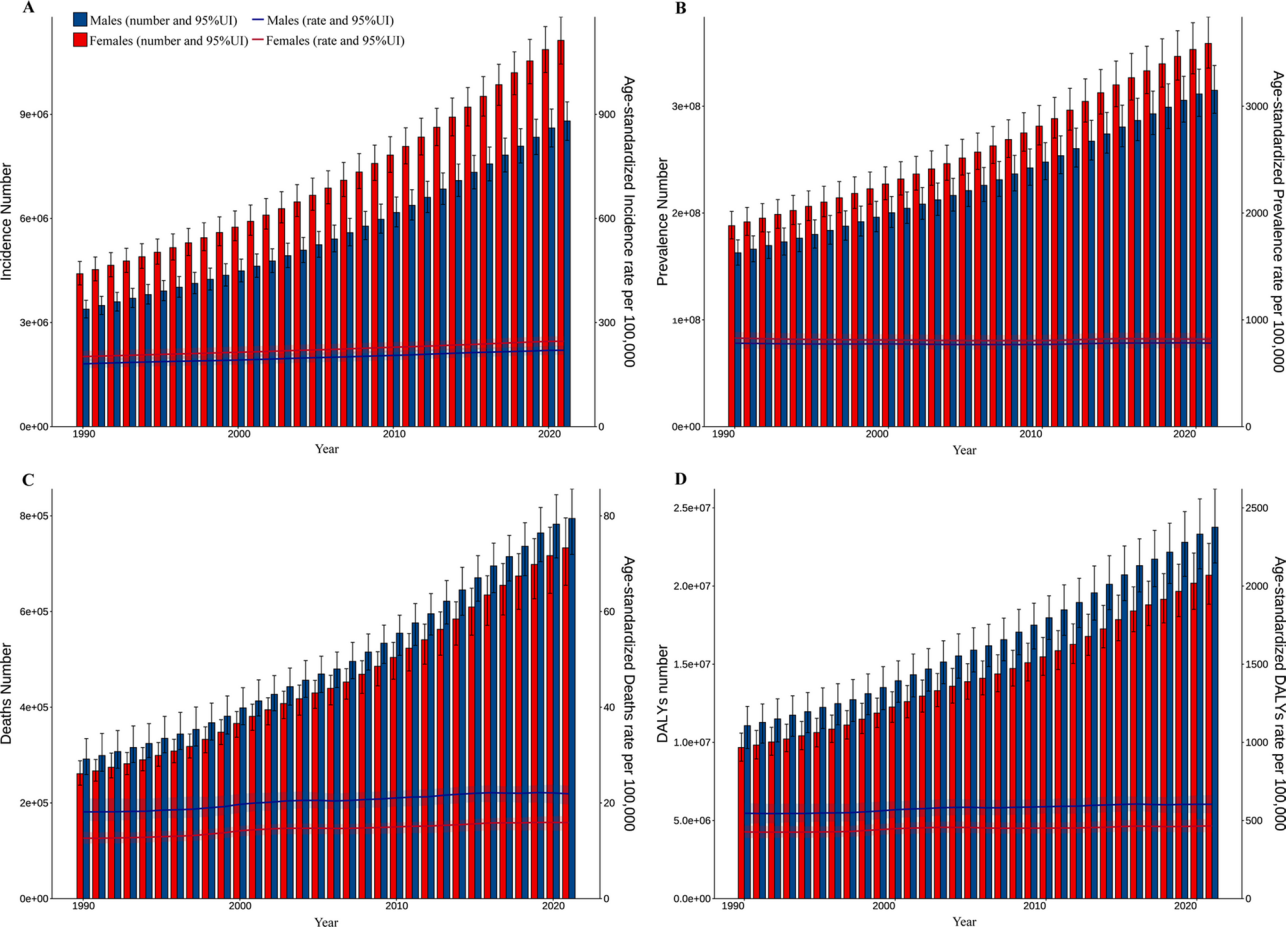
Numbers and age-standardized rates of incidence, prevalence, mortality, and DALYs for CKD by year and sex from 1990 to 2021. (**A**) Incidence; (**B**) Prevalence; (**C**) Mortality; (**D**) DALYs. Bar charts represent counts; lines represent crude rates. CKD, chronic kidney disease; DALYs, disability-adjusted life years

In terms of the incidence and prevalence of CKD, there were significantly more females than males in 2021 (Fig. 2). However, for mortality and DALYs in 2021, the number of males was more dominant (except for those aged over 80). Globally, for the incidence, mortality, and DALYs of CKD, the cases and ASRs showed an increasing trend year by year in both males and females from 1990 to 2021 (Fig. 3). However, although the cases of prevalence for CKD increased year by year, the ASPR decreased first and then increased (Fig. 3, Table S5). Moreover, across all age groups, there were more cases of incidence and prevalence in females compared to males in 2021. The incidence of CKD was mainly concentrated in the 50–84 age group, especially in the 65–69 and 70–74 age groups (Fig. 2). The ASIR also increased with age and started to decrease in people aged > 80 years. The distribution of prevalence cases was relatively scattered among different age groups. The ASPR increased with age and maintained the characteristic that the cases of females always exceeded males (Fig. 2). The cases and rates of mortality and DALYs in most age groups were higher in males compared to females, and the cases of mortality and DALYs increased first and then decreased with age. The cases of mortality were mainly concentrated in the 65–89 age group, while the cases of DALYs were mainly in the 50–84 age group (Fig. 2).

Overall, the cases of prevalence and YLDs for CKD-associated anemia showed a yearly increasing trend, with the cases of females consistently higher than males (Fig S2). The ASPR and age-standardized YLDs also showed the characteristics of more females than males, and a slight decreasing trend in the past three decades (Fig S2). Furthermore, the cases of prevalence and YLDs for CKD-associated anemia increased with age, reaching the largest in the 70–74 and 75–79 age groups, and subsequently showing a downward trend (Fig S3). The prevalence and YLDs of CKD-associated anemia were consistently higher in females compared to males across all age categories. Additionally, the ASPR and age-standardized YLDs increased with age in 2021 (Fig S3).

### Global trends by SDI

**Fig. 4:**
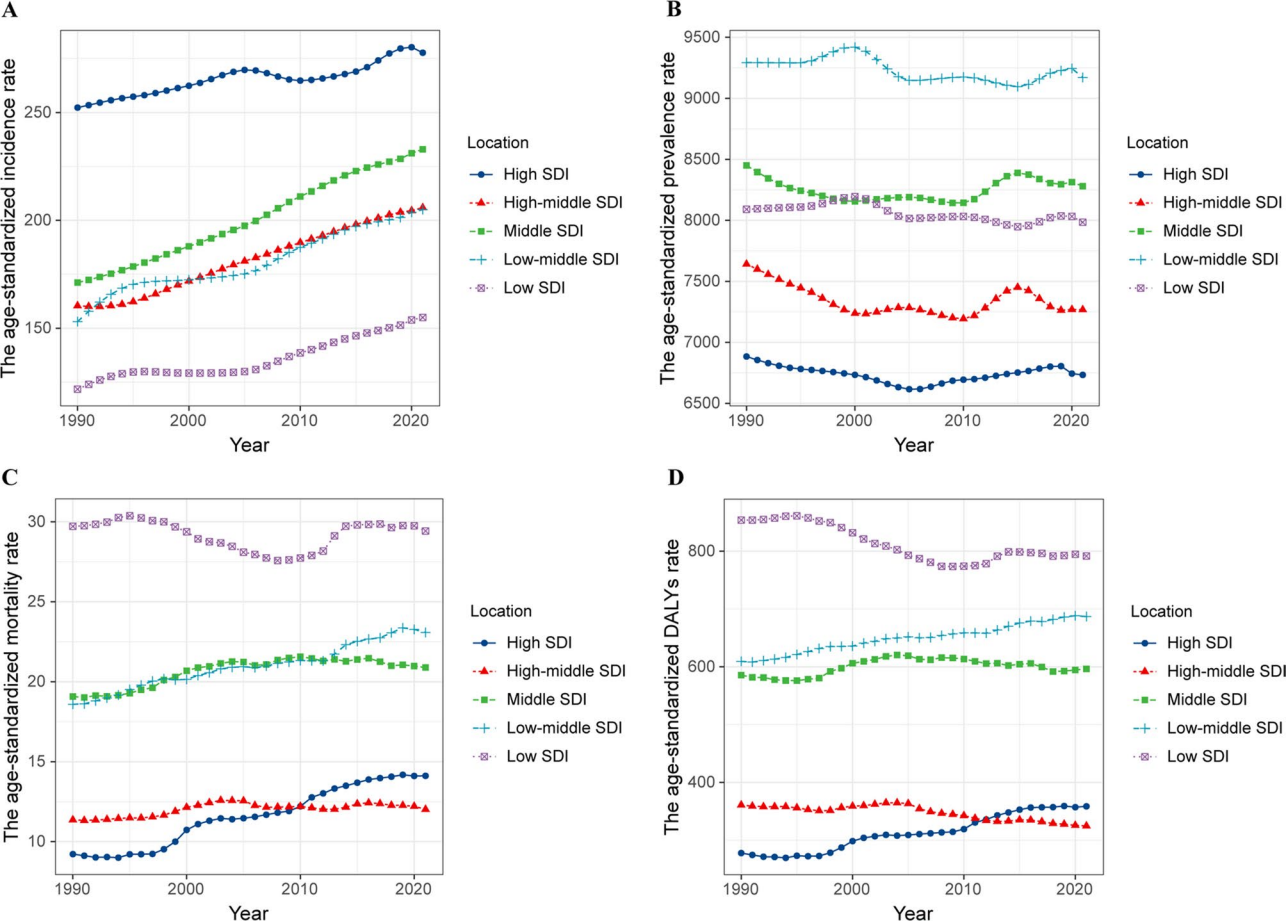
Time trends in age-standardized rates of incidence, prevalence, mortality, and DALYs for CKD from 1990 to 2021 by SDI quintile. (**A**) Incidence; (**B**) Prevalence; (**C**) Mortality; (**D**) DALYs. DALYs, disability-adjusted life years; CKD, chronic kidney disease; SDI, socio-demographic index

Compared with 1990, the cases of incidence, prevalence, mortality, and DALY for CKD in the five SDI regions increased significantly in 2021 (Table S2). From 1990 to 2021, the ASIR of CKD showed an upward trend in the five SDI regions, with the largest increase in the middle SDI region (Table S2, Fig. 4). In contrast, the ASPR of CKD continued to decline in all SDI regions (Table S6). Moreover, although the ASMR in many SDI regions showed a significant increasing trend, especially a significant rise in high SDI region (AAPC: 1.44, 95%CI: 1.16 to 1.71), there was a slight decrease in low SDI regions (Table S7). Furthermore, the age-standardized DALYs of CKD declined in high-middle SDI and low SDI regions but increased in the other SDI quintiles (Table S8, Fig. 4). In 2021, the cases of prevalence and YLDs for CKD-associated anemia had a substantial rise across all SDI regions compared to 1990 (Tables S8, S9). In all SDI regions, the ASRs of prevalence and YLDs for CKD-associated anemia showed a significant downward trend in the past three decades, with the most substantial decrease in high-middle SDI regions (Tables S8, S9, Fig S4).

### Regional and national trends

**Fig. 5:**
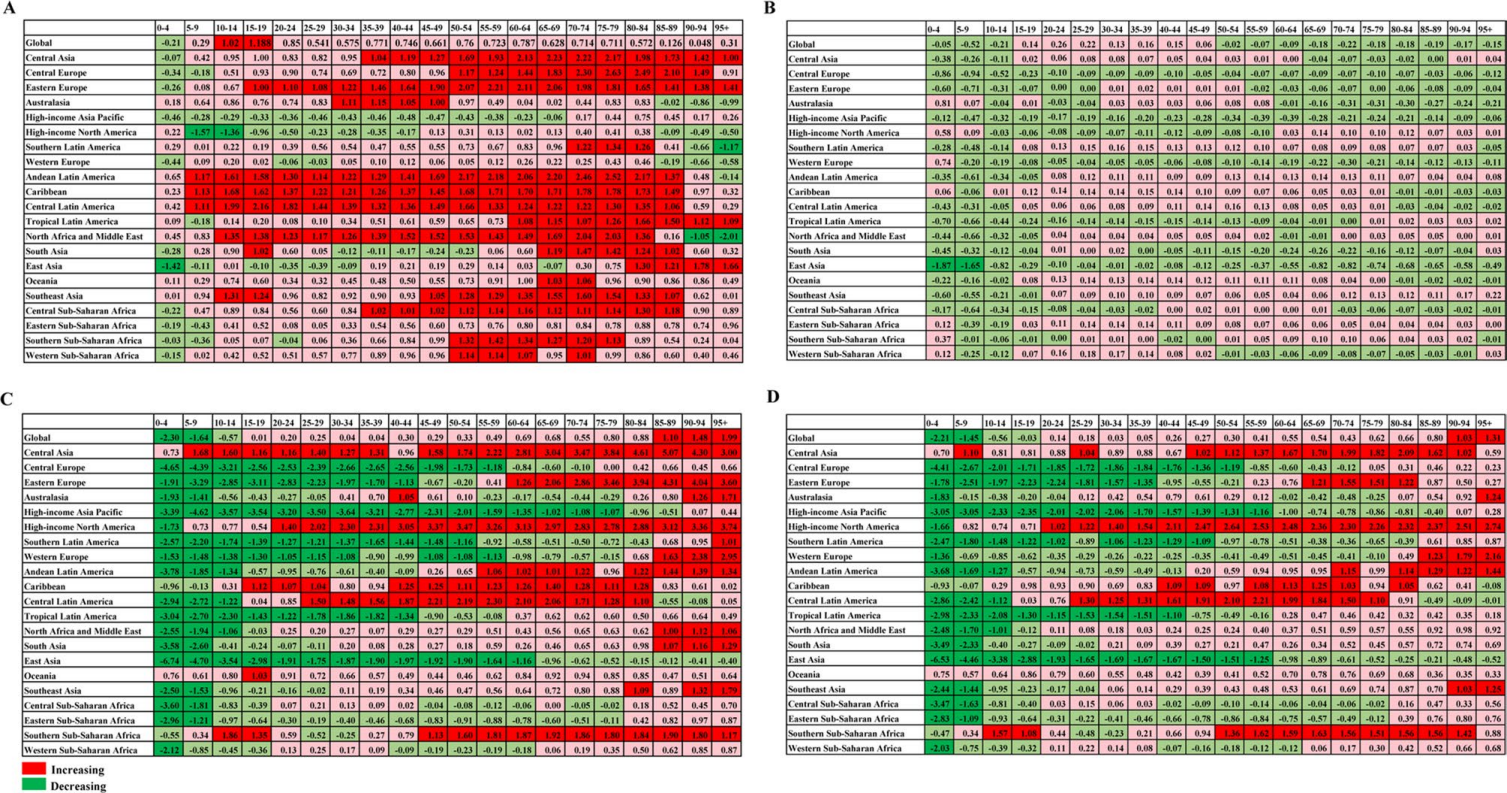
AAPC in age-specific rates of incidence, prevalence, mortality, and DALYs for CKD by age groups and regions from 1990 to 2021. (**A**) AAPC of incidence; (**B**) AAPC of prevalence; (**C**) AAPC of Mortality; (**D**) AAPC of DALYs. AAPC, average annual percentage change; DALYs, disability-adjusted life years; CKD, chronic kidney disease

**Fig. 6:**
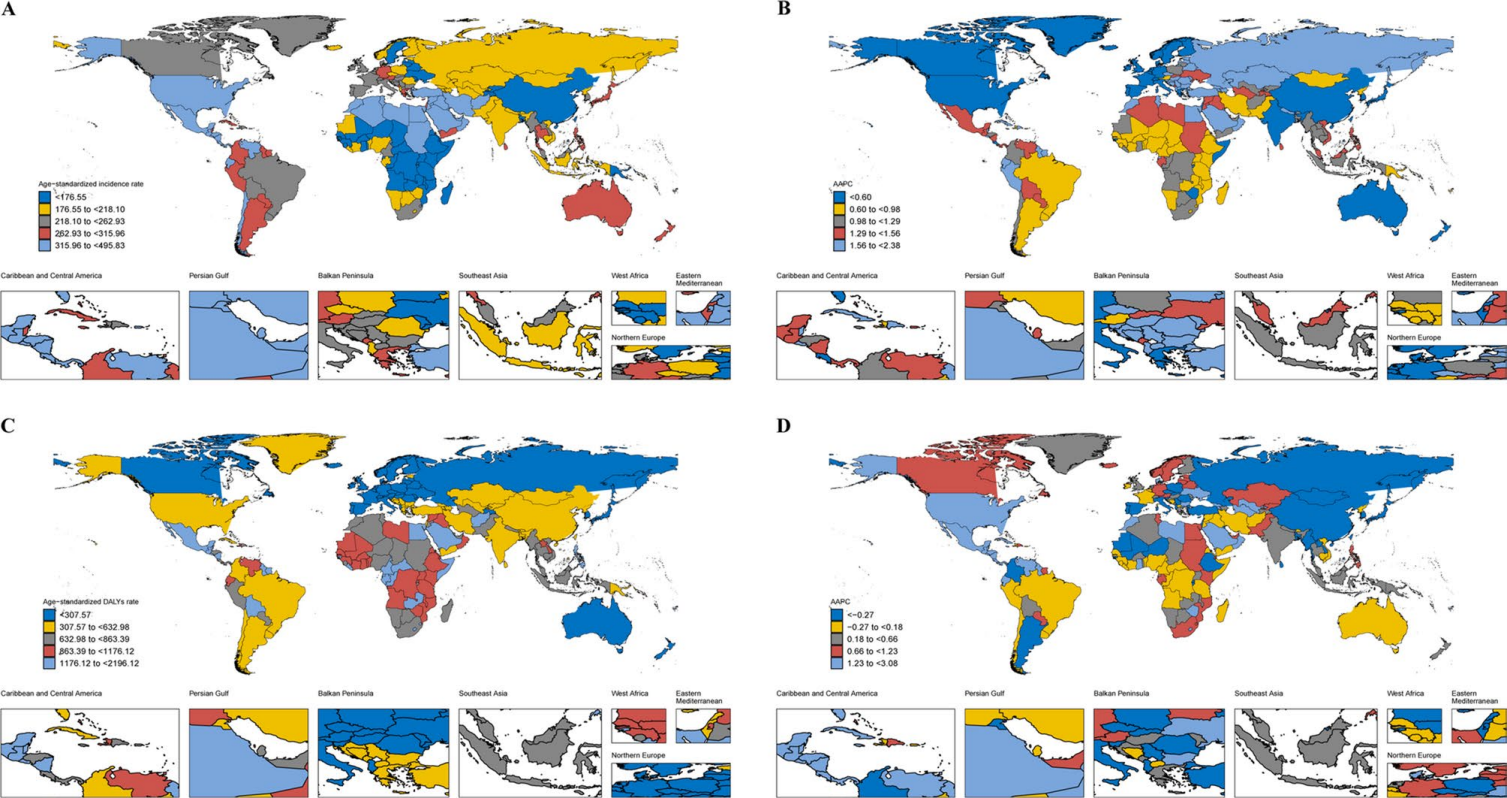
Global maps of CKD burden in 204 countries and territories. (**A**) age-standardized rate of incidence in 2021; (**B**) AAPCs in age-standardized incidence rate from 1990 to 2021; (**C**) age-standardized rate of DALYs in 2021; (**D**) AAPCs in age-standardized DALYs rate from 1990 to 2021. CKD, chronic kidney disease; AAPC, average annual percentage change; DALYs, disability-adjusted life years

**Fig. 7:**
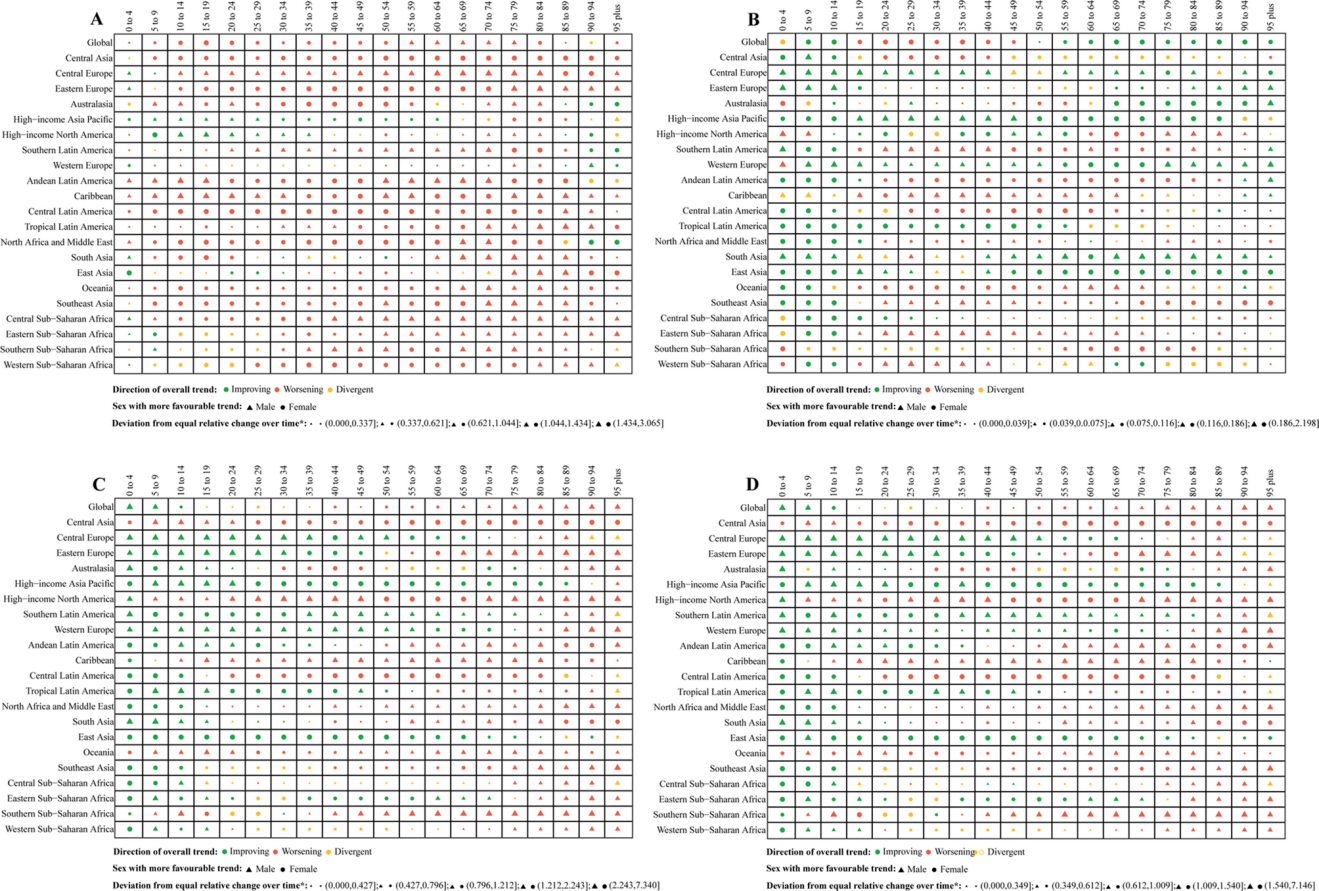
Absolute differences in non-fatal trend equality for males and females in age-standardized rates of incidence, prevalence, mortality, and DALYs for CKD from 1990 to 2021. (**A**) Incidence; (**B**) Prevalence; (**C**) Mortality; (**D**) DALYs. DALYs, disability-adjusted life years; CKD, chronic kidney disease

By geographic category, the case number of incidence, prevalence, mortality, and DALYs of CKD increased significantly in 21 regions (Table S2, Tables S6, S7). In addition, the incidence cases of CKD in most regions and age groups also increased significantly, particularly in Andean Latin America, Caribbean, and Central Latin America (Table S2, Fig. 5). In 2021, the ASIR of CKD was the highest in Saudi Arabia among 204 countries, followed by Qatar and the United Arab Emirates (Table S12, Fig. 6). Among the 204 countries, the ASIR of CKD had the most pronounced increase between 1990 and 2021 in Estonia (AAPC: 2.38, 95% CI: 2.32 to 2.43), followed by Hungary (Table S12, Fig. 6). Figure S7 showed whether females or males in the region-age combinations performed better in terms of relevant indicators over time, either by decreasing more or increasing less. Among the 420 regional-age combinations (excluding any “Global” combinations), the changes in ASIR over time were more favorable for females (56.40%) in 237 combinations, and for males (43.6%) in 183 combinations (Fig. 7). In 2021, Mauritius had the highest ASPR, while Guatemala had the largest increase between 1990 and 2021 (Table S13, Fig S5). Females had more favorable outcomes in ASPR of CKD over time in 246 combinations (58.60%) and males had more favorable outcomes in 174 combinations (41.40%) (Fig. 7). Among them, the ASMR in High-incoming North America had increased the most significantly (AAPC: 2.96, 95% CI: 2.76 to 3.16), while the ASMR in Central Sub-Saharan Africa was the highest in 2021 (43.69, 95% UI: 33.26–56.29) (Table S7). Among 204 countries, Saudi Arabia had the highest ASMR in 2021 (79.26, 95% UI: 59.76–95.73), while the ASMR in Armenia had the most pronounced increase (AAPC: 7.94, 95% CI: 5.28 to 10.67) (Table S14, Fig S5). For the ASMR of CKD, females performed better over time in 199 combinations (47.40%) compared to 221 combinations (52.60%) for males (Fig. 7). In addition, the ASR of DALYs had an increasing trend in 13 of the 21 regions, particularly in High-income North America between 1990 and 2021 (AAPC: 2.11, 95% CI: 1.88 to 2.33) (Table S2). Additionally, Central Latin America in 2021 had the highest age-standardized DALYs (1171.14, 95% UI: 1054.82–1316.26). At the national level, Mauritius had the highest ASR of DALYs in 2021 (2196.12, 95% UI: 2043.11–2318.87). The most substantial increase and decrease in ASR of DALYs were in El Salvador (AAPC: 3.08, 95% CI: 2.80 to 3.36) and Kuwait (AAPC: −2.33, 95% CI: −3.57 to −1.07), respectively (Fig. 6, Table S15). The 420 region-age combinations showed that in terms of age-standardized DALYs, females had more favorable outcomes in 202 combinations compared to 218 in males (Fig. 7). It was worth mentioning that the cases of prevalence and YLDs for CKD-associated anemia decreased across most regions and age groups, but in High-income North America, these indicators increased most significantly in the 75–79 and 80–84 age groups (Fig S6). Moreover, the ASPR of CKD-associated anemia mainly showed a downward trend in 17 of the 21 regions, and Central Asia had the highest ASPR in 2021 (1615.07, 95% UI: 1455.29–1800.63). In addition, the ASPR of CKD-associated anemia had the most pronounced decline in East Asia (AAPC: −1.62, 95% CI: −1.69 to −1.55), followed by Andean Latin America (AAPC: −1.06, 95% CI: −1.09 to −1.03) (Table S8). At the national level, the decline in Republic of Korea (AAPC: −2.57, 95% CI: −2.61 to −2.53) and China (AAPC: −1.67, 95% CI: −1.74 to −1.60) was even more pronounced (Table S16, Fig S7). Among the 420 regional-age combinations (excluding the “Global”), females had 161 combinations (38.30%) that were more favorable in terms of ASPR over time, compared with 259 combinations (61.70%) for males (Fig S8). In almost all regions, the age-standardized YLDs showed a decreasing trend between 1990 and 2021. Among them, the age-standardized YLDs in South Asia were the highest in 2021, and the decline was most significant in East Asia (Table S9). Meanwhile, females had favorable outcomes in 51 combinations compared to 369 for males (Fig S8). Moreover, Nepal had the highest age-standardized YLDs in 2021 (84.28, 95% UI: 54.64–123.86), while Republic of Korea had the most pronounced decline in age-standardized YLD from 1990 to 2021 (AAPC: −2.89, 95% CI: −2.95 to −2.83) (Table S17, Fig S7).

### Decomposition analysis

**Fig. 8:**
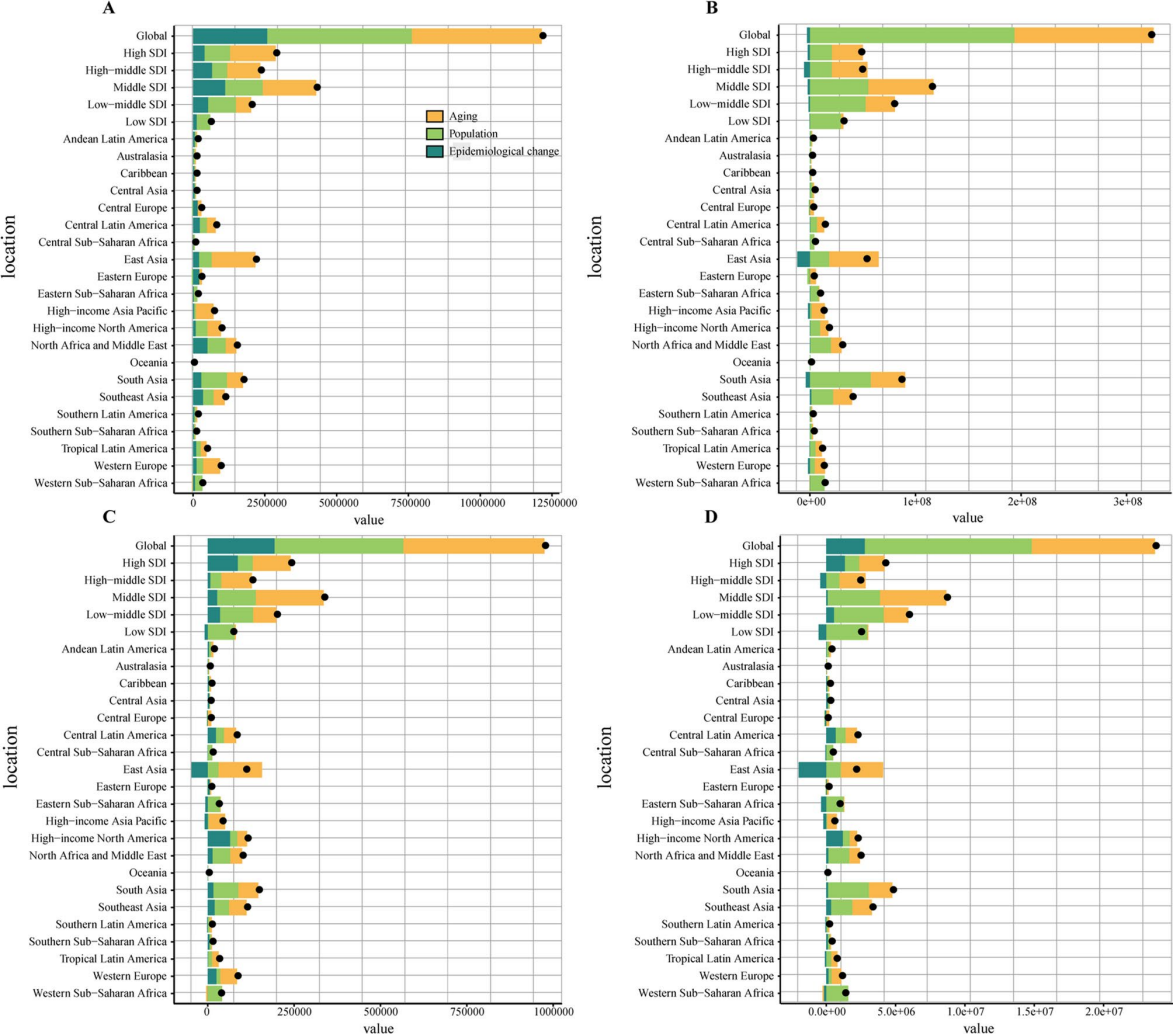
Changes in incidence, prevalence, mortality, and DALYs of CKD according to aging, population growth, and epidemiological change from 1990 to 2021 at the global level by SDI quintile and various regions. (**A**) Incidence; (**B**) Prevalence; (**C**) Mortality; (**D**) DALYs. The black dots indicate the total value of change attributable to all three components. DALYs, disability-adjusted life years; CKD, chronic kidney disease; SDI, socio-demographic index

The decomposition analysis of this study presented the changes in correlation indicators of CKD and its associated anemia attributed to three population-level factors (aging, population growth, and epidemiological changes) based on SDI quintiles and 21 regions. Globally, the incidence, prevalence, mortality, and DALYs of CKD increased substantially, with the largest increase in the middle SDI regions (Fig. 8). The effects of population growth were most pronounced in low-middle SDI and low SDI regions, with the largest impact of population aging in the other SDI regions. Among the 21 regions, East Asia exhibited the highest incidence, mainly due to aging followed by population growth. The prevalence, mortality, and DALYs increased in East Asia from 1990 to 2021, with aging being the main factor followed by population growth, while epidemiologic change showed the opposite effect (Fig. 8). Meanwhile, the increase in prevalence, mortality, and DALYs was most pronounced in South Asia, with population growth having the greatest impact, followed by aging. For CKD-associated anemia, although the contribution of epidemiologic change to prevalence and YLDs was negative in all SDI regions, the prevalence and YLDs increased during the study period (Fig S9). The effects of population growth for CKD-associated anemia prevalence and YLDs were most pronounced in low-middle SDI and low SDI regions, with the largest impact of population aging in the other SDI regions. In addition, the prevalence and YLDs of CKD-associated anemia increased in East Asia, but the epidemiological change had a converse impact, reducing the burden of the above indicators. Furthermore, the increase in prevalence and YLDs was most pronounced in South Asia, which was mainly due to population growth, followed by aging, despite a downward trend in epidemiological changes (Fig S9).

### Predictions of CKD and its associated anemia burden trends to 2050

**Fig. 9:**
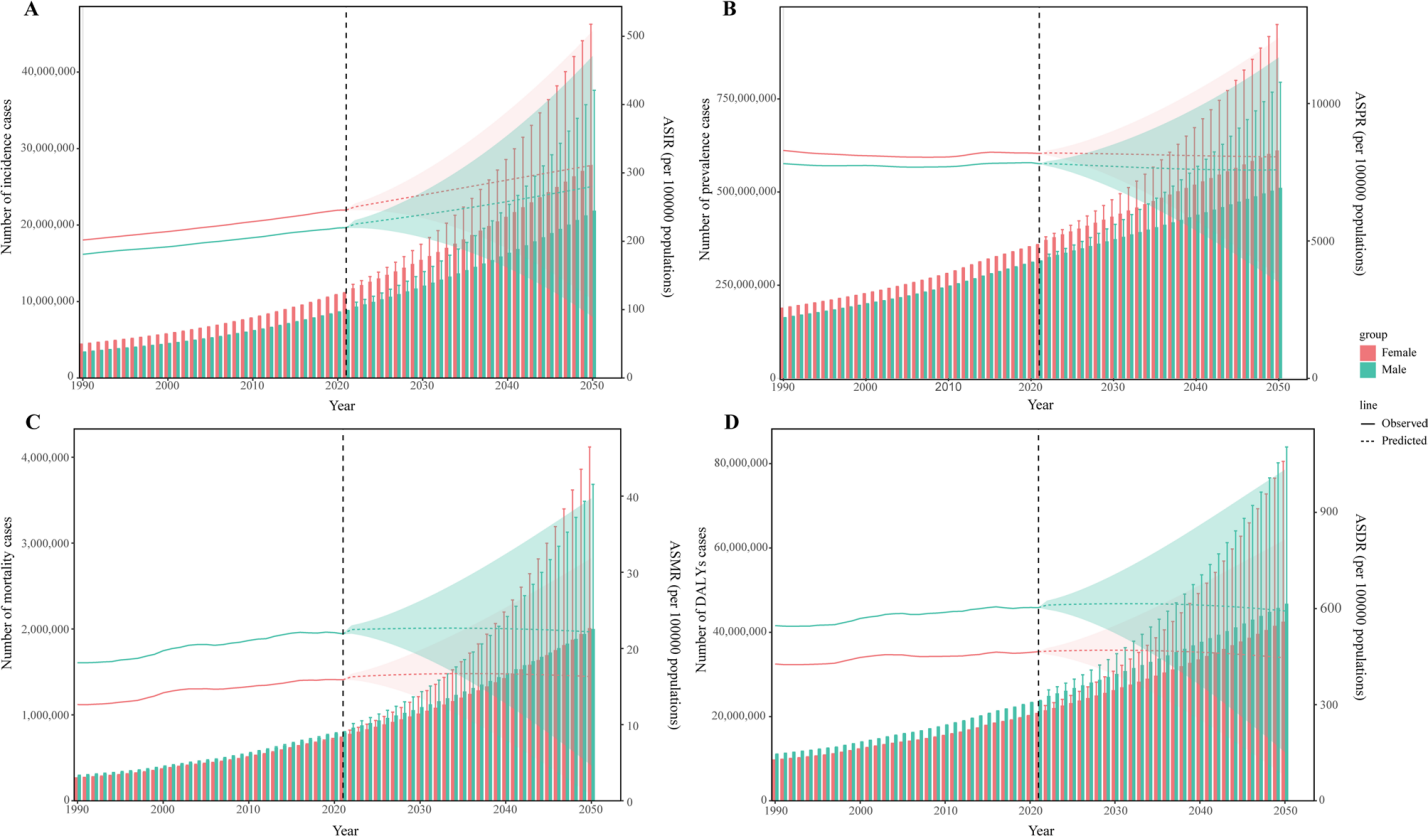
Prediction of CKD in number and age-standardized rate of incidence, prevalence, mortality, and DALYs. (**A**) Incidence; (**B**) Prevalence; (**C**) Mortality; (**D**) DALYs. CKD, chronic kidney disease; DALYs, disability-adjusted life years

The cases of incidence, prevalence, mortality, and DALYs for CKD were projected to continue to increase globally by 2050 in both sexes, as well as in the cases of prevalence and YLDs for its associated anemia (Fig. 9, Fig S10). In addition, the ASIR of CKD was on the rise from 2022 to 2050 and the ASPR declined slightly. The ASRs of mortality and DALYs for CKD remained relatively stable during the predicted period (Fig. 9). Furthermore, the ASRs of prevalence and YLDs for CKD-associated anemia in females exhibited a substantial rising trend between 2022 and 2050. In contrast, the ASR of YLDs for CKD-associated anemia decreased significantly in males (Fig S10).

## Discussion

Based on GBD 2021, this study presented the most up-to-date and comprehensive evaluations of CKD incidence, prevalence, mortality, and DALYs (including the prevalence and YLDs of its associated anemia) at global and regional scales. Due to its long course, poor prognosis, many complications, and high cost of treatment, chronic kidney disease poses a major challenge to health care worldwide. From 1990 to 2021, the number of people with CKD has risen from more than 300 million to nearly 700 million, significantly higher than the number of people with diabetes, COPD, and asthma [34]. Although population aging and the prevalence of risk factors are the main driving forces behind the increase in prevalence, the continuous expansion of CKD diagnostic criteria (established by the KDOQI guidelines in 2002, and included albuminuria as a core diagnostic criterion in 2012, significantly expanding the population eligible for CKD diagnosis, and further expanding the definition in the KDIGO update in 2024) is an important non disease incidence factor leading to an increase in statistical prevalence (i.e., “diagnostic inflation”), especially after 2002. Therefore, although the real growth driven by population aging, prevalence of risk factors, and other factors remains the main driving force, the evolution of CKD definition is a confounding factor that can’t be ignored when analyzing long-term trends. Moreover, In the past few decades, the cases and ASRs of incidence, mortality, and DALYs for CKD have shown a significant increase.

Globally, the cases of incidence, prevalence, mortality, and DALYs for CKD have increased dramatically in the past few decades, placing a large burden on the healthcare system. The accelerating trend of global population aging may also contribute to the increased incidence of CKD. Our study found that in contrast to the other SDI regions, the CKD mortality and DALYs were higher in the low SDI and low-middle SDI regions, and that early diagnosis and optimal management for metabolic diseases (e.g., diabetes and hypertension) and cardiovascular diseases were often elusive in low-income Settings [35, 36]. The mentioned diseases above are often important factors for the occurrence and progression of CKD. Moreover, due to ignorance of the condition and consideration of the cost of drugs, many people tend to use herbs that are considered cheap and safe, which may lead to the delay of the condition, and some herbs have strong renal toxicity, which promotes the further deterioration of the condition [37, 38]. Renal replacement therapy is a big shock to the fragile healthcare system in low and low-middle SDI regions. Studies have shown that the prevalence of renal replacement therapy varies by 200 times between high-income regions (e.g., Chinese Taiwan) and low-income regions (e.g., sub-Saharan African countries) [39]. Due to cultural, religious, and educational differences, along with organ shortages, and economic constraints, approximately 90.00% of kidney replacement therapy patients in low- and middle-income nations and 96.00% of renal failure patients in low-income countries lacked the opportunity for treatment, which in part contributes to the increase of CKD mortality [40]. Therefore, in low-income areas, the government should not only strengthen health education activities, but also increase more investment in medical resources and optimize the early screening of metabolic diseases to cope with the increasing disease burden of CKD. In addition, in high SDI region, our research found that the ASRs of mortality and DALY for CKD increased most significantly. This might be attributed to the rapid advancement of the social economy, shifts in lifestyle, and changes in dietary patterns that have led to the rapid increase of metabolic diseases (e.g., diabetes and hypertension) and cardiovascular diseases in high-income regions, which have become the major contributors to CKD progress [41]. Furthermore, poor awareness and prevention of CKD contribute to the failure of many patients to receive timely and appropriate treatment in some high-income regions. Studies have found a significant increase in the global crude prevalence of CKD categories G3–G5 from 2.80% in 1990 to 3.70% in 2016, which also reflected the increasing trend of CKD mortality and DALYs [21]. Although the prevalence and YLDs of CKD-associated anemia have significantly increased between 1990 and 2021, the ASPR and age-standardized YLDs showed a downward trend, probably due to changes in population structure and improvements in treatment methods, reflecting to some extent the progress made in the prevention, treatment, and management of CKD-associated anemia in the global health field. Therefore, enhancing the comprehension and self-management capacity of CKD, understanding the risk factors and preventive measures of CKD and its associated anemia, and rational use of health resources are essential to improve the overall health of the population and strengthen the capacity to respond to public health emergencies. For example, for patients with metabolic diseases, the government can implement mandatory annual kidney screening to diagnose high-risk patients. In addition, integrating CKD prevention into the national chronic disease strategy can achieve the goal of prevention. Of course, providing remote monitoring (uploading home blood pressure/blood glucose) and AI warning system for high-risk CKD patients in the early stage can timely monitor changes in the patient’s condition.

Globally, our study suggested females occupied a more significant position in the cases of incidence and prevalence for CKD compared with males from 1990 to 2021. This phenomenon also applied to the prevalence and YLDs of CKD-associated anemia. As for the mortality and DALYs of CKD, the number of males was more dominant, which might be because males are more susceptible to major chronic diseases (such as diabetes, hypertension, etc.) or have obvious risk factors for CKD (such as smoking) [42–44]. In addition, the protective capacity of estrogen and the destructive impact of testosterone may play important roles in making kidney disease worse in males compared to females [45–47]. In 2021, the age distribution of CKD-related mortality tended to increase gradually with age, with middle-aged and elderly people accounting for the major proportion of CKD mortalities. Based on this, the detection of serum creatinine and eGFR can be included in the fall risk assessment of elderly men. Of course, the pre-retirement kidney audit system and the elderly label of nephrotoxic drugs are particularly important for the prevention and treatment of CKD in elderly men.

Regionally and nationally, the cases of incidence, prevalence, mortality, and DALYs for CKD increased significantly in 21 regions from 1990 to 2021, indicating that the disease burden of CKD was still substantial. Sub-Saharan Africa contains about 70% of the world’s least developed countries, with a per capita GDP of below $1,500 and approximately half the population subsists below $1 a day [48]. This extreme poverty, the growing prevalence of non-communicable diseases (e.g., hypertension and diabetes), and the persistent burden of infectious diseases (e.g., HIV & AIDS and hepatitis B) make local health care difficult [48, 49]. Furthermore, the implementation of renal replacement therapies, including hemodialysis and kidney transplantation, faces significant challenges in these regions [39, 50]. Even worse, these regions and countries lack effective registers of information on CKD, and it can be inferred that the disease burden of CKD in these regions is more complex than what we currently know [51]. Interestingly, the increase in ASRs of mortality and DALYs between 1990 and 2021 was most pronounced in high-income North America, although the cases of mortality weren’t the highest, which may be attributed to changes in population structure and the impact of underlying diseases. Certainly, these regions with significant increases need to be aware of the impending burden of CKD to avert a cascade of potential public health problems that may arise from inadequate preparation. For CKD-associated anemia, our results showed that the disease burden in South Asia (such as Nepal, Pakistan, etc.) was heavier in 2021, while the disease burden in East Asia (including South Korea, China, etc.) decreased most significantly from 1990 to 2021. The findings can primarily be attributed to the lower economic conditions, lack of health awareness, and more environmental risk factors (such as malaria and schistosomiasis) in South Asia [52, 53]. Therefore, in South Asia, the treatment of diseases such as schistosomiasis and malaria should be synchronized with the detection of serum creatinine to detect CKD at an early stage. In addition, the government should strengthen health education to enhance residents’ awareness of CKD, and increase medical expenditures and preventive screenings to alleviate the economic burden on residents. In contrast, the improvement of living standards and enhanced health awareness enable more patients in East Asia to be diagnosed and treated early, thereby reducing the prevalence and YLDs of CKD-associated anemia. The combined effect of the above factors led to the epidemiological differences of CKD-associated anemia in different regions.

However, our study still has certain unavoidable limitations. First, the GBD data applied has a certain time lag. Second, owing to the difficulty of accessing healthcare services, the disease burden of CKD might be underestimated in certain low-income regions and countries. Third, determining the etiology of CKD is quite challenging, although kidney biopsy is recognized evidence for the etiology of CKD. However, the choice of this method needs to be cautious and should only be considered when determining treatment plans or assessing surgical risks. Fourth, our data source was cross-sectional, which may lead to bias and confounding. Limitations of the GBD framework include reliance on data quality in low-resource settings (where extrapolation may bias estimates), potential model misspecification affecting interval accuracy, incomplete capture of systematic biases within uncertainty intervals, and philosophical debates around aggregating DALYs across diverse severity levels. Furthermore, recent major breakthroughs in the field of CKD treatment (particularly SGLT2 inhibitors and finerenone) and the improvement of SDI may cause the prediction of CKD disease burden to deviate from future reality, which increases the uncertainty of prediction. Despite these limitations, this research provides a new understanding of the disease burden of CKD and its associated anemia worldwide.

## Conclusions

In summary, the disease burden of CKD and its associated anemia worldwide has gradually increased, although the ASRs of its associated anemia has been decreasing over the past three decades. Moreover, there are notable disparities in the disease burden of CKD and its associated anemia across various regions, countries, and SDIs. Meanwhile, the burden of CKD and its associated anemia is predicted to rise until 2050, primarily attributed to population growth and aging. In the future, it is necessary to collect more detailed regional data to conduct in-depth analysis of differences and explore effective strategies for integrating primary and secondary prevention. In addition, using a longitudinal study design to evaluate the long-term effects and sustainability of intervention measures can enhance the prospective guidance value of the research.

## Electronic supplementary material

Below is the link to the electronic supplementary material.

Supplementary Material 1

Supplementary Material 2

## Data Availability

Data are available on the Global Health Data Exchange GBD 2021 website (https://vizhub.healthdata.org/gbd-results/).
