# Supplementary material for: Global, regional, and national burden of chronic kidney disease and its associated anemia, 1990 to 2021 and predictions to 2050: an analysis of the global burden of disease study 2021
Source: BMC Nephrol. 2025 Aug 27;26:495. doi: 10.1186/s12882-025-04398-4 (PMC12382167; doi:10.1186/s12882-025-04398-4)
Supplement: Supplementary file 1 — Supplementary Material 1 [file 12882_2025_4398_MOESM1_ESM.pdf]

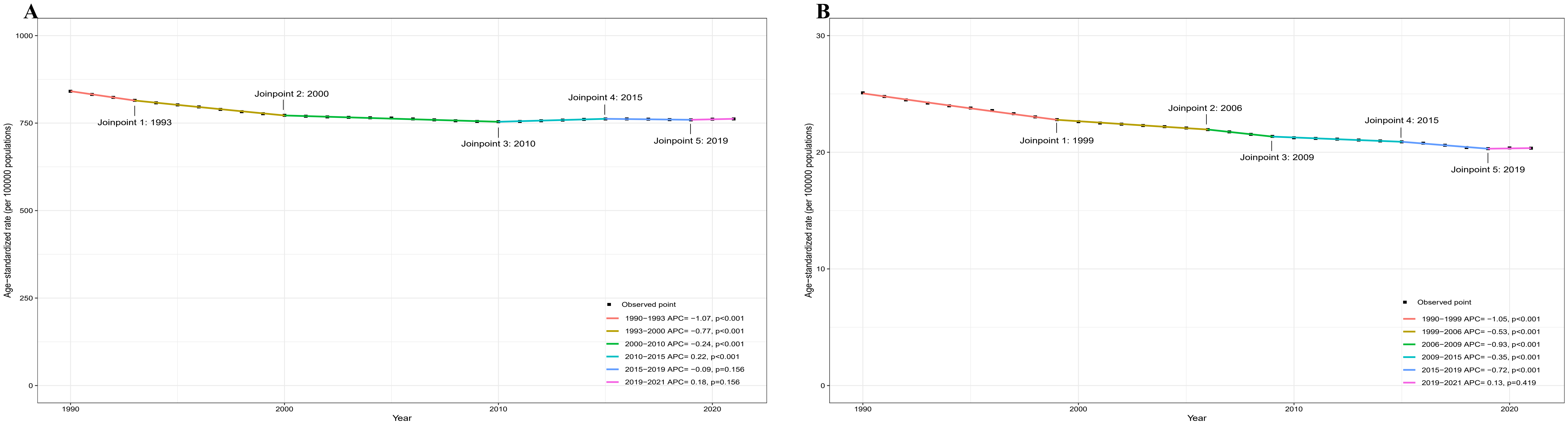

Fig S1. Joinpoint regression analysis of the age-standardized rate of prevalence and YLDs for CKD-associated anemia from 1990 to 2021. (A) Prevalence; (B) YLDs. YLDs, years lived with disability; CKD, chronic kidney disease.

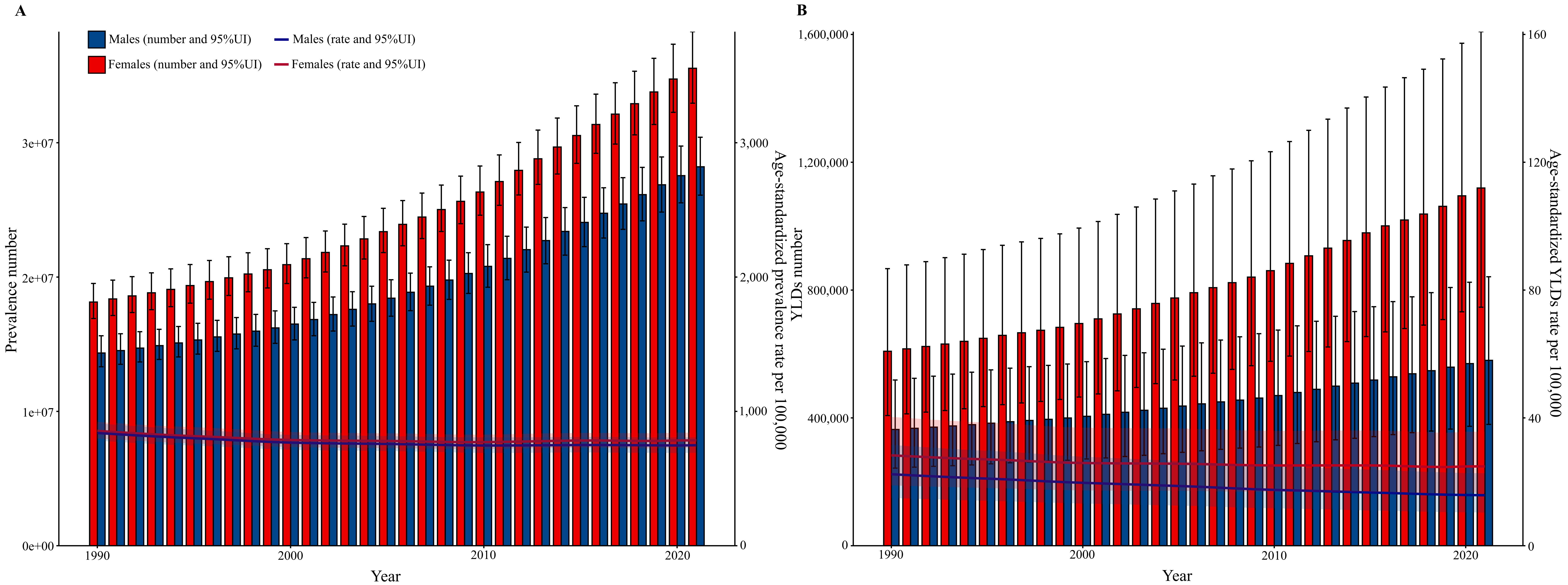

Fig S2. Numbers and age-standardized rates of prevalence and YLDs for CKD-associated anemia by year and sex from 1990 to 2021. (A) Prevalence; (B) YLDs. Bar charts represent counts; lines represent crude rates. YLDs, years lived with disability; CKD, chronic kidney disease.

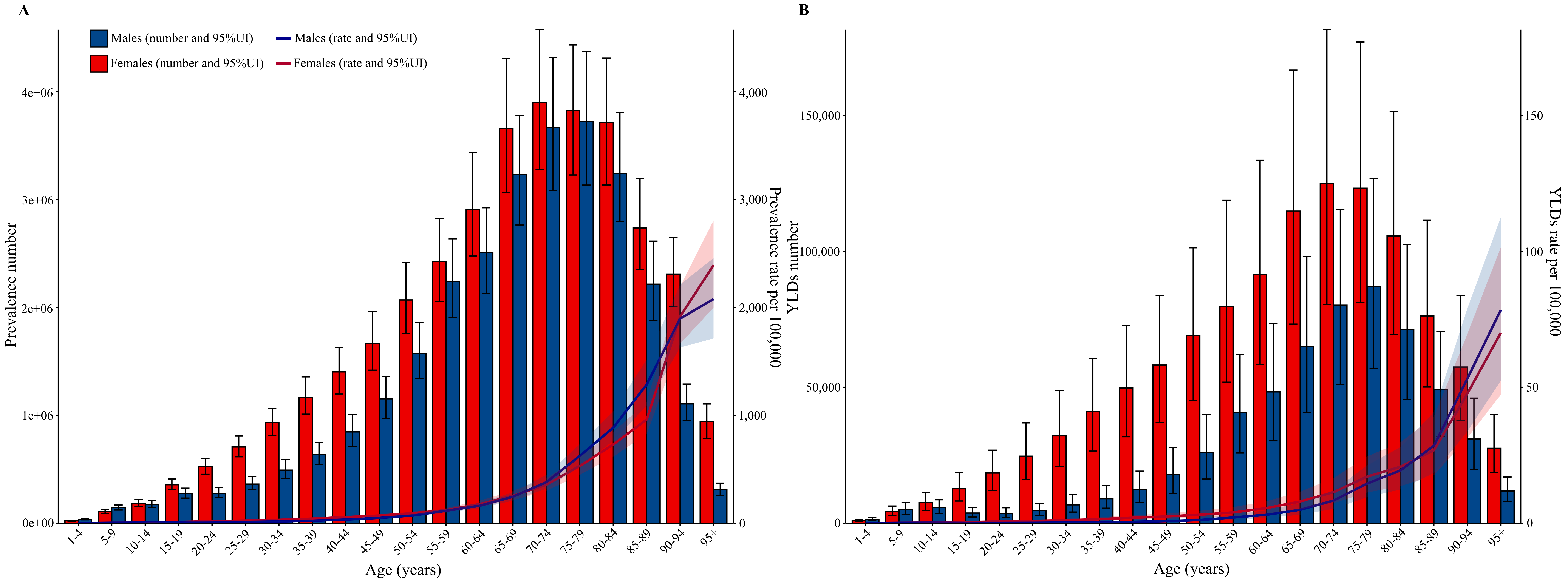

Fig S3. Numbers and age-standardized rates of prevalence and YLDs for CKD-associated anemia by age group and sex in 2021. (A) Prevalence; (B) YLDs. Bar charts represent counts; lines represent crude rates. YLDs, years lived with disability; CKD, chronic kidney disease.

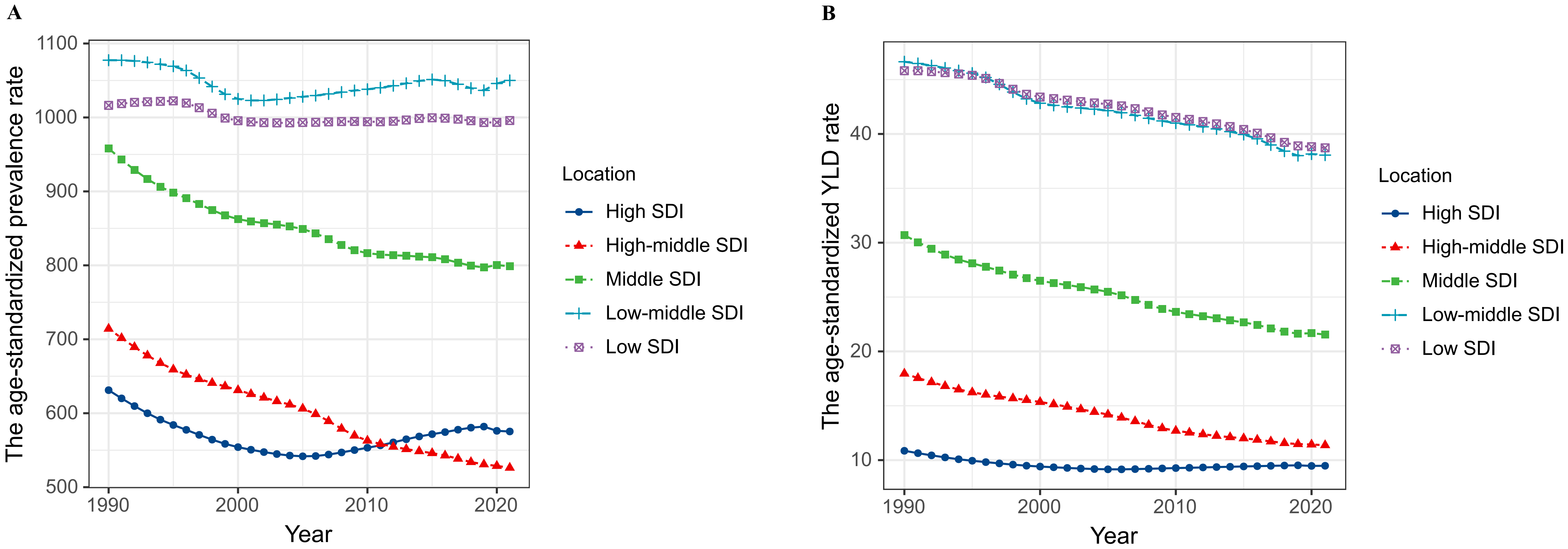

Fig S4. Time trends in age-standardized rates of incidence and YLDs for CKD-associated anemia from 1990 to 2021 by SDI quintile. (A) Prevalence; (B) YLDs. YLDs, years lived with disability; CKD, chronic kidney disease; SDI, socio-demographic index.

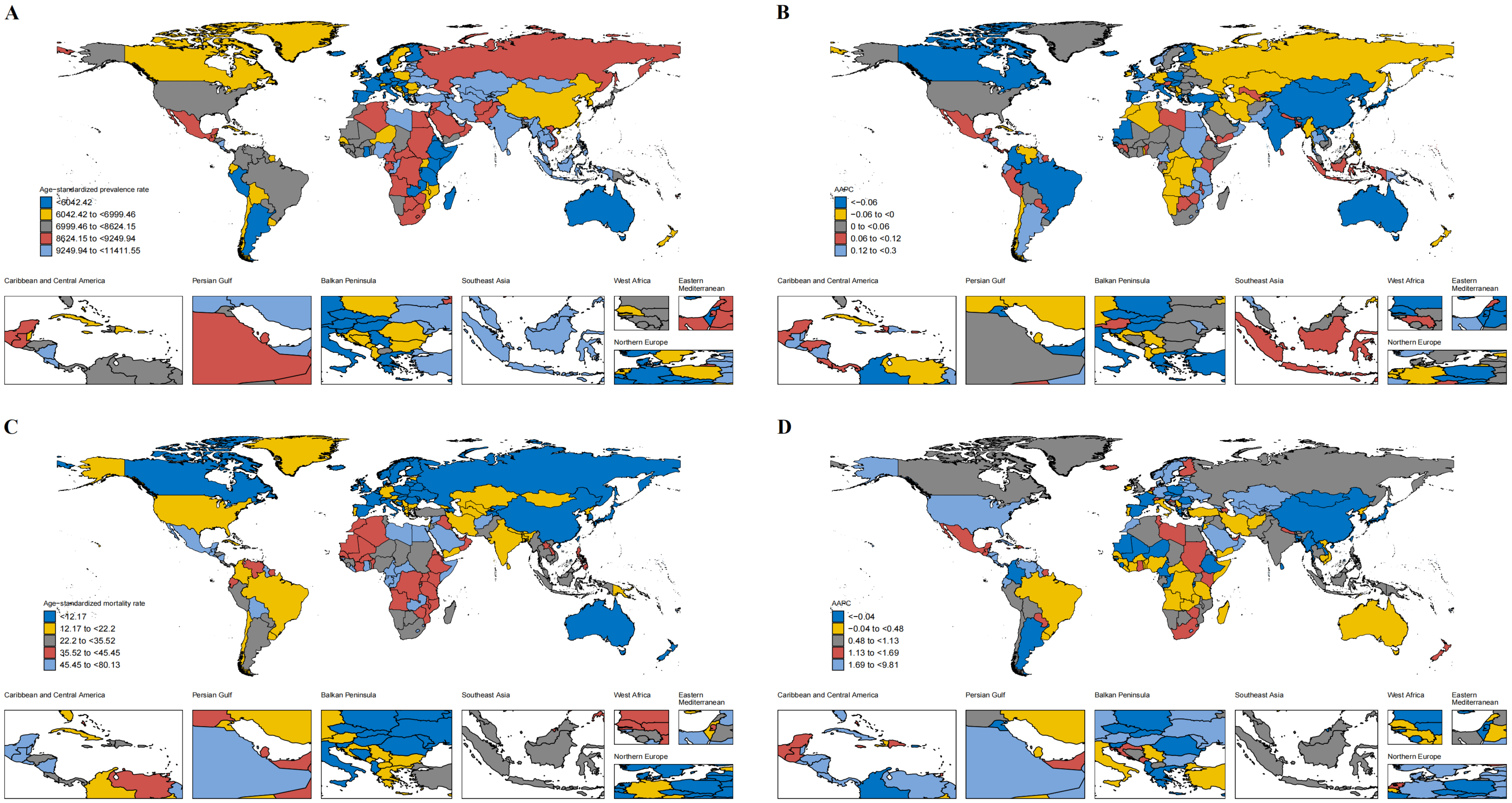

Fig S5. Global maps of age-standardized rate of prevalence and mortality for CKD. (A) Age-standardized rate of prevalence in 2021; (B) AAPCs in age-standardized prevalence rate from 1990 to 2021; (C) Age-standardized rate of mortality in 2021; (D) AAPCs in age-standardized mortality rate from 1990 to 2021. CKD, chronic kidney disease; AAPC, average annual percentage change.

A

	0-4	5-9	10-14	15-19	20-24	25-29	30-34	35-39	40-44	45-49	50-54	55-59	60-64	65-69	70-74	75-79	80-84	85-89	90-94	95+
Global	-0.97	-0.84	-0.54	-0.41	-0.50	-0.49	-0.58	-0.44	-0.41	-0.65	-0.65	-0.61	-0.42	-0.40	-0.28	-0.05	-0.02	-0.14	-0.18	-0.23
Central Asia	-0.65	-0.55	-0.63	-0.33	-0.26	-0.20	-0.16	-0.19	-0.17	-0.05	-0.03	0.10	0.00	0.06	0.59	0.37	0.00	0.06	-0.17	0.55
Central Europe	-1.48	-2.16	-1.95	-1.18	-1.37	-1.37	-1.16	-0.84	-0.96	-0.78	-0.71	-0.59	-0.11	0.06	-0.48	-0.20	0.23	0.43	-0.04	0.17
Eastern Europe	-1.23	-1.59	-1.86	-1.31	-1.17	-1.09	-1.02	-0.84	-0.83	-0.69	-0.54	-0.29	-0.43	-0.21	0.15	0.00	-0.41	-0.38	-0.39	0.36
Australasia	-1.16	-1.79	-1.51	-0.98	-0.95	-0.96	-0.88	-0.79	-0.55	-0.62	-0.56	-0.58	-0.85	-1.03	-0.98	-0.71	-0.43	-0.24	-0.36	-0.24
High-income Asia Pacific	-1.96	-2.50	-2.49	-2.20	-2.28	-2.36	-2.05	-1.86	-1.83	-2.04	-2.12	-1.84	-1.85	-1.44	-1.02	-0.71	-0.54	-0.32	-0.20	0.29
High-income North America	-0.82	-0.38	-0.03	-0.31	-1.18	-1.06	-0.55	-0.43	-0.38	-0.44	0.29	0.38	0.13	0.26	0.38	1.18	1.15	0.28	0.06	-0.49
Southern Latin America	-1.06	-2.52	-2.71	-1.66	-1.59	-1.57	-1.65	-1.31	-1.30	-1.06	-1.69	-1.33	-1.35	-0.96	-0.40	-0.04	0.05	0.21	-0.06	-0.20
Western Europe	-1.22	-2.01	-2.32	-2.03	-1.47	-1.37	-1.29	-1.24	-1.09	-1.47	-1.30	-1.33	-1.38	-1.37	-1.24	-0.75	-0.48	-0.26	-0.29	-0.18
Andean Latin America	-1.27	-2.41	-2.41	-1.32	-2.44	-1.83	-1.57	-1.63	-2.04	-2.48	-1.48	-1.54	-1.63	-1.23	-0.90	-0.32	-0.10	-0.25	-0.27	-0.56
Caribbean	-0.05	-0.23	0.00	0.00	-0.02	-0.09	-0.04	0.00	0.31	-0.10	0.06	0.23	-0.05	-0.17	-0.23	-0.23	-0.29	-0.27	-0.02	0.40
Central Latin America	-0.68	-1.16	-1.34	-1.16	-1.33	-1.03	-0.91	-0.91	-0.91	-0.91	-0.54	-0.48	-0.34	-0.22	0.01	0.09	0.02	-0.03	-0.08	0.08
Tropical Latin America	-0.81	-1.40	-1.85	-1.55	-1.72	-1.70	-1.75	-1.59	-1.48	-1.28	-0.96	-0.69	-0.82	-0.82	-0.79	-0.73	-0.66	-0.35	-0.74	-0.74
North Africa and Middle East	-1.06	-1.84	-2.03	-1.49	-1.46	-1.25	-1.11	-1.18	-1.06	-1.00	-0.86	-0.69	-0.68	-0.43	-0.22	0.10	0.12	0.17	0.21	0.55
South Asia	-0.31	-0.17	-0.28	0.02	-0.10	-0.11	-0.08	-0.03	-0.13	-0.21	-0.27	-0.17	-0.19	-0.03	0.04	-0.18	0.07	-0.15	-0.35	-0.46
East Asia	-3.51	-4.44	-4.52	-4.31	-3.92	-3.68	-3.39	-3.17	-2.80	-2.93	-2.69	-2.50	-2.25	-2.22	-1.57	-1.12	-0.59	-0.20	-0.41	-0.21
Oceania	-0.19	-0.06	-0.46	-0.51	-0.26	-0.14	-0.12	-0.06	0.02	-0.06	0.20	0.16	0.24	0.27	0.29	0.27	0.25	0.15	-0.06	-0.11
Southeast Asia	-2.42	-2.38	-1.91	-1.33	-1.27	-1.23	-1.12	-1.06	-0.93	-0.86	-0.59	-0.43	-0.36	-0.05	0.38	0.42	0.43	0.36	0.31	0.23
Central Sub-Saharan Africa	-0.82	-1.49	-0.66	-0.61	-0.60	-0.40	-0.34	-0.32	-0.21	-0.39	-0.21	-0.17	-0.18	-0.06	0.02	0.04	0.07	0.10	0.46	0.39
Eastern Sub-Saharan Africa	-0.38	-0.45	-1.10	-0.48	-0.61	-0.70	-0.68	-0.56	-0.39	-0.22	-0.24	-0.07	-0.24	-0.10	-0.15	-0.07	-0.05	0.18	0.08	-0.07
Southern Sub-Saharan Africa	-0.02	-0.15	-0.34	-0.64	-0.87	-0.93	-0.80	-0.69	-0.62	-0.33	-0.15	-0.33	-0.09	0.03	-0.17	-0.13	0.26	0.02	-0.12	0.12
Western Sub-Saharan Africa	-0.40	-0.27	-0.03	0.02	-0.17	-0.15	-0.07	-0.10	-0.11	-0.07	0.14	0.35	0.25	0.02	0.02	0.09	0.12	0.10	-0.11	-0.16

Increasing

Decreasing

B

	0-4	5-9	10-14	15-19	20-24	25-29	30-34	35-39	40-44	45-49	50-54	55-59	60-64	65-69	70-74	75-79	80-84	85-89	90-94	95+
Global	-1.41	-1.11	-0.83	-0.62	-0.71	-0.64	-0.82	-0.71	-0.72	-0.99	-1.02	-1.00	-0.88	-0.82	-0.75	-0.31	-0.38	-0.49	-0.50	-0.38
Central Asia	-1.33	-1.04	-0.95	-0.79	-0.59	-0.49	-0.44	-0.44	-0.34	-0.45	-0.30	-0.31	-0.41	-0.51	-0.40	-0.21	-0.20	-0.19	-0.60	-0.73
Central Europe	-2.79	-2.84	-2.43	-2.18	-2.00	-1.87	-1.68	-1.51	-1.30	-1.13	-1.16	-1.23	-0.91	-0.74	-0.80	-0.65	-0.38	-0.41	-0.75	-0.82
Eastern Europe	-2.20	-2.07	-1.99	-1.49	-1.49	-1.42	-1.35	-1.15	-1.08	-0.95	-0.84	-0.75	-0.68	-0.54	-0.27	-0.25	-0.48	-0.61	-0.91	-0.98
Australasia	-1.44	-1.80	-1.36	-0.86	-1.02	-0.97	-0.75	-0.71	-0.55	-0.61	-0.49	-0.54	-0.77	-1.04	-1.04	-0.80	-0.68	-0.90	-0.70	-0.87
High-income Asia Pacific	-2.53	-2.89	-2.66	-1.99	-2.02	-2.18	-1.91	-1.96	-2.03	-2.14	-2.03	-1.83	-1.77	-1.55	-1.22	-0.85	-0.78	-0.71	-0.51	-0.36
High-income North America	-0.86	-0.64	0.02	0.59	-0.54	-0.68	-0.12	0.36	0.62	-0.19	0.31	0.35	0.37	-0.03	0.33	1.19	1.12	-0.38	-0.70	-0.92
Southern Latin America	-1.93	-2.94	-2.48	-1.45	-0.91	-0.94	-1.13	-1.55	-1.41	-1.39	-1.65	-1.69	-1.51	-1.30	-1.05	-0.56	-0.36	-0.47	-0.46	-0.27
Western Europe	-1.61	-2.02	-2.42	-1.48	-1.45	-1.17	-1.15	-1.41	-1.20	-1.33	-1.30	-1.48	-1.45	-1.42	-1.34	-0.92	-0.77	-0.97	-0.78	-0.93
Andean Latin America	-2.49	-2.87	-3.18	-2.42	-3.23	-2.77	-2.74	-2.51	-2.47	-2.38	-1.58	-1.78	-1.76	-1.62	-1.45	-1.21	-0.99	-0.96	-1.13	-1.41
Caribbean	-0.21	-0.15	-0.12	0.04	-0.13	-0.15	-0.23	-0.08	0.22	-0.38	-0.23	-0.43	-0.44	-0.43	-0.48	-0.52	-0.51	-0.52	-0.64	-0.74
Central Latin America	-1.53	-1.64	-1.67	-1.45	-1.30	-0.96	-0.90	-0.64	-0.40	-0.41	-0.69	-0.77	-0.69	-0.59	-0.39	-0.43	-0.48	-0.43	-0.71	-0.74
Tropical Latin America	-1.79	-2.04	-2.13	-1.82	-1.92	-1.68	-1.69	-1.57	-1.42	-1.29	-1.05	-0.99	-1.00	-1.03	-1.10	-1.16	-0.90	-0.76	-1.31	-1.67
North Africa and Middle East	-1.85	-2.21	-2.26	-2.07	-2.11	-1.67	-1.50	-1.41	-1.23	-1.19	-1.17	-1.14	-1.05	-0.88	-0.67	-0.30	-0.29	-0.44	-0.80	-0.62
South Asia	-1.30	-0.79	-0.92	-1.12	-1.09	-1.05	-1.17	-1.13	-1.18	-1.26	-1.22	-1.10	-1.02	-0.79	-0.57	-0.54	-0.56	-0.25	-0.44	-0.36
East Asia	-4.97	-5.16	-5.02	-4.38	-4.11	-3.74	-3.45	-3.36	-3.42	-3.14	-3.57	-3.25	-3.43	-3.34	-2.88	-2.47	-2.08	-1.62	-1.57	-1.38
Oceania	-0.33	-0.33	-0.51	-1.08	-0.77	-0.69	-0.64	-0.65	-0.47	-0.50	-0.27	-0.16	-0.04	0.02	0.03	0.05	0.02	0.00	-0.08	-0.05
Southeast Asia	-2.71	-2.42	-1.94	-1.29	-1.50	-1.42	-1.44	-1.31	-1.06	-1.12	-0.96	-0.92	-0.89	-0.61	-0.25	-0.27	-0.33	-0.43	0.15	0.24
Central Sub-Saharan Africa	-1.96	-1.90	-1.53	-1.70	-1.55	-1.38	-1.26	-1.23	-1.17	-1.28	-1.02	-0.96	-0.83	-0.64	-0.53	-0.48	-0.30	-0.31	-0.90	-0.50
Eastern Sub-Saharan Africa	-1.49	-1.22	-1.43	-1.52	-1.59	-1.60	-1.51	-1.47	-1.25	-0.95	-0.86	-0.69	-0.81	-0.64	-0.69	-0.66	-0.58	-0.48	-0.48	-0.24
Southern Sub-Saharan Africa	-0.35	-0.37	-0.54	-0.85	-1.14	-1.08	-0.96	-0.95	-0.65	-0.43	-0.23	-0.38	-0.17	-0.15	-0.20	-0.23	0.00	-0.12	-0.47	-0.40
Western Sub-Saharan Africa	-0.70	-0.57	-0.50	-0.83	-0.78	-0.69	-0.52	-0.48	-0.39	-0.52	-0.14	0.18	-0.11	-0.45	-0.47	-0.40	-0.34	-0.22	-0.20	-0.09

Fig S6. AAPC in age-specific rates of prevalence and YLDs for CKD-associated by age groups and regions from 1990 to 2021. (A) AAPC of prevalence; (B) AAPC of YLDs. YLDs, years lived with disability; CKD, chronic kidney disease; AAPC, average annual percentage change.

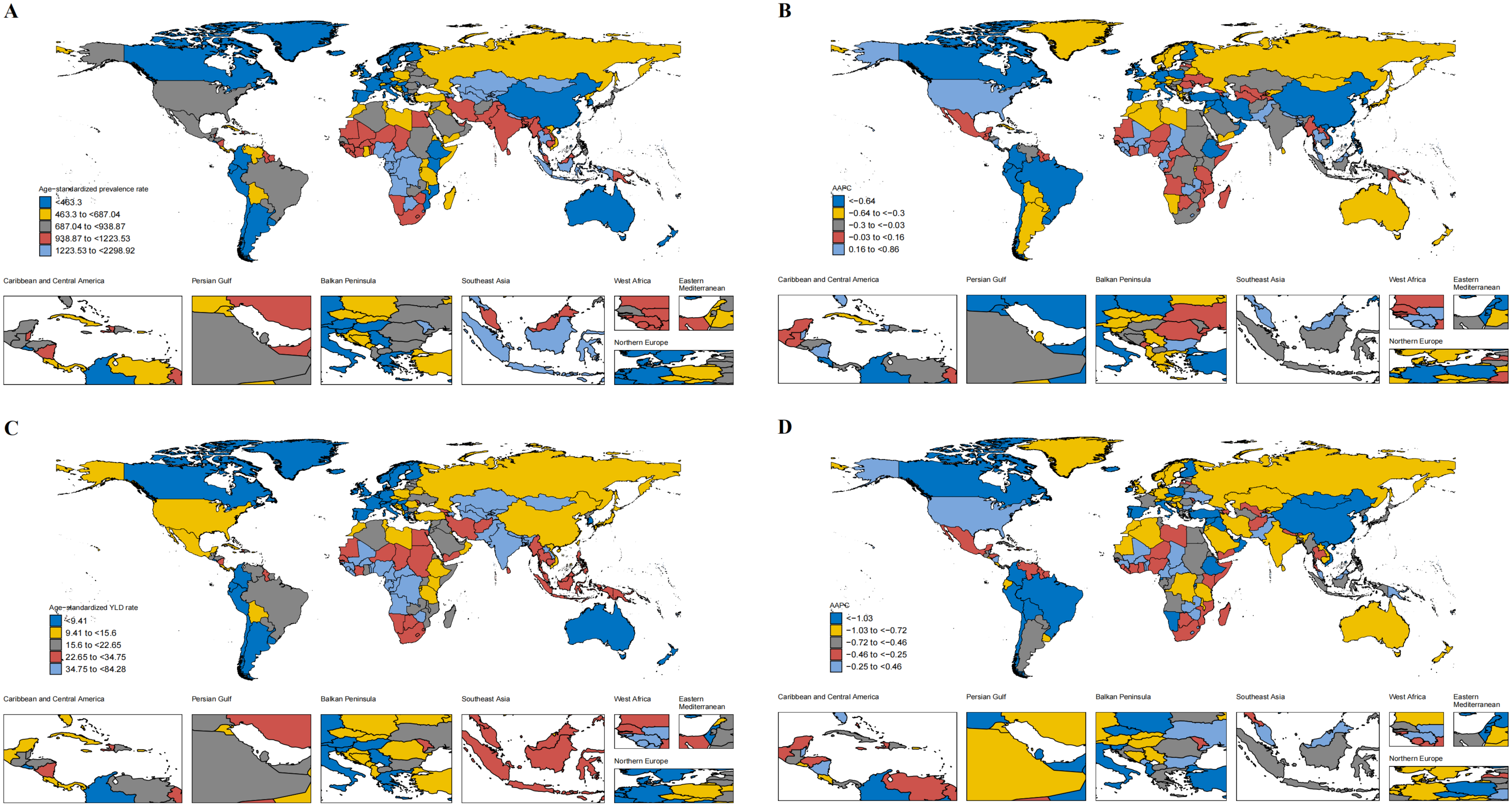

Fig S7. Global maps of CKD-associated anemia burden in 204 countries and territories.. (A) Age-standardized rate of prevalence in 2021; (B) AAPCs in age-standardized prevalence rate from 1990 to 2021; (C) Age-standardized rate of YLDs in 2021; (D) AAPCs in age-standardized YLDs rate from 1990 to 2021. CKD, chronic kidney disease; AAPC, average annual percentage change; YLDs, years lived with disability.

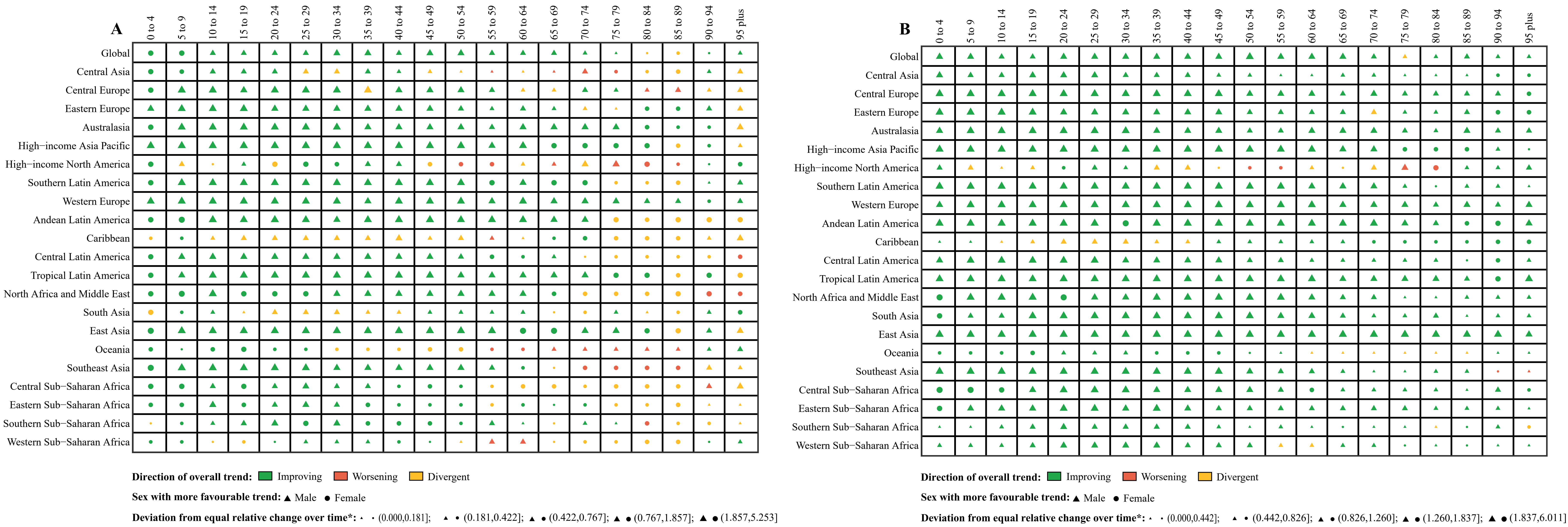

Fig S8. Absolute differences in non-fatal trend equality for males and females in age-standardized rates of prevalence and YLDs for CKD-associated anemia from 1990 to 2021. (A) Prevalence; (B) YLDs. YLDs, years lived with disability; CKD, chronic kidney disease.

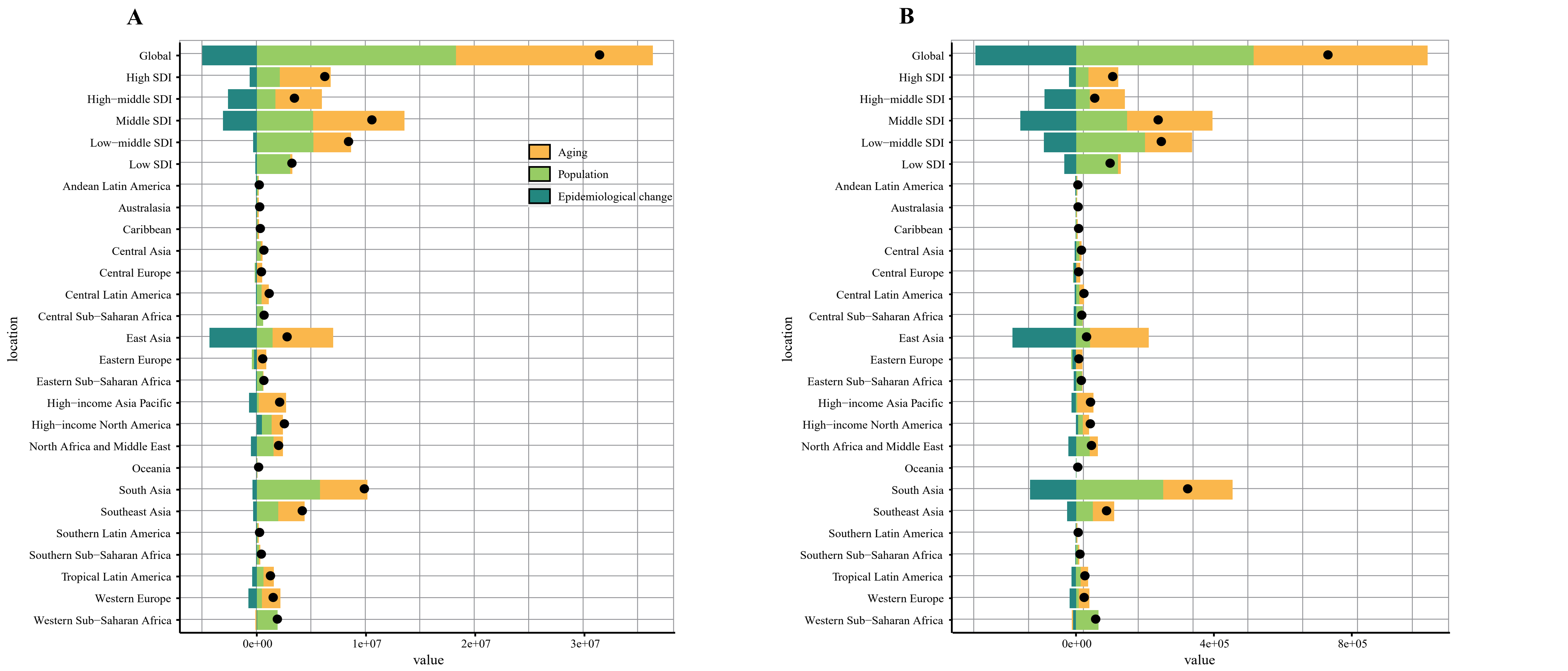

Fig S9. Changes in prevalence and YLDs of CKD-associated anemia according to aging, population growth, and epidemiological change from 1990 to 2021 at the global level by SDI quintile and various regions. (A) Prevalence; (B) YLDs. The black dots indicate the total value of change attributable to all three components. YLDs, years lived with disability; CKD, chronic kidney disease; SDI, socio-demographic Index.

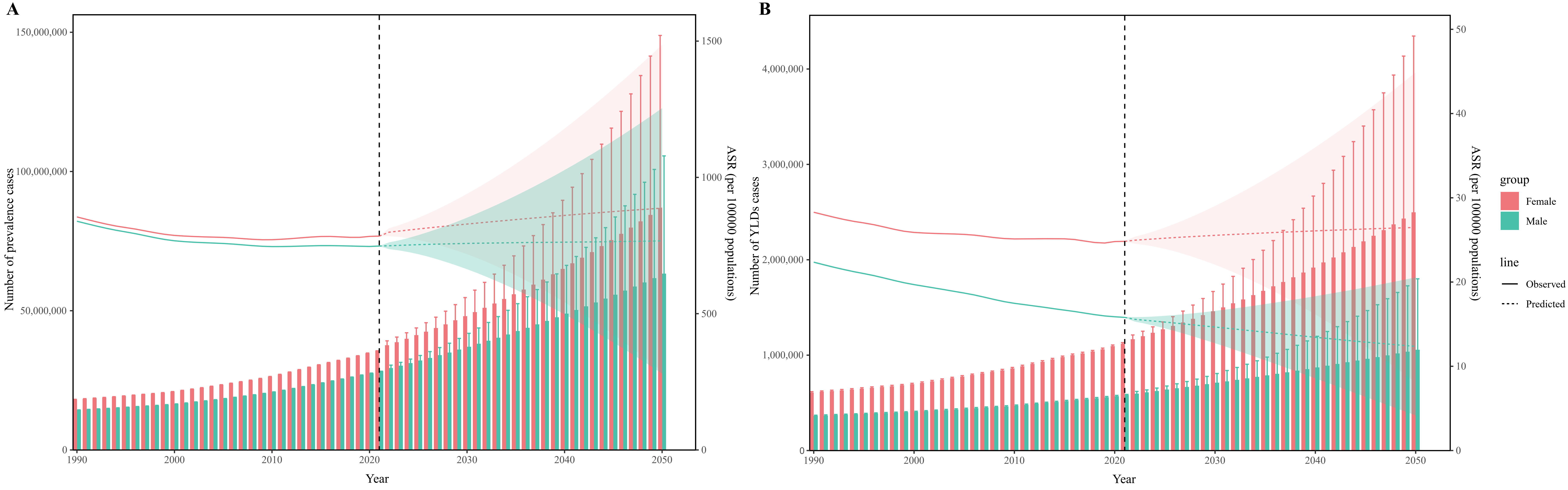

Fig S10. Prediction of CKD-associated anemia in number and age-standardized rate of prevalence and YLDs. (A) Prevalence; (B) YLDs. CKD, chronic kidney disease; YLDs, years lived with disability.
