# Supplementary material for: Global, regional, and national burden of chronic kidney disease and its associated anemia, 1990 to 2021 and predictions to 2050: an analysis of the global burden of disease study 2021
Source: BMC Nephrol. 2025 Aug 27;26:495. doi: 10.1186/s12882-025-04398-4 (PMC12382167; doi:10.1186/s12882-025-04398-4)
Supplement: Supplementary file 2 — Supplementary Material 2 [file 12882_2025_4398_MOESM2_ESM.docx]

**Table S1. Geographical hierarchy.**

| Country name | Region name | Super-region name |
| --- | --- | --- |
| Albania | Central Europe | Central Europe, Eastern Europe, and Central Asia |
| Bosnia and Herzegovina | Central Europe | Central Europe, Eastern Europe, and Central Asia |
| Bulgaria | Central Europe | Central Europe, Eastern Europe, and Central Asia |
| Croatia | Central Europe | Central Europe, Eastern Europe, and Central Asia |
| Czechia | Central Europe | Central Europe, Eastern Europe, and Central Asia |
| Hungary | Central Europe | Central Europe, Eastern Europe, and Central Asia |
| Montenegro | Central Europe | Central Europe, Eastern Europe, and Central Asia |
| North Macedonia | Central Europe | Central Europe, Eastern Europe, and Central Asia |
| Poland | Central Europe | Central Europe, Eastern Europe, and Central Asia |
| Romania | Central Europe | Central Europe, Eastern Europe, and Central Asia |
| Serbia | Central Europe | Central Europe, Eastern Europe, and Central Asia |
| Slovakia | Central Europe | Central Europe, Eastern Europe, and Central Asia |
| Slovenia | Central Europe | Central Europe, Eastern Europe, and Central Asia |
| Belarus | Eastern Europe | Central Europe, Eastern Europe, and Central Asia |
| Estonia | Eastern Europe | Central Europe, Eastern Europe, and Central Asia |
| Latvia | Eastern Europe | Central Europe, Eastern Europe, and Central Asia |
| Lithuania | Eastern Europe | Central Europe, Eastern Europe, and Central Asia |
| Republic of Moldova | Eastern Europe | Central Europe, Eastern Europe, and Central Asia |
| Russian Federation | Eastern Europe | Central Europe, Eastern Europe, and Central Asia |
| Ukraine | Eastern Europe | Central Europe, Eastern Europe, and Central Asia |
| Armenia | Central Asia | Central Europe, Eastern Europe, and Central Asia |
| Azerbaijan | Central Asia | Central Europe, Eastern Europe, and Central Asia |
| Georgia | Central Asia | Central Europe, Eastern Europe, and Central Asia |
| Kazakhstan | Central Asia | Central Europe, Eastern Europe, and Central Asia |
| Kyrgyzstan | Central Asia | Central Europe, Eastern Europe, and Central Asia |
| Mongolia | Central Asia | Central Europe, Eastern Europe, and Central Asia |
| Tajikistan | Central Asia | Central Europe, Eastern Europe, and Central Asia |
| Turkmenistan | Central Asia | Central Europe, Eastern Europe, and Central Asia |
| Uzbekistan | Central Asia | Central Europe, Eastern Europe, and Central Asia |
| Australia | Australasia | High-income |
| New Zealand | Australasia | High-income |
| Brunei Darussalam | High-income Asia Pacific | High-income |
| Japan | High-income Asia Pacific | High-income |
| Republic of Korea | High-income Asia Pacific | High-income |
| Singapore | High-income Asia Pacific | High-income |
| Canada | High-income North America | High-income |
| Greenland | High-income North America | High-income |
| United States of America | High-income North America | High-income |
| Argentina | Southern Latin America | High-income |
| Chile | Southern Latin America | High-income |
| Uruguay | Southern Latin America | High-income |
| Andorra | Western Europe | High-income |
| Austria | Western Europe | High-income |
| Belgium | Western Europe | High-income |
| Cyprus | Western Europe | High-income |
| Denmark | Western Europe | High-income |
| Finland | Western Europe | High-income |
| France | Western Europe | High-income |
| Germany | Western Europe | High-income |
| Greece | Western Europe | High-income |
| Iceland | Western Europe | High-income |
| Ireland | Western Europe | High-income |
| Israel | Western Europe | High-income |
| Italy | Western Europe | High-income |
| Luxembourg | Western Europe | High-income |
| Malta | Western Europe | High-income |
| Monaco | Western Europe | High-income |
| Netherlands | Western Europe | High-income |
| Norway | Western Europe | High-income |
| Portugal | Western Europe | High-income |
| San Marino | Western Europe | High-income |
| Spain | Western Europe | High-income |
| Sweden | Western Europe | High-income |
| Switzerland | Western Europe | High-income |
| United Kingdom | Western Europe | High-income |
| Bolivia (Plurinational State of) | Andean Latin America | Latin America and Caribbean |
| Ecuador | Andean Latin America | Latin America and Caribbean |
| Peru | Andean Latin America | Latin America and Caribbean |
| Colombia | Central Latin America | Latin America and Caribbean |
| Costa Rica | Central Latin America | Latin America and Caribbean |
| El Salvador | Central Latin America | Latin America and Caribbean |
| Guatemala | Central Latin America | Latin America and Caribbean |
| Honduras | Central Latin America | Latin America and Caribbean |
| Mexico | Central Latin America | Latin America and Caribbean |
| Nicaragua | Central Latin America | Latin America and Caribbean |
| Panama | Central Latin America | Latin America and Caribbean |
| Venezuela (Bolivarian Republic of) | Central Latin America | Latin America and Caribbean |
| Brazil | Tropical Latin America | Latin America and Caribbean |
| Paraguay | Tropical Latin America | Latin America and Caribbean |
| Antigua and Barbuda | Caribbean | Latin America and Caribbean |
| Bahamas | Caribbean | Latin America and Caribbean |
| Barbados | Caribbean | Latin America and Caribbean |
| Belize | Caribbean | Latin America and Caribbean |
| Bermuda | Caribbean | Latin America and Caribbean |
| Cuba | Caribbean | Latin America and Caribbean |
| Dominica | Caribbean | Latin America and Caribbean |
| Dominican Republic | Caribbean | Latin America and Caribbean |
| Grenada | Caribbean | Latin America and Caribbean |
| Guyana | Caribbean | Latin America and Caribbean |
| Haiti | Caribbean | Latin America and Caribbean |
| Jamaica | Caribbean | Latin America and Caribbean |
| Puerto Rico | Caribbean | Latin America and Caribbean |
| Saint Kitts and Nevis | Caribbean | Latin America and Caribbean |
| Saint Lucia | Caribbean | Latin America and Caribbean |
| Saint Vincent and the Grenadines | Caribbean | Latin America and Caribbean |
| Suriname | Caribbean | Latin America and Caribbean |
| Trinidad and Tobago | Caribbean | Latin America and Caribbean |
| United States Virgin Islands | Caribbean | Latin America and Caribbean |
| Afghanistan | North Africa and Middle East | North Africa and Middle East |
| Algeria | North Africa and Middle East | North Africa and Middle East |
| Bahrain | North Africa and Middle East | North Africa and Middle East |
| Egypt | North Africa and Middle East | North Africa and Middle East |
| Iran (Islamic Republic of) | North Africa and Middle East | North Africa and Middle East |
| Iraq | North Africa and Middle East | North Africa and Middle East |
| Jordan | North Africa and Middle East | North Africa and Middle East |
| Kuwait | North Africa and Middle East | North Africa and Middle East |
| Lebanon | North Africa and Middle East | North Africa and Middle East |
| Libya | North Africa and Middle East | North Africa and Middle East |
| Morocco | North Africa and Middle East | North Africa and Middle East |
| Oman | North Africa and Middle East | North Africa and Middle East |
| Palestine | North Africa and Middle East | North Africa and Middle East |
| Qatar | North Africa and Middle East | North Africa and Middle East |
| Saudi Arabia | North Africa and Middle East | North Africa and Middle East |
| Sudan | North Africa and Middle East | North Africa and Middle East |
| Syrian Arab Republic | North Africa and Middle East | North Africa and Middle East |
| Tunisia | North Africa and Middle East | North Africa and Middle East |
| Turkey | North Africa and Middle East | North Africa and Middle East |
| United Arab Emirates | North Africa and Middle East | North Africa and Middle East |
| Yemen | North Africa and Middle East | North Africa and Middle East |
| Bangladesh | South Asia | South Asia |
| Bhutan | South Asia | South Asia |
| India | South Asia | South Asia |
| Nepal | South Asia | South Asia |
| Pakistan | South Asia | South Asia |
| Cambodia | Southeast Asia | Southeast Asia, East Asia, and Oceania |
| Indonesia | Southeast Asia | Southeast Asia, East Asia, and Oceania |
| Lao People’s Democratic Republic | Southeast Asia | Southeast Asia, East Asia, and Oceania |
| Malaysia | Southeast Asia | Southeast Asia, East Asia, and Oceania |
| Maldives | Southeast Asia | Southeast Asia, East Asia, and Oceania |
| Mauritius | Southeast Asia | Southeast Asia, East Asia, and Oceania |
| Myanmar | Southeast Asia | Southeast Asia, East Asia, and Oceania |
| Philippines | Southeast Asia | Southeast Asia, East Asia, and Oceania |
| Seychelles | Southeast Asia | Southeast Asia, East Asia, and Oceania |
| Sri Lanka | Southeast Asia | Southeast Asia, East Asia, and Oceania |
| Thailand | Southeast Asia | Southeast Asia, East Asia, and Oceania |
| Timor-Leste | Southeast Asia | Southeast Asia, East Asia, and Oceania |
| Viet Nam | Southeast Asia | Southeast Asia, East Asia, and Oceania |
| China | East Asia | Southeast Asia, East Asia, and Oceania |
| Democratic People’s Republic of Korea | East Asia | Southeast Asia, East Asia, and Oceania |
| Taiwan (Province of China) | East Asia | Southeast Asia, East Asia, and Oceania |
| American Samoa | Oceania | Southeast Asia, East Asia, and Oceania |
| Cook Islands | Oceania | Southeast Asia, East Asia, and Oceania |
| Fiji | Oceania | Southeast Asia, East Asia, and Oceania |
| Guam | Oceania | Southeast Asia, East Asia, and Oceania |
| Kiribati | Oceania | Southeast Asia, East Asia, and Oceania |
| Marshall Islands | Oceania | Southeast Asia, East Asia, and Oceania |
| Micronesia (Federated States of) | Oceania | Southeast Asia, East Asia, and Oceania |
| Nauru | Oceania | Southeast Asia, East Asia, and Oceania |
| Niue | Oceania | Southeast Asia, East Asia, and Oceania |
| Northern Mariana Islands | Oceania | Southeast Asia, East Asia, and Oceania |
| Palau | Oceania | Southeast Asia, East Asia, and Oceania |
| Papua New Guinea | Oceania | Southeast Asia, East Asia, and Oceania |
| Samoa | Oceania | Southeast Asia, East Asia, and Oceania |
| Solomon Islands | Oceania | Southeast Asia, East Asia, and Oceania |
| Tokelau | Oceania | Southeast Asia, East Asia, and Oceania |
| Tonga | Oceania | Southeast Asia, East Asia, and Oceania |
| Tuvalu | Oceania | Southeast Asia, East Asia, and Oceania |
| Vanuatu | Oceania | Southeast Asia, East Asia, and Oceania |
| Angola | Central Sub-Saharan Africa | Sub-Saharan Africa |
| Central African Republic | Central Sub-Saharan Africa | Sub-Saharan Africa |
| Congo | Central Sub-Saharan Africa | Sub-Saharan Africa |
| Democratic Republic of the Congo | Central Sub-Saharan Africa | Sub-Saharan Africa |
| Equatorial Guinea | Central Sub-Saharan Africa | Sub-Saharan Africa |
| Gabon | Central Sub-Saharan Africa | Sub-Saharan Africa |
| Burundi | Eastern sub-Saharan Africa | Sub-Saharan Africa |
| Comoros | Eastern sub-Saharan Africa | Sub-Saharan Africa |
| Djibouti | Eastern sub-Saharan Africa | Sub-Saharan Africa |
| Eritrea | Eastern sub-Saharan Africa | Sub-Saharan Africa |
| Ethiopia | Eastern sub-Saharan Africa | Sub-Saharan Africa |
| Kenya | Eastern sub-Saharan Africa | Sub-Saharan Africa |
| Madagascar | Eastern sub-Saharan Africa | Sub-Saharan Africa |
| Malawi | Eastern sub-Saharan Africa | Sub-Saharan Africa |
| Mozambique | Eastern sub-Saharan Africa | Sub-Saharan Africa |
| Rwanda | Eastern sub-Saharan Africa | Sub-Saharan Africa |
| Somalia | Eastern sub-Saharan Africa | Sub-Saharan Africa |
| South Sudan | Eastern sub-Saharan Africa | Sub-Saharan Africa |
| Uganda | Eastern sub-Saharan Africa | Sub-Saharan Africa |
| United Republic of Tanzania | Eastern sub-Saharan Africa | Sub-Saharan Africa |
| Zambia | Eastern sub-Saharan Africa | Sub-Saharan Africa |
| Botswana | Southern Sub-Saharan Africa | Sub-Saharan Africa |
| Eswatini | Southern Sub-Saharan Africa | Sub-Saharan Africa |
| Lesotho | Southern Sub-Saharan Africa | Sub-Saharan Africa |
| Namibia | Southern Sub-Saharan Africa | Sub-Saharan Africa |
| South Africa | Southern Sub-Saharan Africa | Sub-Saharan Africa |
| Zimbabwe | Southern Sub-Saharan Africa | Sub-Saharan Africa |
| Benin | Western Sub-Saharan Africa | Sub-Saharan Africa |
| Burkina Faso | Western Sub-Saharan Africa | Sub-Saharan Africa |
| Cabo Verde | Western Sub-Saharan Africa | Sub-Saharan Africa |
| Cameroon | Western Sub-Saharan Africa | Sub-Saharan Africa |
| Chad | Western Sub-Saharan Africa | Sub-Saharan Africa |
| Côte d’Ivoire | Western Sub-Saharan Africa | Sub-Saharan Africa |
| Gambia | Western Sub-Saharan Africa | Sub-Saharan Africa |
| Ghana | Western Sub-Saharan Africa | Sub-Saharan Africa |
| Guinea | Western Sub-Saharan Africa | Sub-Saharan Africa |
| Guinea-Bissau | Western Sub-Saharan Africa | Sub-Saharan Africa |
| Liberia | Western Sub-Saharan Africa | Sub-Saharan Africa |
| Mali | Western Sub-Saharan Africa | Sub-Saharan Africa |
| Mauritania | Western Sub-Saharan Africa | Sub-Saharan Africa |
| Niger | Western Sub-Saharan Africa | Sub-Saharan Africa |
| Nigeria | Western Sub-Saharan Africa | Sub-Saharan Africa |
| Sao Tome and Principe | Western Sub-Saharan Africa | Sub-Saharan Africa |
| Senegal | Western Sub-Saharan Africa | Sub-Saharan Africa |
| Sierra Leone | Western Sub-Saharan Africa | Sub-Saharan Africa |
| Togo | Western Sub-Saharan Africa | Sub-Saharan Africa |

**Table S2.** The case number and age-standardized rates of incidence and DALYs for CKD and their AAPCs from 1990 to 2021 at the global and regional levels.

|  | Incidence | | | | |  | DALYs | | | | |
| --- | --- | --- | --- | --- | --- | --- | --- | --- | --- | --- | --- |
|  | Number of cases, 1990 | Age-standardized rate per 100,000 population, 1990 | Number of cases, 2021 | Age-standardized rate per 100,000 population, 2021 | Average annual percentage change, 1990-2021 |  | Number of cases, 1990 | Age-standardized rate per 100,000 population, 1990 | Number of cases, 2021 | Age-standardized rate per 100,000 population, 2021 | Average annual percentage change, 1990-2021 |
| Global | 7790705 (7226165-8402568) | 192.16 (178.69-207.34) | 19935038 (18702793-21170794) | 233.56 (220.02-247.24) | 0.63 (0.62 to 0.65) |  | 20739895 (18843684-22588533) | 479.85 (439.18-523.79) | 44453684 (40840762-48508462) | 529.62 (486.25-577.42) | 0.32 (0.26 to 0.38) |
| Sex |  |  |  |  |  |  |  |  |  |  |  |
| Male | 3385072 (3133504-3642480) | 180.89 (167.97-195.41) | 8806346  (  8248899  -  9359371  ) | 220.11 (207.07-233.07) | 0.64 (0.62 to 0.66) |  | 11069488 (9601627-12296840) | 546.41 (476.93-612.62) | 23759752 (21473235-26215374) | 603.40 (546.08-663.35) | 0.33 (0.23 to 0.43) |
| Female | 4405633  (4083071  -  4760087) | 201.88 (187.73-218.45) | 11128691  (10445862-11806798) | 246.03 (231.84-260.67) | 0.64 (0.63 to 0.65) |  | 9670407 (8788280-10592764) | 426.90 (389.12-467.43) | 20693932 (18836614-22728832) | 465.69 (424.02-511.12) | 0.28 (0.22 to 0.34) |
| SDI region |  |  |  |  |  |  |  |  |  |  |  |
| High SDI | 2789648 (2576788-3024433) | 252.30 (234.15-272.41) | 5666653 (5273251-6033381) | 277.75 (260.70-295.01) | 0.31 (0.29 to 0.34) |  | 2898450 (2605440-3169018) | 277.64 (250.03-303.24) | 7115740 (6464112-7759250) | 358.51 (324.74-390.20) | 0.83 (0.68 to 0.97) |
| High-middle SDI | 1574444 (1447925-1709190) | 160.44 (148.12-174.09) | 3917149 (3653537-4175391) | 205.90 (194.10-218.61) | 0.81 (0.77 to 0.84) |  | 3534406 (3184087-3954975) | 360.84 (325.48-401.53) | 5944277 (5372850-6623407) | 324.64 (293.58-360.92) | -0.35 (-0.48 to -0.23) |
| Middle SDI | 1963348 (1808277-2127370) | 171.16 (157.68-185.82) | 6251147 (5866964-6655539) | 232.96 (219.53-246.12) | 1.00 (0.99 to 1.02) |  | 7031758 (6325501-7794727) | 585.56 (531.80-652.64) | 15700568 (14206921-17147169) | 596.45 (540.33-650.48) | 0.05 (-0.02 to 0.11) |
| Low-middle SDI | 1107201 (1026640-1198009) | 153.07 (141.43-166.40) | 3130960 (2912350-3362301) | 204.97 (189.81-220.29) | 0.94 (0.88 to 1.00) |  | 4690095 (4056126-5163160) | 609.44 (545.07-686.29) | 10611260 (9599821-11771755) | 686.98 (622.50-765.20) | 0.40 (0.30 to 0.50) |
| Low SDI | 348934 (324143-375458) | 121.74 (112.82-132.30) | 950557 (888494-1011543) | 155.00 (143.40-167.34) | 0.77 (0.74 to 0.81) |  | 2564545 (2265146-2870454) | 853.94 (760.14-971.76) | 5042405 (4408653-5841516) | 791.80 (704.14-909.10) | -0.24 (-0.33 to -0.15) |
| Region |  |  |  |  |  |  |  |  |  |  |  |
| Central Asia | 64439 (59475-69705) | 114.20 (105.10-124.85) | 168442 (154020-183300) | 187.28 (173.80-201.56) | 1.62 (1.58 to 1.66) |  | 176292 (152244-202201) | 326.08 (278.46-378.79) | 427633 (377524-490696) | 493.20 (435.99-565.47) | 1.38 (0.67 to 2.10) |
| Central Europe | 193169 (174647-213352) | 132.33 (120.80-145.32) | 458516 (423618-495453) | 218.39 (203.44-234.06) | 1.63 (1.60 to 1.67) |  | 469644 (436516-504841) | 342.78 (318.66-368.53) | 539982 (472193-612672) | 266.92 (233.43-306.23) | -0.74 (-0.93 to -0.54) |
| Eastern Europe | 277461 (253751-306742) | 108.38 (100.30-117.98) | 548051 (501351-595627) | 180.10 (167.54-193.25) | 1.66 (1.63 to 1.69) |  | 521705 (463250-581791) | 207.42 (184.74-230.89) | 642467 (562943-726952) | 204.68 (180.40-232.13) | 0.00 (-0.59 to 0.59) |
| Australasia | 62421 (59023-66438) | 259.18 (245.73-274.30) | 160661 (146984-172346) | 297.30 (274.48-317.56) | 0.45 (0.43 to 0.48) |  | 46405 (42217-50551) | 211.12 (192.43-229.58) | 113693 (101514-125301) | 216.61 (193.23-239.24) | 0.01 (-0.54 to 0.57) |
| High-income Asia Pacific | 538465 (494808-584025) | 269.95 (249.25-291.71) | 1249340 (1151298-1346531) | 274.49 (255.82-293.59) | 0.07 (0.03 to 0.11) |  | 599581 (550356-643830) | 315.66 (289.04-339.18) | 1146613 (990343-1268312) | 235.57 (206.04-259.61) | -1.00 (-1.19 to -0.81) |
| High-income North America | 1050737 (962035-1145711) | 299.22 (274.55-324.91) | 2026546 (1868598-2175327) | 316.15 (294.31-338.42) | 0.19 (0.16 to 0.21) |  | 895097 (798640-986360) | 266.63 (238.28-293.17) | 3120983 (2864207-3351576) | 508.81 (467.04-545.39) | 2.11 (1.88 to 2.33) |
| Southern Latin America | 97990 (89314-107374) | 212.82 (194.87-232.31) | 244517 (224582-263090) | 281.73 (260.45-302.55) | 0.91 (0.89 to 0.94) |  | 281092 (266405-294525) | 612.71 (580.90-641.72) | 441138 (411345-467913) | 515.22 (481.75-546.80) | -0.56 (-1.10 to -0.01) |
| Western Europe | 1328467 (1221423-1459692) | 223.93 (207.15-244.09) | 2271237 (2122275-2425621) | 238.55 (223.94-254.27) | 0.22 (0.19 to 0.24) |  | 1267566 (1125408-1409086) | 234.12 (206.76-260.11) | 2361342 (2067098-2638163) | 241.72 (209.82-271.06) | 0.13 (-0.03 to 0.28) |
| Andean Latin America | 36900 (33960-40277) | 161.47 (147.69-177.34) | 177934 (165435-192798) | 298.18 (276.18-322.60) | 2.02 (1.97 to 2.07) |  | 190986 (174177-211377) | 753.97 (688.02-838.66) | 524246 (434446-625013) | 872.44 (723.54-1037.91) | 0.60 (0.31 to 0.89) |
| Caribbean | 46852 (43426-50526) | 168.79 (155.99-183.62) | 145322 (136425-154427) | 274.22 (257.52-291.86) | 1.59 (1.55 to 1.62) |  | 161590 (149056-180712) | 565.09 (523.55-636.63) | 385286 (331558-454109) | 735.85 (631.01-867.89) | 0.89 (0.57 to 1.20) |
| Central Latin America | 266579 (245613-291023) | 275.61 (252.21-302.08) | 1056443 (1000773-1110306) | 411.41 (390.17-431.32) | 1.32 (1.27 to 1.38) |  | 779859 (736752-822191) | 767.86 (724.18-811.62) | 2993750 (2691663-3371708) | 1171.14 (1054.82-1316.26) | 1.37 (0.76 to 1.97) |
| Tropical Latin America | 194244 (179335-210104) | 192.83 (176.83-209.53) | 663468 (620124-707786) | 259.33 (242.85-275.61) | 0.96 (0.92 to 1.00) |  | 594892 (560161-631009) | 562.72 (527.93-598.67) | 1311915 (1220675-1401164) | 516.97 (480.55-552.11) | -0.23 (-0.44 to -0.01) |
| North Africa and Middle East | 482950 (445183-524328) | 255.33 (235.33-277.99) | 1988923 (1863403-2127840) | 411.19 (385.40-438.45) | 1.56 (1.49 to 1.62) |  | 1493457 (1272351-1963220) | 759.90 (642.73-1059.21) | 3925988 (3427227-4413622) | 846.64 (747.17-948.02) | 0.39 (0.32 to 0.46) |
| South Asia | 1016075 (938359-1099033) | 146.23 (134.74-158.66) | 2755183 (2545113-2967871) | 177.65 (164.06-192.03) | 0.63 (0.54 to 0.72) |  | 3674401 (3157460-4115106) | 509.78 (449.63-573.28) | 8443339 (7372315-9681828) | 540.57 (473.83-620.42) | 0.20 (0.01 to 0.39) |
| East Asia | 1331855 (1207022-1453259) | 149.10 (135.79-163.09) | 3505756 (3245783-3750967) | 166.60 (156.01-176.80) | 0.34 (0.30 to 0.38) |  | 4372608 (3843678-4996867) | 461.27 (406.84-529.07) | 6486167 (5538060-7597380) | 322.36 (275.35-377.34) | -1.15 (-1.25 to -1.04) |
| Oceania | 5052 (4665-5452) | 127.57 (117.55-138.50) | 15011 (13905-16088) | 162.83 (151.06-174.45) | 0.79 (0.76 to 0.82) |  | 22680 (16686-29125) | 583.66 (445.79-741.99) | 65815 (55558-77796) | 699.04 (597.93-821.29) | 0.59 (0.46 to 0.71) |
| Southeast Asia | 466174 (432763-502980) | 156.35 (144.87-169.32) | 1568475 (1457741-1685245) | 231.28 (215.53-247.34) | 1.28 (1.25 to 1.31) |  | 2408834 (2133712-2690991) | 751.94 (672.31-850.85) | 5703263 (5028657-6329025) | 846.26 (753.05-940.53) | 0.41 (0.36 to 0.46) |
| Central Sub-Saharan Africa | 28155 (26057-30337) | 94.53 (86.79-103.02) | 88402 (82071-94885) | 130.46 (120.30-142.08) | 1.05 (1.02 to 1.08) |  | 349929 (293393-410516) | 1158.69 (983.37-1367.04) | 783700 (622595-996532) | 1124.70 (899.03-1436.09) | -0.10(-0.16 to -0.04) |
| Eastern Sub-Saharan Africa | 94221 (87688-101006) | 94.88 (87.60-102.96) | 244189 (228098-260057) | 118.08 (108.97-127.55) | 0.71 (0.68 to 0.74) |  | 1117669 (975468-1248904) | 1095.23 (965.94-1254.96) | 2047629 (1792019-2374164) | 948.36 (838.71-1090.36) | -0.46 (-0.54 to -0.39) |
| Southern Sub-Saharan Africa | 52502 (48770-56528) | 169.07 (156.50-183.90) | 142811 (132638-152029) | 233.60 (216.73-248.74) | 1.04 (0.98 to 1.11) |  | 202978 (179955-234849) | 623.85 (548.64-742.10) | 551466 (494226-622064) | 895.96 (807.34-996.98) | 1.21 (1.02 to 1.40) |
| Western Sub-Saharan Africa | 155997 (145849-167785) | 136.02 (125.90-147.10) | 455812 (426702-486021) | 179.77 (165.43-194.20) | 0.90 (0.86 to 0.95) |  | 1112629 (952298-1275995) | 928.71 (800.33-1067.12) | 2437269 (1992652-2893649) | 930.73 (788.43-1081.08) | 0.00 (-0.07 to 0.08) |

Abbreviations: DALYs, disability-adjusted life-years; CKD, chronic kidney disease; AAPC, average annual percentage change; SDI, Socio-Demographic Index.

**Table S3.** Global AAPCs in the age-standardized rates of incidence, prevalence, mortality, and DALYs for CKD.

|  | Incidence | |  | Prevalence | |  | Mortality | |  | DALYs | |
| --- | --- | --- | --- | --- | --- | --- | --- | --- | --- | --- | --- |
|  | AAPC (95% CI) | p value |  | AAPC (95% CI) | p value |  | AAPC (95% CI) | p value |  | AAPC (95% CI) | p value |
| 1990-1999 | 0.59 (0.57 to 0.62) | < 0.001 |  | -0.18 (-0.21 to -0.15) | < 0.001 |  | 0.88 (0.65 to 1.10) | < 0.001 |  | 0.38 (0.28 to 0.46) | < 0.001 |
| 2000-2009 | 0.66 (0.64 to 0.67) | < 0.001 |  | -0.07 (-0.12 to -0.03) | 0.001 |  | 0.69 (0.54 to 0.84) | <0.001 |  | 0.27 (0.14 to 0.40) | <0.001 |
| 2010-2021 | 0.66 (0.63 to 0.68) | < 0.001 |  | 0.16 (0.13 to 0.19) | < 0.001 |  | 0.51 (0.42 to 0.61) | < 0.001 |  | 0.25 (0.17 to 0.34) | < 0.001 |
| 1990-2021 | 0.63 (0.62 to 0.65) | < 0.001 |  | -0.02 (-0.04 to -0.001) | 0.037 |  | 0.73 (0.64 to 0.82) | < 0.001 |  | 0.32 (0.26 to 0.38) | < 0.001 |

Abbreviations: AAPC, average annual percentage change; DALYs, disability-adjusted life-years; CKD, chronic kidney disease; CI, confidence interval.

**Table S4.** The case number of incidence, prevalence, mortality, and DALYs for CKD from 1990 to 2021 at the global level.

| Year | Incidence | Prevalence | Mortality | DALYs |
| --- | --- | --- | --- | --- |
| 1990 | 7790705 (7226165-8402568) | 350962674 (326973785-376155723) | 552673 (513463-607915) | 20739895 (18843684-22588533) |
| 1991 | 8015667 (7445303-8639819) | 357860994 (333406188-383649726) | 565920 (521790-622917) | 21097250 (19223747-22927090) |
| 1992 | 8243753 (7660231-8880994) | 364760264 (339884604-391115834) | 581770 (536996-638461) | 21520015 (19712469-23295216) |
| 1993 | 8472928 (7875233-9123337) | 371703096 (346391675-398723619) | 597751 (554081-653380) | 21946806 (20142767-23882594) |
| 1994 | 8702550 (8087137-9360900) | 378797176 (353095536-406401390) | 614246 (571592-671724) | 22379440 (20505631-24394007) |
| 1995 | 8933924 (8307376-9602277) | 386194298 (360085336-414363697) | 634204 (588773-692165) | 22861268 (20870750-24886916) |
| 1996 | 9172944 (8533491-9857970) | 393970148 (367380083-422730130) | 652311 (606336-711042) | 23323430 (21379434-25315471) |
| 1997 | 9424628 (8770890-10128263) | 401996836 (374899613-431366427) | 671586 (625986-730188) | 23834063 (21928979-25911902) |
| 1998 | 9685681 (9016385-10413592) | 410246159 (382623312-440227594) | 700271 (653320-757327) | 24591441 (22612127-26639518) |
| 1999 | 9956485 (9267397-10711020) | 418729046 (390571233-449340338) | 729118 (677819-787362) | 25376352 (23525338-27488049) |
| 2000 | 10237730 (9528250-11020106) | 427471050 (398757009-458725037) | 764663 (711740-825005) | 26203703 (24226795-28357236) |
| 2001 | 10540857 (9830059-11338257) | 436262910 (407063801-468197079) | 793950 (741235-857876) | 26923424 (24886878-29233771) |
| 2002 | 10867176 (10141234-11691243) | 444925906 (415500483-477303516) | 821502 (766084-878707) | 27646836 (25595515-29816997) |
| 2003 | 11207543 (10463440-12059452) | 453616859 (424055976-486467392) | 850621 (793001-905377) | 28443131 (26325283-30780550) |
| 2004 | 11558355 (10797258-12413260) | 462703377 (432654355-496207562) | 874341 (813523-934511) | 29116824 (26896735-31496074) |
| 2005 | 11909103 (11131132-12767806) | 472474563 (441836783-506636897) | 899261 (839305-956282) | 29780846 (27626013-32129282) |
| 2006 | 12281083 (11484855-13168741) | 483028705 (451576680-517748614) | 919425 (855362-976965) | 30276215 (27970383-32609097) |
| 2007 | 12688415 (11851600-13611503) | 494068724 (461989783-529342110) | 948517 (881764-1010366) | 30944375 (28597185-33298835) |
| 2008 | 13115766 (12243101-14049366) | 505446135 (472709915-541376028) | 984300 (917223-1041763) | 31770499 (29423770-34204613) |
| 2009 | 13557767 (12659843-14501162) | 517216073 (483812801-554065198) | 1019429 (947007-1079383) | 32591874 (30161229-35122242) |
| 2010 | 13995323 (13085139-14955857) | 529126337 (495066780-566827634) | 1058755 (981418-1123750) | 33435899 (31024231-35825151) |
| 2011 | 14453136 (13522710-15421475) | 542087111 (507251444-580653208) | 1099616 (1018382-1157525) | 34343150 (31911696-36953825) |
| 2012 | 14951800 (13993562-15936219) | 556637474 (520969617-596365067) | 1136308 (1048369-1202187) | 35215701 (32653752-37994642) |
| 2013 | 15472942 (14486340-16481573) | 571789571 (535266045-612718072) | 1184500 (1093146-1251279) | 36341534 (33708905-39202576) |
| 2014 | 16009965 (15002951-17038636) | 586724717 (549293988-628626206) | 1229564 (1131367-1306715) | 37366461 (34793109-40273731) |
| 2015 | 16537859 (15505583-17573066) | 600540006 (562111765-643365916) | 1280017 (1182763-1357536) | 38561976 (35722998-41734531) |
| 2016 | 17088768 (16061712-18156714) | 613370017 (573897470-657221510) | 1329850 (1217347-1401602) | 39711574 (36844756-42686909) |
| 2017 | 17677953 (16653461-18765150) | 626027136 (585323194-670781869) | 1369780 (1257172-1442498) | 40515813 (37587857-43759006) |
| 2018 | 18278682 (17206166-19393351) | 638811738 (596874611-684364880) | 1410445 (1297283-1496331) | 41321304 (38221969-44502156) |
| 2019 | 18879366 (17748527-20014103) | 652293552 (609380392-698783365) | 1462675 (1341360-1552920) | 42453964 (39123282-45975979) |
| 2020 | 19469748 (18262548-20662428) | 664658454 (620819896-713003613) | 1499449 (1366228-1606268) | 43502604 (40074262-47332828) |
| 2021 | 19935038 (18702793-21170794) | 673722703 (629095119-722364096) | 1527639 (1389377-1638914) | 44453684 (40840762-48508462) |

Abbreviations: DALYs, disability-adjusted life-years; CKD, chronic kidney disease.

**Table S5.** The age-standardized rates of incidence, prevalence, mortality, and DALYs for CKD from 1990 to 2021 at the global level.

| Year | Age-standardised rate per 100 000 population | | | |
| --- | --- | --- | --- | --- |
|  | Incidence | Prevalence | Mortality | DALYs |
| 1990 | 192.16 (178.69-207.34) | 8072.75 (7560.37-8634.07) | 14.85 (13.64-16.38) | 479.85 (439.18-523.79) |
| 1991 | 193.45 (180.01-208.59) | 8045.94 (7539.61-8601.22) | 14.84 (13.75-16.38) | 478.15 (435.90-520.51) |
| 1992 | 194.74 (181.24-209.90) | 8019.92 (7517.41-8569.79) | 14.89 (13.63-16.42) | 478.10 (438.95-519.86) |
| 1993 | 196.02 (182.42-211.26) | 7997.01 (7497.95-8542.08) | 14.96 (13.79-16.48) | 478.34 (439.50-520.04) |
| 1994 | 197.20 (183.57-212.50) | 7978.77 (7482.54-8518.50) | 15.03 (13.93-16.50) | 478.82 (438.12-522.80) |
| 1995 | 198.35 (184.73-213.70) | 7967.47 (7471.99-8506.78) | 15.21 (14.03-16.67) | 480.70 (439.46-523.47) |
| 1996 | 199.41 (185.77-214.78) | 7960.12 (7464.37-8498.34) | 15.32 (14.13-16.75) | 481.65 (443.52-523.13) |
| 1997 | 200.49 (186.81-215.91) | 7952.89 (7456.74-8490.21) | 15.42 (14.26-16.82) | 483.08 (444.77-523.66) |
| 1998 | 201.59 (187.87-217.08) | 7946.60 (7449.86-8482.70) | 15.72 (14.56-17.09) | 489.21 (451.74-528.04) |
| 1999 | 202.65 (188.88-218.34) | 7940.84 (7443.49-8475.14) | 16.00 (14.84-17.33) | 495.05 (457.93-534.47) |
| 2000 | 203.77 (189.86-219.70) | 7936.72 (7437.94-8472.32) | 16.45 (15.19-17.80) | 502.00 (463.20-542.00) |
| 2001 | 205.00 (191.14-220.86) | 7929.33 (7431.20-8466.20) | 16.70 (15.46-18.08) | 505.75 (467.72-547.37) |
| 2002 | 206.41 (192.60-222.24) | 7916.43 (7420.01-8449.77) | 16.88 (15.64-18.10) | 508.78 (471.69-548.06) |
| 2003 | 207.93 (194.10-223.72) | 7901.80 (7407.47-8430.87) | 17.06 (15.76-18.19) | 512.62 (475.10-553.27) |
| 2004 | 209.34 (195.44-225.05) | 7888.80 (7396.24-8414.06) | 17.10 (15.73-18.33) | 513.44 (475.33-555.65) |
| 2005 | 210.59 (196.60-226.23) | 7882.39 (7390.36-8404.86) | 17.13 (15.89-18.25) | 513.75 (475.52-554.58) |
| 2006 | 211.77 (197.90-227.11) | 7880.48 (7387.55-8401.86) | 17.05 (15.76-18.16) | 510.61 (473.06-549.03) |
| 2007 | 213.15 (199.33-228.30) | 7879.62 (7385.55-8402.41) | 17.11 (15.78-18.24) | 509.91 (471.79-548.25) |
| 2008 | 214.69 (200.71-229.72) | 7880.57 (7386.41-8407.21) | 17.27 (15.93-18.33) | 511.49 (474.67-550.89) |
| 2009 | 216.19 (202.04-231.05) | 7883.15 (7389.32-8413.96) | 17.38 (16.01-18.44) | 512.40 (475.96-552.50) |
| 2010 | 217.57 (203.45-232.07) | 7888.44 (7392.91-8423.38) | 17.55 (16.17-18.66) | 513.73 (476.60-550.50) |
| 2011 | 218.93 (204.85-233.06) | 7906.42 (7407.83-8444.41) | 17.71 (16.33-18.67) | 515.53 (480.15-554.96) |
| 2012 | 220.52 (206.44-234.64) | 7941.65 (7438.22-8485.36) | 17.77 (16.29-18.86) | 516.03 (478.32-556.89) |
| 2013 | 222.24 (208.32-236.37) | 7982.38 (7474.49-8532.70) | 17.99 (16.46-19.05) | 519.97 (481.57-560.86) |
| 2014 | 223.91 (210.18-238.01) | 8016.71 (7504.96-8572.64) | 18.15 (16.56-19.28) | 522.16 (486.58-563.22) |
| 2015 | 225.40 (211.69-239.26) | 8035.63 (7521.37-8594.52) | 18.36 (16.85-19.49) | 526.26 (488.32-569.60) |
| 2016 | 226.83 (213.20-240.62) | 8037.60 (7521.45-8595.55) | 18.51 (16.84-19.57) | 529.21 (491.28-568.74) |
| 2017 | 228.48 (215.17-242.16) | 8034.35 (7516.19-8591.74) | 18.51 (16.94-19.52) | 527.34 (489.52-569.86) |
| 2018 | 230.13 (216.72-243.67) | 8031.73 (7511.78-8589.94) | 18.50 (16.88-19.68) | 525.33 (485.69-565.57) |
| 2019 | 231.49 (218.05-244.88) | 8034.31 (7513.63-8595.50) | 18.63 (17.01-19.75) | 527.18 (486.34-571.61) |
| 2020 | 233.13 (219.52-247.32) | 8033.46 (7508.79-8612.27) | 18.59 (16.86-19.92) | 528.49 (485.92-574.90) |
| 2021 | 233.56 (220.02-247.24) | 8006.00 (7482.12-8575.62) | 18.50 (16.72-19.85) | 529.62 (486.25-577.42) |

Abbreviations: DALYs, disability-adjusted life-years; CKD, chronic kidney disease.

**Table S6.** The number and age-standardized rate of prevalence for CKD and its AAPC from 1990 to 2021

at the global and regional levels.

|  | Number of cases, 1990 | Age-standardized rate per 100 000 population, 1990 | Number of cases, 2021 | Age-standardized rate per 100 000 population, 2021 | Average annual percentage change, 1990-2021 | p value |
| --- | --- | --- | --- | --- | --- | --- |
| Global | 350962674 (326973785-376155723) | 8072.75 (7560.37-8634.07) | 673722703 (629095119-722364096) | 8006.00 (7482.12-8575.62) | -0.02 (-0.04 to -0.001) | 0.037 |
| Sex |  |  |  |  |  |  |
| Male | 162777648 (151189911-174916268) | 7812.80 (7301.88-8356.28) | 314945280 (293385891-338118606) | 7808.96 (7288.71-8366.60) | 0.01 (-0.03 to 0.05) | 0.651 |
| Female | 188185026 (175770189-201580675) | 8293.08 (7778.75-8870.27) | 358777424 (335684717-383643812) | 8182.65 (7653.14-8764.76) | -0.04 (-0.05 to -0.03) | <0.001 |
| SDI region |  |  |  |  |  |  |
| High SDI | 72796005 (68039542-77665594) | 6883.99 (6452.03-7322.72) | 120879407 (113333443-127967827) | 6733.55 (6322.09-7159.65) | -0.08 (-0.10 to -0.05) | <0.001 |
| High-middle SDI | 78208075 (72803156-84086890) | 7641.21 (7149.96-8188.95) | 127111061 (118337580-136309239) | 7267.74 (6782.08-7816.70) | -0.16 (-0.19 to -0.14) | <0.001 |
| Middle SDI | 105819342 (98201190-113755650) | 8450.59 (7891.90-9069.19) | 220823054 (205500732-237345634) | 8280.06 (7728.02-8885.29) | -0.07 (-0.10 to -0.03) | <0.001 |
| Low-middle SDI | 70588690 (65338440-76043937) | 9292.53 (8660.54-9952.48) | 149861034 (139108422-161539154) | 9171.03 (8543.99-9848.15) | -0.04 (-0.11 to 0.04) | 0.318 |
| Low SDI | 23236078 (21506138-25034232) | 8090.37 (7566.41-8624.95) | 54528960 (50362040-59039888) | 7984.37 (7449.39-8549.73) | -0.03 (-0.09 to 0.02) | 0.242 |
| Region |  |  |  |  |  |  |
| Central Asia | 5534240 (5182702-5905060) | 10649.08 (10005.67-11309.29) | 9356434 (8704146-9969948) | 10698.24 (10022.94-11348.10) | 0.01 (0.01 to 0.02) | <0.001 |
| Central Europe | 8932587 (8363724-9525233) | 6368.64 (5978.05-6771.96) | 11481266 (10766159-12145281) | 6205.18 (5841.05-6596.24) | -0.08 (-0.10 to -0.07) | <0.001 |
| Eastern Europe | 24173788 (22444048-26062971) | 9327.55 (8680.03-10050.13) | 27312843 (25403078-29335270) | 9266.31 (8619.37-9989.49) | -0.02 (-0.03 to -0.01) | <0.001 |
| Australasia | 1399739 (1315064-1481429) | 6084.38 (5719.40-6456.26) | 2810777 (2637789-2995329) | 5910.04 (5548.15-6301.57) | -0.11 (-0.15 to -0.06) | <0.001 |
| High-income Asia Pacific | 16878162 (15709633-18083496) | 8566.14 (7979.52-9169.53) | 29276226 (27336162-31020670) | 7920.71 (7393.74-8457.44) | -0.25 (-0.27 to -0.22) | <0.001 |
| High-income North America | 25125238 (23432071-26893288) | 7401.06 (6920.42-7900.28) | 42488578 (39404535-45165702) | 7434.68 (6959.33-7911.44) | 0.00 (-0.05 to 0.06) | 0.869 |
| Southern Latin America | 2695843 (2510441-2883801) | 5784.40 (5397.31-6182.95) | 4860631 (4530777-5214539) | 5970.43 (5543.08-6414.98) | 0.10 (0.09 to 0.12) | <0.001 |
| Western Europe | 29093594 (27230515-30911868) | 5469.90 (5132.58-5799.43) | 41591466 (39070950-43910754) | 5226.19 (4924.43-5544.2) | -0.15 (-0.16 to -0.13) | <0.001 |
| Andean Latin America | 1465459 (1365798-1575973) | 5766.55 (5377.83-6181.01) | 3743589 (3478926-4028408) | 5946.06 (5524.81-6371.54) | 0.10 (0.09 to 0.11) | <0.001 |
| Caribbean | 1867236 (1741663-2000227) | 6480.16 (6037.62-6925.81) | 3468705 (3235452-3720720) | 6632.98 (6181.25-7112.38) | 0.08 (0.07 to 0.08) | <0.001 |
| Central Latin America | 8628113 (8065183-9238847) | 8477.01 (7931.03-9062.77) | 22101801 (20701763-23422153) | 8642.90 (8089.33-9163.75) | 0.07 (0.06 to 0.07) | <0.001 |
| Tropical Latin America | 8408163 (7820461-9015639) | 7763.01 (7240.67-8281.35) | 19295703 (18022781-20680042) | 7576.23 (7080.97-8107.55) | -0.08 (-0.10 to -0.06) | <0.001 |
| North Africa and Middle East | 19739135 (18244354-21279746) | 9119.72 (8490.94-9769.64) | 49668756 (45835433-53616786) | 9180.02 (8523.53-9830.12) | 0.02 (0.01 to 0.03) | <0.001 |
| South Asia | 72584182 (67087414-78502263) | 9936.56 (9252.61-10645.69) | 158803354 (147190732-171556959) | 9565.26 (8903.67-10265.71) | 0.09 (0.08 to 0.10) | <0.001 |
| East Asia | 69797869 (64858255-75190145) | 7083.56 (6610.07-7593.48) | 122849232 (113607491-132118615) | 6258.13 (5823.13-6729.82) | -0.39 (-0.46 to -0.31) | <0.001 |
| Oceania | 306814 (282467-332777) | 7742.43 (7231.44-8304.17) | 776661 (715870-846878) | 7950.68 (7408.82-8567.73) | 0.09 (0.08 to 0.10) | <0.001 |
| Southeast Asia | 33768821 (31068003-36689892) | 10225.21 (9474.04-11022.10) | 73561437 (67779796-79733436) | 10474.65 (9718.92-11301.72) | 0.09 (0.08 to 0.10) | <0.001 |
| Central Sub-Saharan Africa | 2633228 (2443845-2831336) | 9221.98 (8598.75-9806.74) | 6811951 (6363747-7313187) | 9165.20 (8640.85-9709.73) | -0.02 (-0.03 to -0.01) | 0.006 |
| Eastern Sub-Saharan Africa | 5981606 (5488507-6503182) | 5677.22 (5266.15-6113.13) | 15017846 (13741759-16422384) | 5821.27 (5404.49-6293.60) | 0.08 (0.08 to 0.08) | <0.001 |
| Southern Sub-Saharan Africa | 2945199 (2740170-3170275) | 8946.44 (8370.42-9563.71) | 5972951 (5547456-6415524) | 9037.91 (8440.60-9647.06) | 0.04 (0.03 to 0.06) | <0.001 |
| Western Sub-Saharan Africa | 9003658 (8381320-9663357) | 8310.65 (7767.39-8851.61) | 22472497 (20882526-24191820) | 8324.27 (7782.26-8871.02) | 0.01 (0.00 to 0.02) | 0.119 |

Abbreviations: CKD, chronic kidney disease; AAPC, average annual percentage change; SDI, Socio-Demographic Index.

**Table S7**. The number and age-standardized rate of mortality for CKD and its AAPC from 1990 to 2021 at the

global and regional levels.

|  | Number of cases, 1990 | Age-standardized rate per 100 000 population, 1990 | Number of cases, 2021 | Age-standardized rate per 100 000 population, 2021 | Average annual percentage change, 1990-2021 | p value |
| --- | --- | --- | --- | --- | --- | --- |
| Global | 552673 (513463-607915) | 14.85 (13.64-16.38) | 1527639 (1389377-1638914) | 18.50 (16.72-19.85) | 0.73 (0.64 to 0.82) | <0.001 |
| Sex |  |  |  |  |  |  |
| Male | 291666 (259303-334444) | 18.13 (16.26-21.03) | 794519 (719354-856326) | 21.91 (19.66-23.60) | 0.63 (0.51 to 0.75) | <0.001 |
| Female | 261007 (237127-287997) | 12.64 (11.44-13.99) | 733120 (654830-795633) | 15.9 (14.22-17.27) | 0.75 (0.65 to 0.85) | <0.001 |
| SDI region |  |  |  |  |  |  |
| High SDI | 100025 (92113-104189) | 9.22 (8.45-9.62) | 340083 (289016-369665) | 14.11 (12.30-15.21) | 1.44 (1.16 to 1.71) | <0.001 |
| High-middle SDI | 99196 (91758-110668) | 11.36 (10.43-12.65) | 226797 (201672-252703) | 12.02 (10.68-13.38) | 0.21 (0.04 to 0.38) | 0.014 |
| Middle SDI | 177302 (162783-198065) | 19.07 (17.39-21.35) | 513051 (458865-556752) | 20.89 (18.45-22.67) | 0.31 (0.20 to 0.42) | <0.001 |
| Low-middle SDI | 110899 (98721-127673) | 18.59 (16.43-22.23) | 309509 (280315-349455) | 23.08 (20.97-26.31) | 0.73 (0.54 to 0.92) | <0.001 |
| Low SDI | 64656 (57360-73648) | 29.72 (26.31-34.62) | 136797 (118867-157579) | 29.43 (26.13-33.79) | -0.01 (-0.15 to 0.13) | 0.92 |
| Region |  |  |  |  |  |  |
| Central Asia | 2536 (2250-2933) | 5.01 (4.38-5.94) | 9397 (8278-10515) | 12.11 (10.72-13.53) | 2.88 (1.81 to 3.96) | <0.001 |
| Central Europe | 14335 (13732-14892) | 10.47 (10.00-10.92) | 21635 (19445-23977) | 9.39 (8.41-10.47) | -0.32 (-0.77 to 0.14) | 0.17 |
| Eastern Europe | 9389 (9183-9574) | 3.58 (3.50-3.65) | 17821 (16126-19853) | 5.22 (4.73-5.82) | 1.30 (0.53 to 2.07) | 0.001 |
| Australasia | 1851 (1687-1959) | 8.47 (7.66-8.99) | 5968 (5025-6525) | 9.63 (8.23-10.47) | 0.34 (-0.47 to 1.16) | 0.407 |
| High-income Asia Pacific | 22233 (20303-23333) | 12.61 (11.29-13.32) | 63459 (50432-70953) | 9.74 (8.09-10.68) | -0.87 (-1.23 to -0.50) | <0.001 |
| High-income North America | 29949 (27171-31433) | 8.32 (7.57-8.73) | 143679 (124827-154757) | 20.55 (18.10-22.00) | 2.96 (2.76 to 3.16) | <0.001 |
| Southern Latin America | 11256 (10698-11760) | 26.00 (24.55-27.17) | 21583 (19510-22986) | 23.85 (21.63-25.37) | -0.28 (-0.85 to 0.30) | 0.34 |
| Western Europe | 48662 (44430-50877) | 8.26 (7.52-8.66) | 133481 (109637-148166) | 10.66 (8.90-11.81) | 0.88 (0.57 to 1.19) | <0.001 |
| Andean Latin America | 5795 (5288-6456) | 28.53 (25.87-31.87) | 21619 (17903-25800) | 37.66 (31.27-44.93) | 0.98 (0.52 to 1.44) | <0.001 |
| Caribbean | 4754 (4399-5437) | 18.73 (17.30-21.32) | 13888 (12086-16193) | 25.78 (22.39-30.07) | 1.04 (0.79 to 1.29) | <0.001 |
| Central Latin America | 22182 (21513-22820) | 27.94 (26.82-28.83) | 104444 (94171-116461) | 42.35 (38.32-47.03) | 1.36 (0.68 to 2.03) | <0.001 |
| Tropical Latin America | 15524 (14832-16076) | 17.94 (16.85-18.75) | 46925 (42508-49438) | 18.84 (17.00-19.87) | 0.26 (-0.36 to 0.88) | 0.409 |
| North Africa and Middle East | 45472 (37703-66491) | 31.22 (25.48-47.04) | 144687 (126153-162699) | 37.71 (32.73-42.39) | 0.68 (0.58 to 0.77) | <0.001 |
| South Asia | 79714 (68086-89404) | 13.98 (12.14-15.90) | 226043 (192546-264155) | 16.45 (14.03-19.25) | 0.59 (0.20 to 0.97) | 0.003 |
| East Asia | 107701 (94477-126024) | 14.37 (12.68-16.98) | 217342 (178047-259057) | 11.15 (9.20-13.21) | -0.86 (-1.05 to -0.66) | <0.001 |
| Oceania | 489 (342-661) | 17.22 (12.83-23.08) | 1546 (1265-1892) | 21.56 (17.98-26.37) | 0.72 (0.59 to 0.84) | <0.001 |
| Southeast Asia | 57881 (52199-66490) | 22.86 (20.59-26.70) | 170033 (148864-190537) | 28.47 (24.79-31.87) | 0.74 (0.68 to 0.80) | <0.001 |
| Central Sub-Saharan Africa | 8667 (7200-10419) | 42.68 (35.55-51.04) | 21011 (16114-27467) | 43.69 (33.26-56.29) | 0.08 (0.02 to 0.13) | 0.005 |
| Eastern Sub-Saharan Africa | 30615 (26919-35172) | 42.40 (37.14-50.19) | 60918 (53817-69935) | 40.09 (35.57-46.03) | -0.17 (-0.25 to -0.09) | <0.001 |
| Southern Sub-Saharan Africa | 5376 (4674-6619) | 20.86 (17.97-26.16) | 17365 (15612-19485) | 34.43 (31.05-38.29) | 1.65 (1.28 to 2.02) | <0.001 |
| Western Sub-Saharan Africa | 28294 (24259-32687) | 33.65 (28.97-39.19) | 64796 (53512-76124) | 36.40 (31.14-42.75) | 0.26 (0.17 to 0.35) | <0.001 |

Abbreviations: CKD, chronic kidney disease; AAPC, average annual percentage change; SDI, Socio-Demographic Index.

**Table S8.** The number and age-standardized rate of prevalence for CKD-associated anemia and its AAPC

from 1990 to 2021 at the global and regional levels.

|  | Number of cases, 1990 | Age-standardized rate per 100 000 population, 1990 | Number of cases, 2021 | Age-standardized rate per 100 000 population, 2021 | Average annual percentage change, 1990-2021 | p value |
| --- | --- | --- | --- | --- | --- | --- |
| Global | 32486224 (30356876-35047084) | 841.18 (783.12-906.48) | 63751624 (59045051-68372650) | 762.12 (707.32-817.37) | -0.32 (-0.35 to -0.29) | <0.001 |
| Sex |  |  |  |  |  |  |
| Male | 14346016 (13323901-15630185) | 839.10 (780.83-910.92) | 28208709 (26097779-30409324) | 748.10 (691.69-807.21) | -0.37  (-0.39 to -0.35) | <0.001 |
| Female | 18140208 (16913628-19524601) | 854.93 (798.01-921.56) | 35542915 (32942946-38267108) | 784.53 (728.64-842.42) | -0.27  (-0.30 to -0.25) | <0.001 |
| SDI region |  |  |  |  |  |  |
| High SDI | 6831434 (6294908-7497741) | 631.2 (583.76-687.86) | 12937734 (11594440-14338944) | 575.32 (517.79-636.52) | -0.30 (-0.34 to -0.25) | <0.001 |
| High-middle SDI | 6546839 (6049891-7127759) | 714.28 (658.63-777.30) | 9876782 (9030632-10648516) | 526.27 (483.55-565.57) | -0.98 (-1.00 to -0.96) | <0.001 |
| Middle SDI | 9868168 (9081277-10734606) | 958.14 (883.95-1046.64) | 20291028 (18699076-21983141) | 1050.06 (971.42-1140.28) | -0.59 (-0.65 to -0.53) | <0.001 |
| Low-middle SDI | 6735513 (6240338-7312831) | 1077.42 (996.26-1174.43) | 15019551 (13936960-16277999) | 995.84 (916.54-1080.20) | -0.08 (-0.11 to -0.05) | <0.001 |
| Low SDI | 2472885 (2274376-2695336) | 1016.33 (931.79-1110.53) | 5573562 (5150095-6019435) | 798.65 (737.73-861.88) | -0.07 (-0.09 to -0.05) | <0.001 |
| Region |  |  |  |  |  |  |
| Central Asia | 753089 (682295-848101) | 1580.52 (1428.16-1779.32) | 1280182 (1151960-1434237) | 1615.07 (1455.29-1800.63) | 0.07 (0.06 to 0.08) | <0.001 |
| Central Europe | 903346 (818831-994151) | 677.19 (617.68-739.56) | 1214132 (1099707-1333265) | 603.57 (551.82-660.03) | -0.37 (-0.40 to -0.34) | <0.001 |
| Eastern Europe | 2029244 (1743826-2358566) | 802.18 (696.67-918.63) | 2453293 (2124762-2834246) | 719.56 (626.84-820.29) | -0.35 (-0.36 to -0.33) | <0.001 |
| Australasia | 109319 (92469-131869) | 492.68 (418.17-591.29) | 249955 (206209-306611) | 414.66 (346.56-504.35) | -0.57 (-0.68 to -0.47) | <0.001 |
| High-income Asia Pacific | 1695803 (1479538-1944644) | 896.63 (786.34-1031.46) | 3673315 (3070457-4328397) | 660.18 (553.76-776.23) | -0.98 (-1.02 to -0.95) | <0.001 |
| High-income North America | 2135480 (1927296-2386674) | 595.36 (540.26-661.86) | 4539513 (3869673-5366310) | 678.80 (578.75-796.49) | 0.43 (0.38 to 0.47) | <0.001 |
| Southern Latin America | 219055 (193329-253899) | 490.00 (431.76-566.09) | 351189 (308706-405781) | 402.33 (353.04-463.19) | -0.65 (-0.69 to -0.61) | <0.001 |
| Western Europe | 2775555 (2527159-3050494) | 493.40 (450.23-539.26) | 4156665 (3725128-4612421) | 383.47 (346.65-423.88) | -0.81 (-0.82 to -0.79) | <0.001 |
| Andean Latin America | 132454 (119249-147457) | 569.78 (507.17-640.82) | 242931 (216487-272859) | 409.48 (362.36-461.34) | -1.06 (-1.09 to -1.03) | <0.001 |
| Caribbean | 200558 (179692-225735) | 770.39 (682.92-867.64) | 401404 (356259-443422) | 748.32 (665.25-826.02) | -0.10 (-0.12 to -0.07) | <0.001 |
| Central Latin America | 588517 (546592-641158) | 700.08 (643.27-768.31) | 1600975 (1476233-1736995) | 656.59 (604.70-711.45) | -0.20 (-0.22 to -0.19) | <0.001 |
| Tropical Latin America | 970336 (822163-1131309) | 1086.40 (921.29-1266.76) | 2100626 (1773962-2497967) | 836.38 (708.66-992.28) | -0.83 (-0.90 to -0.76) | <0.001 |
| North Africa and Middle East | 1667561 (1545140-1807267) | 905.38 (837.21-986.84) | 3549996 (3241471-3913046) | 787.49 (714.66-863.13) | -0.44 (-0.45 to -0.43) | <0.001 |
| South Asia | 6924406 (6394247-7543929) | 1165.20 (1073.07-1273.73) | 16656701 (15357588-18058350) | 1125.96 (1038.89-1221.98) | -0.11 (-0.15 to -0.07) | <0.001 |
| East Asia | 5879239 (5398796-6421814) | 716.66 (656.76-782.00) | 8538579 (7771758-9371900) | 431.26 (394.33-471.04) | -1.62 (-1.69 to -1.55) | <0.001 |
| Oceania | 32681 (29085-36696) | 1062.49 (942.62-1195.30) | 85278 (75472-96636) | 1114.25 (981.63-1253.29) | 0.15 (0.13 to 0.17) | <0.001 |
| Southeast Asia | 3073915 (2820958-3341850) | 1196.48 (1087.40-1318.58) | 7120216 (6502555-7795828) | 1172.36 (1067.22-1289.52) | -0.06 (-0.09 to -0.03) | <0.001 |
| Central Sub-Saharan Africa | 382313 (328260-443207) | 1573.69 (1363.26-1809.69) | 925957 (809409-1056026) | 1531.84 (1329.91-1743.46) | -0.09 (-0.14 to -0.04) | <0.001 |
| Eastern Sub-Saharan Africa | 451657 (415493-490009) | 542.32 (496.49-594.58) | 981576 (904339-1061590) | 514.99 (470.35-563.06) | -0.17 (-0.19 to -0.15) | <0.001 |
| Southern Sub-Saharan Africa | 329327 (290231-378457) | 1176.42 (1025.96-1353.77) | 626863 (565016-698805) | 1115.92 (1007.14-1249.47) | -0.16 (-0.18 to -0.14) | <0.001 |
| Western Sub-Saharan Africa | 1232370 (1100877-1378607) | 1309.22 (1159.08-1464.82) | 3002279 (2723834-3278186) | 1340.06 (1211.16-1473.27) | 0.08 (0.06 to 0.09) | <0.001 |

Abbreviations: AAPC, average annual percentage change; SDI, Socio-Demographic Index.

**Table S9.** The number and age-standardized rate of YLDs for CKD-associated anemia and its AAPC from

1990 to 2021 at the global and regional levels.

|  | Number of cases, 1990 | Age-standardized rate per 100 000 population, 1990 | Number of cases, 2021 | Age-standardized rate per 100 000 population, 2021 | Average annual percentage change, 1990-2021 | p value |
| --- | --- | --- | --- | --- | --- | --- |
| Global | 972375 (650009-1385335) | 25.09 (16.78-35.65) | 1699516 (1131250-2433689) | 20.34 (13.54-29.09) | -0.67 (-0.71 to -0.62) | <0.001 |
| Sex |  |  |  |  |  |  |
| Male | 363831 (242840-518598) | 22.38 (14.95-31.57) | 580117 (379681-841698) | 15.81 (10.36-22.71) | -1.11  (-1.16 to -1.06) | <0.001 |
| Female | 608543 (407384-867082) | 28.29 (18.89-40.31) | 1119399 (746450-1608623) | 24.83 (16.56-35.73) | -0.41  (-0.45 to -0.37) | <0.001 |
| SDI region |  |  |  |  |  |  |
| High SDI | 116697 (73289-172849) | 10.86 (6.86-16.08) | 219456 (138759-328837) | 9.48 (5.96-14.33) | -0.44 (-0.47 to -0.40) | <0.001 |
| High-middle SDI | 161892 (105819-233309) | 17.96 (11.79-25.63) | 212507 (136908-309323) | 11.37 (7.33-16.54) | -1.46 (-1.50 to -1.42) | <0.001 |
| Middle SDI | 301289 (201303-429323) | 30.69 (20.54-43.39) | 535887 (353697-770788) | 21.55 (14.24-30.81) | -1.13 (-1.20 to -1.05) | <0.001 |
| Low-middle SDI | 284512 (192875-401690) | 46.64 (31.74-65.62) | 528032 (357456-744046) | 38.05 (25.87-53.82) | -0.65 (-0.68 to -0.62) | <0.001 |
| Low SDI | 107237 (72771-149542) | 45.82 (31.06-63.05) | 202492 (136829-287497) | 38.72 (26.28-54.37) | -0.54 (-0.57 to -0.51) | <0.001 |
| Region |  |  |  |  |  |  |
| Central Asia | 24083 (15983-35378) | 49.41 (32.85-72.23) | 36107 (23378-53926) | 43.68 (28.66-64.84) | -0.40 (-0.43 to -0.38) | <0.001 |
| Central Europe | 21561 (13946-31508) | 16.39 (10.63-23.79) | 25270 (16262-37491) | 12.21 (7.83-18.33) | -0.95 (-0.99 to -0.92) | <0.001 |
| Eastern Europe | 45404 (29051-68026) | 18.25 (11.77-26.93) | 49613 (31217-72897) | 14.71 (9.26-21.55) | -0.70 (-0.75 to -0.64) | <0.001 |
| Australasia | 1906 (1207-2847) | 8.75 (5.53-12.88) | 4173 (2526-6419) | 6.85 (4.18-10.5) | -0.77 (-0.87 to -0.67) | <0.001 |
| High-income Asia Pacific | 24298 (14718-37731) | 13.73 (8.47-21.01) | 62594 (38435-93818) | 9.95 (6.02-15.22) | -1.04 (-1.07 to -1.00) | <0.001 |
| High-income North America | 35452 (22429-52116) | 9.80 (6.21-14.47) | 73173 (46001-111686) | 10.86 (6.8-16.52) | 0.34 (0.27 to 0.40) | <0.001 |
| Southern Latin America | 3845 (2454-5827) | 9.18 (5.88-13.72) | 6411 (4091-9751) | 7.14 (4.54-10.87) | -0.81 (-0.86 to -0.77) | <0.001 |
| Western Europe | 47137 (29475-70001) | 8.32 (5.17-12.49) | 67143 (42230-101645) | 5.98 (3.73-9.09) | -1.06 (-1.09 to -1.02) | <0.001 |
| Andean Latin America | 3198 (2049-4677) | 13.82 (8.88-19.94) | 4878 (3097-7265) | 8.24 (5.23-12.23) | -1.65 (-1.68 to -1.62) | <0.001 |
| Caribbean | 4374 (2877-6513) | 17.14 (11.33-25.62) | 8176 (5285-12480) | 15.12 (9.77-22.9) | -0.41 (-0.44 to -0.37) | <0.001 |
| Central Latin America | 12862 (8328-18875) | 15.71 (10.18-22.77) | 31974 (20712-47500) | 13.18 (8.54-19.54) | -0.57 (-0.59 to -0.55) | <0.001 |
| Tropical Latin America | 22703 (14572-33475) | 25.29 (16.29-37.66) | 44582 (27738-68794) | 17.77 (11.04-27.49) | -1.13 (-1.20 to -1.06) | <0.001 |
| North Africa and Middle East | 46618 (30686-66454) | 25.79 (17.16-36.41) | 87592 (58046-126409) | 19.59 (13.04-28.19) | -0.87 (-0.90 to -0.84) | <0.001 |
| South Asia | 335078 (228658-465434) | 58.91 (40.28-81.82) | 655301 (443569-918707) | 46.47 (31.70-65.28) | -0.76 (-0.79 to -0.73) | <0.001 |
| East Asia | 176064 (116720-249045) | 23.83 (15.95-33.91) | 202914 (132596-290845) | 10.61 (6.93-15.12) | -2.57 (-2.62 to -2.51) | <0.001 |
| Oceania | 921 (594-1342) | 33.16 (22.04-47.06) | 2188 (1432-3266) | 31.94 (20.89-47.52) | -0.12 (-0.14 to -0.10) | <0.001 |
| Southeast Asia | 81832 (53750-117033) | 34.08 (22.35-48.60) | 166644 (108276-241395) | 28.79 (18.78-41.46) | -0.53 (-0.57 to -0.49) | <0.001 |
| Central Sub-Saharan Africa | 14027 (9389-20298) | 58.27 (39.62-82.80) | 26839 (17650-39070) | 45.70 (29.87-65.92) | -0.78 (-0.80 to -0.76) | <0.001 |
| Eastern Sub-Saharan Africa | 15536 (10362-22157) | 18.79 (12.69-26.72) | 27264 (17903-39910) | 14.72 (9.78-21.29) | -0.79 (-0.80 to -0.77) | <0.001 |
| Southern Sub-Saharan Africa | 9591 (6228-14114) | 33.07 (21.67-48.50) | 17405 (11342-25197) | 29.87 (19.50-43.35) | -0.32 (-0.37 to -0.27) | <0.001 |
| Western Sub-Saharan Africa | 45885 (31014-65723) | 48.75 (32.77-70.46) | 99278 (65592-142655) | 44.43 (29.57-63.25) | -0.30 (-0.31 to -0.28) | <0.001 |

Abbreviations: YLDs, years lived with disability; CKD, chronic kidney disease; AAPC, average annual percentage

change; SDI, Socio-Demographic Index.

**Table S10.** The number and age-standardized rates of prevalence and YLD for CKD-associated anemia from 1990 to 2021

at the global level.

| Year | Prevalence | |  | YLD | |
| --- | --- | --- | --- | --- | --- |
|  | Number of cases | Age-standardized rate per 100 000 population |  | Number of cases | Age-standardized rate per 100 000 population |
| 1990 | 32486224 (30356876-35047084) | 841.18 (783.12-906.48) |  | 972375 (650009-1385335) | 25.09 (16.78-35.65) |
| 1991 | 32899291 (30754770-35504212) | 832.14 (775.17-896.08) |  | 983566 (658187-1402193) | 24.79 (16.58-35.23) |
| 1992 | 33308874 (31141298-35949388) | 823.37 (767.45-886.62) |  | 994623 (666023-1418911) | 24.49 (16.37-34.83) |
| 1993 | 33724979 (31532457-36388533) | 815.20 (761.02-879.31) |  | 1006009 (674341-1435037) | 24.22 (16.22-34.44) |
| 1994 | 34176468 (31980823-36859405) | 807.96 (754.81-871.65) |  | 1018388 (681791-1452095) | 23.98 (16.04-34.09) |
| 1995 | 34684548 (32479942-37386407) | 802.10 (749.51-865.54) |  | 1032564 (691820-1472519) | 23.78 (15.91-33.82) |
| 1996 | 35208694 (32991462-37966927) | 796.28 (745.00-858.14) |  | 1046396 (700879-1492975) | 23.57 (15.76-33.53) |
| 1997 | 35701935 (33462322-38456792) | 789.42 (739.14-850.44) |  | 1058431 (709572-1508842) | 23.31 (15.6-33.12) |
| 1998 | 36200707 (33920956-38965980) | 782.59 (732.65-842.05) |  | 1069836 (717714-1523957) | 23.03 (15.42-32.72) |
| 1999 | 36758902 (34438251-39543619) | 776.64 (726.53-834.74) |  | 1083154 (727288-1544316) | 22.79 (15.27-32.41) |
| 2000 | 37423938 (35043169-40234048) | 772.40 (722.00-830.48) |  | 1100371 (738338-1569828) | 22.63 (15.14-32.19) |
| 2001 | 38203941 (35751518-41043236) | 769.80 (718.59-828.12) |  | 1121156 (750724-1599900) | 22.51 (15.07-32.03) |
| 2002 | 39042649 (36522069-41866239) | 767.94 (716.80-825.45) |  | 1142929 (765517-1632279) | 22.40 (14.99-31.91) |
| 2003 | 39917927 (37302828-42773302) | 766.57 (716.26-823.70) |  | 1165228 (779939-1663540) | 22.30 (14.93-31.75) |
| 2004 | 40844863 (38154770-43773999) | 765.43 (714.63-821.93) |  | 1188487 (795944-1698984) | 22.20 (14.87-31.64) |
| 2005 | 41807083 (39023414-44802444) | 764.19 (712.92-819.95) |  | 1212233 (811685-1733756) | 22.09 (14.80-31.50) |
| 2006 | 42793574 (39957021-45834877) | 762.14 (710.92-817.31) |  | 1235785 (828975-1765593) | 21.94 (14.72-31.27) |
| 2007 | 43789300 (40850570-46883455) | 759.36 (708.55-814.52) |  | 1257708 (841556-1798456) | 21.75 (14.56-31.01) |
| 2008 | 44804322 (41762994-47940383) | 756.62 (706.27-811.12) |  | 1278909 (857555-1829195) | 21.54 (14.45-30.72) |
| 2009 | 45904083 (42755563-49154069) | 754.56 (703.87-808.48) |  | 1302657 (872455-1861549) | 21.36 (14.34-30.45) |
| 2010 | 47127176 (43877331-50469896) | 754.07 (702.81-807.50) |  | 1330695 (891890-1901535) | 21.25 (14.27-30.28) |
| 2011 | 48500707 (45155181-51898904) | 755.04 (703.58-807.64) |  | 1363112 (913176-1948173) | 21.19 (14.22-30.21) |
| 2012 | 49984013 (46542390-53447651) | 756.60 (705.08-808.73) |  | 1396628 (933374-1995848) | 21.12 (14.15-30.12) |
| 2013 | 51520453 (48011970-55088522) | 758.64 (706.86-810.53) |  | 1430648 (953859-2044523) | 21.05 (14.08-30.03) |
| 2014 | 53076654 (49502125-56699204) | 760.54 (708.96-812.32) |  | 1464039 (976220-2092937) | 20.97 (14.03-29.93) |
| 2015 | 54605780 (50955728-58298018) | 762.01 (710.75-814.32) |  | 1497550 (997327-2141834) | 20.89 (13.96-29.84) |
| 2016 | 56108455 (52359929-59870545) | 762.20 (711.10-813.55) |  | 1529511 (1017904-2186712) | 20.78 (13.87-29.67) |
| 2017 | 57568616 (53664015-61494465) | 761.08 (709.25-812.80) |  | 1557362 (1033853-2229562) | 20.59 (13.70-29.45) |
| 2018 | 59042235 (54879114-63175865) | 759.79 (707.61-813.00) |  | 1585598 (1051956-2271294) | 20.42 (13.56-29.20) |
| 2019 | 60647734 (56366870-64945879) | 759.40 (706.37-814.64) |  | 1620787 (1076723-2319355) | 20.31 (13.51-29.04) |
| 2020 | 62290779 (57813200-66821005) | 760.96 (706.89-816.64) |  | 1664866 (1108402-2383206) | 20.36 (13.57-29.11) |
| 2021 | 63751624 (59045051-68372650) | 762.12 (707.32-817.37) |  | 1699516 (1131250-2433689) | 20.34 (13.54-29.09) |

Abbreviations: YLDs, years lived with disability; CKD, chronic kidney disease.

**Table S11.** Global AAPCs in the age-standardized rates of prevalence and YLDs for CKD-associated anemia.

|  | Prevalence | |  | YLD | |
| --- | --- | --- | --- | --- | --- |
|  | AAPC (95% CI) | p value |  | AAPC (95% CI) | p value |
| 1990-1999 | -0.87 (-0.91 to -0.82) | < 0.001 |  | -1.05 (-1.08 to -1.02) | < 0.001 |
| 2000-2009 | -0.24 (-0.26 to -0.21) | < 0.001 |  | -0.66 (-0.77 to -0.56) | < 0.001 |
| 2010-2021 | -0.10 (0.03 to 0.17) | 0.004 |  | -0.40 (-0.48 to -0.32) | < 0.001 |
| 1990-2021 | -0.32 (-0.35 to -0.29) | < 0.001 |  | -0.67 (-0.71 to -0.62) | < 0.001 |

Abbreviations: AAPC, average annual percentage change; YLDs, years lived with disability; CI, confidence interval.

**Table S12.** The number and age-standardized rate of incidence for chronic kidney disease in 1990 and 2021, and the temporal trends between 1990-2021 in 204 countries and territories.

| Location | Number of cases, 1990 | Age-standardized rate per 100,000 population, 1990 | Number of cases, 2021 | Age-standardized rate per 100,000 population, 2021 | Average annual percentage change, 1990-2021 | p value |
| --- | --- | --- | --- | --- | --- | --- |
| Afghanistan | 18833 (16975-21041) | 255.14 (230.97-282.18) | 45514 (42294-49787) | 367.19 (338.49-402.22) | 1.19 (1.07 to 1.32) | < 0.001 |
| Albania | 2815 (2542-3126) | 120.75 (108.14-134.53) | 8621 (7734-9518) | 206.70 (188.54-225.03) | 1.76 (1.70 to 1.81) | < 0.001 |
| Algeria | 35771 (32578-39279) | 256.61 (233.40-281.88) | 153075 (140677-165614) | 407.45 (375.52-438.80) | 1.50 (1.45 to 1.55) | < 0.001 |
| American Samoa | 61 (57-66) | 212.79 (195.75-230.96) | 156 (143-171) | 312.56 (289.11-337.20) | 1.28 (1.20 to 1.35) | < 0.001 |
| Andorra | 126 (113-142) | 224.52 (203.43-250.77) | 336 (307-370) | 222.64 (202.74-245.42) | 0.02 (-0.03 to 0.08) | 0.428 |
| Angola | 4839 (4507-5162) | 89.54 (82.64-97.10) | 19883 (18458-21540) | 129.13 (118.15-140.51) | 1.19 (1.13 to 1.25) | < 0.001 |
| Antigua and Barbuda | 114 (105-124) | 219.08 (200.99-238.07) | 375 (343-409) | 344.75 (318.73-371.78) | 1.50 (1.43 to 1.56) | < 0.001 |
| Argentina | 68543 (62476-75476) | 213.61 (195.31-234.45) | 150963 (137930-163854) | 269.56 (246.95-290.68) | 0.75 (0.72 to 0.79) | < 0.001 |
| Armenia | 2819 (2506-3157) | 94.28 (84.40-105.10) | 7532 (6762-8397) | 184.33 (168.31-201.69) | 2.20 (2.16 to 2.24) | < 0.001 |
| Australia | 51807 (49084-54962) | 258.05 (245.00-272.10) | 135305 (123723-145659) | 296.27 (272.00-317.25) | 0.45 (0.42 to 0.49) | < 0.001 |
| Austria | 27499 (24724-30726) | 224.60 (203.96-247.29) | 50647 (46366-55368) | 275.88 (249.36-302.98) | 0.68 (0.65 to 0.71) | < 0.001 |
| Azerbaijan | 6575 (5913-7257) | 110.18 (99.67-121.02) | 22387 (19939-24791) | 203.81 (187.05-221.06) | 2.02 (1.98 to 2.05) | < 0.001 |
| Bahamas | 346 (321-374) | 197.33 (182.27-216.20) | 1252 (1141-1353) | 295.86 (274.03-317.19) | 1.33 (1.26 to 1.41) | < 0.001 |
| Bahrain | 612 (557-676) | 292.78 (268.00-320.81) | 4873 (4361-5457) | 451.21 (413.39-487.18) | 1.40 (1.37 to 1.44) | < 0.001 |
| Bangladesh | 52608 (48339-57513) | 89.98 (81.87-99.39) | 187707 (170316-207797) | 131.83 (119.95-144.85) | 1.25 (1.20 to 1.31) | < 0.001 |
| Barbados | 523 (480-576) | 194.41 (178.58-213.82) | 1473 (1348-1605) | 308.00 (286.36-332.91) | 1.50 (1.43 to 1.58) | < 0.001 |
| Belarus | 10524 (9525-11767) | 87.22 (79.76-96.22) | 20010 (18173-22131) | 141.29 (128.88-154.23) | 1.57 (1.46 to 1.68) | < 0.001 |
| Belgium | 38016 (34545-42363) | 240.48 (220.28-264.10) | 58480 (53425-63159) | 247.39 (226.54-265.93) | 0.10 (0.08 to 0.11) | < 0.001 |
| Belize | 213 (197-231) | 193.55 (176.10-212.41) | 1052 (972-1129) | 312.05 (288.73-335.71) | 1.56 (1.48 to 1.65) | < 0.001 |
| Benin | 3701 (3454-3984) | 135.76 (124.78-147.44) | 12189 (11329-13016) | 177.75 (162.42-192.63) | 0.88 (0.82 to 0.95) | < 0.001 |
| Bermuda | 121 (110-133) | 192.65 (175.32-211.44) | 392 (356-428) | 321.10 (295.63-348.34) | 1.67 (1.61 to 1.74) | < 0.001 |
| Bhutan | 425 (391-459) | 125.40 (115.46-137.73) | 1197 (1094-1305) | 185.79 (169.68-202.65) | 1.28 (1.26 to 1.31) | < 0.001 |
| Bolivia (Plurinational State of) | 6471 (5994-6956) | 175.99 (162.00-190.05) | 24667 (22843-26922) | 262.38 (242.50-285.89) | 1.31 (1.26 to 1.35) | < 0.001 |
| Bosnia and Herzegovina | 5153 (4623-5700) | 121.04 (109.70-132.48) | 13515 (12363-14759) | 227.92 (209.44-246.38) | 2.07 (2.03 to 2.12) | < 0.001 |
| Botswana | 1010 (928-1097) | 151.59 (138.71-165.30) | 3507 (3222-3805) | 217.22 (199.90-237.03) | 1.17 (1.10 to 1.24) | < 0.001 |
| Brazil | 189483 (174873-205093) | 192.91 (176.91-209.57) | 644744 (602333-688117) | 258.11 (241.70-274.48) | 0.94 (0.90 to 0.99) | < 0.001 |
| Brunei Darussalam | 322 (296-352) | 295.01 (269.33-322.69) | 1126 (1022-1250) | 328.34 (302.52-357.58) | 0.34 (0.31 to 0.38) | < 0.001 |
| Bulgaria | 15334 (13681-17204) | 129.03 (117.32-142.46) | 29662 (26863-32623) | 232.22 (214.99-250.97) | 1.92 (1.87 to 1.97) | < 0.001 |
| Burkina Faso | 7168 (6643-7771) | 126.47 (116.58-138.30) | 20261 (19126-21650) | 165.80 (153.53-179.63) | 0.89 (0.82 to 0.96) | < 0.001 |
| Burundi | 2856 (2658-3083) | 93.91 (86.38-102.24) | 6740 (6244-7274) | 109.68 (101.01-119.57) | 0.50 (0.47 to 0.53) | < 0.001 |
| Cabo Verde | 304 (280-331) | 114.82 (105.2-125.22) | 816 (742-885) | 179.47 (162.80-195.61) | 1.47 (1.38 to 1.56) | < 0.001 |
| Cambodia | 7527 (6954-8200) | 126.42 (115.83-139.56) | 24542 (22491-27011) | 183.25 (168.50-200.91) | 1.22 (1.16 to 1.28) | < 0.001 |
| Cameroon | 10676 (9919-11509) | 178.41 (163.99-194.72) | 39453 (36599-41901) | 236.22 (219.22-253.76) | 0.90 (0.83 to 0.98) | < 0.001 |
| Canada | 85191 (77748-90551) | 260.65 (239.01-276.27) | 184153 (167875-204154) | 256.43 (234.42-282.28) | -0.04 (-0.11 to 0.04) | 0.329 |
| Central African Republic | 1379 (1268-1499) | 91.18 (83.57-99.19) | 3387 (3140-3657) | 118.17 (109.20-128.46) | 0.84 (0.78 to 0.90) | < 0.001 |
| Chad | 4705 (4391-5083) | 127.55 (117.01-139.45) | 13105 (12245-14003) | 154.87 (142.44-168.79) | 0.63 (0.55 to 0.71) | < 0.001 |
| Chile | 21716 (19660-24017) | 216.57 (196.57-239.17) | 80300 (73877-86347) | 316.55 (292.45-340.00) | 1.26 (1.18 to 1.33) | < 0.001 |
| China | 1267533 (1145753-1385805) | 147.29 (133.86-161.46) | 3323175 (3068978-3559324) | 163.74 (153.03-174.11) | 0.31 (0.21 to 0.41) | < 0.001 |
| Colombia | 45649 (41528-50029) | 227.23 (206.64-250.95) | 171450 (156961-186839) | 314.56 (289.42-341.07) | 1.07 (1.05 to 1.10) | < 0.001 |
| Comoros | 268 (249-290) | 103.43 (95.25-112.63) | 672 (627-725) | 127.50 (118.48-138.78) | 0.68 (0.64 to 0.72) | < 0.001 |
| Congo | 1393 (1291-1512) | 102.25 (94.45-111.22) | 4645 (4325-5062) | 147.09 (136.70-160.00) | 1.18 (1.10 to 1.26) | < 0.001 |
| Cook Islands | 24 (22-26) | 174.21 (158.51-190.06) | 70 (63-76) | 284.54 (260.54-308.16) | 1.60 (1.55 to 1.65) | < 0.001 |
| Costa Rica | 8200 (7966-8432) | 436.29 (425.05-446.44) | 24923 (24422-25363) | 454.46 (445.16-462.62) | 0.13 (0.13 to 0.14) | < 0.001 |
| Coted'Ivoire | 9012 (8352-9722) | 150.85 (139.01-164.29) | 28314 (26275-30296) | 193.17 (178.04-209.93) | 0.81 (0.71 to 0.92) | < 0.001 |
| Croatia | 9145 (8175-10238) | 150.56 (136.31-166.12) | 21856 (19855-23659) | 259.73 (238.67-279.34) | 1.82 (1.71 to 1.92) | < 0.001 |
| Cuba | 15078 (13825-16515) | 146.81 (133.91-160.69) | 49193 (44769-53427) | 271.14 (248.75-295.36) | 2.01 (1.95 to 2.07) | < 0.001 |
| Cyprus | 2321 (2070-2607) | 275.50 (254.54-300.59) | 6413 (5860-7052) | 296.02 (272.09-324.03) | 0.25 (0.22 to 0.28) | < 0.001 |
| Czechia | 17191 (15474-19227) | 129.98 (118.05-143.63) | 44762 (40513-49824) | 217.99 (200.55-238.47) | 1.69 (1.65 to 1.73) | < 0.001 |
| Democratic People's Republic of Korea | 23090 (20851-25212) | 138.39 (125.15-150.99) | 58636 (52975-64306) | 185.31 (168.51-201.74) | 0.94 (0.88 to 1.00) | < 0.001 |
| Democratic Republic of the Congo | 19562 (18062-21127) | 94.59 (86.76-103.22) | 57306 (53005-62101) | 128.30 (117.98-140.77) | 1.00 (0.96 to 1.04) | < 0.001 |
| Denmark | 17126 (15298-19107) | 207.06 (187.32-228.28) | 29642 (27034-32472) | 243.44 (224.01-263.12) | 0.53 (0.51 to 0.55) | < 0.001 |
| Djibouti | 185 (171-200) | 98.69 (90.60-107.55) | 936 (862-1011) | 128.56 (117.96-139.00) | 0.86 (0.80 to 0.91) | < 0.001 |
| Dominica | 139 (129-151) | 229.62 (212.37-247.74) | 262 (238-285) | 320.81 (295.72-345.22) | 1.09 (1.02 to 1.17) | < 0.001 |
| Dominican Republic | 6202 (5697-6821) | 139.23 (126.43-153.72) | 24963 (23057-26895) | 243.01 (223.96-261.73) | 1.84 (1.78 to 1.89) | < 0.001 |
| Ecuador | 10748 (9822-11799) | 180.68 (163.64-199.76) | 57934 (53462-63236) | 348.19 (321.29-379.96) | 2.16 (2.10 to 2.21) | < 0.001 |
| Egypt | 80783 (73484-89103) | 263.81 (240.7-289.49) | 301550 (272715-333684) | 428.76 (397.28-465.61) | 1.58 (1.54 to 1.62) | < 0.001 |
| El Salvador | 7712 (7063-8457) | 229.52 (209.07-253.91) | 24978 (23302-26600) | 408.39 (379.98-435.70) | 1.88 (1.83 to 1.94) | < 0.001 |
| Equatorial Guinea | 227 (210-246) | 92.55 (85.25-101.49) | 1065 (987-1146) | 165.20 (151.27-180.45) | 1.92 (1.81 to 2.02) | < 0.001 |
| Eritrea | 1470 (1358-1586) | 91.20 (83.99-99.69) | 3984 (3698-4299) | 116.86 (106.79-127.37) | 0.81 (0.77 to 0.84) | < 0.001 |
| Estonia | 1993 (1779-2238) | 104.45 (94.27-116.27) | 4909 (4493-5418) | 215.89 (198.03-235.66) | 2.38 (2.32 to 2.43) | < 0.001 |
| Eswatini | 689 (635-750) | 184.48 (169.23-202.93) | 1568 (1441-1700) | 232.86 (212.29-252.21) | 0.76 (0.68 to 0.84) | < 0.001 |
| Ethiopia | 25075 (23288-27040) | 92.87 (85.97-100.67) | 60892 (56785-64771) | 114.61 (104.99-124.17) | 0.67 (0.61 to 0.73) | < 0.001 |
| Fiji | 881 (811-959) | 195.28 (179.74-212.95) | 2163 (1978-2345) | 261.89 (243.66-283.22) | 0.95 (0.92 to 0.99) | < 0.001 |
| Finland | 12464 (11114-14015) | 171.14 (154.02-189.57) | 25918 (23654-28781) | 195.36 (180.13-214.08) | 0.43 (0.41 to 0.45) | < 0.001 |
| France | 179845 (162635-201949) | 207.47 (188.80-229.56) | 326611 (300075-352984) | 229.79 (212.38-249.84) | 0.34 (0.32 to 0.36) | < 0.001 |
| Gabon | 755 (698-818) | 115.79 (106.83-125.64) | 2115 (1944-2293) | 182.33 (168.31-196.86) | 1.50 (1.43 to 1.56) | < 0.001 |
| Gambia | 662 (623-706) | 133.86 (123.59-144.69) | 2198 (2064-2346) | 176.56 (162.39-191.77) | 0.90 (0.84 to 0.96) | < 0.001 |
| Georgia | 6362 (5720-7116) | 105.04 (95.24-116.56) | 9241 (8320-10163) | 177.75 (164.07-193.02) | 1.72 (1.67 to 1.77) | < 0.001 |
| Germany | 312633 (283719-347849) | 242.78 (222.26-269.74) | 526670 (491104-565042) | 269.85 (254.46-287.45) | 0.36 (0.33 to 0.39) | < 0.001 |
| Ghana | 9860 (9230-10595) | 120.08 (111.48-130.61) | 34499 (31872-37394) | 171.14 (157.26-186.33) | 1.18 (1.08 to 1.27) | < 0.001 |
| Greece | 44006 (40001-48285) | 283.94 (258.67-310.51) | 65278 (60702-70955) | 274.86 (254.18-297.79) | -0.10 (-0.13 to -0.06) | < 0.001 |
| Greenland | 60 (54-65) | 200.98 (181.77-220.52) | 149 (136-165) | 231.05 (213.58-252.80) | 0.46 (0.41 to 0.51) | < 0.001 |
| Grenada | 149 (139-160) | 210.49 (194.11-227.44) | 413 (380-447) | 355.05 (328.52-380.04) | 1.70 (1.66 to 1.75) | < 0.001 |
| Guam | 166 (152-183) | 180.83 (165.51-199.06) | 534 (488-587) | 265.31 (244.89-289.50) | 1.26 (1.23 to 1.29) | < 0.001 |
| Guatemala | 10599 (9674-11591) | 244.05 (221.48-267.00) | 45885 (42467-49772) | 387.90 (358.04-420.42) | 1.52 (1.49 to 1.55) | < 0.001 |
| Guinea | 5459 (5060-5901) | 134.10 (123.08-146.22) | 12458 (11579-13322) | 171.10 (157.52-184.40) | 0.79 (0.73 to 0.84) | < 0.001 |
| Guinea-Bissau | 795 (741-851) | 142.90 (133.39-154.42) | 1762 (1638-1884) | 170.44 (157.21-184.07) | 0.57 (0.52 to 0.63) | < 0.001 |
| Guyana | 836 (776-899) | 177.15 (163.43-191.62) | 2046 (1880-2219) | 294.58 (273.70-317.60) | 1.65 (1.58 to 1.72) | < 0.001 |
| Haiti | 6772 (6073-7507) | 170.38 (152.56-191.54) | 19290 (17777-20946) | 225.97 (207.08-245.88) | 0.92 (0.90 to 0.94) | < 0.001 |
| Honduras | 5948 (5452-6582) | 236.15 (214.11-264.73) | 22686 (20945-24585) | 320.50 (295.77-348.50) | 1.00 (0.97 to 1.02) | < 0.001 |
| Hungary | 15676 (14056-17503) | 111.68 (101.48-123.79) | 42185 (38412-46628) | 227.65 (209.30-248.77) | 2.35 (2.30 to 2.39) | < 0.001 |
| Iceland | 560 (509-616) | 192.18 (175.12-210.84) | 1166 (1066-1264) | 199.78 (182.82-216.94) | 0.13 (0.09 to 0.18) | < 0.001 |
| India | 849302 (780462-920171) | 152.76 (140.36-165.72) | 2235892 (2056366-2414555) | 180.72 (166.53-195.39) | 0.54 (0.44 to 0.64) | < 0.001 |
| Indonesia | 155644 (144275-167963) | 130.27 (120.15-141.66) | 469570 (432377-511150) | 185.09 (171.22-200.10) | 1.13 (1.04 to 1.21) | < 0.001 |
| Iran (Islamic Republic of) | 93053 (83778-103125) | 304.23 (277.32-334.65) | 330887 (308639-354798) | 408.97 (382.61-436.00) | 0.95 (0.90 to 1.00) | < 0.001 |
| Iraq | 27066 (24993-29348) | 291.50 (267.28-319.75) | 117615 (108689-127825) | 440.28 (407.19-473.91) | 1.35 (1.27 to 1.42) | < 0.001 |
| Ireland | 11405 (10693-11949) | 273.96 (257.21-285.84) | 19201 (18876-19506) | 245.55 (242.28-248.78) | -0.33 (-0.36 to -0.30) | < 0.001 |
| Israel | 13680 (12393-15202) | 273.62 (249.31-301.51) | 37867 (34116-41515) | 305.76 (275.26-333.62) | 0.38 (0.32 to 0.44) | < 0.001 |
| Italy | 195977 (175595-217213) | 217.42 (196.25-239.32) | 333142 (306700-361005) | 218.54 (202.72-235.12) | 0.03 (0.00 to 0.07) | 0.044 |
| Jamaica | 3710 (3408-4045) | 203.90 (186.17-222.80) | 8989 (8236-9704) | 295.44 (271.45-318.44) | 1.22 (1.16 to 1.28) | < 0.001 |
| Japan | 471525 (434120-511167) | 280.03 (259.15-302.10) | 996708 (911293-1081250) | 285.18 (263.72-305.66) | 0.08 (0.04 to 0.11) | < 0.001 |
| Jordan | 4664 (4322-5035) | 289.60 (266.65-312.65) | 37316 (34745-40073) | 448.81 (420.86-478.15) | 1.42 (1.39 to 1.46) | < 0.001 |
| Kazakhstan | 14602 (13439-15891) | 101.76 (93.51-111.24) | 35051 (31601-38749) | 183.92 (168.89-201.62) | 1.93 (1.87 to 1.99) | < 0.001 |
| Kenya | 10255 (9567-10957) | 92.88 (85.46-101.07) | 31651 (29148-34092) | 118.9 (109.21-129.48) | 0.80 (0.70 to 0.90) | < 0.001 |
| Kiribati | 77 (71-84) | 154.97 (142.78-169.03) | 192 (177-207) | 215.92 (199.81-232.07) | 1.07 (1.05 to 1.09) | < 0.001 |
| Kuwait | 2252 (2070-2441) | 293.05 (271.19-317.02) | 13109 (11785-14632) | 397.04 (363.69-431.75) | 1.00 (0.92 to 1.08) | < 0.001 |
| Kyrgyzstan | 3991 (3710-4283) | 105.47 (97.40-113.97) | 8823 (8020-9622) | 151.01 (137.96-164.46) | 1.17 (1.15 to 1.20) | < 0.001 |
| Lao People's Democratic Republic | 4381 (4022-4785) | 169.68 (155.23-185.94) | 12021 (11060-12971) | 227.71 (209.17-246.18) | 0.99 (0.94 to 1.04) | < 0.001 |
| Latvia | 2877 (2583-3214) | 87.64 (79.51-96.72) | 5423 (4898-6022) | 165.91 (152.48-181.71) | 2.07 (2.01 to 2.14) | < 0.001 |
| Lebanon | 6124 (5556-6781) | 267.39 (245.37-292.65) | 25285 (23327-27213) | 435.30 (397.96-470.82) | 1.60 (1.52 to 1.67) | < 0.001 |
| Lesotho | 1419 (1305-1543) | 149.85 (137.2-163.67) | 2455 (2265-2646) | 201.81 (186.09-216.67) | 0.98 (0.92 to 1.03) | < 0.001 |
| Liberia | 1959 (1816-2115) | 130.28 (119.69-142.17) | 4919 (4558-5317) | 177.94 (161.69-194.48) | 1.04 (1.00 to 1.09) | < 0.001 |
| Libya | 5633 (5189-6163) | 262.39 (239.49-288.31) | 23278 (21519-25396) | 417.75 (384.87-453.56) | 1.52 (1.40 to 1.63) | < 0.001 |
| Lithuania | 4205 (3814-4676) | 99.04 (90.44-109.62) | 6974 (6366-7568) | 147.64 (137.04-159.15) | 1.29 (1.21 to 1.36) | < 0.001 |
| Luxembourg | 1367 (1246-1511) | 243.76 (223.33-267.27) | 2759 (2529-2961) | 263.07 (240.94-282.99) | 0.27 (0.19 to 0.35) | < 0.001 |
| Madagascar | 5781 (5385-6208) | 86.15 (79.26-93.66) | 15047 (13993-16361) | 107.00 (97.67-116.36) | 0.72 (0.66 to 0.77) | < 0.001 |
| Malawi | 4890 (4550-5238) | 96.85 (89.11-105.85) | 11239 (10343-11977) | 124.02 (112.77-133.90) | 0.81 (0.79 to 0.83) | < 0.001 |
| Malaysia | 18546 (17026-20013) | 175.49 (159.31-190.28) | 77138 (70879-83439) | 259.98 (240.09-281.17) | 1.32 (1.23 to 1.42) | < 0.001 |
| Maldives | 214 (193-237) | 186.89 (169.21-206.05) | 1059 (952-1155) | 288.32 (259.90-317.90) | 1.43 (1.39 to 1.47) | < 0.001 |
| Mali | 6631 (6181-7134) | 125.11 (115.55-135.77) | 19506 (18189-20764) | 159.68 (147.15-172.12) | 0.80 (0.73 to 0.87) | < 0.001 |
| Malta | 1095 (1000-1202) | 256.24 (235.59-279.70) | 2663 (2436-2926) | 266.70 (246.75-289.77) | 0.15 (0.12 to 0.18) | < 0.001 |
| Marshall Islands | 39 (36-42) | 167.21 (154.19-182.59) | 102 (93-110) | 245.16 (225.91-264.04) | 1.24 (1.21 to 1.27) | < 0.001 |
| Mauritania | 1888 (1754-2046) | 148.64 (136.98-161.33) | 5232 (4833-5667) | 200.89 (183.17-219.10) | 1.00 (0.95 to 1.06) | < 0.001 |
| Mauritius | 2071 (1884-2281) | 254.92 (231.38-279.25) | 7451 (6925-8055) | 406.79 (383.30-433.70) | 1.53 (1.49 to 1.57) | < 0.001 |
| Mexico | 151265 (138996-165600) | 304.82 (277.96-335.41) | 612577 (580429-645104) | 464.33 (440.38-488.10) | 1.39 (1.35 to 1.43) | < 0.001 |
| Micronesia (Federated States of) | 115 (106-126) | 186.54 (171.93-204.53) | 247 (223-269) | 290.81 (268.19-313.56) | 1.44 (1.41 to 1.47) | < 0.001 |
| Monaco | 156 (140-175) | 211.38 (191.51-233.27) | 227 (206-250) | 228.39 (209.34-249.89) | 0.27 (0.22 to 0.33) | < 0.001 |
| Mongolia | 2040 (1864-2228) | 139.64 (125.96-153.16) | 5269 (4830-5728) | 188.33 (171.50-203.80) | 0.98 (0.92 to 1.05) | < 0.001 |
| Montenegro | 1134 (1029-1251) | 181.18 (164.66-199.23) | 2639 (2368-2916) | 274.18 (249.16-301.37) | 1.35 (1.33 to 1.38) | < 0.001 |
| Morocco | 32617 (29938-35664) | 210.12 (191.81-230.84) | 135407 (123555-147157) | 384.26 (352.31-415.80) | 1.97 (1.86 to 2.08) | < 0.001 |
| Mozambique | 6980 (6451-7573) | 93.07 (85.24-102.04) | 17355 (16180-18602) | 118.65 (109.29-129.06) | 0.78 (0.74 to 0.83) | < 0.001 |
| Myanmar | 43845 (39884-48151) | 159.87 (145.82-176.19) | 116368 (106212-125983) | 227.58 (209.28-245.02) | 1.15 (1.12 to 1.19) | < 0.001 |
| Namibia | 1156 (1061-1260) | 153.57 (140.73-167.23) | 2985 (2740-3260) | 198.04 (181.92-217.06) | 0.83 (0.77 to 0.88) | < 0.001 |
| Nauru | 11 (10-12) | 179.92 (165.28-195.96) | 18 (17-20) | 256.09 (234.76-279.13) | 1.15 (1.11 to 1.18) | < 0.001 |
| Nepal | 14842 (13643-16160) | 124.26 (114.00-136.79) | 54613 (49880-59454) | 213.74 (195.45-232.37) | 1.75 (1.69 to 1.81) | < 0.001 |
| Netherlands | 44064 (40881-47584) | 213.48 (198.88-229.67) | 88460 (81320-95824) | 242.77 (224.71-261.33) | 0.44 (0.39 to 0.49) | < 0.001 |
| New Zealand | 10613 (9606-11747) | 264.73 (242.79-290.53) | 25356 (23124-27531) | 301.67 (277.33-327.31) | 0.42 (0.40 to 0.45) | < 0.001 |
| Nicaragua | 5618 (5153-6112) | 277.67 (252.08-306.69) | 24136 (22516-25614) | 438.24 (408.73-465.27) | 1.49 (1.47 to 1.51) | < 0.001 |
| Niger | 5267 (4896-5655) | 125.05 (115.67-136.13) | 17881 (16629-19193) | 152.09 (138.04-165.17) | 0.64 (0.61 to 0.67) | < 0.001 |
| Nigeria | 76055 (70880-81863) | 135.98 (125.16-148.38) | 210760 (196709-225476) | 181.33 (165.23-196.44) | 0.92 (0.85 to 1.00) | < 0.001 |
| Niue | 4 (4-4) | 182.65 (166.78-200.17) | 6 (5-6) | 277.86 (252.87-305.83) | 1.37 (1.34 to 1.40) | < 0.001 |
| North Macedonia | 2592 (2353-2867) | 135.20 (123.50-147.43) | 8708 (7721-9571) | 260.47 (236.73-282.51) | 2.15 (2.11 to 2.19) | < 0.001 |
| Northern Mariana Islands | 62 (55-68) | 239.87 (216.44-262.37) | 192 (170-217) | 340.32 (312.96-378.11) | 1.14 (1.11 to 1.18) | < 0.001 |
| Norway | 13125 (11763-14781) | 182.58 (166.38-202.55) | 21817 (19979-23671) | 212.31 (195.33-229.09) | 0.51 (0.46 to 0.57) | < 0.001 |
| Oman | 1754 (1614-1896) | 218.40 (197.23-237.94) | 9118 (8291-9915) | 396.60 (366.06-431.67) | 1.97 (1.82 to 2.13) | < 0.001 |
| Pakistan | 98898 (91761-107003) | 142.46 (131.44-155.25) | 275774 (256521-294773) | 181.88 (167.78-195.95) | 0.81 (0.76 to 0.87) | < 0.001 |
| Palau | 24 (21-27) | 213.24 (190.87-238.54) | 82 (74-93) | 348.84 (322.35-385.33) | 1.60 (1.54 to 1.65) | < 0.001 |
| Palestine | 2997 (2749-3268) | 290.30 (264.86-317.37) | 12369 (11405-13455) | 430.73 (398.67-464.21) | 1.29 (1.17 to 1.41) | < 0.001 |
| Panama | 3972 (3641-4354) | 243.65 (221.54-269.13) | 15956 (15016-17069) | 363.79 (341.46-389.29) | 1.35 (1.31 to 1.38) | < 0.001 |
| Papua New Guinea | 2466 (2243-2731) | 97.31 (87.81-108.55) | 8614 (7916-9315) | 128.73 (116.82-139.84) | 0.90 (0.84 to 0.96) | < 0.001 |
| Paraguay | 4761 (4373-5210) | 191.86 (174.61-210.72) | 18724 (17247-20324) | 307.76 (282.75-334.34) | 1.53 (1.50 to 1.57) | < 0.001 |
| Peru | 19680 (18106-21486) | 148.84 (136.14-163.48) | 95333 (88068-103496) | 284.09 (261.38-308.38) | 2.13 (2.07 to 2.19) | < 0.001 |
| Philippines | 66080 (61640-70710) | 184.45 (170.37-199.25) | 245592 (230483-262725) | 271.10 (254.37-289.22) | 1.29 (1.23 to 1.35) | < 0.001 |
| Poland | 63497 (56153-71143) | 148.95 (133.49-165.67) | 137937 (123815-154597) | 201.67 (183.51-223.14) | 1.00 (0.91 to 1.09) | < 0.001 |
| Portugal | 29572 (26316-33103) | 211.28 (190.16-233.78) | 60079 (54791-66350) | 236.41 (215.27-260.20) | 0.38 (0.35 to 0.40) | < 0.001 |
| Puerto Rico | 8257 (7527-9060) | 228.91 (208.57-251.43) | 21077 (19420-22999) | 354.68 (332.40-384.64) | 1.43 (1.40 to 1.47) | < 0.001 |
| Qatar | 463 (413-513) | 294.64 (267.04-326.89) | 6175 (5515-6879) | 467.39 (432.92-503.88) | 1.50 (1.46 to 1.53) | < 0.001 |
| Republic of Korea | 61023 (54616-68362) | 214.95 (193.76-239.29) | 226256 (210509-241377) | 242.84 (225.84-258.55) | 0.40 (0.37 to 0.44) | < 0.001 |
| Republic of Moldova | 2996 (2720-3309) | 69.38 (63.48-76.22) | 6551 (5870-7305) | 123.63 (112.70-136.18) | 1.86 (1.78 to 1.94) | < 0.001 |
| Romania | 30710 (27578-34445) | 113.06 (103.32-125.21) | 72834 (67186-78514) | 210.87 (196.04-225.81) | 2.05 (1.98 to 2.11) | < 0.001 |
| Russian Federation | 201892 (185153-222440) | 121.16 (112.76-131.68) | 422141 (385788-459285) | 201.91 (187.06-217.07) | 1.68 (1.63 to 1.73) | < 0.001 |
| Rwanda | 3596 (3326-3889) | 95.81 (88.13-104.85) | 8666 (7877-9399) | 120.75 (110.03-132.18) | 0.74 (0.72 to 0.76) | < 0.001 |
| Saint Kitts and Nevis | 81 (73-89) | 223.11 (203.62-245.66) | 253 (228-280) | 343.85 (319.49-374.16) | 1.42 (1.35 to 1.49) | < 0.001 |
| Saint Lucia | 190 (176-205) | 200.55 (185.46-216.19) | 736 (682-807) | 314.36 (293.07-343.46) | 1.47 (1.42 to 1.51) | < 0.001 |
| Saint Vincent and the Grenadines | 145 (134-157) | 189.14 (175.46-206.37) | 411 (373-445) | 294.08 (270.42-314.58) | 1.44 (1.39 to 1.49) | < 0.001 |
| Samoa | 196 (182-212) | 190.40 (176.36-206.44) | 433 (402-471) | 269.71 (250.26-292.63) | 1.15 (1.10 to 1.20) | < 0.001 |
| San Marino | 70 (63-79) | 194.97 (175.17-216.38) | 157 (141-174) | 207.35 (186.79-229.43) | 0.21 (0.18 to 0.24) | < 0.001 |
| Sao Tome and Principe | 130 (119-142) | 163.22 (148.02-178.81) | 325 (302-350) | 240.52 (222.60-260.76) | 1.28 (1.19 to 1.37) | < 0.001 |
| Saudi Arabia | 21157 (19638-22839) | 295.83 (272.14-320.38) | 123154 (113311-133632) | 495.83 (465.09-529.64) | 1.69 (1.62 to 1.75) | < 0.001 |
| Senegal | 5896 (5479-6341) | 136.13 (125.93-146.92) | 15909 (14807-17097) | 171.24 (158.95-185.48) | 0.75 (0.71 to 0.78) | < 0.001 |
| Serbia | 15572 (13985-17192) | 134.77 (123.15-146.90) | 39137 (35808-43025) | 246.20 (226.98-268.22) | 1.99 (1.94 to 2.04) | < 0.001 |
| Seychelles | 127 (117-138) | 217.23 (200.10-236.55) | 379 (345-414) | 315.56 (289.33-341.18) | 1.23 (1.19 to 1.27) | < 0.001 |
| Sierra Leone | 3353 (3108-3634) | 126.59 (115.64-139.00) | 7939 (7427-8505) | 163.15 (149.59-176.27) | 0.83 (0.77 to 0.90) | < 0.001 |
| Singapore | 5595 (5322-5869) | 254.11 (241.44-266.80) | 25251 (23413-27411) | 298.99 (279.18-325.60) | 0.54 (0.52 to 0.56) | < 0.001 |
| Slovakia | 8255 (7427-9098) | 140.95 (128.07-154.90) | 20773 (18945-22792) | 226.55 (207.13-244.61) | 1.54 (1.51 to 1.58) | < 0.001 |
| Slovenia | 3003 (2710-3343) | 125.99 (114.01-139.41) | 9213 (8322-10191) | 220.86 (200.94-243.54) | 1.85 (1.76 to 1.95) | < 0.001 |
| Solomon Islands | 347 (324-372) | 162.81 (150.76-175.88) | 825 (765-887) | 178.99 (165.17-193.51) | 0.30 (0.27 to 0.33) | < 0.001 |
| Somalia | 3438 (3200-3700) | 96.22 (88.58-104.36) | 10305 (9580-10987) | 113.03 (103.05-122.34) | 0.52 (0.49 to 0.56) | < 0.001 |
| South Africa | 40375 (37410-43475) | 172.86 (160.07-187.50) | 116840 (108463-124634) | 242.59 (225.07-257.95) | 1.09 (1.04 to 1.15) | < 0.001 |
| South Sudan | 3133 (2915-3386) | 97.83 (90.15-106.87) | 5863 (5451-6351) | 120.20 (110.54-131.24) | 0.68 (0.63 to 0.73) | < 0.001 |
| Spain | 132317 (121229-145627) | 237.83 (219.69-258.28) | 235484 (215974-254667) | 232.21 (211.91-250.03) | -0.04 (-0.09 to 0.01) | 0.099 |
| Sri Lanka | 21972 (20287-23967) | 186.57 (171.80-203.07) | 77127 (70184-84714) | 280.69 (257.94-305.20) | 1.36 (1.28 to 1.43) | < 0.001 |
| Sudan | 22886 (20725-25488) | 216.76 (195.29-241.94) | 72866 (66723-78876) | 334.15 (303.81-366.32) | 1.41 (1.28 to 1.55) | < 0.001 |
| Suriname | 540 (493-591) | 188.36 (171.76-206.62) | 1937 (1753-2093) | 303.00 (278.56-326.70) | 1.57 (1.52 to 1.62) | < 0.001 |
| Sweden | 28865 (26001-32782) | 180.28 (164.10-201.27) | 38458 (34200-43016) | 171.34 (155.23-188.60) | -0.16 (-0.17 to -0.14) | < 0.001 |
| Switzerland | 25404 (23197-28054) | 237.27 (218.25-259.52) | 47328 (42804-51366) | 256.74 (232.96-278.09) | 0.26 (0.23 to 0.30) | < 0.001 |
| Syrian Arab Republic | 16987 (15686-18588) | 269.10 (247.07-295.78) | 56594 (52294-61422) | 407.40 (380.27-438.93) | 1.36 (1.26 to 1.47) | < 0.001 |
| Taiwan (Province of China) | 41232 (38269-44342) | 253.71 (236.83-271.74) | 123945 (116112-131502) | 302.04 (284.60-319.00) | 0.57 (0.55 to 0.58) | < 0.001 |
| Tajikistan | 3032 (2708-3348) | 79.38 (70.82-88.37) | 9226 (8357-10093) | 127.73 (116.14-140.00) | 1.56 (1.52 to 1.59) | < 0.001 |
| Thailand | 83500 (76620-91125) | 209.97 (193.60-228.52) | 317728 (294103-344411) | 301.42 (281.43-325.41) | 1.20 (1.11 to 1.29) | < 0.001 |
| Timor-Leste | 589 (545-639) | 148.86 (136.71-164.16) | 1927 (1773-2103) | 202.45 (186.05-220.15) | 1.01 (0.97 to 1.05) | < 0.001 |
| Togo | 2470 (2302-2654) | 137.25 (126.56-148.73) | 8279 (7641-8954) | 176.54 (160.07-192.24) | 0.81 (0.76 to 0.86) | < 0.001 |
| Tokelau | 2 (2-2) | 156.46 (144.13-170.08) | 4 (3-4) | 248.64 (228.62-269.09) | 1.52 (1.47 to 1.57) | < 0.001 |
| Tonga | 120 (110-131) | 184.57 (169.88-203.59) | 226 (209-243) | 264.39 (244.83-284.90) | 1.17 (1.14 to 1.20) | < 0.001 |
| Trinidad and Tobago | 1663 (1540-1811) | 183.34 (168.93-200.97) | 5778 (5277-6286) | 308.46 (285.17-332.51) | 1.71 (1.63 to 1.79) | < 0.001 |
| Tunisia | 13411 (12220-14805) | 249.46 (228.06-273.32) | 54366 (49514-59189) | 402.84 (370.59-435.32) | 1.56 (1.49 to 1.63) | < 0.001 |
| Turkey | 80831 (75539-86876) | 223.53 (208.16-239.61) | 384231 (352192-414424) | 404.10 (371.72-432.15) | 1.91 (1.87 to 1.96) | < 0.001 |
| Turkmenistan | 3149 (2914-3384) | 115.59 (107.05-125.72) | 8401 (7732-9038) | 179.00 (165.84-191.12) | 1.42 (1.39 to 1.46) | < 0.001 |
| Tuvalu | 11 (10-12) | 149.43 (137.38-164.84) | 26 (24-29) | 239.74 (221.71-260.75) | 1.54 (1.51 to 1.57) | < 0.001 |
| Uganda | 7432 (6918-7994) | 86.93 (79.94-94.55) | 19849 (18513-21371) | 106.71 (97.77-116.52) | 0.65 (0.56 to 0.74) | < 0.001 |
| Ukraine | 52974 (47075-59414) | 81.45 (73.62-89.89) | 82042 (75038-90447) | 123.52 (113.87-134.46) | 1.34 (1.24 to 1.44) | < 0.001 |
| United Arab Emirates | 2135 (1941-2336) | 327.34 (300.54-358.32) | 31938 (28288-36332) | 466.42 (432.99-505.58) | 1.17 (1.01 to 1.32) | < 0.001 |
| United Kingdom | 195682 (178100-218323) | 209.00 (191.31-229.71) | 290434 (265595-316836) | 219.73 (201.61-237.17) | 0.15 (0.13 to 0.17) | < 0.001 |
| United Republic of Tanzania | 14481 (13439-15616) | 104.14 (95.20-113.18) | 38553 (35790-41463) | 128.12 (117.66-139.12) | 0.69 (0.64 to 0.74) | < 0.001 |
| United States of America | 965462 (882901-1056989) | 303.42 (278.01-331.01) | 1842212 (1695726-1979976) | 323.19 (300.14-346.30) | 0.21 (0.18 to 0.23) | < 0.001 |
| United States Virgin Islands | 186 (171-202) | 201.39 (186.04-217.73) | 512 (467-565) | 315.13 (291.58-344.04) | 1.48 (1.42 to 1.54) | < 0.001 |
| Uruguay | 7727 (7006-8507) | 197.08 (179.76-215.55) | 13240 (12080-14386) | 238.26 (219.31-257.28) | 0.62 (0.60 to 0.64) | < 0.001 |
| Uzbekistan | 21869 (20280-23618) | 143.91 (133.02-157.29) | 62513 (56559-68430) | 205.69 (188.05-223.65) | 1.17 (1.11 to 1.23) | < 0.001 |
| Vanuatu | 124 (114-132) | 144.67 (133.48-157.38) | 447 (413-485) | 215.16 (198.25-231.45) | 1.28 (1.21 to 1.35) | < 0.001 |
| Venezuela (Bolivarian Republic of) | 27615 (25358-30284) | 246.23 (225.06-272.61) | 113852 (105689-122187) | 371.16 (346.13-396.46) | 1.33 (1.28 to 1.37) | < 0.001 |
| Viet Nam | 61003 (55831-66358) | 136.89 (125.14-149.23) | 215387 (195155-237672) | 214.70 (196.62-234.13) | 1.49 (1.46 to 1.51) | < 0.001 |
| Yemen | 12658 (11432-13948) | 212.97 (191.63-236.63) | 48349 (44356-52264) | 303.73 (277.20-330.97) | 1.15 (1.07 to 1.23) | < 0.001 |
| Zambia | 4313 (4015-4642) | 108.48 (100.19-118.21) | 12225 (11417-13120) | 137.13 (126.39-148.92) | 0.80 (0.74 to 0.86) | < 0.001 |
| Zimbabwe | 7854 (7224-8575) | 158.79 (145.39-175.57) | 15456 (14249-16766) | 189.54 (174.01-205.68) | 0.57 (0.50 to 0.64) | < 0.001 |

**Table S13.** The number and age-standardized rate of prevalence for chronic kidney disease in 1990 and 2021, and the temporal trends between 1990-2021 in 204 countries and territories.

| Location | Number of cases, 1990 | Age-standardized rate per 100,000 population, 1990 | Number of cases, 2021 | Age-standardized rate per 100,000 population, 2021 | Average annual percentage change, 1990-2021 | p value |
| --- | --- | --- | --- | --- | --- | --- |
| Afghanistan | 611982 (569081-656341) | 8808.89 (8175.35-9452.34) | 1440765 (1317910-1571513) | 8934.90 (8262.77-9627.27) | 0.05 (0.04 to 0.05) | < 0.001 |
| Albania | 152351 (142462-163786) | 6243.74 (5830.90-6667.44) | 230609 (214471-246180) | 6153.95 (5711.78-6544.94) | -0.04 (-0.05 to -0.04) | < 0.001 |
| Algeria | 1413887 (1302734-1527151) | 8945.97 (8267.19-9615.92) | 3553584 (3275804-3852065) | 8807.19 (8150.01-9515.20) | -0.05 (-0.06 to -0.04) | < 0.001 |
| American Samoa | 2729 (2519-2958) | 8831.31 (8197.44-9501.62) | 4468 (4153-4802) | 9256.65 (8599.04-9943.02) | 0.15 (0.14 to 0.17) | < 0.001 |
| Andorra | 3305 (3059-3549) | 5604.92 (5216.11-5996.52) | 7623 (7073-8129) | 5534.99 (5147.91-5919.35) | -0.04 (-0.05 to -0.02) | < 0.001 |
| Angola | 473841 (440001-510739) | 9090.82 (8510.84-9718.36) | 1482282 (1374318-1605870) | 9018.88 (8383.16-9636.04) | -0.03 (-0.03 to -0.02) | < 0.001 |
| Antigua and Barbuda | 3729 (3474-3994) | 6700.12 (6253.50-7179.10) | 7270 (6762-7815) | 6956.33 (6479.95-7455.65) | 0.12 (0.11 to 0.13) | < 0.001 |
| Argentina | 1774583 (1648138-1900776) | 5585.84 (5197.53-5980.00) | 3114327 (2893145-3345866) | 5876.08 (5442.40-6323.90) | 0.16 (0.15 to 0.18) | < 0.001 |
| Armenia | 300635 (278865-323273) | 10265.94 (9580.75-10973.98) | 404070 (378068-430935) | 10245.02 (9598.01-10928.04) | -0.01 (-0.01 to 0.00) | < 0.001 |
| Australia | 1165447 (1094777-1238029) | 6076.77 (5714.75-6452.52) | 2353593 (2211156-2523412) | 5877.91 (5525.88-6272.06) | -0.12 (-0.17 to -0.07) | < 0.001 |
| Austria | 611202 (567555-653621) | 5590.87 (5205.69-5978.51) | 892225 (838208-953902) | 5671.05 (5326.44-6084.53) | 0.06 (0.04 to 0.07) | < 0.001 |
| Azerbaijan | 593465 (554684-636804) | 10594.84 (9927.52-11288.36) | 1146469 (1073053-1221894) | 10695.96 (10018.97-11338.19) | 0.03 (0.01 to 0.05) | 0.002 |
| Bahamas | 12715 (11806-13734) | 6542.75 (6098.88-7002.89) | 28177 (26096-30221) | 6759.28 (6258.56-7239.09) | 0.11 (0.10 to 0.11) | < 0.001 |
| Bahrain | 31525 (28728-34958) | 9152.15 (8487.52-9866.93) | 130181 (119335-142831) | 9108.49 (8445.16-9765.84) | -0.02 (-0.03 to -0.01) | < 0.001 |
| Bangladesh | 5331909 (4950686-5772489) | 8395.62 (7832.81-8968.06) | 12882108 (11949483-13899210) | 8555.39 (7963.38-9177.27) | 0.07 (0.04 to 0.09) | < 0.001 |
| Barbados | 17997 (16818-19169) | 6459.85 (6051.67-6909.15) | 28988 (27004-31010) | 6676.76 (6215.33-7161.73) | 0.11 (0.10 to 0.11) | < 0.001 |
| Belarus | 1162192 (1079543-1248012) | 9654.09 (8993.23-10364.91) | 1314740 (1220742-1405904) | 9704.35 (9000.43-10430.51) | 0.02 (0.01 to 0.03) | 0.001 |
| Belgium | 808609 (750019-867640) | 5722.14 (5342.51-6109.53) | 1120459 (1051828-1196504) | 5633.19 (5305.47-6009.37) | -0.04 (-0.06 to -0.03) | < 0.001 |
| Belize | 7609 (7071-8133) | 6629.92 (6188.80-7130.86) | 24923 (23248-26780) | 6996.94 (6519.92-7476.11) | 0.17 (0.16 to 0.18) | < 0.001 |
| Benin | 188015 (174766-201634) | 7343.41 (6857.48-7853.45) | 552484 (512421-599062) | 7482.30 (7003.24-8021.89) | 0.06 (0.05 to 0.07) | < 0.001 |
| Bermuda | 4041 (3755-4358) | 6209.73 (5792.54-6656.89) | 6571 (6087-7048) | 6114.22 (5679.31-6583.54) | -0.05 (-0.06 to -0.04) | < 0.001 |
| Bhutan | 31715 (29384-34240) | 9135.27 (8522.13-9749.86) | 64186 (59913-69314) | 9213.54 (8612.13-9876.63) | 0.03 (0.02 to 0.04) | < 0.001 |
| Bolivia (Plurinational State of) | 242900 (225810-260389) | 6093.33 (5681.07-6496.89) | 628320 (584306-676494) | 6177.14 (5740.24-6632.37) | 0.04 (0.03 to 0.05) | < 0.001 |
| Bosnia and Herzegovina | 260939 (242972-279575) | 6132.37 (5735.30-6544.34) | 323305 (300335-346499) | 6233.46 (5827.17-6665.08) | 0.05 (0.04 to 0.06) | < 0.001 |
| Botswana | 60835 (56449-65830) | 8684.67 (8075.88-9285.61) | 164894 (153184-177219) | 8867.29 (8244.14-9441.16) | 0.09 (0.06 to 0.12) | < 0.001 |
| Brazil | 8215420 (7640129-8812335) | 7772.73 (7248.74-8291.21) | 18814692 (17566365-20174535) | 7576.55 (7078.47-8111.19) | -0.08 (-0.10 to -0.06) | < 0.001 |
| Brunei Darussalam | 15220 (13998-16528) | 9497.75 (8852.21-10130.49) | 39451 (36520-42780) | 9461.95 (8796.56-10168.95) | -0.01 (-0.02 to 0.00) | 0.006 |
| Bulgaria | 673785 (626288-720194) | 6187.85 (5772.96-6611.13) | 726001 (674875-778060) | 6282.06 (5866.87-6704.40) | 0.05 (0.03 to 0.07) | < 0.001 |
| Burkina Faso | 365246 (337985-392297) | 7076.19 (6568.58-7570.49) | 901049 (834507-973982) | 7181.79 (6699.93-7686.70) | 0.05 (0.04 to 0.05) | < 0.001 |
| Burundi | 187278 (170970-204259) | 5868.15 (5412.10-6352.77) | 456223 (414259-502680) | 5921.59 (5472.49-6436.62) | 0.03 (0.02 to 0.03) | < 0.001 |
| Cabo Verde | 17443 (16294-18677) | 7283.33 (6802.80-7767.94) | 38020 (35307-41075) | 7366.66 (6831.08-7880.32) | 0.04 (0.03 to 0.06) | < 0.001 |
| Cambodia | 566875 (518696-615039) | 9455.04 (8749.38-10222.83) | 1378217 (1266270-1503301) | 9452.98 (8751.36-10231.34) | 0.00 (0.00 to 0.01) | 0.584 |
| Cameroon | 463067 (431742-496225) | 8270.67 (7742.07-8827.31) | 1594660 (1486358-1713045) | 9043.60 (8503.39-9609.76) | 0.29 (0.26 to 0.32) | < 0.001 |
| Canada | 2229477 (2055472-2415616) | 7026.44 (6480.64-7603.72) | 3809329 (3540568-4060952) | 6524.37 (6091.51-7000.69) | -0.24 (-0.27 to -0.21) | < 0.001 |
| Central African Republic | 133406 (123251-143340) | 9207.41 (8552.54-9860.33) | 276101 (255353-297770) | 9141.25 (8513.25-9769.20) | -0.03 (-0.03 to -0.02) | < 0.001 |
| Chad | 241571 (223146-258353) | 7195.07 (6688.44-7683.20) | 603512 (558192-650782) | 7271.05 (6764.68-7787.57) | 0.04 (0.03 to 0.04) | < 0.001 |
| Chile | 699136 (650457-755320) | 6210.96 (5786.85-6653.92) | 1444161 (1343232-1545985) | 6106.55 (5659.58-6545.88) | -0.06 (-0.08 to -0.03) | < 0.001 |
| China | 67398525 (62608781-72614585) | 7091.90 (6614.31-7603.09) | 118403911 (109388671-127484286) | 6249.41 (5812.36-6720.21) | -0.40 (-0.47 to -0.32) | < 0.001 |
| Colombia | 1697055 (1583361-1826064) | 7912.44 (7376.24-8461.75) | 4222423 (3939788-4478779) | 7695.61 (7194.03-8158.77) | -0.09 (-0.10 to -0.08) | < 0.001 |
| Comoros | 15628 (14361-16936) | 5870.66 (5455.67-6305.40) | 35339 (32389-38509) | 5851.82 (5407.43-6323.60) | -0.01 (-0.02 to 0.00) | 0.133 |
| Congo | 122987 (114132-132085) | 9370.06 (8763.10-9997.99) | 331895 (307907-357776) | 9319.63 (8696.92-9931.83) | -0.02 (-0.03 to 0.00) | 0.025 |
| Cook Islands | 1150 (1062-1242) | 8019.63 (7447.92-8644.54) | 1841 (1712-1970) | 8230.01 (7649.94-8830.44) | 0.08 (0.07 to 0.09) | < 0.001 |
| Costa Rica | 197345 (183956-211905) | 9636.74 (8996.25-10298.8) | 517788 (484174-550601) | 9532.08 (8909.84-10127.10) | 0.06 (0.04 to 0.09) | < 0.001 |
| Coted'Ivoire | 462576 (427133-500797) | 7562.00 (7049.37-8106.42) | 1249475 (1157292-1360894) | 7597.71 (7075.19-8139.17) | 0.02 (0.02 to 0.02) | < 0.001 |
| Croatia | 352794 (326765-378976) | 6188.88 (5755.54-6628.99) | 441010 (407740-471831) | 6109.13 (5670.01-6576.33) | -0.04 (-0.04 to -0.04) | < 0.001 |
| Cuba | 670575 (622916-717810) | 6258.54 (5824.65-6706.15) | 1023168 (949443-1092062) | 6157.34 (5728.33-6614.34) | -0.05 (-0.06 to -0.05) | < 0.001 |
| Cyprus | 47988 (44580-51332) | 6008.97 (5608.31-6395.05) | 112031 (104079-120582) | 5817.59 (5430.82-6247.88) | -0.10 (-0.11 to -0.10) | < 0.001 |
| Czechia | 777458 (723745-829773) | 6159.60 (5756.88-6580.72) | 1033665 (960274-1099534) | 5875.02 (5479.31-6268.38) | -0.15 (-0.16 to -0.15) | < 0.001 |
| Democratic People's Republic of Korea | 1150078 (1067243-1233674) | 6571.77 (6117.41-7043.46) | 2027239 (1883443-2173423) | 6390.78 (5955.90-6816.79) | -0.10 (-0.11 to -0.08) | < 0.001 |
| Democratic Republic of the Congo | 1824071 (1691866-1955647) | 9255.33 (8613.46-9831.06) | 4529099 (4238150-4861233) | 9196.85 (8707.06-9726.56) | -0.02 (-0.03 to -0.01) | < 0.001 |
| Denmark | 415234 (386838-442513) | 5589.55 (5229.32-5953.90) | 564571 (524056-607172) | 5607.02 (5237.41-6009.77) | 0.01 (0.01 to 0.02) | < 0.001 |
| Djibouti | 13012 (11876-14272) | 5545.12 (5142.12-5961.77) | 55546 (50733-60942) | 5776.89 (5342.94-6232.52) | 0.13 (0.12 to 0.14) | < 0.001 |
| Dominica | 4263 (3961-4572) | 6991.41 (6509.20-7479.94) | 5745 (5329-6134) | 7303.91 (6813.01-7818.62) | 0.14 (0.13 to 0.15) | < 0.001 |
| Dominican Republic | 299732 (277339-324408) | 6357.90 (5927.63-6822.57) | 710332 (660993-766986) | 6688.81 (6211.08-7193.25) | 0.16 (0.15 to 0.17) | < 0.001 |
| Ecuador | 392453 (366304-421830) | 5893.37 (5491.99-6356.84) | 1073132 (996177-1149397) | 6249.30 (5811.70-6689.18) | 0.19 (0.18 to 0.20) | < 0.001 |
| Egypt | 3069526 (2834187-3319651) | 8613.59 (8031.71-9206.03) | 7320032 (6762507-7887829) | 9167.78 (8515.01-9825.36) | 0.20 (0.19 to 0.21) | < 0.001 |
| El Salvador | 239034 (222018-257099) | 6892.21 (6378.22-7415.71) | 460009 (431238-489587) | 7296.14 (6841.63-7770.96) | 0.19 (0.16 to 0.21) | < 0.001 |
| Equatorial Guinea | 20935 (19447-22604) | 9087.34 (8498.49-9734.45) | 75546 (69523-82103) | 9342.30 (8719.87-9945.44) | 0.09 (0.08 to 0.09) | < 0.001 |
| Eritrea | 99470 (90769-109284) | 5529.86 (5117.87-5982.06) | 244600 (224167-268056) | 5676.57 (5255.07-6117.57) | 0.09 (0.06 to 0.11) | < 0.001 |
| Estonia | 181645 (168130-196037) | 9678.72 (8968.05-10435.79) | 206581 (192387-221597) | 9660.85 (9006.34-10389.66) | 0.00 (-0.02 to 0.01) | 0.419 |
| Eswatini | 33811 (31458-36377) | 8783.16 (8183.64-9417.55) | 66871 (61736-72023) | 9047.60 (8414.14-9624.29) | 0.13 (0.11 to 0.15) | < 0.001 |
| Ethiopia | 1542523 (1422919-1673239) | 5569.28 (5168.79-5985.12) | 3762262 (3443544-4113603) | 5649.28 (5230.25-6098.21) | 0.05 (0.04 to 0.06) | < 0.001 |
| Fiji | 43084 (39626-46792) | 8584.29 (7980.69-9231.03) | 74318 (68583-80409) | 8913.76 (8286.56-9600.93) | 0.12 (0.11 to 0.13) | < 0.001 |
| Finland | 368157 (345612-392962) | 5556.65 (5209.26-5940.57) | 555910 (513129-595333) | 5349.21 (4968.62-5714.85) | -0.12 (-0.13 to -0.10) | < 0.001 |
| France | 3209806 (2980224-3426824) | 4202.18 (3898.73-4488.25) | 5214795 (4858423-5614715) | 4368.82 (4085.59-4698.68) | 0.13 (0.11 to 0.14) | < 0.001 |
| Gabon | 57987 (53943-62001) | 9126.03 (8487.06-9779.76) | 117028 (108707-125772) | 9237.99 (8645.40-9881.02) | 0.04 (0.03 to 0.04) | < 0.001 |
| Gambia | 36537 (33712-39478) | 7285.83 (6764.38-7776.61) | 103566 (95771-112154) | 7469.17 (6954.59-8002.94) | 0.09 (0.08 to 0.10) | < 0.001 |
| Georgia | 632971 (590879-679007) | 10617.53 (9919.39-11342.70) | 544204 (506648-579897) | 10551.11 (9851.30-11224.00) | -0.02 (-0.03 to -0.02) | < 0.001 |
| Germany | 6261066 (5866849-6644060) | 5349.62 (5017.36-5674.99) | 8931944 (8467049-9441979) | 5278.41 (4985.46-5622.16) | -0.04 (-0.07 to -0.01) | 0.014 |
| Ghana | 516871 (472923-562875) | 5943.09 (5509.33-6423.82) | 1422793 (1305227-1553890) | 6033.80 (5584.86-6513.19) | 0.05 (0.04 to 0.06) | < 0.001 |
| Greece | 798820 (740666-853941) | 5762.34 (5361.41-6162.89) | 1143247 (1066167-1220356) | 5587.78 (5236.26-5992.78) | -0.09 (-0.11 to -0.07) | < 0.001 |
| Greenland | 2958 (2729-3212) | 6970.68 (6515.60-7414.55) | 4412 (4094-4732) | 6988.24 (6509.14-7491.40) | 0.01 (0.00 to 0.01) | 0.186 |
| Grenada | 5042 (4684-5387) | 6983.13 (6481.81-7464.02) | 8182 (7614-8784) | 7279.46 (6800.35-7818.68) | 0.13 (0.13 to 0.14) | < 0.001 |
| Guam | 8188 (7532-8928) | 7935.12 (7378.36-8544.49) | 16160 (14994-17291) | 8480.20 (7890.47-9105.98) | 0.21 (0.20 to 0.23) | < 0.001 |
| Guatemala | 342544 (318619-365583) | 8065.47 (7574.08-8638.44) | 1096863 (1023152-1176256) | 8857.74 (8275.10-9478.35) | 0.30 (0.29 to 0.32) | < 0.001 |
| Guinea | 275726 (256995-296333) | 7313.88 (6820.17-7803.84) | 568301 (528813-612479) | 7498.27 (6998.79-7995.51) | 0.09 (0.08 to 0.10) | < 0.001 |
| Guinea-Bissau | 40061 (37305-43253) | 7641.76 (7095.74-8167.34) | 85214 (78709-92411) | 7643.78 (7104.09-8145.37) | 0.01 (0.00 to 0.02) | 0.188 |
| Guyana | 34192 (31637-36857) | 6803.89 (6325.87-7280.49) | 48899 (45521-52822) | 7062.48 (6578.99-7608.24) | 0.12 (0.11 to 0.13) | < 0.001 |
| Haiti | 274957 (254724-296317) | 6959.23 (6473.01-7441.57) | 662956 (613153-717829) | 7123.74 (6614.82-7647.93) | 0.08 (0.07 to 0.08) | < 0.001 |
| Honduras | 209413 (194859-225041) | 8405.32 (7844.10-8968.83) | 633449 (591340-678996) | 8612.98 (8025.92-9216.03) | 0.08 (0.07 to 0.09) | < 0.001 |
| Hungary | 816867 (760112-874417) | 6193.16 (5788.18-6630.36) | 965275 (898080-1028450) | 5992.86 (5610.73-6393.59) | -0.10 (-0.12 to -0.09) | < 0.001 |
| Iceland | 13378 (12516-14287) | 4739.04 (4420.17-5063.17) | 23128 (21625-24673) | 4528.93 (4231.01-4840.19) | -0.14 (-0.18 to -0.10) | < 0.001 |
| India | 60111283 (55460535-65038616) | 10258.75 (9547.88-10986.05) | 128031911 (118513994-138721278) | 9710.51 (9024.24-10420.26) | -0.15 (-0.34 to 0.05) | 0.14 |
| Indonesia | 13599739 (12496422-14810294) | 10447.73 (9661.88-11264.48) | 29413896 (26931317-32010165) | 10740.30 (9942.08-11598.81) | 0.10 (0.09 to 0.12) | < 0.001 |
| Iran (Islamic Republic of) | 3141831 (2920460-3371296) | 9513.67 (8874.05-10180.40) | 8145077 (7562399-8737333) | 9474.79 (8837.14-10118.39) | -0.01 (-0.02 to 0.00) | 0.077 |
| Iraq | 1026557 (949992-1108210) | 9536.93 (8851.49-10239.34) | 2977727 (2740400-3234895) | 9464.18 (8764.44-10188.22) | -0.03 (-0.03 to -0.02) | < 0.001 |
| Ireland | 261985 (241344-282388) | 6681.69 (6202.54-7184.27) | 465353 (440528-488132) | 6594.14 (6237.54-6952.01) | -0.03 (-0.05 to -0.02) | < 0.001 |
| Israel | 288923 (270285-308809) | 6007.76 (5618.93-6420.26) | 674873 (630913-722204) | 5866.76 (5487.92-6273.74) | -0.07 (-0.08 to -0.06) | < 0.001 |
| Italy | 4457622 (4142484-4769497) | 5591.86 (5235.46-5978.78) | 5921997 (5501809-6294811) | 5061.28 (4746.22-5410.53) | -0.31 (-0.35 to -0.27) | < 0.001 |
| Jamaica | 122884 (115047-131333) | 6456.88 (6045.33-6921.34) | 209554 (194578-224704) | 6708.14 (6219.00-7188.82) | 0.12 (0.12 to 0.13) | < 0.001 |
| Japan | 13889911 (12903717-14885294) | 8744.79 (8150.41-9364.15) | 22877631 (21341971-24294034) | 8264.30 (7717.71-8855.73) | -0.18 (-0.23 to -0.14) | < 0.001 |
| Jordan | 187654 (172497-203471) | 9249.53 (8606.77-9945.62) | 889238 (817131-968041) | 8989.67 (8318.59-9729.30) | -0.09 (-0.11 to -0.08) | < 0.001 |
| Kazakhstan | 1471284 (1368347-1573180) | 10730.43 (10056.28-11411.96) | 1952569 (1812225-2090896) | 10662.37 (9928.10-11394.35) | -0.02 (-0.03 to -0.02) | < 0.001 |
| Kenya | 700742 (642264-764896) | 5761.73 (5343.22-6221.90) | 1979251 (1807371-2167364) | 5900.75 (5470.00-6368.96) | 0.08 (0.07 to 0.08) | < 0.001 |
| Kiribati | 4029 (3716-4354) | 8459.58 (7856.76-9093.47) | 7836 (7239-8460) | 8718.24 (8109.87-9329.42) | 0.10 (0.09 to 0.11) | < 0.001 |
| Kuwait | 106214 (96298-117759) | 8982.78 (8321.07-9651.59) | 393166 (356902-436552) | 8593.08 (7931.84-9269.39) | -0.15 (-0.15 to -0.14) | < 0.001 |
| Kyrgyzstan | 349912 (326466-372023) | 10650.21 (9986.27-11306.14) | 554675 (518240-592588) | 10147.73 (9422.6-10780.31) | -0.16 (-0.17 to -0.15) | < 0.001 |
| Lao People's Democratic Republic | 277122 (254762-299243) | 10702 (9904.76-11504.62) | 621716 (570484-678042) | 10478.69 (9688.02-11292.97) | -0.07 (-0.07 to -0.06) | < 0.001 |
| Latvia | 313358 (290679-336853) | 9660.91 (8967.62-10391.33) | 303626 (282328-323377) | 9785.76 (9110.46-10514.29) | 0.04 (0.04 to 0.05) | < 0.001 |
| Lebanon | 211783 (195737-228352) | 8827.59 (8183.84-9494.60) | 548008 (508010-589947) | 8988.21 (8309.11-9607.83) | 0.06 (0.04 to 0.08) | < 0.001 |
| Lesotho | 79823 (74328-85461) | 8435.22 (7898.67-8992.70) | 114258 (105784-123018) | 8824.57 (8201.21-9408.83) | 0.18 (0.14 to 0.21) | < 0.001 |
| Liberia | 107783 (99699-116096) | 7548.18 (7002.10-8076.21) | 244755 (226016-265719) | 7551.19 (7049.82-8085.22) | 0.00 (-0.01 to 0.01) | 0.483 |
| Libya | 228746 (211473-246316) | 9024.45 (8359.99-9727.35) | 610601 (560616-667943) | 9269.11 (8567.50-10032.22) | 0.09 (0.08 to 0.09) | < 0.001 |
| Lithuania | 414295 (384463-446122) | 9790.23 (9086.72-10563.46) | 441211 (411206-472545) | 9837.2 (9134.92-10590.03) | 0.01 (0.01 to 0.02) | < 0.001 |
| Luxembourg | 30243 (27852-32537) | 5937.62 (5491.41-6400.12) | 55107 (50921-59381) | 5775.64 (5340.05-6220.77) | -0.09 (-0.10 to -0.08) | < 0.001 |
| Madagascar | 389060 (359027-423079) | 5625.82 (5196.63-6061.50) | 994473 (908917-1089154) | 5685.27 (5246.18-6164.05) | 0.03 (0.03 to 0.04) | < 0.001 |
| Malawi | 326018 (298440-355273) | 5918.53 (5492.05-6409.40) | 705439 (644778-770229) | 6124.79 (5658.19-6619.29) | 0.11 (0.10 to 0.12) | < 0.001 |
| Malaysia | 1328704 (1222289-1442379) | 10828.26 (10076.24-11666.58) | 3443338 (3171290-3729769) | 11008.65 (10171.17-11849.41) | 0.05 (0.02 to 0.09) | 0.001 |
| Maldives | 12689 (11699-13709) | 10424.93 (9701.37-11220.84) | 52048 (47116-57976) | 10202.65 (9431.96-11087.04) | -0.06 (-0.07 to -0.05) | < 0.001 |
| Mali | 358053 (333871-381898) | 7349.84 (6875.48-7816.73) | 909702 (841914-979718) | 7419.92 (6929.48-7932.56) | 0.03 (0.03 to 0.04) | < 0.001 |
| Malta | 24308 (22689-26237) | 5881.70 (5499.49-6324.87) | 46498 (43037-49500) | 5665.77 (5292.24-6053.13) | -0.12 (-0.13 to -0.10) | < 0.001 |
| Marshall Islands | 1905 (1760-2065) | 8175.25 (7594.65-8766.64) | 3728 (3432-4030) | 8476.00 (7879.65-9041.98) | 0.12 (0.11 to 0.12) | < 0.001 |
| Mauritania | 91156 (84420-98252) | 7479.43 (6957.67-8019.85) | 197970 (183860-213616) | 7285.48 (6794.61-7783.79) | -0.08 (-0.09 to -0.08) | < 0.001 |
| Mauritius | 96542 (88907-105229) | 10712.2 (9939.06-11620.82) | 189008 (175935-203155) | 11411.55 (10649.12-12263.72) | 0.21 (0.18 to 0.24) | < 0.001 |
| Mexico | 4594717 (4287702-4924515) | 8781.48 (8206.61-9393.83) | 11780174 (11009027-12569139) | 9052.16 (8453.85-9672.71) | 0.10 (0.08 to 0.11) | < 0.001 |
| Micronesia (Federated States of) | 5110 (4740-5505) | 8313.35 (7738.62-8898.94) | 7394 (6849-8003) | 8744.07 (8145.41-9371.47) | 0.16 (0.14 to 0.18) | < 0.001 |
| Monaco | 3295 (3048-3530) | 5539.92 (5150.24-5916.29) | 4350 (4042-4640) | 5497.16 (5141.16-5862.88) | -0.02 (-0.03 to -0.01) | < 0.001 |
| Mongolia | 146040 (136801-156132) | 11239.6 (10569.26-11958.83) | 287978 (267255-309250) | 10667.74 (9937.15-11381.17) | -0.17 (-0.18 to -0.16) | < 0.001 |
| Montenegro | 40300 (37650-42973) | 6416.04 (6010.81-6835.49) | 54152 (50266-57833) | 6414.96 (5962.07-6863.77) | 0.00 (-0.01 to 0.01) | 0.827 |
| Morocco | 1521333 (1399644-1649317) | 8496.57 (7863.97-9155.09) | 3125307 (2880319-3372664) | 8498.36 (7864.02-9135.02) | 0.00 (-0.01 to 0.01) | 0.857 |
| Mozambique | 471644 (433799-513878) | 5918.70 (5479.55-6395.09) | 1066862 (978804-1170332) | 6162.57 (5705.70-6708.87) | 0.13 (0.12 to 0.14) | < 0.001 |
| Myanmar | 2982783 (2758045-3235228) | 10357.18 (9596.64-11185.41) | 5373282 (4929525-5841826) | 10172.97 (9393.11-11017.34) | -0.05 (-0.07 to -0.04) | < 0.001 |
| Namibia | 68175 (63117-73308) | 8664.38 (8056.49-9233.53) | 144692 (133912-155904) | 8510.12 (7908.45-9112.39) | -0.04 (-0.07 to 0.00) | 0.027 |
| Nauru | 544 (502-592) | 8632.12 (8048.05-9264.68) | 698 (644-757) | 9104.97 (8473.18-9748.89) | 0.17 (0.17 to 0.18) | < 0.001 |
| Nepal | 1177468 (1101596-1257964) | 10501.16 (9843.11-11204.30) | 2762106 (2575557-3032129) | 10887.67 (10177.44-11883.1) | 0.12 (0.07 to 0.17) | < 0.001 |
| Netherlands | 1147619 (1073580-1226033) | 6067.39 (5675.85-6498.24) | 1745668 (1634215-1877107) | 6048.17 (5674.28-6481.09) | -0.01 (-0.06 to 0.04) | 0.649 |
| New Zealand | 234292 (217327-250752) | 6128.41 (5708.20-6546.64) | 457184 (423466-488353) | 6072.56 (5649.20-6499.05) | -0.03 (-0.04 to -0.01) | 0.001 |
| Nicaragua | 190029 (177519-203548) | 9675.18 (9075.43-10313.24) | 575347 (537918-614144) | 10511.16 (9794.29-11157.83) | 0.27 (0.25 to 0.28) | < 0.001 |
| Niger | 265038 (246140-284905) | 6880.22 (6411.46-7331.36) | 790430 (731816-852136) | 6881.82 (6387.81-7357.14) | 0.00 (0.00 to 0.01) | 0.143 |
| Nigeria | 4951044 (4609650-5305923) | 9402.32 (8798.45-10034.70) | 11730967 (10926433-12598672) | 9488.90 (8881.14-10113.28) | 0.03 (0.02 to 0.05) | < 0.001 |
| Niue | 186 (173-199) | 8670.53 (8041.87-9333.97) | 175 (162-188) | 8957.48 (8282.57-9662.99) | 0.10 (0.10 to 0.11) | < 0.001 |
| North Macedonia | 140331 (130960-149494) | 7386.01 (6902.04-7876.80) | 220103 (205048-234493) | 7377.40 (6929.87-7832.19) | -0.01 (-0.03 to 0.01) | 0.518 |
| Northern Mariana Islands | 2867 (2626-3141) | 8797.60 (8200.85-9441.36) | 4746 (4387-5099) | 9078.76 (8455.65-9714.06) | 0.10 (0.09 to 0.12) | < 0.001 |
| Norway | 314586 (291897-335963) | 5207.12 (4866.27-5562.04) | 469377 (437256-500382) | 5534.40 (5167.84-5922.49) | 0.19 (0.16 to 0.21) | < 0.001 |
| Oman | 98358 (89680-108123) | 8427.69 (7838.18-9068.71) | 344284 (313617-379720) | 9024.46 (8385.68-9702.41) | 0.22 (0.21 to 0.23) | < 0.001 |
| Pakistan | 5931806 (5491196-6396491) | 8589.09 (7994.62-9202.42) | 15063043 (13945283-16289223) | 9164.60 (8522.84-9774.19) | 0.20 (0.18 to 0.22) | < 0.001 |
| Palau | 1027 (956-1114) | 8544.17 (7955.58-9159.80) | 1947 (1797-2108) | 8900.40 (8266.84-9595.25) | 0.13 (0.13 to 0.14) | < 0.001 |
| Palestine | 105072 (97202-113180) | 9224.59 (8569.81-9850.23) | 316240 (292025-342754) | 8942.60 (8314.54-9603.52) | -0.10 (-0.11 to -0.09) | < 0.001 |
| Panama | 142393 (132809-153036) | 8261.64 (7709.25-8864.77) | 373196 (347346-398963) | 8453.52 (7857.18-9031.74) | 0.07 (0.07 to 0.08) | < 0.001 |
| Papua New Guinea | 179603 (165528-195405) | 7388.60 (6896.15-7935.26) | 538462 (494564-590048) | 7685.65 (7155.00-8282.92) | 0.13 (0.12 to 0.14) | < 0.001 |
| Paraguay | 192742 (179980-206867) | 7374.35 (6876.73-7891.93) | 481010 (448476-516329) | 7561.39 (7051.56-8091.32) | 0.08 (0.08 to 0.09) | < 0.001 |
| Peru | 830105 (773577-894482) | 5621.62 (5237.55-6016.02) | 2042137 (1897519-2195297) | 5732.73 (5325.10-6154.29) | 0.06 (0.06 to 0.07) | < 0.001 |
| Philippines | 4286723 (3941958-4660864) | 10549.81 (9776.74-11356.13) | 10193697 (9395294-11071571) | 10530.35 (9775.30-11372.86) | 0.00 (-0.01 to 0.01) | 0.577 |
| Poland | 2866015 (2680925-3059816) | 6850.93 (6418.54-7310.93) | 3872502 (3620089-4134821) | 6415.29 (6012.65-6855.34) | -0.21 (-0.24 to -0.19) | < 0.001 |
| Portugal | 641583 (596339-685707) | 5124.52 (4804.74-5501.10) | 941606 (850524-1011350) | 4777.02 (4420.43-5132.33) | -0.23 (-0.24 to -0.21) | < 0.001 |
| Puerto Rico | 241664 (224964-259466) | 6681.74 (6222.45-7166.22) | 380144 (353031-405700) | 6833.02 (6356.42-7333.30) | 0.07 (0.06 to 0.08) | < 0.001 |
| Qatar | 28926 (26054-32385) | 9257.25 (8540.34-9961.49) | 238389 (214245-267350) | 9111.21 (8447.96-9852.64) | -0.05 (-0.07 to -0.02) | < 0.001 |
| Republic of Korea | 2703535 (2505415-2935211) | 7789.36 (7279.97-8381.08) | 5620395 (5254764-6008210) | 6872.19 (6416.99-7364.36) | -0.39 (-0.42 to -0.36) | < 0.001 |
| Republic of Moldova | 469578 (435381-501649) | 10729.28 (10014.30-11456.67) | 596118 (553806-636138) | 11293.66 (10519.57-12048.12) | 0.17 (0.12 to 0.22) | < 0.001 |
| Romania | 1605525 (1495525-1715394) | 6180.42 (5775.44-6591.06) | 1993675 (1890808-2098153) | 6300.66 (5964.64-6652.48) | 0.06 (-0.01 to 0.12) | 0.116 |
| Russian Federation | 15387961 (14274967-16655381) | 9062.21 (8429.24-9771.39) | 18056081 (16744178-19455656) | 8973.81 (8345.48-9704.77) | -0.03 (-0.03 to -0.03) | < 0.001 |
| Rwanda | 234499 (214398-255853) | 5852.58 (5422.12-6320.00) | 513932 (471486-561218) | 5696.48 (5277.18-6164.15) | -0.09 (-0.09 to -0.08) | < 0.001 |
| Saint Kitts and Nevis | 2582 (2409-2774) | 7034.18 (6570.19-7551.83) | 4826 (4470-5192) | 6962.38 (6470.03-7455.74) | -0.04 (-0.05 to -0.02) | < 0.001 |
| Saint Lucia | 6820 (6312-7305) | 6901.64 (6410.08-7371.53) | 15679 (14634-16864) | 6954.06 (6489.84-7468.21) | 0.02 (0.01 to 0.03) | < 0.001 |
| Saint Vincent and the Grenadines | 5218 (4816-5601) | 6544.36 (6043.47-7001.28) | 9229 (8590-9910) | 6902.60 (6435.84-7413.47) | 0.17 (0.16 to 0.18) | < 0.001 |
| Samoa | 8628 (8012-9299) | 8335.31 (7759.99-8940.84) | 13962 (12935-15079) | 8626.94 (8017.80-9290.73) | 0.11 (0.10 to 0.12) | < 0.001 |
| San Marino | 1771 (1651-1899) | 5453.49 (5097.14-5832.29) | 3397 (3165-3648) | 5392.57 (5034.83-5768.75) | -0.04 (-0.04 to -0.03) | < 0.001 |
| Sao Tome and Principe | 5810 (5424-6228) | 7967.41 (7434.12-8520.10) | 12272 (11408-13236) | 8194.64 (7639.23-8749.18) | 0.09 (0.09 to 0.10) | < 0.001 |
| Saudi Arabia | 854299 (786274-926817) | 9213.18 (8560.75-9892.33) | 3067523 (2798321-3367524) | 9245.46 (8568.21-9935.62) | 0.01 (-0.01 to 0.03) | 0.25 |
| Senegal | 294300 (271578-318380) | 6986.31 (6472.32-7529.83) | 679249 (627366-734863) | 6614.01 (6185.73-7067.77) | -0.18 (-0.20 to -0.16) | < 0.001 |
| Serbia | 603591 (559791-648669) | 5667.21 (5283.66-6068.98) | 753941 (699986-811166) | 5593.29 (5189.17-6041.03) | -0.04 (-0.05 to -0.03) | < 0.001 |
| Seychelles | 6521 (6032-7044) | 10613.02 (9840.16-11453.24) | 12837 (11856-13936) | 10884.54 (10070.48-11752.96) | 0.09 (0.08 to 0.10) | < 0.001 |
| Sierra Leone | 191300 (177895-206334) | 7521.27 (7006.42-8070.43) | 405349 (372950-439223) | 7610.57 (7071.49-8155.26) | 0.04 (0.03 to 0.05) | < 0.001 |
| Singapore | 269496 (247876-292201) | 9889.29 (9177.06-10490.16) | 738749 (680520-792459) | 9229.95 (8547.75-9877.06) | -0.23 (-0.26 to -0.20) | < 0.001 |
| Slovakia | 357483 (334696-382908) | 6221.57 (5843.73-6644.97) | 486371 (454761-518472) | 5960.02 (5575.94-6352.67) | -0.14 (-0.15 to -0.12) | < 0.001 |
| Slovenia | 142299 (133101-152114) | 6083.57 (5696.48-6507.42) | 213545 (199557-227477) | 5968.15 (5539.43-6369.43) | -0.06 (-0.07 to -0.06) | < 0.001 |
| Solomon Islands | 14747 (13720-15956) | 8066.65 (7526.00-8676.10) | 37933 (34962-41296) | 8231.57 (7603.53-8851.13) | 0.07 (0.06 to 0.07) | < 0.001 |
| Somalia | 224333 (205558-246035) | 5516.90 (5100.65-5954.84) | 603690 (549230-663805) | 5564.97 (5152.59-6029.57) | 0.03 (0.01 to 0.05) | < 0.001 |
| South Africa | 2257302 (2099020-2428551) | 9062.77 (8465.35-9680.97) | 4677573 (4347292-5025759) | 9097.40 (8501.89-9729.11) | 0.02 (0.01 to 0.03) | 0.002 |
| South Sudan | 193641 (177480-211370) | 5618.84 (5175.07-6089.53) | 323627 (296973-354259) | 5730.31 (5317.92-6182.15) | 0.06 (0.04 to 0.08) | < 0.001 |
| Spain | 2768974 (2588950-2935877) | 5464.92 (5131.06-5781.93) | 4288430 (3948305-4576051) | 5022.83 (4690.95-5348.81) | -0.27 (-0.29 to -0.24) | < 0.001 |
| Sri Lanka | 1386481 (1270757-1506959) | 10274.64 (9504.26-11118.70) | 2647885 (2443533-2855363) | 10450.52 (9660.37-11299.19) | 0.06 (0.04 to 0.07) | < 0.001 |
| Sudan | 1061226 (976894-1142517) | 8869.18 (8238.70-9502.05) | 2607644 (2388823-2834157) | 9227.47 (8545.45-9928.63) | 0.13 (0.12 to 0.13) | < 0.001 |
| Suriname | 19697 (18356-21176) | 6671.59 (6215.35-7151.58) | 42747 (39950-45625) | 6878.34 (6435.81-7347.10) | 0.09 (0.08 to 0.11) | < 0.001 |
| Sweden | 821047 (759582-881515) | 6415.63 (5942.97-6890.71) | 1121779 (1040388-1207272) | 6424.01 (5973.50-6887.77) | 0.01 (-0.03 to 0.05) | 0.601 |
| Switzerland | 639460 (600031-681319) | 6582.84 (6179.02-7026.16) | 995630 (934109-1061548) | 6353.97 (5957.15-6788.60) | -0.11 (-0.17 to -0.05) | < 0.001 |
| Syrian Arab Republic | 651505 (602699-705033) | 9137.89 (8502.54-9774.17) | 1213830 (1120670-1309607) | 8947.44 (8264.65-9632.36) | -0.07 (-0.08 to -0.06) | < 0.001 |
| Taiwan (Province of China) | 1249266 (1163816-1344723) | 7155.39 (6728.12-7647.10) | 2418082 (2270339-2579826) | 6571.23 (6152.15-7040.81) | -0.28 (-0.30 to -0.25) | < 0.001 |
| Tajikistan | 322282 (300325-346451) | 9924.67 (9264.55-10630.85) | 708588 (659872-761599) | 9851.20 (9246.71-10479.04) | -0.03 (-0.05 to -0.02) | < 0.001 |
| Thailand | 4699377 (4333121-5101891) | 10448.07 (9717.11-11188.54) | 10328746 (9637500-11140578) | 10839.23 (10107.78-11724.93) | 0.12 (0.10 to 0.14) | < 0.001 |
| Timor-Leste | 45151 (41110-49397) | 9936.45 (9194.89-10715.17) | 104170 (96163-112715) | 10410.83 (9601.84-11301.73) | 0.16 (0.15 to 0.16) | < 0.001 |
| Togo | 131761 (121749-142772) | 7346.00 (6804.03-7869.05) | 382466 (353706-416128) | 7283.93 (6781.11-7815.85) | -0.02 (-0.04 to -0.01) | < 0.001 |
| Tokelau | 107 (99-114) | 8149.51 (7578.01-8739.12) | 121 (112-130) | 8483.99 (7876.48-9158.20) | 0.13 (0.12 to 0.14) | < 0.001 |
| Tonga | 5372 (4996-5776) | 8442.75 (7847.36-9065.87) | 7502 (6986-8081) | 8763.81 (8179.97-9417.79) | 0.12 (0.11 to 0.12) | < 0.001 |
| Trinidad and Tobago | 64179 (59428-68941) | 6674.10 (6204.16-7119.34) | 124244 (115206-133282) | 7009.53 (6525.04-7524.91) | 0.16 (0.14 to 0.17) | < 0.001 |
| Tunisia | 522181 (483654-562934) | 8690.33 (8101.63-9339.38) | 1144106 (1055790-1229704) | 8690.16 (8056.88-9347.17) | 0.00 (0.00 to 0.01) | 0.57 |
| Turkey | 4172206 (3844649-4504302) | 9813.75 (9115.61-10517.89) | 8863959 (8169195-9513046) | 9503.67 (8759.62-10189.42) | -0.10 (-0.12 to -0.08) | < 0.001 |
| Turkmenistan | 254957 (238341-273926) | 11011.08 (10342.05-11708.90) | 484074 (452902-515793) | 10909.7 (10221.7-11599.92) | -0.03 (-0.06 to 0.00) | 0.064 |
| Tuvalu | 609 (566-656) | 8324.33 (7745.82-8960.21) | 948 (879-1026) | 8641.39 (8027.92-9325.05) | 0.12 (0.12 to 0.13) | < 0.001 |
| Uganda | 554191 (507911-603268) | 6119.94 (5653.83-6571.60) | 1414668 (1296989-1547642) | 6081.41 (5638.50-6582.44) | -0.02 (-0.03 to -0.01) | < 0.001 |
| Ukraine | 6244758 (5793184-6760948) | 9772.49 (9071.53-10538.34) | 6394485 (5969352-6867844) | 9814.15 (9113.94-10548.54) | 0.01 (0.01 to 0.02) | < 0.001 |
| United Arab Emirates | 123088 (111138-138046) | 9906.14 (9179.72-10668.90) | 940579 (846409-1053023) | 9512.71 (8796.60-10259.41) | -0.13 (-0.14 to -0.12) | < 0.001 |
| United Kingdom | 5130703 (4779037-5480493) | 6119.79 (5722.53-6520.16) | 6254851 (5837368-6635678) | 5449.07 (5106.17-5788.72) | -0.36 (-0.45 to -0.27) | < 0.001 |
| United Republic of Tanzania | 793891 (729869-860415) | 5368.92 (4960.67-5794.76) | 2186544 (2008467-2390011) | 5861.53 (5421.89-6348.59) | 0.28 (0.27 to 0.30) | < 0.001 |
| United States of America | 22892228 (21306718-24521743) | 7438.31 (6947.03-7942.80) | 38674171 (35778672-41193631) | 7550.75 (7050.60-8067.36) | 0.04 (-0.02 to 0.10) | 0.236 |
| United States Virgin Islands | 6113 (5650-6602) | 6554.33 (6091.76-7043.68) | 9684 (8946-10346) | 6791.65 (6302.59-7285.97) | 0.12 (0.11 to 0.12) | < 0.001 |
| Uruguay | 221996 (206709-237075) | 6192.59 (5767.88-6613.59) | 301874 (280956-322913) | 6282.78 (5818.03-6738.20) | 0.04 (0.01 to 0.07) | 0.005 |
| Uzbekistan | 1462693 (1370470-1565221) | 10757.79 (10084.06-11467.13) | 3273806 (3046385-3512972) | 11069.08 (10338.81-11793.57) | 0.09 (0.08 to 0.10) | < 0.001 |
| Vanuatu | 7356 (6741-7986) | 8514.55 (7893.37-9206.91) | 19551 (18111-21165) | 8886.13 (8277.84-9532.41) | 0.14 (0.13 to 0.14) | < 0.001 |
| Venezuela (Bolivarian Republic of) | 1015584 (943619-1092889) | 8453.02 (7868.51-9056.88) | 2442552 (2275365-2599385) | 8317.30 (7756.36-8846.76) | -0.05 (-0.06 to -0.04) | < 0.001 |
| Viet Nam | 4431264 (4056446-4826467) | 9094.40 (8336.88-9858.13) | 9699996 (8886323-10558442) | 9152.06 (8434.76-9895.76) | 0.02 (0.00 to 0.05) | 0.066 |
| Yemen | 560437 (518019-609175) | 8318.03 (7725.77-8913.91) | 1752190 (1608715-1906736) | 8511.90 (7887.12-9137.90) | 0.07 (0.07 to 0.08) | < 0.001 |
| Zambia | 231401 (212298-251949) | 5513.63 (5099.73-5947.91) | 662316 (603070-727722) | 5769.88 (5330.94-6256.55) | 0.15 (0.14 to 0.15) | < 0.001 |
| Zimbabwe | 445253 (413595-477795) | 8575.21 (7995.12-9192.84) | 804662 (746942-870315) | 8832.91 (8242.96-9439.29) | 0.11 (0.08 to 0.13) | < 0.001 |

**Table S14.** The number and age-standardized rate of mortality for chronic kidney disease in 1990 and 2021, and the temporal trends between 1990-2021 in 204 countries and territories.

| Location | Number of cases, 1990 | Age-standardized rate per 100,000 population, 1990 | Number of cases, 2021 | Age-standardized rate per 100,000 population, 2021 | Average annual percentage change, 1990-2021 | p value |
| --- | --- | --- | --- | --- | --- | --- |
| Afghanistan | 3009 (2175-4675) | 47.31 (34.52-75.86) | 4893 (2926-8625) | 53.88 (32.94-102.20) | 0.44 (0.38 to 0.51) | < 0.001 |
| Albania | 234 (196-277) | 11.99 (10.05-14.31) | 424 (322-541) | 10.60 (8.07-13.41) | -0.48 (-0.83 to -0.13) | 0.008 |
| Algeria | 2344 (1667-4128) | 27.12 (19.03-47.74) | 9785 (7909-12092) | 37.33 (30.61-45.46) | 1.05 (0.83 to 1.28) | < 0.001 |
| American Samoa | 7 (5-10) | 33.53 (24.6-48.13) | 31 (24-39) | 73.81 (57.35-91.7) | 2.74 (2.40 to 3.09) | < 0.001 |
| Andorra | 5 (4-7) | 12.15 (8.81-16.68) | 17 (12-23) | 9.32 (6.85-12.45) | -0.93 (-1.35 to -0.51) | < 0.001 |
| Angola | 1417 (1093-1793) | 37.62 (29.25-46.86) | 4073 (2988-5384) | 39.16 (27.9-50.34) | 0.16 (0.01 to 0.31) | 0.04 |
| Antigua and Barbuda | 17 (16-18) | 30.15 (27.88-32.24) | 46 (42-49) | 47.12 (43.05-50.47) | 1.52 (0.47 to 2.59) | 0.005 |
| Argentina | 9185 (8697-9648) | 30.39 (28.61-31.92) | 15213 (13851-16266) | 26.34 (24.04-28.12) | -0.44 (-1.20 to 0.34) | 0.269 |
| Armenia | 27 (22-31) | 0.97 (0.82-1.13) | 432 (354-523) | 10.25 (8.40-12.44) | 7.94 (5.28 to 10.67) | < 0.001 |
| Australia | 1580 (1436-1674) | 8.72 (7.87-9.26) | 5011 (4201-5515) | 9.41 (8.01-10.32) | 0.15 (-0.98 to 1.28) | 0.8 |
| Austria | 786 (724-834) | 6.58 (6.07-6.98) | 3411 (2801-3759) | 14.28 (11.90-15.62) | 2.62 (1.94 to 3.30) | < 0.001 |
| Azerbaijan | 357 (277-452) | 6.59 (5.09-8.35) | 992 (717-1295) | 10.40 (7.56-13.38) | 1.51 (1.14 to 1.88) | < 0.001 |
| Bahamas | 37 (34-40) | 23.09 (21.31-25.08) | 144 (117-178) | 37.50 (30.66-46.19) | 1.56 (0.67 to 2.46) | 0.001 |
| Bahrain | 48 (36-63) | 41.68 (31.28-55.66) | 259 (207-316) | 52.48 (42.08-63.37) | 0.78 (0.12 to 1.44) | 0.021 |
| Bangladesh | 9553 (6572-11510) | 14.89 (12.84-18.33) | 15175 (11740-20173) | 12.27 (9.51-16.21) | -0.52 (-1.05 to 0.02) | 0.057 |
| Barbados | 60 (57-64) | 20.40 (19.14-21.43) | 148 (117-182) | 29.32 (23.28-35.97) | 1.31 (0.23 to 2.41) | 0.018 |
| Belarus | 100 (93-106) | 0.83 (0.78-0.89) | 360 (296-427) | 2.35 (1.94-2.78) | 3.14 (1.98 to 4.30) | < 0.001 |
| Belgium | 1386 (1260-1478) | 8.81 (7.98-9.39) | 2816 (2274-3150) | 9.00 (7.46-9.99) | 0.22 (-0.25 to 0.69) | 0.366 |
| Belize | 24 (23-26) | 24.40 (22.82-25.97) | 134 (117-151) | 45.45 (39.52-51.13) | 1.95 (1.43 to 2.47) | < 0.001 |
| Benin | 782 (673-912) | 37.31 (32.31-44.65) | 1940 (1558-2414) | 39.41 (32.46-48.40) | 0.19 (0.06 to 0.31) | 0.003 |
| Bermuda | 9 (9-10) | 15.49 (14.55-16.51) | 23 (19-27) | 15.65 (13.18-19.01) | -0.08 (-0.91 to 0.75) | 0.845 |
| Bhutan | 42 (30-58) | 16.98 (12.44-24.17) | 120 (81-165) | 20.86 (14.03-28.51) | 0.66 (0.55 to 0.76) | < 0.001 |
| Bolivia (Plurinational State of) | 1325 (1115-1675) | 42.98 (36.19-53.94) | 4731 (3675-6140) | 58.23 (45.72-75.35) | 0.97 (0.80 to 1.14) | < 0.001 |
| Bosnia and Herzegovina | 361 (307-422) | 9.76 (8.26-11.43) | 686 (512-869) | 10.99 (8.27-13.95) | 0.48 (0.19 to 0.77) | 0.001 |
| Botswana | 109 (79-157) | 23.13 (17.01-32.05) | 348 (270-479) | 28.52 (23.01-39.03) | 0.67 (-0.02 to 1.36) | 0.056 |
| Brazil | 15168 (14505-15699) | 17.98 (16.90-18.76) | 45537 (41271-47987) | 18.70 (16.88-19.75) | 0.23 (-0.24 to 0.70) | 0.336 |
| Brunei Darussalam | 32 (26-42) | 37.51 (30.68-47.76) | 86 (73-100) | 36.33 (30.61-42.27) | -0.17 (-0.46 to 0.12) | 0.25 |
| Bulgaria | 729 (668-785) | 7.63 (6.98-8.17) | 2428 (2049-2872) | 17.38 (14.56-20.82) | 2.63 (1.40 to 3.86) | < 0.001 |
| Burkina Faso | 1555 (1281-1850) | 38.32 (31.80-45.82) | 3812 (3071-4633) | 43.65 (35.29-53.03) | 0.44 (0.30 to 0.58) | < 0.001 |
| Burundi | 909 (711-1148) | 39.49 (31.04-50.64) | 1515 (1109-2172) | 34.97 (25.85-49.93) | -0.42 (-0.65 to -0.20) | < 0.001 |
| Cabo Verde | 38 (32-44) | 15.37 (12.74-17.94) | 124 (84-155) | 28.90 (19.42-35.98) | 2.08 (1.45 to 2.71) | < 0.001 |
| Cambodia | 1050 (857-1277) | 21.21 (17.48-25.70) | 2681 (2007-3484) | 23.47 (17.80-30.16) | 0.35 (0.26 to 0.43) | < 0.001 |
| Cameroon | 2151 (1677-2797) | 52.00 (40.00-67.11) | 5928 (4179-8334) | 51.19 (37.14-71.11) | -0.05 (-0.14 to 0.04) | 0.284 |
| Canada | 2119 (1921-2236) | 6.77 (6.11-7.17) | 7789 (6588-8473) | 9.48 (8.16-10.26) | 1.12 (0.38 to 1.86) | 0.003 |
| Central African Republic | 507 (420-615) | 46.99 (38.58-56.38) | 963 (698-1352) | 47.39 (35.38-63.92) | 0.04 (-0.04 to 0.13) | 0.336 |
| Chad | 751 (593-1013) | 26.39 (20.47-36.89) | 1811 (1330-2568) | 31.60 (23.42-45.57) | 0.58 (0.45 to 0.71) | < 0.001 |
| Chile | 1471 (1390-1543) | 16.00 (14.99-16.88) | 5202 (4582-5596) | 19.81 (17.48-21.30) | 0.56 (-0.21 to 1.33) | 0.154 |
| China | 102726 (89745-120908) | 14.16 (12.44-16.78) | 204230 (164736-246372) | 10.84 (8.77-12.96) | -0.87 (-1.06 to -0.69) | < 0.001 |
| Colombia | 3340 (3177-3471) | 19.52 (18.39-20.41) | 8646 (7184-10116) | 15.48 (12.88-18.12) | -0.92 (-1.32 to -0.51) | < 0.001 |
| Comoros | 70 (54-88) | 38.99 (30.80-47.99) | 182 (132-235) | 42.26 (30.99-54.37) | 0.23 (0.07 to 0.40) | 0.005 |
| Congo | 531 (406-637) | 55.83 (41.04-67.22) | 1252 (847-1626) | 54.79 (35.56-69.00) | -0.05 (-0.29 to 0.20) | 0.703 |
| Cook Islands | 2 (2-3) | 17.50 (13.81-23.18) | 5 (4-7) | 22.01 (16.6-27.23) | 0.76 (0.59 to 0.94) | < 0.001 |
| Costa Rica | 264 (247-279) | 15.27 (14.25-16.16) | 1605 (1391-1794) | 28.95 (25.22-32.30) | 2.06 (1.31 to 2.81) | < 0.001 |
| Coted'Ivoire | 1455 (1171-1787) | 38.90 (32.03-47.66) | 4060 (3151-5240) | 40.49 (32.99-51.20) | 0.14 (0.01 to 0.27) | 0.039 |
| Croatia | 502 (464-540) | 9.47 (8.76-10.19) | 1337 (1158-1549) | 13.47 (11.60-15.74) | 1.16 (0.09 to 2.24) | 0.033 |
| Cuba | 831 (789-866) | 8.31 (7.87-8.67) | 3209 (2758-3638) | 16.14 (13.88-18.31) | 2.28 (1.82 to 2.74) | < 0.001 |
| Cyprus | 188 (156-238) | 41.28 (34.14-51.47) | 343 (284-403) | 21.54 (18.09-25.01) | -1.98 (-2.61 to -1.35) | < 0.001 |
| Czechia | 1096 (999-1200) | 8.18 (7.47-8.90) | 1562 (1326-1820) | 6.80 (5.76-7.99) | -0.51 (-1.28 to 0.27) | 0.201 |
| Democratic People's Republic of Korea | 2021 (1438-2685) | 14.03 (10.07-19.34) | 4298 (3284-5596) | 13.95 (10.65-18.26) | -0.01 (-0.04 to 0.02) | 0.425 |
| Democratic Republic of the Congo | 5868 (4671-7377) | 42.25 (34.51-51.95) | 13845 (9966-18759) | 42.95 (31.38-57.74) | 0.06 (-0.02 to 0.14) | 0.149 |
| Denmark | 413 (379-437) | 4.83 (4.45-5.09) | 1699 (1457-1893) | 12.10 (10.45-13.46) | 3.16 (2.06 to 4.27) | < 0.001 |
| Djibouti | 35 (26-47) | 29.35 (22.40-37.73) | 211 (152-285) | 41.82 (31.38-54.67) | 1.15 (1.11 to 1.19) | < 0.001 |
| Dominica | 19 (16-21) | 32.36 (28.23-36.34) | 40 (32-49) | 50.29 (40.46-61.83) | 1.45 (1.35 to 1.54) | < 0.001 |
| Dominican Republic | 621 (533-750) | 16.66 (14.30-20.34) | 2392 (1646-3064) | 23.99 (16.46-30.70) | 1.34 (0.97 to 1.71) | < 0.001 |
| Ecuador | 1227 (1150-1287) | 23.70 (22.11-24.95) | 6502 (4898-8822) | 42.06 (32.21-56.05) | 1.80 (1.12 to 2.50) | < 0.001 |
| Egypt | 10610 (8478-16104) | 51.75 (40.34-82.61) | 33311 (27183-40816) | 71.73 (59.72-86.59) | 1.13 (0.93 to 1.33) | < 0.001 |
| El Salvador | 797 (702-1141) | 25.26 (22.08-36.29) | 4441 (3314-5504) | 69.81 (52.25-86.75) | 3.38 (2.64 to 4.12) | < 0.001 |
| Equatorial Guinea | 79 (62-98) | 43.75 (35.01-53.38) | 255 (156-372) | 54.70 (31.29-76.52) | 0.76 (0.51 to 1.02) | < 0.001 |
| Eritrea | 358 (267-498) | 32.63 (24.67-45.46) | 902 (610-1462) | 38.08 (26.57-60.61) | 0.49 (0.37 to 0.61) | < 0.001 |
| Estonia | 120 (112-127) | 6.39 (5.98-6.77) | 527 (437-605) | 17.03 (14.19-19.73) | 3.21 (2.01 to 4.43) | < 0.001 |
| Eswatini | 100 (79-125) | 38.66 (30.49-48.34) | 274 (174-383) | 53.52 (36.51-71.62) | 1.10 (0.81 to 1.39) | < 0.001 |
| Ethiopia | 12912 (10097-14949) | 69.02 (56.98-79.61) | 16918 (14059-20068) | 42.38 (34.84-50.16) | -1.56 (-1.72 to -1.39) | < 0.001 |
| Fiji | 95 (67-136) | 27.70 (19.63-40.48) | 314 (231-410) | 47.97 (36.39-61.87) | 1.77 (1.59 to 1.95) | < 0.001 |
| Finland | 214 (194-227) | 3.03 (2.74-3.22) | 818 (657-927) | 4.89 (3.99-5.50) | 1.62 (1.17 to 2.07) | < 0.001 |
| France | 6888 (6157-7355) | 7.75 (6.94-8.26) | 15133 (12606-16843) | 7.33 (6.23-8.09) | -0.14 (-0.55 to 0.27) | 0.511 |
| Gabon | 265 (212-318) | 51.10 (40.66-61.64) | 621 (324-822) | 71.55 (35.5-92.96) | 1.10 (0.86 to 1.34) | < 0.001 |
| Gambia | 115 (90-143) | 33.70 (26.83-41.33) | 403 (305-513) | 43.16 (33.15-54.70) | 0.79 (0.26 to 1.32) | 0.003 |
| Georgia | 197 (161-247) | 3.30 (2.70-4.06) | 713 (574-898) | 12.28 (9.90-15.55) | 4.31 (2.09 to 6.58) | < 0.001 |
| Germany | 11051 (10128-11739) | 8.31 (7.60-8.81) | 39563 (31943-45262) | 15.37 (12.62-17.42) | 2.06 (1.44 to 2.69) | < 0.001 |
| Ghana | 1745 (1343-2490) | 29.52 (22.83-41.83) | 7076 (5563-8854) | 47.67 (37.88-59.27) | 1.56 (1.39 to 1.73) | < 0.001 |
| Greece | 2985 (2744-3174) | 21.33 (19.45-22.71) | 7053 (6077-7641) | 21.35 (18.70-23.01) | -0.10 (-1.92 to 1.76) | 0.917 |
| Greenland | 3 (3-4) | 13.11 (10.99-15.31) | 8 (6-10) | 14.79 (10.65-18.57) | 0.49 (0.27 to 0.70) | < 0.001 |
| Grenada | 27 (24-29) | 35.72 (32.47-39.23) | 62 (54-69) | 58.28 (51.02-64.92) | 1.61 (0.79 to 2.43) | < 0.001 |
| Guam | 14 (12-17) | 22.93 (20.02-28.29) | 51 (42-58) | 24.05 (20.39-27.20) | 0.01 (-0.59 to 0.61) | 0.977 |
| Guatemala | 1108 (1063-1158) | 33.69 (31.98-35.31) | 5448 (4533-6387) | 50.65 (42.25-59.27) | 1.38 (0.54 to 2.22) | 0.001 |
| Guinea | 1055 (850-1342) | 32.32 (25.52-41.77) | 1874 (1414-2582) | 34.51 (25.87-47.82) | 0.21 (0.14 to 0.29) | < 0.001 |
| Guinea-Bissau | 201 (157-246) | 50.12 (40.29-60.74) | 316 (246-404) | 46.37 (36.98-58.88) | -0.25 (-0.35 to -0.14) | < 0.001 |
| Guyana | 117 (106-128) | 30.52 (27.65-33.44) | 349 (269-446) | 56.53 (43.78-71.21) | 2.03 (1.41 to 2.66) | < 0.001 |
| Haiti | 962 (707-1555) | 28.70 (20.53-48.93) | 2177 (1207-4587) | 30.89 (17.21-64.76) | 0.27 (0.14 to 0.39) | < 0.001 |
| Honduras | 259 (215-328) | 12.32 (10.08-15.98) | 1439 (1077-1889) | 24.58 (18.76-31.69) | 2.31 (1.91 to 2.72) | < 0.001 |
| Hungary | 842 (795-888) | 6.26 (5.91-6.59) | 2173 (1852-2483) | 10.14 (8.66-11.63) | 1.78 (1.44 to 2.11) | < 0.001 |
| Iceland | 11 (10-12) | 3.59 (3.23-3.88) | 41 (33-46) | 5.79 (4.71-6.47) | 1.55 (1.18 to 1.93) | < 0.001 |
| India | 58475 (50679-67536) | 13.10 (11.18-15.16) | 175637 (146072-208715) | 15.85 (13.14-18.73) | 0.68 (0.22 to 1.14) | 0.003 |
| Indonesia | 18490 (15846-22280) | 17.33 (14.88-21.77) | 51165 (41553-63408) | 22.98 (18.83-29.04) | 0.92 (0.78 to 1.06) | < 0.001 |
| Iran (Islamic Republic of) | 3718 (2816-5017) | 17.81 (12.99-24.73) | 13627 (11262-15175) | 20.23 (16.49-22.59) | 0.41 (0.12 to 0.69) | 0.005 |
| Iraq | 2948 (2325-3775) | 36.71 (28.74-47.56) | 8279 (5635-10644) | 43.43 (30.46-54.84) | 0.58 (0.31 to 0.84) | < 0.001 |
| Ireland | 311 (288-330) | 8.20 (7.54-8.69) | 703 (587-792) | 8.18 (6.85-9.20) | 0.17 (-1.02 to 1.37) | 0.784 |
| Israel | 970 (893-1037) | 22.19 (20.17-23.75) | 2790 (2314-3075) | 19.69 (16.5-21.68) | -0.51 (-1.20 to 0.18) | 0.146 |
| Italy | 7528 (6774-7922) | 8.76 (7.81-9.24) | 19086 (15314-21515) | 9.29 (7.62-10.48) | 0.22 (-0.11 to 0.55) | 0.188 |
| Jamaica | 434 (408-457) | 23.34 (21.90-24.60) | 935 (707-1205) | 29.15 (22.02-37.49) | 1.17 (-0.99 to 3.38) | 0.289 |
| Japan | 17679 (16010-18557) | 11.61 (10.35-12.26) | 52717 (41038-59205) | 9.48 (7.78-10.42) | -0.69 (-1.07 to -0.31) | < 0.001 |
| Jordan | 360 (293-470) | 31.64 (25.56-41.46) | 1876 (1492-2406) | 33.14 (26.22-41.85) | 0.17 (-0.63 to 0.97) | 0.686 |
| Kazakhstan | 737 (644-832) | 5.33 (4.65-6.05) | 1982 (1631-2335) | 12.22 (10.08-14.43) | 2.74 (1.43 to 4.08) | < 0.001 |
| Kenya | 1950 (1548-3039) | 25.18 (19.62-40.84) | 7330 (5884-9860) | 37.56 (29.77-49.48) | 1.31 (1.13 to 1.50) | < 0.001 |
| Kiribati | 11 (9-13) | 29.96 (24.18-36.76) | 29 (18-42) | 43.10 (28.00-62.31) | 1.20 (1.10 to 1.30) | < 0.001 |
| Kuwait | 160 (144-178) | 30.68 (26.6-34.35) | 374 (276-459) | 15.98 (11.70-19.74) | -2.00 (-3.51 to -0.46) | 0.011 |
| Kyrgyzstan | 159 (141-180) | 4.57 (4.04-5.18) | 498 (415-596) | 9.98 (8.34-11.90) | 2.47 (1.76 to 3.19) | < 0.001 |
| Lao People's Democratic Republic | 917 (677-1191) | 43.94 (33.2-56.93) | 1775 (1242-2408) | 40.89 (29.08-54.72) | -0.23 (-0.28 to -0.18) | < 0.001 |
| Latvia | 95 (89-101) | 2.90 (2.72-3.07) | 321 (265-392) | 7.74 (6.36-9.54) | 3.17 (2.30 to 4.04) | < 0.001 |
| Lebanon | 592 (455-790) | 32.26 (24.68-42.72) | 2102 (1733-2534) | 31.87 (26.28-38.54) | -0.03 (-0.21 to 0.15) | 0.74 |
| Lesotho | 153 (119-202) | 19.86 (15.35-26.5) | 445 (311-595) | 46.19 (32.76-59.42) | 2.86 (2.52 to 3.20) | < 0.001 |
| Liberia | 533 (427-673) | 46.42 (38.02-57.83) | 954 (706-1263) | 48.42 (36.28-63.33) | 0.13 (-0.04 to 0.29) | 0.142 |
| Libya | 503 (374-704) | 28.09 (20.54-40.14) | 2127 (1413-2796) | 46.94 (31.29-61.08) | 1.67 (1.33 to 2.02) | < 0.001 |
| Lithuania | 104 (97-109) | 2.46 (2.31-2.58) | 332 (281-400) | 5.79 (4.87-7.07) | 2.81 (1.49 to 4.15) | < 0.001 |
| Luxembourg | 52 (49-55) | 10.28 (9.50-10.87) | 158 (130-184) | 12.60 (10.53-14.50) | 0.81 (0.23 to 1.39) | 0.006 |
| Madagascar | 1300 (1031-1793) | 26.70 (20.97-38.18) | 2660 (1941-3524) | 26.98 (19.68-35.45) | 0.03 (-0.24 to 0.30) | 0.829 |
| Malawi | 1438 (1170-1736) | 38.69 (31.24-48.98) | 3141 (2543-3818) | 45.45 (37.84-54.52) | 0.56 (0.41 to 0.70) | < 0.001 |
| Malaysia | 2075 (1795-2376) | 22.56 (19.43-25.89) | 7575 (6352-8581) | 29.16 (24.39-32.96) | 0.85 (0.62 to 1.08) | < 0.001 |
| Maldives | 40 (34-51) | 48.40 (39.66-69.29) | 88 (71-105) | 28.89 (23.43-34.46) | -1.74 (-1.88 to -1.59) | < 0.001 |
| Mali | 1405 (1153-1734) | 38.30 (31.27-47.75) | 3001 (2407-3922) | 37.40 (30.33-48.05) | -0.06 (-0.21 to 0.10) | 0.465 |
| Malta | 46 (42-49) | 12.09 (10.91-12.89) | 141 (116-160) | 12.47 (10.44-14.10) | 0.10 (-0.42 to 0.62) | 0.714 |
| Marshall Islands | 5 (3-9) | 32.53 (20.19-58.70) | 17 (6-49) | 54.79 (17.42-160.83) | 1.72 (1.55 to 1.90) | < 0.001 |
| Mauritania | 394 (305-502) | 42.58 (33.22-54.27) | 774 (532-1095) | 40.37 (27.46-56.45) | -0.16 (-0.25 to -0.07) | 0.001 |
| Mauritius | 302 (288-315) | 44.32 (42.02-46.39) | 1414 (1304-1494) | 80.13 (74.12-84.55) | 1.58 (0.64 to 2.53) | 0.001 |
| Mexico | 14133 (13701-14565) | 36.94 (35.44-38.37) | 67044 (58757-76983) | 54.32 (47.78-61.89) | 1.27 (0.26 to 2.29) | 0.014 |
| Micronesia (Federated States of) | 16 (11-25) | 33.55 (22.78-54.50) | 38 (27-53) | 57.60 (42.14-80.91) | 1.78 (1.70 to 1.85) | < 0.001 |
| Monaco | 5 (4-6) | 6.05 (4.64-7.41) | 12 (9-14) | 9.56 (7.61-11.38) | 1.51 (1.37 to 1.65) | < 0.001 |
| Mongolia | 173 (139-213) | 15.37 (12.40-18.87) | 296 (236-365) | 13.61 (10.77-16.75) | -0.36 (-0.72 to 0.01) | 0.056 |
| Montenegro | 76 (61-93) | 12.91 (10.38-15.77) | 165 (124-208) | 19.07 (14.04-23.87) | 1.34 (0.92 to 1.76) | < 0.001 |
| Morocco | 3070 (2131-5653) | 23.11 (15.70-45.57) | 11890 (8911-15306) | 39.98 (30.25-51.29) | 1.76 (1.67 to 1.86) | < 0.001 |
| Mozambique | 1433 (1145-1942) | 26.02 (20.77-37.37) | 3627 (2744-4922) | 35.66 (26.92-48.79) | 1.06 (0.85 to 1.27) | < 0.001 |
| Myanmar | 6501 (4977-8336) | 26.56 (20.56-34.38) | 11244 (9001-14131) | 24.94 (20.30-31.09) | -0.21 (-0.26 to -0.16) | < 0.001 |
| Namibia | 119 (92-169) | 21.79 (16.74-30.97) | 313 (231-422) | 26.65 (20.29-35.15) | 0.70 (0.42 to 0.98) | < 0.001 |
| Nauru | 2 (1-3) | 41.07 (26.11-68.81) | 3 (2-5) | 62.96 (42.86-105.35) | 1.40 (1.30 to 1.51) | < 0.001 |
| Nepal | 1249 (960-1663) | 13.47 (10.41-18.13) | 4048 (2947-5424) | 19.19 (14.08-25.14) | 1.16 (0.97 to 1.34) | < 0.001 |
| Netherlands | 1325 (1179-1417) | 6.65 (5.89-7.11) | 4435 (3715-4925) | 10.89 (9.18-12.06) | 1.62 (1.12 to 2.11) | < 0.001 |
| New Zealand | 271 (249-288) | 7.25 (6.63-7.71) | 957 (835-1041) | 10.67 (9.41-11.56) | 1.25 (0.26 to 2.25) | 0.013 |
| Nicaragua | 492 (444-663) | 29.38 (26.20-40.14) | 2686 (2148-3230) | 55.35 (44.71-66.47) | 2.10 (1.62 to 2.58) | < 0.001 |
| Niger | 874 (706-1119) | 29.49 (23.91-38.74) | 2007 (1440-3038) | 26.96 (19.88-40.06) | -0.28 (-0.36 to -0.20) | < 0.001 |
| Nigeria | 12895 (10282-15658) | 30.97 (25.14-37.63) | 25429 (19318-32599) | 31.27 (24.98-38.12) | 0.03 (-0.04 to 0.11) | 0.384 |
| Niue | 1 (1-1) | 29.88 (23.07-40.27) | 1 (1-2) | 56.11 (33.12-86.18) | 2.01 (1.83 to 2.19) | < 0.001 |
| North Macedonia | 173 (145-208) | 10.08 (8.49-12.16) | 370 (282-484) | 13.59 (10.66-17.26) | 1.09 (0.64 to 1.54) | < 0.001 |
| Northern Mariana Islands | 6 (5-8) | 37.25 (30.15-47.45) | 25 (20-30) | 57.62 (47.32-68.42) | 1.23 (0.88 to 1.59) | < 0.001 |
| Norway | 268 (241-282) | 3.53 (3.18-3.70) | 805 (673-878) | 6.48 (5.50-7.02) | 2.04 (1.32 to 2.76) | < 0.001 |
| Oman | 140 (102-207) | 22.91 (16.58-33.81) | 616 (490-771) | 41.71 (33.02-50.37) | 2.00 (1.14 to 2.87) | < 0.001 |
| Pakistan | 10395 (8266-13167) | 18.75 (14.93-23.71) | 31063 (24330-39844) | 26.59 (21.40-33.90) | 1.12 (1.03 to 1.22) | < 0.001 |
| Palau | 3 (2-4) | 29.53 (21.14-39.31) | 9 (6-12) | 50.19 (36.24-66.32) | 1.69 (1.47 to 1.91) | < 0.001 |
| Palestine | 348 (260-460) | 45.37 (34.16-61.60) | 838 (711-1010) | 43.22 (36.65-51.88) | -0.20 (-0.52 to 0.11) | 0.2 |
| Panama | 201 (188-215) | 13.50 (12.58-14.47) | 1381 (1090-1653) | 30.72 (24.32-36.83) | 2.86 (2.26 to 3.46) | < 0.001 |
| Papua New Guinea | 220 (145-312) | 11.28 (7.85-15.91) | 728 (561-945) | 13.62 (10.35-19.02) | 0.65 (0.43 to 0.86) | < 0.001 |
| Paraguay | 355 (301-418) | 16.14 (13.59-19.07) | 1389 (1036-1761) | 24.92 (18.68-31.54) | 1.48 (1.14 to 1.82) | < 0.001 |
| Peru | 3242 (2796-3748) | 27.00 (23.29-31.43) | 10386 (7395-13295) | 30.94 (22.09-39.66) | 0.53 (-0.76 to 1.84) | 0.421 |
| Philippines | 8626 (7839-9922) | 31.72 (28.86-35.59) | 32883 (27438-38113) | 41.71 (35.01-48.11) | 0.89 (0.64 to 1.14) | < 0.001 |
| Poland | 5309 (5123-5460) | 12.85 (12.33-13.23) | 4981 (4384-5654) | 6.61 (5.81-7.57) | -2.19 (-2.86 to -1.52) | < 0.001 |
| Portugal | 1597 (1486-1687) | 13.03 (12.04-13.78) | 4736 (3954-5301) | 14.51 (12.28-16.23) | 0.34 (-0.16 to 0.83) | 0.183 |
| Puerto Rico | 1091 (1031-1148) | 31.76 (29.90-33.44) | 2537 (2093-2995) | 32.14 (26.55-37.93) | -0.09 (-1.13 to 0.97) | 0.868 |
| Qatar | 28 (16-43) | 40.71 (22.74-65.96) | 198 (153-254) | 39.89 (32.38-49.22) | -0.02 (-1.00 to 0.97) | 0.969 |
| Republic of Korea | 4257 (3775-4740) | 18.61 (16.33-21.39) | 9823 (7902-11473) | 10.84 (8.73-12.65) | -1.77 (-1.88 to -1.65) | < 0.001 |
| Republic of Moldova | 83 (79-87) | 2.02 (1.93-2.12) | 206 (179-242) | 3.61 (3.15-4.25) | 1.71 (-0.10 to 3.56) | 0.064 |
| Romania | 2551 (2408-2682) | 10.42 (9.84-10.94) | 3325 (2869-3831) | 8.62 (7.42-10.04) | -0.68 (-2.06 to 0.71) | 0.336 |
| Russian Federation | 8804 (8612-8979) | 5.14 (5.03-5.25) | 14471 (13013-16149) | 6.18 (5.56-6.89) | 0.69 (-0.64 to 2.04) | 0.311 |
| Rwanda | 1301 (1059-1534) | 47.93 (39.12-56.99) | 2068 (1481-2765) | 38.67 (28.00-51.22) | -0.70 (-0.92 to -0.48) | < 0.001 |
| Saint Kitts and Nevis | 16 (15-17) | 43.57 (40.54-46.80) | 34 (28-40) | 57.57 (48.51-65.85) | 0.93 (0.22 to 1.65) | 0.01 |
| Saint Lucia | 30 (29-32) | 37.32 (35.33-39.16) | 93 (77-111) | 40.31 (33.50-47.93) | 0.25 (-0.19 to 0.69) | 0.262 |
| Saint Vincent and the Grenadines | 19 (17-20) | 26.90 (24.86-28.68) | 54 (48-61) | 41.06 (36.19-46.44) | 1.31 (0.42 to 2.20) | 0.004 |
| Samoa | 24 (17-33) | 31.00 (22.20-41.05) | 58 (44-75) | 43.46 (33.04-55.51) | 1.10 (0.97 to 1.22) | < 0.001 |
| San Marino | 2 (2-2) | 5.46 (4.53-6.57) | 4 (3-6) | 3.89 (2.64-5.26) | -1.17 (-1.75 to -0.59) | < 0.001 |
| Sao Tome and Principe | 34 (28-38) | 53.67 (45.26-60.79) | 69 (50-85) | 72.37 (51.38-86.92) | 0.95 (0.69 to 1.20) | < 0.001 |
| Saudi Arabia | 2408 (1798-3342) | 45.07 (33.47-64.13) | 12226 (9096-15498) | 79.26 (59.76-95.73) | 1.82 (1.66 to 1.99) | < 0.001 |
| Senegal | 1300 (1084-1593) | 40.62 (34.05-50.30) | 3035 (2315-4175) | 43.53 (33.56-59.16) | 0.15 (0.02 to 0.27) | 0.019 |
| Serbia | 1464 (1214-1828) | 16.49 (13.61-20.76) | 2713 (2196-3252) | 15.88 (12.83-19.07) | 0.00 (-0.31 to 0.30) | 0.976 |
| Seychelles | 17 (15-19) | 29.29 (25.52-33.44) | 50 (40-58) | 47.22 (37.91-55.38) | 1.48 (1.21 to 1.76) | < 0.001 |
| Sierra Leone | 628 (495-784) | 30.08 (23.77-37.16) | 1048 (802-1392) | 28.95 (22.59-37.45) | -0.12 (-0.24 to 0.01) | 0.063 |
| Singapore | 264 (250-275) | 13.74 (12.83-14.43) | 833 (718-926) | 10.18 (8.73-11.34) | -0.90 (-1.48 to -0.32) | 0.003 |
| Slovakia | 617 (530-708) | 10.59 (9.14-12.20) | 831 (677-1002) | 8.90 (7.25-10.74) | -0.59 (-0.89 to -0.30) | < 0.001 |
| Slovenia | 152 (142-163) | 6.37 (5.92-6.88) | 327 (261-415) | 6.10 (4.88-7.81) | -0.05 (-0.52 to 0.43) | 0.85 |
| Solomon Islands | 31 (13-50) | 22.14 (11.64-34.59) | 90 (69-116) | 25.03 (20.06-31.45) | 0.41 (0.07 to 0.75) | 0.018 |
| Somalia | 1051 (755-1454) | 45.70 (33.39-63.19) | 2791 (1917-4130) | 47.94 (33.14-68.33) | 0.17 (0.05 to 0.29) | 0.006 |
| South Africa | 4020 (3508-4993) | 19.75 (16.85-25.08) | 13502 (12124-14916) | 33.24 (29.60-36.64) | 1.69 (1.26 to 2.13) | < 0.001 |
| South Sudan | 1009 (753-1391) | 40.95 (31.05-56.24) | 1949 (1428-2539) | 54.98 (39.86-71.17) | 0.96 (0.87 to 1.06) | < 0.001 |
| Spain | 6907 (6235-7340) | 13.30 (11.93-14.14) | 14802 (11854-16621) | 10.49 (8.59-11.76) | -0.70 (-0.99 to -0.40) | < 0.001 |
| Sri Lanka | 2614 (2219-3080) | 27.67 (23.33-32.77) | 5243 (3567-7155) | 21.22 (14.56-28.53) | -0.78 (-1.35 to -0.21) | 0.007 |
| Sudan | 2043 (1462-3607) | 22.73 (15.89-43.49) | 5602 (4127-7495) | 32.06 (24.26-43.82) | 1.13 (1.06 to 1.20) | < 0.001 |
| Suriname | 71 (61-81) | 27.68 (24.19-31.82) | 253 (189-329) | 41.40 (31.04-53.55) | 1.39 (0.92 to 1.86) | < 0.001 |
| Sweden | 636 (578-678) | 3.82 (3.48-4.07) | 2518 (2087-2834) | 8.85 (7.43-9.92) | 2.74 (2.06 to 3.41) | < 0.001 |
| Switzerland | 808 (722-860) | 7.15 (6.43-7.61) | 2599 (2042-2973) | 10.21 (8.14-11.71) | 1.28 (0.74 to 1.82) | < 0.001 |
| Syrian Arab Republic | 1951 (1524-2563) | 39.11 (30.41-52.82) | 4836 (3387-6504) | 47.74 (33.59-62.79) | 0.64 (0.42 to 0.86) | < 0.001 |
| Taiwan (Province of China) | 2954 (2802-3083) | 24.13 (22.45-25.37) | 8814 (7593-9771) | 20.00 (17.37-22.09) | -0.64 (-1.24 to -0.03) | 0.039 |
| Tajikistan | 59 (48-83) | 1.76 (1.42-2.56) | 152 (106-220) | 2.37 (1.68-3.26) | 0.94 (0.56 to 1.32) | < 0.001 |
| Thailand | 7941 (6624-9995) | 24.26 (20.24-30.58) | 31734 (24197-39501) | 29.90 (22.77-37.14) | 0.63 (0.43 to 0.84) | < 0.001 |
| Timor-Leste | 76 (57-101) | 25.64 (19.69-35.58) | 238 (172-335) | 29.76 (21.78-41.42) | 0.45 (0.27 to 0.64) | < 0.001 |
| Togo | 381 (314-467) | 30.96 (26.13-38.00) | 1136 (854-1520) | 34.53 (26.70-45.18) | 0.35 (0.25 to 0.46) | < 0.001 |
| Tokelau | 0 (0-1) | 24.56 (17.15-42.52) | 1 (0-1) | 37.26 (27.02-54.74) | 1.39 (1.23 to 1.54) | < 0.001 |
| Tonga | 7 (5-10) | 13.38 (9.39-18.57) | 16 (11-21) | 20.55 (14.34-27.02) | 1.43 (1.16 to 1.69) | < 0.001 |
| Trinidad and Tobago | 192 (184-201) | 24.70 (23.52-25.82) | 755 (564-967) | 40.17 (30.11-51.33) | 1.66 (0.78 to 2.54) | < 0.001 |
| Tunisia | 926 (706-1392) | 22.22 (16.99-33.34) | 3765 (2674-5162) | 32.12 (23.11-43.87) | 1.14 (0.91 to 1.37) | < 0.001 |
| Turkey | 9286 (7515-12586) | 30.59 (24.46-42.54) | 24805 (19764-30524) | 29.53 (23.34-36.39) | -0.02 (-0.36 to 0.31) | 0.892 |
| Turkmenistan | 209 (191-228) | 8.94 (8.12-9.83) | 744 (574-947) | 17.99 (13.95-22.74) | 2.19 (1.02 to 3.37) | < 0.001 |
| Tuvalu | 2 (1-3) | 27.78 (20.77-43.65) | 4 (3-6) | 40.69 (29.68-58.88) | 1.25 (1.17 to 1.33) | < 0.001 |
| Uganda | 1868 (1404-2443) | 30.99 (23.46-40.74) | 5279 (4093-6903) | 38.83 (30.81-50.76) | 0.72 (0.64 to 0.81) | < 0.001 |
| Ukraine | 84 (80-88) | 0.13 (0.13-0.14) | 1605 (1204-2049) | 2.40 (1.79-3.07) | 9.81 (7.35 to 12.33) | < 0.001 |
| United Arab Emirates | 83 (57-119) | 23.06 (16.05-32.58) | 672 (427-914) | 37.54 (20.93-49.19) | 1.57 (-0.84 to 4.04) | 0.204 |
| United Kingdom | 4239 (3908-4410) | 4.60 (4.23-4.79) | 9679 (8207-10541) | 6.25 (5.36-6.79) | 1.00 (0.41 to 1.60) | 0.001 |
| United Republic of Tanzania | 3665 (3049-4635) | 36.08 (30.25-46.22) | 8860 (7121-11155) | 37.65 (30.14-46.93) | 0.15 (0.02 to 0.29) | 0.029 |
| United States of America | 27826 (25245-29210) | 8.49 (7.72-8.90) | 135880 (118167-146606) | 21.93 (19.31-23.51) | 3.11 (2.92 to 3.29) | < 0.001 |
| United States Virgin Islands | 15 (12-19) | 19.89 (16.55-23.98) | 33 (23-43) | 20.47 (14.73-26.56) | 0.07 (-0.27 to 0.42) | 0.667 |
| Uruguay | 598 (561-628) | 15.50 (14.50-16.23) | 1167 (1034-1270) | 17.91 (16.11-19.42) | 0.47 (-0.22 to 1.18) | 0.182 |
| Uzbekistan | 617 (441-920) | 5.05 (3.43-7.88) | 3589 (3013-4204) | 14.24 (12.06-16.66) | 3.48 (1.77 to 5.21) | < 0.001 |
| Vanuatu | 13 (8-22) | 22.02 (14.27-38.66) | 55 (40-85) | 34.34 (24.70-52.56) | 1.45 (1.31 to 1.59) | < 0.001 |
| Venezuela (Bolivarian Republic of) | 1587 (1501-1651) | 16.32 (15.23-17.09) | 11753 (8833-14984) | 40.52 (30.44-51.29) | 2.95 (2.46 to 3.45) | < 0.001 |
| Viet Nam | 9150 (6657-11695) | 23.45 (17.13-30.09) | 23706 (16677-29759) | 27.03 (19.07-33.40) | 0.47 (0.41 to 0.53) | < 0.001 |
| Yemen | 871 (580-1629) | 19.15 (12.74-38.89) | 2471 (1678-4179) | 20.63 (14.22-34.62) | 0.26 (0.10 to 0.42) | 0.002 |
| Zambia | 1293 (1071-1518) | 46.31 (39.52-54.33) | 3432 (2424-4962) | 52.24 (39.21-69.77) | 0.41 (0.23 to 0.59) | < 0.001 |
| Zimbabwe | 874 (693-1148) | 26.00 (20.48-34.24) | 2483 (1893-3317) | 41.43 (31.84-53.59) | 1.61 (1.21 to 2.01) | < 0.001 |

**Table S15.** The number and age-standardized rate of DALYs for chronic kidney disease in 1990 and 2021, and the temporal trends between 1990-2021 in 204 countries and territories.

| Location | Number of cases, 1990 | Age-standardized rate per 100,000 population, 1990 | Number of cases, 2021 | Age-standardized rate per 100,000 population, 2021 | Average annual percentage change, 1990-2021 | p value |
| --- | --- | --- | --- | --- | --- | --- |
| Afghanistan | 94773 (70923-137292) | 1274.29 (955.98-1918.62) | 173983 (108001-286182) | 1343.24 (830.87-2358.01) | 0.17 (0.07 to 0.27) | 0.001 |
| Albania | 9416 (8198-10999) | 378.25 (328.57-441.42) | 11183 (9088-13561) | 303.95 (249.15-366.31) | -0.78 (-1.06 to -0.51) | < 0.001 |
| Algeria | 79125 (60711-119848) | 593.89 (442.44-962.72) | 241373 (197533-295747) | 724.15 (595.94-868.77) | 0.66 (0.56 to 0.75) | < 0.001 |
| American Samoa | 260 (201-353) | 953.16 (740.75-1294.18) | 938 (740-1163) | 1950.94 (1545.88-2408.41) | 2.49 (2.18 to 2.80) | < 0.001 |
| Andorra | 148 (115-187) | 286.89 (224.78-360.92) | 357 (285-435) | 229.21 (181.74-279.93) | -0.82 (-1.04 to -0.59) | < 0.001 |
| Angola | 60560 (47304-74043) | 1068.56 (834.87-1326.96) | 158051 (120480-207619) | 1011.81 (761.71-1314.95) | -0.15 (-0.44 to 0.14) | 0.304 |
| Antigua and Barbuda | 436 (405-466) | 807.80 (751.92-862.86) | 1162 (1070-1242) | 1116.49 (1028.08-1190.11) | 0.97 (0.13 to 1.82) | 0.024 |
| Argentina | 223942 (212662-235443) | 705.33 (669.33-740.30) | 315536 (295283-335059) | 573.23 (537.71-608.51) | -0.59 (-0.87 to -0.31) | < 0.001 |
| Armenia | 4757 (3535-5893) | 168.21 (126.18-208.59) | 15239 (12675-18395) | 380.18 (316.18-457.36) | 2.72 (1.61 to 3.84) | < 0.001 |
| Australia | 38617 (35222-42200) | 211.20 (193.05-230.14) | 93074 (82639-102916) | 207.14 (183.49-229.01) | -0.16 (-0.60 to 0.28) | 0.478 |
| Austria | 23090 (20228-25966) | 213.38 (187.00-238.48) | 56356 (49530-62593) | 288.20 (253.46-323.87) | 1.04 (0.53 to 1.54) | < 0.001 |
| Azerbaijan | 22175 (18576-26606) | 374.09 (313.47-447.81) | 47430 (37765-57959) | 451.90 (364.79-546.71) | 0.63 (0.31 to 0.95) | < 0.001 |
| Bahamas | 1314 (1209-1413) | 710.66 (653.57-766.60) | 4315 (3520-5314) | 1035.59 (848.99-1270.93) | 1.23 (0.51 to 1.96) | 0.001 |
| Bahrain | 1611 (1272-2077) | 856.94 (670.32-1110.27) | 7698 (6382-9087) | 963.54 (793.27-1153.68) | 0.39 (-0.02 to 0.81) | 0.065 |
| Bangladesh | 551460 (306452-697970) | 604.30 (447.85-701.97) | 577364 (481335-715354) | 407.97 (343.85-505.99) | -1.22 (-1.35 to -1.10) | < 0.001 |
| Barbados | 1555 (1455-1646) | 564.83 (528.52-598.06) | 3422 (2743-4181) | 732.57 (589.23-902.98) | 0.98 (0.06 to 1.90) | 0.036 |
| Belarus | 13038 (10184-15922) | 109.57 (86.12-132.79) | 21603 (17974-26090) | 149.78 (125.81-179.79) | 1.03 (0.58 to 1.49) | < 0.001 |
| Belgium | 36155 (31529-40640) | 248.07 (215.35-279.24) | 55840 (47926-63208) | 234.03 (201.28-266.85) | -0.18 (-0.45 to 0.10) | 0.214 |
| Belize | 848 (796-903) | 718.79 (674.47-763.42) | 4198 (3725-4677) | 1236.14 (1098.78-1377.85) | 1.70 (1.15 to 2.25) | < 0.001 |
| Benin | 29653 (25048-34516) | 999.34 (868.44-1155.68) | 71721 (57427-87409) | 990.10 (808.63-1210.26) | -0.02 (-0.18 to 0.14) | 0.789 |
| Bermuda | 264 (244-284) | 421.43 (389.52-452.83) | 482 (414-570) | 395.45 (339.73-465.48) | -0.27 (-0.71 to 0.17) | 0.232 |
| Bhutan | 1915 (1398-2531) | 571.18 (432.97-749.01) | 3865 (2821-5112) | 600.10 (438.76-786.09) | 0.18 (0.07 to 0.28) | 0.001 |
| Bolivia (Plurinational State of) | 45375 (38779-56751) | 1123.66 (953.42-1408.46) | 121335 (92262-157620) | 1307.42 (1008.75-1688.68) | 0.52 (0.46 to 0.58) | < 0.001 |
| Bosnia and Herzegovina | 13424 (11787-15111) | 326.86 (287.86-366.20) | 17500 (14014-21236) | 311.17 (253.16-378.48) | -0.09 (-0.35 to 0.17) | 0.485 |
| Botswana | 4159 (3143-5808) | 645.59 (492.96-883.78) | 11766 (9282-16126) | 734.52 (596.11-978.22) | 0.41 (-0.06 to 0.88) | 0.089 |
| Brazil | 582735 (547944-618485) | 565.18 (529.70-600.78) | 1273112 (1182007-1361964) | 513.85 (476.98-550.10) | -0.26 (-0.48 to -0.04) | 0.02 |
| Brunei Darussalam | 1038 (881-1248) | 843.62 (710.86-1036.17) | 2607 (2282-2940) | 776.58 (676.45-884.25) | -0.31 (-0.46 to -0.15) | < 0.001 |
| Bulgaria | 27816 (24930-30650) | 274.08 (246.81-301.38) | 59631 (50184-71490) | 487.91 (408.34-590.67) | 1.83 (0.97 to 2.70) | < 0.001 |
| Burkina Faso | 60272 (50415-71118) | 1014.18 (852.47-1190.59) | 140312 (112697-173625) | 1094.71 (897.51-1322.28) | 0.27 (0.08 to 0.47) | 0.006 |
| Burundi | 31943 (25440-39942) | 1032.58 (815.32-1304.68) | 52752 (39451-75471) | 837.29 (623.89-1184.66) | -0.70 (-0.92 to -0.48) | < 0.001 |
| Cabo Verde | 1204 (1015-1391) | 462.89 (388.99-534.49) | 3178 (2305-3934) | 677.38 (486.86-834.96) | 1.24 (1.09 to 1.39) | < 0.001 |
| Cambodia | 48373 (39570-58172) | 757.37 (633.33-895.87) | 101003 (77788-132516) | 733.58 (574.41-943.93) | -0.09 (-0.20 to 0.03) | 0.152 |
| Cameroon | 80907 (63460-102530) | 1348.10 (1082.99-1721.87) | 224422 (163790-306794) | 1319.97 (973.00-1795.06) | -0.08 (-0.17 to 0.02) | 0.124 |
| Canada | 55398 (49172-61889) | 178.37 (158.92-198.89) | 150955 (134749-165748) | 224.70 (200.23-246.05) | 0.76 (0.42 to 1.11) | < 0.001 |
| Central African Republic | 20433 (16924-24299) | 1327.27 (1105.97-1592.63) | 39107 (28354-53349) | 1303.33 (972.36-1758.22) | -0.06 (-0.17 to 0.05) | 0.295 |
| Chad | 28291 (22969-36811) | 736.77 (598.10-968.73) | 73527 (56687-98420) | 840.46 (646.72-1175.37) | 0.46 (0.39 to 0.52) | < 0.001 |
| Chile | 43420 (40600-46183) | 413.69 (384.78-440.39) | 104137 (95786-111766) | 415.15 (383.43-445.69) | -0.07 (-0.72 to 0.58) | 0.824 |
| China | 4195631 (3673738-4813077) | 457.69 (402.14-526.92) | 6127923 (5184384-7208227) | 315.33 (266.58-371.53) | -1.20 (-1.31 to -1.09) | < 0.001 |
| Colombia | 122275 (114363-131312) | 573.83 (533.11-613.66) | 233106 (198873-269966) | 424.45 (362.45-491.30) | -1.11 (-1.44 to -0.78) | < 0.001 |
| Comoros | 2494 (1867-3109) | 984.80 (763.59-1217.01) | 5242 (3955-6654) | 990.68 (748.15-1260.42) | -0.04 (-0.66 to 0.59) | 0.907 |
| Congo | 19457 (15403-23362) | 1483.45 (1157.43-1769.47) | 44198 (31384-57320) | 1365.57 (964.25-1712.34) | -0.24 (-0.50 to 0.01) | 0.057 |
| Cook Islands | 71 (59-89) | 535.04 (446.10-669.07) | 151 (120-180) | 631.41 (503.79-752.99) | 0.54 (0.41 to 0.68) | < 0.001 |
| Costa Rica | 9157 (8313-10118) | 468.52 (425.52-515.95) | 41809 (37382-46132) | 764.45 (683.83-842.99) | 1.63 (0.93 to 2.34) | < 0.001 |
| Coted'Ivoire | 62444 (50103-77072) | 1012.18 (827.29-1226.77) | 153321 (116483-194558) | 1019.68 (809.33-1277.16) | 0.03 (-0.13 to 0.19) | 0.687 |
| Croatia | 15608 (14199-16944) | 284.15 (259.01-307.03) | 26558 (22898-31103) | 308.78 (265.45-364.88) | 0.27 (-0.56 to 1.10) | 0.525 |
| Cuba | 29762 (27495-32222) | 285.64 (263.50-308.72) | 79000 (69453-88900) | 436.64 (383.77-494.15) | 1.53 (0.98 to 2.08) | < 0.001 |
| Cyprus | 3922 (3338-4773) | 660.43 (562.01-797.89) | 6772 (5876-7764) | 377.75 (329.25-432.39) | -1.78 (-2.15 to -1.41) | < 0.001 |
| Czechia | 35113 (31942-38431) | 277.03 (253.00-303.17) | 39158 (33376-45630) | 197.69 (167.80-233.11) | -1.10 (-1.62 to -0.57) | < 0.001 |
| Democratic People's Republic of Korea | 83478 (63914-105819) | 483.43 (373.12-605.87) | 153167 (124247-191899) | 479.21 (390.56-602.59) | -0.03 (-0.05 to -0.01) | 0.002 |
| Democratic Republic of the Congo | 238276 (194665-290653) | 1138.92 (935.70-1393.94) | 513717 (387253-677593) | 1112.28 (844.16-1452.76) | -0.08 (-0.17 to 0.02) | 0.106 |
| Denmark | 13497 (11412-15540) | 177.56 (150.59-205.12) | 31945 (28185-35763) | 266.66 (235.02-300.93) | 1.44 (1.05 to 1.84) | < 0.001 |
| Djibouti | 1395 (1048-1830) | 727.50 (561.13-939.78) | 6952 (5050-9547) | 959.05 (723.50-1275.45) | 0.88 (0.80 to 0.96) | < 0.001 |
| Dominica | 502 (447-555) | 836.06 (744.57-925.44) | 1029 (819-1263) | 1295.53 (1035.63-1585.14) | 1.44 (1.37 to 1.51) | < 0.001 |
| Dominican Republic | 25415 (22411-29374) | 515.44 (449.45-606.13) | 73332 (54273-90211) | 701.53 (518.50-864.79) | 1.06 (0.68 to 1.45) | < 0.001 |
| Ecuador | 40275 (37767-42623) | 619.34 (581.45-655.64) | 155833 (114778-216359) | 950.39 (705.52-1310.61) | 1.34 (0.65 to 2.03) | < 0.001 |
| Egypt | 344984 (287412-482687) | 1140.62 (932.58-1686.68) | 933318 (767956-1131670) | 1501.66 (1248.83-1804.26) | 0.92 (0.75 to 1.10) | < 0.001 |
| El Salvador | 28536 (25800-38102) | 784.26 (704.61-1063.58) | 118020 (88527-144949) | 1904.01 (1419.63-2342.53) | 3.08 (2.80 to 3.36) | < 0.001 |
| Equatorial Guinea | 3014 (2404-3713) | 1226.61 (976.57-1504.50) | 9444 (6004-13862) | 1323.06 (834.28-1860.78) | 0.34 (0.11 to 0.57) | 0.004 |
| Eritrea | 15112 (11352-20868) | 900.21 (680.90-1231.14) | 32532 (22413-51729) | 930.22 (649.13-1463.48) | 0.08 (-0.07 to 0.23) | 0.294 |
| Estonia | 5689 (5107-6257) | 315.43 (284.60-344.78) | 11443 (9973-13008) | 455.76 (396.13-518.50) | 1.18 (0.68 to 1.69) | < 0.001 |
| Eswatini | 3616 (2910-4479) | 1010.49 (813.54-1237.87) | 9663 (6172-13306) | 1431.04 (945-1924.97) | 1.17 (0.88 to 1.46) | < 0.001 |
| Ethiopia | 466721 (355203-540047) | 1765.66 (1400.45-2039.43) | 524752 (440481-620029) | 966.00 (806.93-1140.34) | -1.93 (-2.09 to -1.78) | < 0.001 |
| Fiji | 3880 (2939-5283) | 849.72 (647.64-1147.10) | 10463 (7966-13388) | 1312.98 (1004.23-1661.09) | 1.38 (1.15 to 1.61) | < 0.001 |
| Finland | 8482 (7019-9973) | 126.01 (103.95-147.99) | 18425 (15613-21274) | 145.15 (120.51-168.55) | 0.48 (0.29 to 0.67) | < 0.001 |
| France | 146507 (130176-162496) | 180.76 (160.54-199.74) | 266719 (232254-297859) | 174.03 (151.48-196.15) | -0.08 (-0.36 to 0.21) | 0.59 |
| Gabon | 8190 (6669-9730) | 1304.75 (1072.43-1558.00) | 19184 (11205-25666) | 1670.81 (951.89-2188.47) | 0.80 (0.55 to 1.06) | < 0.001 |
| Gambia | 4626 (3620-5727) | 892.62 (713.95-1097.58) | 13852 (10535-17707) | 1074.77 (822.36-1368.13) | 0.59 (-0.25 to 1.43) | 0.17 |
| Georgia | 15560 (12706-18459) | 264.19 (216.63-310.62) | 24983 (20472-30628) | 478.54 (394.29-587.65) | 1.67 (1.14 to 2.20) | < 0.001 |
| Germany | 300086 (265594-331706) | 249.22 (219.66-276.89) | 635444 (555490-709238) | 302.92 (265.96-338.81) | 0.66 (0.28 to 1.05) | 0.001 |
| Ghana | 67702 (52675-92629) | 772.79 (608.91-1076.69) | 237458 (184981-297856) | 1144.99 (904.59-1420.12) | 1.29 (1.06 to 1.51) | < 0.001 |
| Greece | 59717 (54719-64585) | 424.92 (388.70-459.38) | 112985 (100431-122488) | 435.48 (396.06-475.30) | 0.20 (-1.00 to 1.42) | 0.746 |
| Greenland | 117 (102-135) | 335.71 (294.94-381.58) | 216 (178-257) | 352.22 (285.85-418.33) | 0.26 (0.11 to 0.41) | 0.001 |
| Grenada | 742 (678-812) | 1044.67 (958.18-1144.04) | 1684 (1467-1904) | 1487.17 (1300.75-1673.58) | 1.19 (0.56 to 1.82) | < 0.001 |
| Guam | 535 (470-650) | 626.57 (552.23-754.35) | 1682 (1452-1875) | 846.82 (733.41-946.49) | 0.94 (0.63 to 1.24) | < 0.001 |
| Guatemala | 44304 (42312-46649) | 920.20 (874.26-970.62) | 167359 (143276-194780) | 1387.50 (1182.18-1617.19) | 1.35 (0.48 to 2.24) | 0.002 |
| Guinea | 39480 (32723-48547) | 900.19 (741.28-1118.65) | 67059 (52117-90058) | 896.96 (697.85-1221.30) | -0.01 (-0.13 to 0.11) | 0.859 |
| Guinea-Bissau | 8272 (6561-10146) | 1414.38 (1129.62-1709.67) | 12715 (9952-16050) | 1220.65 (970.38-1525.53) | -0.47 (-0.58 to -0.36) | < 0.001 |
| Guyana | 4232 (3811-4631) | 883.22 (800.53-971.40) | 10889 (8428-13860) | 1574.82 (1227.32-1986.44) | 1.91 (1.25 to 2.57) | < 0.001 |
| Haiti | 40217 (31355-58452) | 909.47 (697.65-1415.13) | 82512 (50633-163742) | 916.41 (561.09-1807.57) | 0.07 (-0.08 to 0.23) | 0.364 |
| Honduras | 11630 (10042-13654) | 444.70 (379.37-536.18) | 46141 (35816-59163) | 682.72 (536.58-870.96) | 1.42 (1.13 to 1.71) | < 0.001 |
| Hungary | 30171 (27070-33010) | 235.27 (211.20-256.76) | 46215 (39960-53280) | 249.28 (214.43-289.00) | 0.23 (0.01 to 0.46) | 0.041 |
| Iceland | 348 (298-399) | 120.54 (102.80-138.35) | 884 (755-1011) | 149.81 (127.15-172.36) | 0.74 (0.55 to 0.94) | < 0.001 |
| India | 2650925 (2335452-3030444) | 481.56 (422.54-547.62) | 6487629 (5592187-7501487) | 521.84 (451.82-601.82) | 0.28 (-0.04 to 0.60) | 0.089 |
| Indonesia | 875942 (750362-1005150) | 664.03 (575.98-770.26) | 1990496 (1655069-2360240) | 760.37 (644.61-906.69) | 0.45 (0.35 to 0.55) | < 0.001 |
| Iran (Islamic Republic of) | 139435 (116402-174468) | 463.62 (372.31-595.58) | 356124 (310077-393518) | 467.46 (406.76-515.06) | 0.02 (-0.13 to 0.17) | 0.783 |
| Iraq | 94335 (75090-120601) | 950.26 (757.69-1205.44) | 232396 (157615-296780) | 940.84 (660.57-1195.03) | -0.01 (-0.22 to 0.20) | 0.924 |
| Ireland | 9959 (8464-11701) | 256.44 (219.03-299.89) | 18990 (16118-22176) | 246.73 (206.96-289.36) | -0.13 (-0.69 to 0.43) | 0.649 |
| Israel | 22516 (20420-24257) | 480.75 (435.30-518.10) | 52713 (46207-58485) | 417.34 (366.33-463.46) | -0.50 (-1.03 to 0.03) | 0.066 |
| Italy | 198890 (174312-221494) | 245.83 (215.46-275.21) | 328820 (282849-371176) | 216.97 (185.00-247.90) | -0.43 (-0.60 to -0.25) | < 0.001 |
| Jamaica | 11644 (10948-12362) | 617.40 (578.48-656.55) | 25773 (19982-32671) | 831.31 (642.36-1055.41) | 1.42 (-0.48 to 3.37) | 0.144 |
| Japan | 460213 (416011-499612) | 290.39 (262.49-315.05) | 923022 (792680-1028099) | 239.94 (209.67-266.06) | -0.68 (-0.92 to -0.43) | < 0.001 |
| Jordan | 12490 (10264-15613) | 749.85 (617.21-949.47) | 54300 (44489-67799) | 710.10 (581.36-884.90) | -0.14 (-0.69 to 0.41) | 0.605 |
| Kazakhstan | 49636 (42866-56925) | 351.46 (300.01-403.84) | 80323 (68213-94683) | 448.35 (381.60-524.18) | 0.91 (0.07 to 1.75) | 0.034 |
| Kenya | 66436 (54525-94453) | 608.86 (488.93-921.43) | 230896 (188910-304540) | 863.66 (701.70-1139.37) | 1.14 (0.97 to 1.30) | < 0.001 |
| Kiribati | 460 (380-548) | 969.96 (797.74-1154.42) | 1109 (759-1633) | 1285.47 (892.42-1861.85) | 0.93 (0.85 to 1.02) | < 0.001 |
| Kuwait | 5748 (5276-6312) | 716.82 (647.33-792.74) | 11585 (9417-13665) | 360.69 (286.12-427.92) | -2.33 (-3.57 to -1.07) | < 0.001 |
| Kyrgyzstan | 12369 (10585-14240) | 346.85 (294.57-401.76) | 25313 (21875-29466) | 448.27 (386.94-523.65) | 0.70 (0.19 to 1.20) | 0.007 |
| Lao People's Democratic Republic | 36532 (27933-46903) | 1380.46 (1067.06-1764.16) | 63295 (45478-85994) | 1174.57 (854.11-1578.45) | -0.52 (-0.58 to -0.46) | < 0.001 |
| Latvia | 5859 (5024-6735) | 184.25 (159.87-210.47) | 9429 (7832-11172) | 271.26 (225.78-322.44) | 1.21 (0.80 to 1.62) | < 0.001 |
| Lebanon | 16012 (12337-21059) | 730.51 (569.04-955.01) | 39230 (33152-46228) | 630.32 (532.31-739.35) | -0.48 (-0.63 to -0.33) | < 0.001 |
| Lesotho | 5121 (4128-6439) | 560.65 (450.89-707.10) | 14547 (10549-19456) | 1221.51 (900.96-1605.09) | 2.64 (2.33 to 2.95) | < 0.001 |
| Liberia | 20964 (16740-25988) | 1240.99 (1006.84-1562.17) | 35243 (26717-46785) | 1197.03 (916.25-1548.13) | -0.13 (-0.33 to 0.07) | 0.198 |
| Libya | 15078 (11908-19609) | 675.23 (524.42-903.51) | 58512 (37581-76498) | 1066.96 (712.68-1379.31) | 1.48 (1.23 to 1.73) | < 0.001 |
| Lithuania | 7168 (6069-8288) | 172.28 (147.24-198.05) | 11654 (9803-13772) | 237.60 (201.66-283.94) | 1.04 (0.37 to 1.72) | 0.002 |
| Luxembourg | 1331 (1185-1491) | 264.27 (234.27-295.59) | 3013 (2613-3410) | 276.37 (240.00-313.98) | 0.20 (-0.24 to 0.65) | 0.368 |
| Madagascar | 47382 (38780-60549) | 702.76 (568.49-945.44) | 100664 (76256-129965) | 678.66 (508.16-884.33) | -0.07 (-0.39 to 0.24) | 0.645 |
| Malawi | 56194 (46192-66313) | 1003.16 (830.02-1214.13) | 110135 (88010-133712) | 1114.87 (915.06-1337.98) | 0.36 (0.18 to 0.54) | < 0.001 |
| Malaysia | 77325 (68494-86547) | 713.37 (633.73-796.59) | 238095 (207099-268811) | 817.72 (710.59-922.90) | 0.43 (0.23 to 0.62) | < 0.001 |
| Maldives | 1558 (1280-1852) | 1347.65 (1152.43-1701.12) | 2804 (2338-3335) | 719.76 (606.71-852.73) | -2.06 (-2.19 to -1.93) | < 0.001 |
| Mali | 56774 (47423-68232) | 1040.28 (873.63-1283.93) | 114837 (94129-145340) | 950.46 (780.38-1210.60) | -0.28 (-0.41 to -0.14) | < 0.001 |
| Malta | 1203 (1076-1338) | 303.04 (271.34-335.96) | 2837 (2467-3202) | 305.75 (267.12-347.23) | 0.02 (-0.29 to 0.34) | 0.895 |
| Marshall Islands | 212 (144-351) | 990.73 (669.09-1662.57) | 669 (256-1739) | 1587.83 (613.11-4196.39) | 1.53 (1.41 to 1.65) | < 0.001 |
| Mauritania | 13123 (10398-16336) | 1080.69 (859.44-1353.39) | 23187 (16553-31976) | 937.84 (665.72-1292.50) | -0.47 (-0.62 to -0.32) | < 0.001 |
| Mauritius | 10096 (9532-10683) | 1253.62 (1184.91-1323.58) | 38582 (35823-40737) | 2196.12 (2043.11-2318.87) | 1.51 (0.60 to 2.42) | 0.001 |
| Mexico | 474218 (450937-498550) | 932.17 (884.86-983.28) | 1940953 (1680240-2260842) | 1489.68 (1295.18-1728.00) | 1.52 (0.60 to 2.44) | 0.001 |
| Micronesia (Federated States of) | 595 (425-860) | 1017.49 (724.12-1495.09) | 1337 (979-1830) | 1629.46 (1205.42-2229.31) | 1.55 (1.48 to 1.62) | < 0.001 |
| Monaco | 121 (100-145) | 182.17 (150.33-216.07) | 228 (194-266) | 233.45 (198.78-273.13) | 0.81 (0.69 to 0.93) | < 0.001 |
| Mongolia | 8119 (6668-9950) | 603.16 (498.71-719.26) | 13518 (11300-16158) | 508.14 (425.37-602.36) | -0.51 (-0.95 to -0.07) | 0.024 |
| Montenegro | 2342 (1990-2734) | 382.90 (325.89-447.32) | 3844 (3139-4690) | 440.54 (361.73-537.89) | 0.56 (0.31 to 0.81) | < 0.001 |
| Morocco | 94039 (70697-146372) | 572.69 (420.85-963.46) | 289479 (223424-366427) | 861.54 (664.15-1088.94) | 1.34 (1.24 to 1.43) | < 0.001 |
| Mozambique | 59057 (48400-73912) | 681.40 (559.04-901.29) | 141035 (107847-186077) | 882.16 (684.62-1181.50) | 0.85 (0.76 to 0.94) | < 0.001 |
| Myanmar | 292701 (227943-364529) | 964.29 (759.56-1204.66) | 410628 (337703-500939) | 799.64 (662.62-966.40) | -0.60 (-0.67 to -0.54) | < 0.001 |
| Namibia | 4420 (3529-5944) | 613.58 (493.19-821.67) | 10401 (7883-13859) | 695.84 (539.88-902.08) | 0.44 (0.17 to 0.72) | 0.001 |
| Nauru | 70 (47-100) | 1204.19 (807.06-1774.85) | 127 (95-167) | 1785.00 (1311.59-2476.39) | 1.28 (1.19 to 1.38) | < 0.001 |
| Nepal | 65171 (52099-79837) | 548.64 (437.43-676.33) | 160662 (126887-204193) | 652.71 (513.55-826.05) | 0.58 (0.49 to 0.66) | < 0.001 |
| Netherlands | 37878 (32720-42847) | 202.05 (174.60-228.66) | 82167 (72158-93046) | 238.48 (208.27-270.25) | 0.51 (0.24 to 0.79) | < 0.001 |
| New Zealand | 7788 (7047-8501) | 211.30 (190.84-230.61) | 20618 (18809-22453) | 263.59 (241.22-286.59) | 0.65 (-0.08 to 1.38) | 0.08 |
| Nicaragua | 20446 (18371-26070) | 922.33 (832.85-1189.34) | 86099 (68224-102148) | 1596.85 (1282.90-1885.75) | 1.79 (1.34 to 2.23) | < 0.001 |
| Niger | 41923 (33643-50607) | 842.51 (698.06-1076.13) | 80122 (59677-120176) | 687.81 (523.37-1007.48) | -0.65 (-0.75 to -0.54) | < 0.001 |
| Nigeria | 506251 (409099-599064) | 880.97 (719.40-1053.33) | 1007285 (771422-1268598) | 824.39 (662.51-1015.24) | -0.23 (-0.30 to -0.15) | < 0.001 |
| Niue | 19 (16-25) | 880.22 (706.01-1130.78) | 32 (20-47) | 1612.30 (1011.21-2371.92) | 1.97 (1.78 to 2.16) | < 0.001 |
| North Macedonia | 6763 (5934-7869) | 365.47 (320.31-422.99) | 10889 (8783-13799) | 373.54 (303.82-461.85) | 0.11 (-0.01 to 0.22) | 0.069 |
| Northern Mariana Islands | 259 (200-334) | 1022.79 (833.17-1275.72) | 773 (644-914) | 1474.97 (1249.09-1715.80) | 1.11 (0.95 to 1.27) | < 0.001 |
| Norway | 9013 (7541-10541) | 137.17 (113.60-160.28) | 17052 (14870-19536) | 168.58 (144.00-194.09) | 0.68 (0.18 to 1.19) | 0.008 |
| Oman | 4965 (3836-6906) | 578.20 (442.15-815.39) | 19110 (15607-23751) | 878.99 (718.95-1088.52) | 1.39 (1.04 to 1.75) | < 0.001 |
| Pakistan | 404931 (332986-499986) | 590.50 (491.66-724.18) | 1213820 (972621-1508801) | 795.11 (645.52-990.21) | 0.96 (0.89 to 1.03) | < 0.001 |
| Palau | 97 (72-125) | 880.09 (657.44-1119.24) | 293 (214-385) | 1382.22 (1017.84-1796.05) | 1.45 (1.29 to 1.62) | < 0.001 |
| Palestine | 9946 (7801-12853) | 979.35 (756.41-1283.12) | 23468 (20331-27681) | 885.69 (758.84-1049.00) | -0.34 (-0.53 to -0.14) | 0.001 |
| Panama | 7457 (6800-8224) | 442.04 (401.21-486.71) | 36062 (29067-42682) | 814.72 (656.02-963.88) | 2.06 (1.60 to 2.52) | < 0.001 |
| Papua New Guinea | 11577 (8052-15381) | 451.54 (332.06-589.01) | 36547 (30525-44537) | 515.14 (430.93-644.45) | 0.45 (0.25 to 0.65) | < 0.001 |
| Paraguay | 12158 (10659-13822) | 465.64 (403.39-535.98) | 38803 (31124-48290) | 643.36 (516.02-802.81) | 1.11 (0.82 to 1.39) | < 0.001 |
| Peru | 105337 (92303-120681) | 713.69 (619.92-822.67) | 247079 (184433-311887) | 720.90 (537.44-910.56) | 0.01 (-1.05 to 1.09) | 0.982 |
| Philippines | 354857 (323905-395725) | 919.29 (837.47-1037.38) | 1136959 (958347-1307840) | 1232.68 (1049.55-1414.45) | 0.98 (0.82 to 1.13) | < 0.001 |
| Poland | 166060 (154655-176751) | 405.68 (378.08-432.19) | 136197 (117984-158787) | 206.20 (177.91-241.21) | -2.22 (-2.64 to -1.79) | < 0.001 |
| Portugal | 40610 (36847-44376) | 330.49 (300.61-360.89) | 76569 (67526-85773) | 296.47 (262.68-332.80) | -0.36 (-0.70 to -0.02) | 0.037 |
| Puerto Rico | 28212 (26770-29738) | 793.00 (753.43-835.16) | 51201 (43010-59665) | 821.94 (692.54-956.42) | 0.02 (-0.89 to 0.94) | 0.963 |
| Qatar | 1101 (745-1589) | 821.94 (512.07-1248.95) | 7653 (6095-9741) | 757.02 (614.47-939.61) | -0.24 (-0.95 to 0.48) | 0.518 |
| Republic of Korea | 130194 (117908-142679) | 429.64 (388.71-470.41) | 200278 (171976-226706) | 225.58 (193.71-255.8) | -2.05 (-2.22 to -1.87) | < 0.001 |
| Republic of Moldova | 7738 (6388-9225) | 184.45 (151.18-219.78) | 12608 (10418-14983) | 233.78 (193.33-279.17) | 0.63 (-0.26 to 1.53) | 0.168 |
| Romania | 87976 (81527-94928) | 348.63 (324.00-375.68) | 92610 (78865-107339) | 277.98 (236.88-323.94) | -0.68 (-1.07 to -0.29) | 0.001 |
| Russian Federation | 428507 (391751-466416) | 259.23 (236.91-281.99) | 464004 (409800-529294) | 213.61 (189.10-243.61) | -0.54 (-1.30 to 0.22) | 0.165 |
| Rwanda | 48445 (39539-56316) | 1255.64 (1043.57-1472.42) | 65862 (48112-87152) | 891.79 (663.55-1176.62) | -1.11 (-1.36 to -0.87) | < 0.001 |
| Saint Kitts and Nevis | 420 (392-454) | 1164.39 (1090.21-1254.49) | 914 (747-1082) | 1350.37 (1133.20-1575.83) | 0.49 (-0.13 to 1.10) | 0.119 |
| Saint Lucia | 885 (837-936) | 955.02 (904.03-1009.73) | 2376 (1996-2807) | 1041.57 (875.76-1229.97) | 0.28 (-0.34 to 0.91) | 0.372 |
| Saint Vincent and the Grenadines | 569 (534-610) | 737.17 (689.71-789.00) | 1457 (1294-1663) | 1083.48 (962.51-1234.39) | 1.16 (0.46 to 1.87) | 0.001 |
| Samoa | 868 (651-1147) | 891.33 (674.94-1163.79) | 1888 (1466-2394) | 1214.39 (948.01-1524.87) | 1.00 (0.90 to 1.11) | < 0.001 |
| San Marino | 56 (47-66) | 167.12 (138.93-195.72) | 113 (89-140) | 145.82 (113.78-182.28) | -0.52 (-0.67 to -0.37) | < 0.001 |
| Sao Tome and Principe | 1102 (882-1290) | 1348.97 (1113.57-1530.18) | 2097 (1609-2634) | 1636.60 (1240.02-1979.71) | 0.61 (0.39 to 0.84) | < 0.001 |
| Saudi Arabia | 80180 (61535-108291) | 1089.81 (832.64-1468.18) | 416093 (307761-529386) | 1762.78 (1351.11-2169.63) | 1.55 (1.36 to 1.74) | < 0.001 |
| Senegal | 49306 (41083-60783) | 1065.12 (898.19-1309.69) | 95390 (73587-131487) | 1027.56 (798.98-1391.70) | -0.21 (-0.36 to -0.05) | 0.01 |
| Serbia | 42655 (36005-50866) | 434.23 (367.69-519.71) | 58087 (48712-67910) | 374.35 (316.47-434.51) | -0.44 (-0.84 to -0.04) | 0.031 |
| Seychelles | 522 (466-589) | 886.74 (790.44-1000.76) | 1424 (1193-1643) | 1226.42 (1031.36-1409.77) | 1.07 (0.85 to 1.30) | < 0.001 |
| Sierra Leone | 24287 (18928-30087) | 832.15 (664.20-1021.91) | 39388 (29974-52547) | 770.10 (604.34-1010.78) | -0.25 (-0.39 to -0.11) | < 0.001 |
| Singapore | 8136 (7624-8708) | 355.79 (333.26-379.48) | 20705 (18435-23087) | 251.85 (223.87-280.27) | -1.11 (-1.47 to -0.75) | < 0.001 |
| Slovakia | 19699 (17271-22267) | 344.18 (302.53-388.60) | 22738 (19458-26211) | 265.28 (228.19-305.88) | -0.80 (-1.06 to -0.54) | < 0.001 |
| Slovenia | 5091 (4580-5635) | 220.21 (197.75-243.27) | 7512 (6152-9249) | 175.21 (142.33-217.91) | -0.73 (-1.02 to -0.44) | < 0.001 |
| Solomon Islands | 1408 (669-2226) | 774.73 (410.44-1191.76) | 4011 (3105-5040) | 870.92 (699.08-1073.28) | 0.39 (0.08 to 0.69) | 0.013 |
| Somalia | 42592 (31540-57931) | 1182.70 (866.99-1615.40) | 109717 (77473-161379) | 1218.61 (853.72-1741.00) | 0.11 (0.04 to 0.19) | 0.004 |
| South Africa | 155415 (136560-179958) | 616.68 (540.36-733.34) | 415624 (377739-462537) | 862.34 (784.92-953.72) | 1.09 (0.82 to 1.35) | < 0.001 |
| South Sudan | 33407 (25693-43506) | 1014.31 (768.79-1354.62) | 69366 (50852-90453) | 1345.01 (1002.09-1742.34) | 0.93 (0.78 to 1.08) | < 0.001 |
| Spain | 156194 (141120-171158) | 309.55 (280.34-339.01) | 243084 (212322-273844) | 225.37 (196.25-255.48) | -0.95 (-1.15 to -0.74) | < 0.001 |
| Sri Lanka | 91811 (79288-106066) | 768.96 (660.00-894.39) | 161739 (117194-209551) | 628.14 (460.84-813.21) | -0.64 (-1.06 to -0.22) | 0.003 |
| Sudan | 76183 (58482-110063) | 624.87 (468.41-1022.65) | 180786 (136192-237944) | 770.74 (589.96-1012.61) | 0.69 (0.61 to 0.77) | < 0.001 |
| Suriname | 2433 (2055-2721) | 831.29 (717.62-937.39) | 7429 (5840-9298) | 1181.21 (931.76-1473.05) | 1.19 (0.82 to 1.56) | < 0.001 |
| Sweden | 19881 (16791-23071) | 141.21 (119.74-164.35) | 44455 (38202-50852) | 196.33 (169.22-224.94) | 1.05 (0.78 to 1.32) | < 0.001 |
| Switzerland | 21014 (18209-23719) | 208.22 (180.63-235.20) | 44996 (38424-51285) | 228.57 (194.36-262.67) | 0.32 (0.06 to 0.58) | 0.017 |
| Syrian Arab Republic | 68209 (55197-84454) | 968.46 (772.30-1242.81) | 126862 (93695-168253) | 1020.57 (759.99-1328.87) | 0.16 (-0.14 to 0.47) | 0.295 |
| Taiwan (Province of China) | 93499 (86835-100493) | 604.47 (560.65-649.34) | 205078 (181400-224760) | 502.71 (447.01-550.52) | -0.62 (-1.07 to -0.16) | 0.008 |
| Tajikistan | 6728 (5319-8239) | 198.22 (154.91-245.31) | 15292 (12007-19273) | 217.64 (169.26-274.65) | 0.33 (0.22 to 0.43) | < 0.001 |
| Thailand | 306169 (262369-368081) | 746.17 (641.01-897.81) | 865932 (699035-1045232) | 859.06 (698.47-1033.12) | 0.44 (0.23 to 0.65) | < 0.001 |
| Timor-Leste | 3510 (2766-4402) | 805.69 (633.98-1037.57) | 8029 (6032-10955) | 851.98 (645.26-1147.67) | 0.20 (-0.02 to 0.43) | 0.078 |
| Togo | 16010 (13190-19324) | 839.21 (706.86-1015.63) | 42127 (31777-54924) | 888.19 (691.77-1154.82) | 0.19 (0.04 to 0.34) | 0.015 |
| Tokelau | 10 (7-16) | 752.82 (553.02-1180.89) | 16 (12-22) | 1136.19 (878.92-1548.38) | 1.43 (1.18 to 1.68) | < 0.001 |
| Tonga | 294 (223-388) | 481.54 (368.88-628.62) | 535 (402-689) | 639.25 (479.51-818.76) | 0.94 (0.79 to 1.09) | < 0.001 |
| Trinidad and Tobago | 6161 (5850-6493) | 681.79 (647.7-716.40) | 20236 (15316-25658) | 1101.28 (836.59-1394.70) | 1.63 (0.91 to 2.35) | < 0.001 |
| Tunisia | 28625 (23139-40343) | 534.62 (427.15-765.07) | 85581 (62643-113057) | 677.13 (500.89-889.40) | 0.75 (0.59 to 0.91) | < 0.001 |
| Turkey | 288241 (239747-364731) | 749.18 (624.47-967.68) | 552195 (453860-666416) | 619.43 (509.50-750.05) | -0.57 (-0.89 to -0.25) | 0.001 |
| Turkmenistan | 12416 (11128-13743) | 475.91 (423.17-536.5) | 33906 (27087-41471) | 730.88 (586.81-893.28) | 1.38 (0.65 to 2.11) | < 0.001 |
| Tuvalu | 65 (51-93) | 888.07 (693.67-1286.52) | 127 (95-175) | 1178.46 (885.00-1617.17) | 0.93 (0.86 to 1.00) | < 0.001 |
| Uganda | 65235 (50387-82208) | 764.55 (582.01-980.29) | 183294 (144104-239789) | 919.24 (725.47-1193.43) | 0.56 (0.40 to 0.72) | < 0.001 |
| Ukraine | 53706 (38096-69878) | 84.80 (60.75-108.71) | 111725 (89366-135575) | 171.79 (138.89-207.44) | 2.46 (1.75 to 3.17) | < 0.001 |
| United Arab Emirates | 3892 (2982-5153) | 586.60 (434.33-801.63) | 29680 (21617-37421) | 782.88 (522.73-997.84) | 0.79 (-1.18 to 2.80) | 0.436 |
| United Kingdom | 155905 (129827-180904) | 192.44 (160.59-223.04) | 258501 (216482-299277) | 213.44 (177.75-247.35) | 0.31 (0.05 to 0.58) | 0.021 |
| United Republic of Tanzania | 131082 (109800-160936) | 903.09 (755.1-1140.85) | 288849 (234235-364048) | 899.32 (731.73-1124.44) | 0.01 (-0.15 to 0.16) | 0.937 |
| United States of America | 839563 (749127-924830) | 276.06 (246.69-303.29) | 2969764 (2728422-3188334) | 543.30 (499.54-582.58) | 2.21 (1.97 to 2.44) | < 0.001 |
| United States Virgin Islands | 510 (432-606) | 563.51 (478.28-667.31) | 835 (611-1068) | 599.20 (452.26-758.01) | 0.24 (-0.02 to 0.51) | 0.074 |
| Uruguay | 13717 (12910-14498) | 364.84 (343.32-385.10) | 21441 (19615-23134) | 387.07 (357.18-417.47) | 0.17 (-0.40 to 0.75) | 0.554 |
| Uzbekistan | 44533 (35798-54486) | 315.26 (247.36-392.70) | 171630 (147004-200979) | 580.79 (501.42-677.54) | 2.01 (1.08 to 2.96) | < 0.001 |
| Vanuatu | 551 (376-866) | 696.8 (485.08-1113.23) | 2161 (1586-3205) | 1042.00 (776.42-1542.19) | 1.30 (1.13 to 1.47) | < 0.001 |
| Venezuela (Bolivarian Republic of) | 61836 (57241-66347) | 527.63 (484.65-568.04) | 324201 (249903-407035) | 1087.01 (843.33-1362.67) | 2.43 (1.80 to 3.06) | < 0.001 |
| Viet Nam | 305955 (223778-380082) | 671.53 (506.42-832.43) | 676321 (501845-849831) | 685.86 (514.20-847.41) | 0.07 (0.03 to 0.11) | < 0.001 |
| Yemen | 33670 (24098-53572) | 521.10 (367.11-927.38) | 82899 (60825-131865) | 509.97 (372.62-812.52) | -0.06 (-0.20 to 0.09) | 0.441 |
| Zambia | 49375 (39792-57931) | 1179.33 (995.88-1379.63) | 123798 (84564-188445) | 1289.74 (932.63-1817.44) | 0.33 (0.17 to 0.49) | < 0.001 |
| Zimbabwe | 30248 (24565-39272) | 663.96 (538.02-846.97) | 89465 (68552-116945) | 1084.27 (848.29-1401.87) | 1.73 (1.32 to 2.13) | < 0.001 |

Abbreviations: DALYs, disability-adjusted life-years.

**Table S16.** The number and age-standardized rate of prevalence for CKD-associated anemia in 1990 and 2021, and the temporal trends between 1990-2021 in 204 countries and territories.

| Location | Number of cases, 1990 | Age-standardized rate per 100,000 population, 1990 | Number of cases, 2021 | Age-standardized rate per 100,000 population, 2021 | Average annual percentage change, 1990-2021 | p value |
| --- | --- | --- | --- | --- | --- | --- |
| Afghanistan | 58926 (48916-72184) | 890.69 (747.01-1091.71) | 89992 (75923-107028) | 855.95 (694.88-1077.84) | -0.13 (-0.17 to -0.08) | < 0.001 |
| Albania | 20542 (17385-24362) | 882.46 (752.13-1051.74) | 29339 (24681-35364) | 755.96 (651.20-885.97) | -0.49 (-0.54 to -0.45) | < 0.001 |
| Algeria | 128831 (106232-152371) | 941.32 (776.51-1115.92) | 289978 (240037-347889) | 829.90 (684.25-990.78) | -0.40 (-0.42 to -0.39) | < 0.001 |
| American Samoa | 296 (249-354) | 1283.51 (1065.25-1527.43) | 674 (556-809) | 1463.32 (1224.33-1751.16) | 0.42 (0.40 to 0.44) | < 0.001 |
| Andorra | 221 (179-272) | 461.96 (373.00-564.36) | 652 (512-802) | 374.38 (302.50-460.66) | -0.67 (-0.70 to -0.65) | < 0.001 |
| Angola | 61793 (51839-72466) | 1439.83 (1228.14-1713.47) | 192281 (159401-229551) | 1446.57 (1194.90-1731.82) | 0.02 (-0.04 to 0.07) | 0.565 |
| Antigua and Barbuda | 489 (395-595) | 877.07 (708.48-1075.85) | 847 (697-1050) | 843.35 (701.31-1031.43) | -0.13 (-0.17 to -0.09) | < 0.001 |
| Argentina | 154599 (131073-184276) | 499.14 (426.35-594.31) | 234796 (195067-281470) | 419.64 (350.49-504.61) | -0.57 (-0.61 to -0.53) | < 0.001 |
| Armenia | 34381 (28598-41141) | 1312.68 (1092.07-1551.49) | 54574 (45958-66438) | 1301.93 (1103.18-1566.28) | -0.03 (-0.04 to -0.01) | < 0.001 |
| Australia | 91680 (75686-114550) | 496.25 (410.65-617.28) | 212176 (166957-269463) | 413.78 (331.21-523.91) | -0.61 (-0.72 to -0.49) | < 0.001 |
| Austria | 55384 (45462-68601) | 476.98 (398.86-578.52) | 88909 (71993-113007) | 417.41 (342.71-520.86) | -0.42 (-0.46 to -0.38) | < 0.001 |
| Azerbaijan | 79643 (67643-95788) | 1595.43 (1354.94-1954.43) | 171384 (142066-211457) | 1759.88 (1469.43-2127.35) | 0.32 (0.29 to 0.34) | < 0.001 |
| Bahamas | 1431 (1179-1722) | 879.65 (714.55-1063.70) | 3243 (2662-3911) | 830.76 (688.29-999.37) | -0.19 (-0.23 to -0.15) | < 0.001 |
| Bahrain | 2157 (1830-2542) | 997.72 (814.17-1201.19) | 7368 (6037-8998) | 851.19 (683.97-1046.32) | -0.51 (-0.53 to -0.48) | < 0.001 |
| Bangladesh | 443232 (381227-505768) | 840.75 (716.25-964.04) | 1287122 (1112099-1529999) | 967.98 (832.79-1144.93) | 0.45 (0.42 to 0.49) | < 0.001 |
| Barbados | 2173 (1801-2607) | 738.37 (623.65-872.15) | 3529 (2888-4378) | 722.77 (599.37-876.53) | -0.07 (-0.08 to -0.06) | < 0.001 |
| Belarus | 104077 (87029-129560) | 843.20 (705.90-1035.89) | 119957 (97444-147842) | 766.32 (631.49-930.96) | -0.31 (-0.34 to -0.28) | < 0.001 |
| Belgium | 73164 (60486-90033) | 485.04 (405.75-587.54) | 108736 (87449-135239) | 386.12 (318.29-467.51) | -0.72 (-0.75 to -0.70) | < 0.001 |
| Belize | 976 (831-1162) | 942.45 (785.58-1143.23) | 3116 (2489-3730) | 999.15 (804.11-1207.42) | 0.18 (0.17 to 0.20) | < 0.001 |
| Benin | 17317 (15373-19652) | 786.80 (688.44-910.31) | 61491 (53526-71657) | 987.17 (853.35-1148.11) | 0.73 (0.72 to 0.75) | < 0.001 |
| Bermuda | 377 (308-455) | 637.98 (521.02-763.37) | 612 (460-769) | 451.55 (351.57-557.41) | -1.11 (-1.15 to -1.06) | < 0.001 |
| Bhutan | 2529 (2174-2900) | 946.15 (811.96-1096.83) | 6875 (5881-8054) | 1091.32 (925.62-1283.58) | 0.46 (0.44 to 0.47) | < 0.001 |
| Bolivia (Plurinational State of) | 28572 (24279-32985) | 804.62 (677.81-935.23) | 60420 (49519-73667) | 683.75 (559.95-828.23) | -0.52 (-0.54 to -0.50) | < 0.001 |
| Bosnia and Herzegovina | 26531 (21748-32655) | 667.35 (561.10-793.18) | 35396 (29498-42588) | 636.97 (535.22-754.46) | -0.15 (-0.19 to -0.10) | < 0.001 |
| Botswana | 7370 (6194-8822) | 1278.50 (1077.61-1530.54) | 19199 (15980-22916) | 1277.9 (1071.88-1526.36) | 0.01 (-0.01 to 0.04) | 0.339 |
| Brazil | 949134 (801929-1109541) | 1089.82 (920.72-1273.01) | 2054503 (1732410-2452075) | 837.13 (707.77-994.98) | -0.84 (-0.91 to -0.77) | < 0.001 |
| Brunei Darussalam | 868 (772-990) | 793.13 (703.48-908.73) | 2341 (1960-2851) | 762.11 (637.80-939.00) | -0.13 (-0.15 to -0.10) | < 0.001 |
| Bulgaria | 66527 (54317-81541) | 669.29 (557.95-799.41) | 88686 (73344-107096) | 747.94 (623.01-902.52) | 0.36 (0.31 to 0.41) | < 0.001 |
| Burkina Faso | 45347 (38258-53450) | 1018.66 (857.44-1198.48) | 114723 (96847-133008) | 1120.48 (938.95-1320.87) | 0.31 (0.28 to 0.34) | < 0.001 |
| Burundi | 15489 (13298-18574) | 598.22 (508.97-728.35) | 29385 (24782-34458) | 529.40 (442.33-637.59) | -0.39 (-0.46 to -0.33) | < 0.001 |
| Cabo Verde | 2146 (1800-2563) | 907.24 (758.78-1088.45) | 4142 (3443-4898) | 887.28 (732.80-1050.66) | -0.07 (-0.08 to -0.06) | < 0.001 |
| Cambodia | 46709 (39183-56274) | 1011.54 (839.14-1226.87) | 114044 (94682-138587) | 988.20 (817.54-1194.20) | -0.08 (-0.11 to -0.04) | < 0.001 |
| Cameroon | 57590 (49426-66806) | 1237.26 (1059.06-1455.74) | 215847 (183596-251129) | 1523.65 (1287.94-1789.21) | 0.67 (0.61 to 0.73) | < 0.001 |
| Canada | 120905 (98397-148218) | 377.26 (309.53-460.17) | 189903 (153982-240884) | 274.56 (224.78-340.89) | -1.02 (-1.06 to -0.97) | < 0.001 |
| Central African Republic | 18059 (14781-21495) | 1509.33 (1241.49-1814.71) | 36303 (30698-42915) | 1497.14 (1261.30-1761.41) | -0.03 (-0.05 to -0.01) | 0.005 |
| Chad | 27984 (23856-32366) | 949.01 (807.17-1109.73) | 68433 (58237-80032) | 1013.95 (853.72-1192.63) | 0.22 (0.20 to 0.24) | < 0.001 |
| Chile | 44908 (37970-54488) | 457.68 (386.86-556.43) | 91330 (74031-114096) | 355.07 (289.00-442.90) | -0.81 (-0.85 to -0.77) | < 0.001 |
| China | 5645085 (5170014-6161351) | 713.99 (653.40-779.90) | 8057990 (7313368-8886242) | 422.68 (386.50-464.60) | -1.67 (-1.74 to -1.60) | < 0.001 |
| Colombia | 111026 (95362-129844) | 597.00 (509.15-706.46) | 233951 (196391-281664) | 416.29 (349.29-505.64) | -1.16 (-1.17 to -1.14) | < 0.001 |
| Comoros | 1456 (1222-1693) | 628.22 (533.84-728.73) | 2963 (2470-3518) | 573.31 (480.02-680.85) | -0.29 (-0.33 to -0.26) | < 0.001 |
| Congo | 16174 (13515-19403) | 1453.28 (1221.12-1740.79) | 44037 (36630-53233) | 1499.86 (1241.39-1789.61) | 0.10 (0.07 to 0.13) | < 0.001 |
| Cook Islands | 135 (113-163) | 1116.13 (920.44-1357.66) | 290 (232-359) | 1147.65 (929.32-1399.28) | 0.09 (0.06 to 0.12) | < 0.001 |
| Costa Rica | 16149 (13685-18955) | 850.91 (712.59-1005.87) | 36559 (30407-43780) | 670.09 (556.08-812.30) | -0.74 (-0.78 to -0.70) | < 0.001 |
| Coted'Ivoire | 53062 (46067-61732) | 1090.85 (926.36-1294.11) | 139557 (117765-165030) | 1099.69 (907.19-1308.61) | 0.04 (-0.01 to 0.09) | 0.161 |
| Croatia | 30765 (25742-36759) | 561.28 (473.67-663.52) | 44437 (36099-54254) | 528.03 (438.64-642.97) | -0.20 (-0.24 to -0.16) | < 0.001 |
| Cuba | 65305 (53172-79678) | 649.65 (531.02-789.56) | 110734 (88430-135237) | 569.00 (462.48-695.95) | -0.42 (-0.45 to -0.40) | < 0.001 |
| Cyprus | 4603 (3723-5626) | 658.93 (549.50-791.19) | 8809 (7000-11024) | 459.57 (374.87-559.35) | -1.15 (-1.18 to -1.12) | < 0.001 |
| Czechia | 72080 (60614-86182) | 594.99 (498.75-716.22) | 98759 (80209-119731) | 500.60 (410.98-611.63) | -0.56 (-0.59 to -0.52) | < 0.001 |
| Democratic People's Republic of Korea | 116589 (99345-137643) | 739.83 (635.13-869.36) | 206009 (174586-246560) | 669.51 (574.07-789.92) | -0.32 (-0.34 to -0.31) | < 0.001 |
| Democratic Republic of the Congo | 275350 (226854-330271) | 1627.25 (1362.56-1925.66) | 627972 (531800-736232) | 1564.49 (1305.57-1840.03) | -0.13 (-0.16 to -0.09) | < 0.001 |
| Denmark | 42902 (34845-52203) | 512.29 (420.41-614.53) | 57400 (46845-71719) | 437.37 (359.86-539.36) | -0.50 (-0.52 to -0.49) | < 0.001 |
| Djibouti | 1071 (900-1277) | 583.61 (491.58-689.07) | 4163 (3429-5223) | 567.14 (473.14-696.56) | -0.10 (-0.15 to -0.04) | < 0.001 |
| Dominica | 590 (483-709) | 989.99 (815.12-1181.85) | 817 (660-991) | 1020.54 (834.72-1223.17) | 0.10 (0.07 to 0.12) | < 0.001 |
| Dominican Republic | 32346 (26693-39351) | 841.69 (692.99-1040.24) | 82430 (66106-101004) | 819.61 (654.94-1001.67) | -0.09 (-0.11 to -0.07) | < 0.001 |
| Ecuador | 24790 (20824-28954) | 434.46 (359.09-517.33) | 56489 (46741-69596) | 349.67 (288.95-431.52) | -0.71 (-0.74 to -0.67) | < 0.001 |
| Egypt | 299600 (250935-351033) | 1007.56 (833.35-1205.01) | 618360 (510166-766240) | 959.82 (785.67-1173.02) | -0.15 (-0.17 to -0.14) | < 0.001 |
| El Salvador | 14287 (12341-16755) | 455.35 (390.92-536.13) | 27796 (23170-34048) | 432.19 (358.64-531.97) | -0.17 (-0.21 to -0.12) | < 0.001 |
| Equatorial Guinea | 2877 (2404-3414) | 1421.22 (1201.05-1691.51) | 9006 (7589-10698) | 1490.48 (1242.83-1792.25) | 0.16 (0.10 to 0.21) | < 0.001 |
| Eritrea | 8853 (7490-10394) | 618.6 (519.30-733.55) | 19128 (16029-22704) | 587.01 (489.24-706.46) | -0.16 (-0.23 to -0.09) | < 0.001 |
| Estonia | 17097 (13991-21156) | 880.08 (725.48-1072.13) | 24354 (19581-30654) | 854.41 (697.04-1039.21) | -0.08 (-0.13 to -0.04) | < 0.001 |
| Eswatini | 3769 (3208-4463) | 1242.71 (1044.22-1468.55) | 6974 (5864-8315) | 1242.26 (1042.37-1486.22) | 0.03 (-0.02 to 0.08) | 0.208 |
| Ethiopia | 117308 (105865-130456) | 555.83 (493.45-624.66) | 206071 (184185-227455) | 451.20 (400.37-507.85) | -0.67 (-0.69 to -0.66) | < 0.001 |
| Fiji | 4421 (3853-5078) | 1163.94 (1001.69-1345.08) | 11434 (9584-13506) | 1519.83 (1276.77-1788.05) | 0.86 (0.81 to 0.92) | < 0.001 |
| Finland | 27901 (23186-33500) | 406.66 (338.82-485.70) | 48805 (38821-60517) | 319.35 (262.95-387.03) | -0.76 (-0.79 to -0.74) | < 0.001 |
| France | 276097 (220781-339892) | 322.57 (263.90-391.71) | 538033 (426115-690106) | 290.97 (238.01-362.81) | -0.35 (-0.38 to -0.31) | < 0.001 |
| Gabon | 8061 (6769-9694) | 1401.37 (1172.26-1688.58) | 16357 (13738-18977) | 1498.11 (1245.19-1764.33) | 0.21 (0.18 to 0.25) | < 0.001 |
| Gambia | 3522 (3005-4164) | 901.30 (767.73-1059.92) | 10427 (8720-12438) | 946.02 (787.07-1133.49) | 0.16 (0.14 to 0.17) | < 0.001 |
| Georgia | 80713 (65986-98638) | 1376.88 (1145.27-1661.79) | 83473 (67730-102582) | 1397.41 (1157.44-1701.02) | 0.05 (0.03 to 0.06) | < 0.001 |
| Germany | 684736 (562228-814078) | 560.76 (468.01-659.00) | 986751 (805086-1236184) | 456.65 (383.62-551.04) | -0.65 (-0.69 to -0.61) | < 0.001 |
| Ghana | 41592 (34400-48749) | 602.27 (493.19-708.87) | 121764 (100140-142715) | 647.15 (536.03-773.59) | 0.24 (0.21 to 0.26) | < 0.001 |
| Greece | 73523 (62093-86323) | 526.92 (450.19-617.58) | 131412 (105603-162107) | 457.83 (383.12-553.70) | -0.44 (-0.48 to -0.41) | < 0.001 |
| Greenland | 164 (135-198) | 525.67 (438.95-641.90) | 271 (217-348) | 462.16 (374.18-580.19) | -0.42 (-0.52 to -0.32) | < 0.001 |
| Grenada | 695 (585-818) | 914.68 (769.70-1077.15) | 1030 (822-1275) | 949.12 (758.11-1164.09) | 0.11 (0.10 to 0.13) | < 0.001 |
| Guam | 796 (646-972) | 1086.28 (893.14-1330.11) | 2559 (2066-3096) | 1242.90 (991.76-1488.55) | 0.43 (0.37 to 0.48) | < 0.001 |
| Guatemala | 28480 (24732-32971) | 747.19 (644.41-867.19) | 87127 (74084-101599) | 766.73 (648.32-904.92) | 0.08 (0.06 to 0.10) | < 0.001 |
| Guinea | 33176 (27924-38851) | 977.08 (822.69-1150.97) | 66615 (56715-77262) | 1054.80 (887.20-1237.72) | 0.26 (0.23 to 0.29) | < 0.001 |
| Guinea-Bissau | 4646 (3938-5453) | 1047.46 (880.31-1254.82) | 9421 (7918-11235) | 1074.49 (901.84-1303.45) | 0.09 (0.08 to 0.10) | < 0.001 |
| Guyana | 4715 (4051-5446) | 1083.84 (923.93-1274.38) | 6910 (5689-8324) | 1084.64 (895.67-1300.02) | 0.00 (-0.04 to 0.04) | 0.92 |
| Haiti | 28441 (24183-33993) | 896.42 (749.75-1068.94) | 74189 (62103-88621) | 999.60 (833.42-1191.21) | 0.35 (0.33 to 0.37) | < 0.001 |
| Honduras | 16961 (14624-19664) | 762.19 (648.53-897.12) | 47045 (40111-56159) | 740.64 (625.81-886.67) | -0.09 (-0.10 to -0.07) | < 0.001 |
| Hungary | 58401 (50123-70202) | 452.91 (388.30-540.68) | 71424 (61183-84064) | 413.26 (350.02-493.14) | -0.29 (-0.32 to -0.26) | < 0.001 |
| Iceland | 1026 (830-1246) | 335.94 (273.26-406.02) | 1753 (1392-2186) | 255.29 (210.36-314.62) | -0.87 (-0.94 to -0.80) | < 0.001 |
| India | 5719581 (5239569-6244507) | 1205.00 (1107.60-1322.76) | 13182656 (12140711-14325056) | 1108.91 (1019.64-1206.99) | -0.26 (-0.30 to -0.23) | < 0.001 |
| Indonesia | 1357287 (1211162-1516905) | 1354.45 (1196.72-1525.36) | 2903824 (2581487-3274575) | 1295.55 (1145.08-1476.68) | -0.14 (-0.23 to -0.04) | 0.004 |
| Iran (Islamic Republic of) | 349009 (296732-407935) | 1306.81 (1121.45-1519.71) | 770198 (660221-905261) | 1034.59 (875.52-1231.02) | -0.74 (-0.80 to -0.68) | < 0.001 |
| Iraq | 79019 (67929-91000) | 823.56 (711.28-961.49) | 172077 (144313-204047) | 670.65 (565.80-805.29) | -0.66 (-0.69 to -0.63) | < 0.001 |
| Ireland | 26206 (20663-33351) | 671.77 (535.10-829.36) | 38206 (30964-46128) | 464.06 (382.84-554.17) | -1.19 (-1.23 to -1.15) | < 0.001 |
| Israel | 31525 (25466-38676) | 683.50 (559.24-829.93) | 71004 (58227-86097) | 537.36 (441.44-644.06) | -0.77 (-0.79 to -0.76) | < 0.001 |
| Italy | 377351 (327523-432736) | 455.25 (399.01-518.71) | 535154 (436944-644098) | 311.84 (263.20-369.01) | -1.22 (-1.26 to -1.18) | < 0.001 |
| Jamaica | 16001 (13247-19211) | 858.57 (708.64-1035.26) | 26666 (21949-32297) | 832.73 (679.84-1011.18) | -0.10 (-0.13 to -0.07) | < 0.001 |
| Japan | 1431258 (1223085-1661520) | 886.61 (765.97-1028.05) | 3250568 (2659571-3886118) | 734.20 (603.69-879.98) | -0.61 (-0.65 to -0.57) | < 0.001 |
| Jordan | 12601 (10706-14930) | 740.35 (632.58-873.56) | 50998 (43823-61359) | 628.10 (539.25-755.25) | -0.53 (-0.57 to -0.50) | < 0.001 |
| Kazakhstan | 199284 (161897-240544) | 1583.05 (1295.79-1888.72) | 255971 (208477-311648) | 1518.23 (1257.09-1842.71) | -0.14 (-0.15 to -0.13) | < 0.001 |
| Kenya | 40040 (36770-43815) | 460.77 (419.51-511.15) | 102742 (94500-112401) | 449.23 (411.00-491.45) | -0.09 (-0.12 to -0.06) | < 0.001 |
| Kiribati | 495 (424-577) | 1252.66 (1050.23-1484.87) | 1026 (854-1221) | 1359.33 (1116.96-1614.36) | 0.26 (0.24 to 0.28) | < 0.001 |
| Kuwait | 5691 (5037-6437) | 657.68 (572.06-764.04) | 16238 (13652-19100) | 506.56 (432.95-619.24) | -0.85 (-0.89 to -0.81) | < 0.001 |
| Kyrgyzstan | 50140 (41566-59729) | 1636.81 (1352.55-1959.55) | 71058 (59857-84198) | 1475.90 (1244.19-1752.72) | -0.33 (-0.34 to -0.32) | < 0.001 |
| Lao People's Democratic Republic | 26136 (21905-30750) | 1225.38 (1015.52-1440.32) | 56353 (47046-67497) | 1212.79 (1013.98-1443.09) | -0.03 (-0.06 to 0.00) | 0.023 |
| Latvia | 29199 (23816-36382) | 849.99 (700.81-1038.26) | 34864 (28045-43809) | 849.62 (698.46-1049.25) | 0.00 (-0.01 to 0.02) | 0.855 |
| Lebanon | 14735 (12615-17908) | 684.29 (585.19-820.03) | 32829 (27790-39320) | 530.17 (448.51-633.40) | -0.82 (-0.84 to -0.80) | < 0.001 |
| Lesotho | 9701 (8209-11842) | 1170.68 (990.46-1445.33) | 12514 (10530-15285) | 1209.37 (1014.91-1480.63) | 0.16 (0.11 to 0.21) | < 0.001 |
| Liberia | 10857 (9102-12784) | 893.55 (754.26-1061.88) | 23539 (19841-28861) | 939.03 (794.85-1124.71) | 0.16 (0.15 to 0.18) | < 0.001 |
| Libya | 15909 (13604-18596) | 717.48 (600.91-861.99) | 34467 (29123-42090) | 624.81 (525.76-754.36) | -0.44 (-0.46 to -0.42) | < 0.001 |
| Lithuania | 37696 (30700-46144) | 853.49 (706.40-1034.86) | 49148 (39105-60981) | 824.73 (674.35-1006.91) | -0.11 (-0.12 to -0.09) | < 0.001 |
| Luxembourg | 2755 (2216-3451) | 536.82 (438.99-656.07) | 4666 (3659-5726) | 396.50 (315.38-477.20) | -0.97 (-1.00 to -0.95) | < 0.001 |
| Madagascar | 28986 (24386-33804) | 512.83 (425.93-605.85) | 65929 (55558-78474) | 517.21 (433.71-625.34) | 0.02 (0.00 to 0.04) | 0.026 |
| Malawi | 26871 (23106-32076) | 613.25 (523.76-731.64) | 58641 (49652-67819) | 638.66 (529.30-754.26) | 0.14 (0.09 to 0.18) | < 0.001 |
| Malaysia | 93249 (83384-104303) | 968.68 (858.93-1098.80) | 326970 (271538-393666) | 1208.08 (1001.01-1456.34) | 0.73 (0.69 to 0.78) | < 0.001 |
| Maldives | 1078 (893-1304) | 1218.34 (1002.08-1469.52) | 3294 (2721-3908) | 1045.34 (836.74-1274.99) | -0.49 (-0.52 to -0.47) | < 0.001 |
| Mali | 44613 (38103-53529) | 1040.51 (895.47-1238.76) | 117887 (98122-137904) | 1136.44 (964.27-1339.87) | 0.29 (0.27 to 0.30) | < 0.001 |
| Malta | 2300 (1870-2854) | 589.07 (479.47-724.08) | 4874 (3917-6074) | 456.81 (375.57-565.03) | -0.81 (-0.86 to -0.77) | < 0.001 |
| Marshall Islands | 205 (174-245) | 1151.20 (953.75-1385.53) | 443 (364-530) | 1273.10 (1057.23-1522.55) | 0.32 (0.31 to 0.34) | < 0.001 |
| Mauritania | 11174 (9497-13081) | 1040.71 (887.79-1209.91) | 24164 (19924-29308) | 1033.32 (861.00-1241.92) | -0.02 (-0.03 to -0.01) | 0.001 |
| Mauritius | 9507 (7674-11495) | 1321.30 (1067.59-1581.82) | 26266 (21625-31785) | 1499.83 (1258.75-1791.20) | 0.42 (0.39 to 0.44) | < 0.001 |
| Mexico | 301589 (279340-330425) | 739.99 (681.18-811.10) | 891775 (818690-976313) | 742.06 (677.41-813.05) | 0.01 (-0.02 to 0.03) | 0.515 |
| Micronesia (Federated States of) | 696 (585-820) | 1315.25 (1098.63-1567.76) | 1125 (934-1337) | 1516.28 (1263.61-1803.86) | 0.46 (0.43 to 0.49) | < 0.001 |
| Monaco | 285 (229-359) | 379.26 (309.60-466.48) | 401 (310-521) | 337.23 (274.77-420.35) | -0.38 (-0.39 to -0.37) | < 0.001 |
| Mongolia | 15704 (13817-18205) | 1441.88 (1237.27-1705.44) | 27107 (23241-31521) | 1283.14 (1091.83-1506.27) | -0.38 (-0.39 to -0.36) | < 0.001 |
| Montenegro | 3900 (3266-4669) | 637.24 (534.81-755.31) | 5240 (4324-6511) | 634.19 (532.34-759.12) | -0.01 (-0.04 to 0.02) | 0.357 |
| Morocco | 93131 (80332-110442) | 579.28 (498.67-683.22) | 168297 (138325-202626) | 498.14 (413.11-592.20) | -0.48 (-0.49 to -0.46) | < 0.001 |
| Mozambique | 28819 (24820-33622) | 458.38 (392.92-544.02) | 58035 (49299-67409) | 457.92 (378.84-549.60) | -0.01 (-0.12 to 0.11) | 0.929 |
| Myanmar | 266070 (222707-316830) | 1135.89 (945.20-1362.80) | 519784 (426507-612352) | 1121.82 (917.91-1332.02) | -0.01 (-0.07 to 0.04) | 0.658 |
| Namibia | 9500 (8072-11011) | 1394.21 (1197.65-1624.24) | 16022 (13642-18802) | 1162.84 (984.07-1349.33) | -0.57 (-0.59 to -0.55) | < 0.001 |
| Nauru | 66 (55-78) | 1331.02 (1121.11-1600.57) | 90 (75-107) | 1465.80 (1217.43-1756.07) | 0.31 (0.28 to 0.34) | < 0.001 |
| Nepal | 189394 (158303-224471) | 1832.39 (1541.71-2160.72) | 546232 (453993-669574) | 2298.92 (1904.13-2765.01) | 0.73 (0.67 to 0.79) | < 0.001 |
| Netherlands | 87226 (72717-105755) | 440.40 (367.40-527.00) | 140146 (112994-171641) | 371.51 (305.00-445.53) | -0.55 (-0.60 to -0.50) | < 0.001 |
| New Zealand | 17639 (15123-20403) | 475.91 (406.90-551.05) | 37779 (31285-45827) | 418.26 (351.11-502.94) | -0.44 (-0.49 to -0.40) | < 0.001 |
| Nicaragua | 15914 (13913-18629) | 939.49 (806.16-1113.64) | 49290 (41752-58148) | 1016.32 (855.69-1209.13) | 0.25 (0.23 to 0.27) | < 0.001 |
| Niger | 32146 (26847-37941) | 1009.48 (840.85-1212.68) | 94104 (78644-109967) | 1021.88 (846.05-1204.26) | 0.04 (0.02 to 0.06) | < 0.001 |
| Nigeria | 782690 (677604-909794) | 1660.90 (1421.82-1930.33) | 1769453 (1539190-1987103) | 1687.90 (1469.66-1904.23) | 0.05 (0.03 to 0.08) | < 0.001 |
| Niue | 29 (24-35) | 1280.41 (1061.00-1532.11) | 29 (24-35) | 1390.13 (1158.23-1653.27) | 0.26 (0.22 to 0.29) | < 0.001 |
| North Macedonia | 15453 (12993-18693) | 844.19 (715.34-1012.25) | 22735 (18915-27591) | 829.88 (702.40-984.42) | -0.07 (-0.12 to -0.03) | 0.002 |
| Northern Mariana Islands | 306 (252-371) | 1483.74 (1231.52-1788.62) | 732 (590-903) | 1509.23 (1244.96-1805.82) | 0.06 (0.03 to 0.09) | < 0.001 |
| Norway | 26766 (22191-32363) | 370.00 (308.82-439.21) | 36293 (29947-43786) | 313.96 (260.39-376.65) | -0.52 (-0.58 to -0.46) | < 0.001 |
| Oman | 9183 (7802-10864) | 1126.33 (907.66-1358.62) | 20136 (16634-24507) | 882.04 (712.01-1112.22) | -0.79 (-0.82 to -0.76) | < 0.001 |
| Pakistan | 569669 (499261-660746) | 972.56 (848.22-1131.25) | 1633816 (1430198-1872955) | 1203.47 (1055.16-1394.71) | 0.66 (0.62 to 0.70) | < 0.001 |
| Palau | 136 (114-160) | 1351.51 (1130.50-1600.69) | 309 (249-379) | 1466.18 (1215.65-1760.32) | 0.25 (0.23 to 0.28) | < 0.001 |
| Palestine | 9170 (7825-10665) | 888.49 (758.23-1050.79) | 20109 (17064-24029) | 697.46 (591.34-831.45) | -0.78 (-0.79 to -0.76) | < 0.001 |
| Panama | 11771 (10225-13594) | 744.48 (636.32-863.15) | 30670 (25935-35865) | 683.21 (577.54-800.12) | -0.27 (-0.29 to -0.26) | < 0.001 |
| Papua New Guinea | 18657 (15898-21796) | 988.27 (837.20-1164.88) | 52591 (44624-61902) | 995.17 (838.74-1176.56) | 0.02 (-0.002 to 0.04) | 0.082 |
| Paraguay | 21203 (17889-25224) | 943.65 (785.53-1135.82) | 46123 (39304-55245) | 801.91 (674.64-971.81) | -0.52 (-0.54 to -0.49) | < 0.001 |
| Peru | 79092 (68823-91579) | 570.38 (489.12-670.19) | 126022 (107881-146443) | 367.54 (312.34-429.23) | -1.41 (-1.46 to -1.36) | < 0.001 |
| Philippines | 352568 (309274-402186) | 1162.59 (1017.00-1337.70) | 678417 (597102-770949) | 860.25 (756.53-981.38) | -0.97 (-1.02 to -0.92) | < 0.001 |
| Poland | 312580 (265313-362612) | 782.92 (675.38-902.27) | 411439 (344844-485228) | 617.91 (525.66-725.46) | -0.77 (-0.80 to -0.74) | < 0.001 |
| Portugal | 56155 (46085-68626) | 459.82 (382.59-548.92) | 88373 (69082-111230) | 309.83 (251.00-378.98) | -1.27 (-1.32 to -1.23) | < 0.001 |
| Puerto Rico | 27249 (22034-34015) | 772.88 (629.32-965.24) | 46018 (35466-57711) | 622.29 (492.50-783.28) | -0.70 (-0.73 to -0.68) | < 0.001 |
| Qatar | 1567 (1285-1919) | 956.27 (778.58-1155.70) | 8388 (6978-10485) | 813.73 (643.58-1024.27) | -0.51 (-0.56 to -0.46) | < 0.001 |
| Republic of Korea | 245246 (210038-284652) | 936.31 (779.10-1086.91) | 376465 (288157-460334) | 418.42 (317.74-512.50) | -2.57 (-2.61 to -2.53) | < 0.001 |
| Republic of Moldova | 51872 (42536-64009) | 1284.10 (1059.50-1562.91) | 74372 (61177-90651) | 1272.25 (1056.82-1545.98) | -0.04 (-0.07 to 0.00) | 0.024 |
| Romania | 183843 (151346-223463) | 750.75 (624.47-887.40) | 253928 (213032-308626) | 751.99 (634.85-923.90) | 0.00 (-0.03 to 0.04) | 0.798 |
| Russian Federation | 1309372 (1072347-1602765) | 801.47 (664.92-967.75) | 1573187 (1317357-1904227) | 681.31 (575.41-813.42) | -0.52 (-0.55 to -0.50) | < 0.001 |
| Rwanda | 18631 (15780-22470) | 592.71 (501.33-719.62) | 33816 (27814-40772) | 516.51 (414.67-625.70) | -0.44 (-0.49 to -0.40) | < 0.001 |
| Saint Kitts and Nevis | 389 (321-474) | 1058.67 (889.11-1281.67) | 599 (478-745) | 914.58 (736.03-1126.28) | -0.48 (-0.54 to -0.43) | < 0.001 |
| Saint Lucia | 864 (718-1032) | 959.59 (791.84-1145.87) | 2098 (1711-2556) | 903.61 (741.65-1092.21) | -0.20 (-0.23 to -0.17) | < 0.001 |
| Saint Vincent and the Grenadines | 668 (556-801) | 908.23 (749.81-1091.95) | 1263 (1031-1555) | 930.36 (771.74-1135.98) | 0.07 (0.03 to 0.12) | 0.002 |
| Samoa | 918 (767-1105) | 1088.84 (902.51-1311.78) | 1603 (1335-1981) | 1155.5 (955.38-1428.40) | 0.19 (0.17 to 0.22) | < 0.001 |
| San Marino | 137 (110-167) | 378.10 (308.40-458.35) | 344 (260-435) | 343.56 (277.07-418.73) | -0.30 (-0.33 to -0.28) | < 0.001 |
| Sao Tome and Principe | 836 (703-982) | 1223.42 (1014.31-1439.85) | 1649 (1376-1955) | 1274.48 (1051.25-1520.37) | 0.13 (0.12 to 0.15) | < 0.001 |
| Saudi Arabia | 68023 (58018-79226) | 1012.86 (837.50-1222.33) | 197667 (161692-235175) | 938.20 (755.04-1143.85) | -0.25 (-0.26 to -0.23) | < 0.001 |
| Senegal | 27292 (23378-31653) | 770.43 (654.14-902.59) | 67084 (57837-78171) | 775.63 (661.74-905.93) | 0.03 (-0.01 to 0.06) | 0.161 |
| Serbia | 50073 (41175-62200) | 492.55 (410.35-593.94) | 65486 (53203-81162) | 430.29 (352.90-532.60) | -0.42 (-0.47 to -0.37) | < 0.001 |
| Seychelles | 689 (568-817) | 1191.53 (978.91-1416.47) | 1344 (1097-1654) | 1235.75 (999.40-1506.39) | 0.13 (0.12 to 0.13) | < 0.001 |
| Sierra Leone | 21283 (18298-24998) | 967.63 (827.17-1149.78) | 44310 (38116-51573) | 1036.37 (878.61-1230.97) | 0.22 (0.21 to 0.23) | < 0.001 |
| Singapore | 18432 (15304-21885) | 869.54 (716.26-1047.53) | 43940 (35089-57661) | 540.12 (431.34-706.09) | -1.50 (-1.55 to -1.45) | < 0.001 |
| Slovakia | 36141 (30331-42565) | 662.47 (560.06-786.40) | 49407 (40558-60212) | 580.23 (483.86-701.61) | -0.42 (-0.45 to -0.39) | < 0.001 |
| Slovenia | 12062 (10083-14595) | 522.92 (438.11-621.68) | 20183 (16035-25551) | 442.41 (361.86-548.47) | -0.54 (-0.58 to -0.49) | < 0.001 |
| Solomon Islands | 1885 (1586-2236) | 1234.12 (1050.49-1455.48) | 4913 (4055-5822) | 1295.61 (1072.66-1522.05) | 0.16 (0.15 to 0.17) | < 0.001 |
| Somalia | 19869 (16698-23477) | 618.38 (525.04-723.64) | 51066 (43693-59222) | 615.51 (520.49-729.18) | -0.01 (-0.06 to 0.04) | 0.759 |
| South Africa | 243861 (208920-284035) | 1145.24 (972.59-1348.63) | 486820 (431886-553515) | 1089.08 (963.50-1236.36) | -0.15 (-0.20 to -0.09) | < 0.001 |
| South Sudan | 17440 (14782-20667) | 590.69 (499.96-709.55) | 26303 (22075-30701) | 572.92 (483.34-677.35) | -0.10 (-0.16 to -0.04) | 0.001 |
| Spain | 309114 (249206-369083) | 590.31 (479.07-704.80) | 467780 (362779-595040) | 386.13 (309.95-476.83) | -1.35 (-1.41 to -1.29) | < 0.001 |
| Sri Lanka | 127884 (107265-149951) | 1195.90 (988.82-1412.34) | 274071 (217783-342934) | 1071.31 (865.45-1319.76) | -0.35 (-0.37 to -0.33) | < 0.001 |
| Sudan | 87061 (71189-104827) | 867.40 (712.72-1042.82) | 174390 (145625-206225) | 808.06 (674.71-964.77) | -0.23 (-0.24 to -0.21) | < 0.001 |
| Suriname | 2657 (2228-3198) | 998.36 (824.98-1198.60) | 6217 (5126-7514) | 1008.41 (840.93-1213.33) | 0.03 (0.01 to 0.04) | < 0.001 |
| Sweden | 58910 (48340-74024) | 375.84 (314.30-463.79) | 81834 (66067-102277) | 311.41 (258.06-380.23) | -0.60 (-0.63 to -0.57) | < 0.001 |
| Switzerland | 59094 (49861-71107) | 567.18 (482.76-677.12) | 95829 (77137-120169) | 464.50 (390.08-563.58) | -0.63 (-0.66 to -0.60) | < 0.001 |
| Syrian Arab Republic | 60796 (51672-71234) | 986.57 (834.23-1171.28) | 97370 (80814-118377) | 797.52 (666.08-970.03) | -0.68 (-0.74 to -0.61) | < 0.001 |
| Taiwan (Province of China) | 117565 (98429-142559) | 818.44 (694.18-980.15) | 274579 (230669-337553) | 646.15 (541.45-787.11) | -0.76 (-0.83 to -0.68) | < 0.001 |
| Tajikistan | 35546 (29926-41856) | 1266.27 (1062.29-1502.19) | 75033 (63852-89132) | 1330.72 (1109.32-1572.42) | 0.16 (0.14 to 0.17) | < 0.001 |
| Thailand | 508199 (434075-592000) | 1527.44 (1302.60-1808.25) | 1715726 (1387505-2056540) | 1607.78 (1297.76-1918.89) | 0.17 (0.11 to 0.22) | < 0.001 |
| Timor-Leste | 3443 (2892-4112) | 1137.34 (942.77-1364.65) | 9371 (7602-11113) | 1118.13 (926.80-1321.97) | -0.05 (-0.09 to -0.01) | 0.019 |
| Togo | 15057 (12776-17670) | 1021.89 (851.17-1215.39) | 47634 (39837-55741) | 1097.46 (922.67-1311.14) | 0.23 (0.22 to 0.25) | < 0.001 |
| Tokelau | 16 (13-18) | 1199.94 (984.57-1408.50) | 19 (15-23) | 1269.68 (1029.06-1526.54) | 0.18 (0.16 to 0.21) | < 0.001 |
| Tonga | 677 (550-803) | 1219.27 (998.75-1428.94) | 1047 (863-1238) | 1294.91 (1064.78-1545.76) | 0.19 (0.16 to 0.22) | < 0.001 |
| Trinidad and Tobago | 7746 (6376-9399) | 911.39 (749.59-1110.88) | 16206 (13085-20028) | 883.87 (726.62-1074.47) | -0.11 (-0.13 to -0.08) | < 0.001 |
| Tunisia | 30698 (26622-35907) | 605.95 (527.83-709.28) | 71319 (59158-85074) | 571.30 (478.91-677.45) | -0.18 (-0.21 to -0.15) | < 0.001 |
| Turkey | 297790 (258334-346485) | 778.82 (673.76-902.34) | 553692 (473933-652767) | 619.69 (528.58-728.95) | -0.72 (-0.74 to -0.69) | < 0.001 |
| Turkmenistan | 29128 (24965-33947) | 1482.75 (1259.98-1746.10) | 60164 (50427-72596) | 1534.96 (1286.38-1857.24) | 0.11 (0.10 to 0.13) | < 0.001 |
| Tuvalu | 80 (67-97) | 1238.67 (1038.02-1474.63) | 136 (114-162) | 1337.23 (1134.55-1591.84) | 0.25 (0.23 to 0.26) | < 0.001 |
| Uganda | 43413 (36838-50778) | 602.48 (502.54-712.99) | 100896 (86707-118102) | 585.27 (494.77-700.22) | -0.10 (-0.11 to -0.08) | < 0.001 |
| Ukraine | 479930 (397236-600466) | 745.23 (616.24-917.06) | 577411 (476768-713237) | 761.20 (632.96-934.43) | 0.07 (0.04 to 0.10) | < 0.001 |
| United Arab Emirates | 9768 (8191-11858) | 1290.71 (1067.34-1561.65) | 59631 (46548-78534) | 941.35 (770.23-1150.33) | -1.01 (-1.06 to -0.96) | < 0.001 |
| United Kingdom | 495894 (415912-595000) | 557.37 (473.16-653.25) | 616841 (515475-731462) | 441.78 (372.13-520.13) | -0.75 (-0.78 to -0.72) | < 0.001 |
| United Republic of Tanzania | 62830 (53352-73915) | 505.06 (428.67-593.74) | 161090 (136475-186203) | 521.20 (437.51-613.42) | 0.08 (0.05 to 0.11) | < 0.001 |
| United States of America | 2014362 (1815307-2249758) | 616.66 (558.19-686.32) | 4349268 (3694305-5177287) | 729.43 (619.87-861.63) | 0.54 (0.49 to 0.59) | < 0.001 |
| United States Virgin Islands | 656 (525-808) | 813.81 (657.46-997.24) | 1295 (1045-1623) | 769.35 (637.05-924.69) | -0.18 (-0.21 to -0.15) | < 0.001 |
| Uruguay | 19539 (16001-24103) | 524.12 (432.23-635.33) | 25043 (20390-31233) | 428.25 (352.08-538.43) | -0.66 (-0.69 to -0.62) | < 0.001 |
| Uzbekistan | 228549 (192360-278051) | 1820.95 (1529.03-2222.88) | 481418 (398055-589799) | 1834.86 (1542.38-2205.31) | 0.02 (0.00 to 0.03) | 0.019 |
| Vanuatu | 781 (664-924) | 1175.51 (983.31-1403.48) | 2430 (2041-2858) | 1336.44 (1122.25-1580.89) | 0.42 (0.40 to 0.43) | < 0.001 |
| Venezuela (Bolivarian Republic of) | 72338 (61962-83586) | 713.87 (602.03-840.55) | 196762 (166985-233529) | 684.43 (579.85-815.68) | -0.13 (-0.17 to -0.08) | < 0.001 |
| Viet Nam | 276650 (232220-332031) | 683.78 (571.05-823.88) | 480821 (397959-584406) | 534.65 (439.97-653.74) | -0.77 (-0.85 to -0.69) | < 0.001 |
| Yemen | 32981 (27391-39113) | 700.65 (566.78-839.30) | 93178 (79480-111114) | 637.61 (532.26-767.42) | -0.3 (-0.33 to -0.26) | < 0.001 |
| Zambia | 20258 (17075-23465) | 610.98 (504.83-723.87) | 60493 (50392-70280) | 704.34 (582.36-833.44) | 0.46 (0.41 to 0.51) | < 0.001 |
| Zimbabwe | 55126 (46156-65752) | 1276.44 (1076.38-1502.79) | 85334 (71255-100877) | 1215.38 (1014.43-1450) | -0.14 (-0.17 to -0.10) | < 0.001 |

**Table S17.** The number and age-standardized rate of YLDs for CKD-associated anemia in 1990 and 2021, and the temporal trends between 1990-2021 in 204 countries and territories.

| Location | Number of cases, 1990 | Age-standardized rate per 100,000 population, 1990 | Number of cases, 2021 | Age-standardized rate per 100,000 population, 2021 | Average annual percentage change, 1990-2021 | p value |
| --- | --- | --- | --- | --- | --- | --- |
| Afghanistan | 1971 (1279-2878) | 30.59 (20.20-44.34) | 2730 (1775-3972) | 27.08 (17.58-38.88) | -0.39 (-0.41 to -0.37) | < 0.001 |
| Albania | 487 (304-715) | 21.87 (13.68-32.19) | 574 (356-875) | 14.81 (9.28-22.75) | -1.26 (-1.31 to -1.21) | < 0.001 |
| Algeria | 3482 (2299-5105) | 26.01 (16.89-37.95) | 7137 (4639-10628) | 20.47 (13.45-30.46) | -0.76 (-0.80 to -0.73) | < 0.001 |
| American Samoa | 7 (4-10) | 33.43 (21.81-50.05) | 16 (10-24) | 36.96 (23.41-55.92) | 0.33 (0.30 to 0.35) | < 0.001 |
| Andorra | 3 (2-5) | 7.32 (4.45-11.43) | 10 (6-16) | 5.70 (3.37-9.26) | -0.81 (-0.84 to -0.77) | < 0.001 |
| Angola | 2000 (1276-2896) | 48.38 (31.50-68.77) | 4995 (3207-7365) | 39.05 (24.98-56.89) | -0.69 (-0.74 to -0.64) | < 0.001 |
| Antigua and Barbuda | 9 (6-14) | 16.63 (10.64-25.39) | 14 (8-22) | 14.54 (8.78-22.21) | -0.44 (-0.48 to -0.39) | < 0.001 |
| Argentina | 2659 (1683-4084) | 9.09 (5.81-13.89) | 4238 (2649-6683) | 7.38 (4.62-11.64) | -0.69 (-0.73 to -0.64) | < 0.001 |
| Armenia | 904 (580-1354) | 34.25 (22.12-50.94) | 1228 (758-1835) | 29.51 (18.47-44.34) | -0.48 (-0.51 to -0.46) | < 0.001 |
| Australia | 1570 (987-2347) | 8.65 (5.48-12.73) | 3515 (2120-5478) | 6.77 (4.12-10.43) | -0.81 (-0.93 to -0.70) | < 0.001 |
| Austria | 959 (576-1479) | 8.22 (4.92-12.64) | 1428 (856-2231) | 6.55 (3.96-10.25) | -0.72 (-0.80 to -0.63) | < 0.001 |
| Azerbaijan | 2335 (1492-3497) | 45.13 (28.99-66.32) | 4099 (2606-6134) | 40.54 (25.76-60.93) | -0.33 (-0.46 to -0.21) | < 0.001 |
| Bahamas | 27 (17-43) | 16.95 (10.57-26.91) | 57 (36-88) | 14.98 (9.42-23.07) | -0.41 (-0.45 to -0.37) | < 0.001 |
| Bahrain | 54 (34-80) | 25.90 (16.46-38.41) | 158 (99-238) | 18.91 (11.75-28.92) | -1.00 (-1.03 to -0.97) | < 0.001 |
| Bangladesh | 21896 (14977-31110) | 44.21 (30.27-61.28) | 46056 (30587-66970) | 35.48 (23.77-51.75) | -0.72 (-0.76 to -0.67) | < 0.001 |
| Barbados | 40 (24-62) | 14.41 (8.93-21.98) | 62 (39-96) | 13.16 (8.31-19.90) | -0.29 (-0.32 to -0.25) | < 0.001 |
| Belarus | 2342 (1459-3503) | 19.17 (12.02-28.49) | 2387 (1466-3650) | 15.39 (9.53-23.48) | -0.70 (-0.74 to -0.66) | < 0.001 |
| Belgium | 1337 (806-1986) | 8.88 (5.43-13.26) | 1855 (1115-2846) | 6.47 (3.91-9.77) | -1.01 (-1.06 to -0.95) | < 0.001 |
| Belize | 22 (14-32) | 20.75 (13.35-30.32) | 63 (39-94) | 20.07 (12.26-29.87) | -0.10 (-0.13 to -0.06) | < 0.001 |
| Benin | 840 (573-1156) | 38.29 (26.12-52.84) | 2312 (1519-3291) | 38.16 (24.80-53.74) | -0.01 (-0.04 to 0.02) | 0.647 |
| Bermuda | 6 (4-10) | 10.60 (6.46-16.49) | 9 (5-14) | 6.61 (3.88-10.95) | -1.51 (-1.55 to -1.46) | < 0.001 |
| Bhutan | 137 (93-193) | 52.06 (35.95-72.12) | 278 (183-392) | 45.74 (30.17-64.17) | -0.42 (-0.44 to -0.40) | < 0.001 |
| Bolivia (Plurinational State of) | 698 (454-1022) | 20.35 (13.25-30.08) | 1227 (754-1885) | 14.41 (8.90-21.84) | -1.11 (-1.14 to -1.08) | < 0.001 |
| Bosnia and Herzegovina | 639 (404-947) | 16.87 (11.01-24.72) | 750 (469-1112) | 13.32 (8.35-19.76) | -0.76 (-0.87 to -0.66) | < 0.001 |
| Botswana | 206 (133-302) | 35.84 (23.21-52.47) | 479 (300-711) | 31.86 (20.31-47.05) | -0.35 (-0.41 to -0.28) | < 0.001 |
| Brazil | 22272 (14301-32853) | 25.45 (16.37-37.96) | 43683 (27178-67561) | 17.82 (11.08-27.60) | -1.14 (-1.18 to -1.09) | < 0.001 |
| Brunei Darussalam | 16 (10-24) | 17.09 (10.73-25.00) | 40 (25-63) | 14.73 (9.22-22.05) | -0.48 (-0.50 to -0.45) | < 0.001 |
| Bulgaria | 1568 (971-2383) | 16.15 (10.08-23.58) | 1934 (1230-2904) | 15.79 (9.88-23.41) | -0.07 (-0.15 to 0.01) | 0.092 |
| Burkina Faso | 1658 (1078-2429) | 37.57 (24.63-54.20) | 3803 (2520-5672) | 37.53 (25.34-55.42) | 0.00 (-0.03 to 0.02) | 0.808 |
| Burundi | 444 (284-671) | 18.47 (12.04-26.97) | 788 (510-1174) | 15.22 (10.08-22.54) | -0.62 (-0.65 to -0.58) | < 0.001 |
| Cabo Verde | 64 (41-95) | 27.22 (17.58-40.72) | 102 (65-150) | 21.88 (13.97-32.20) | -0.70 (-0.73 to -0.67) | < 0.001 |
| Cambodia | 1683 (1087-2379) | 37.57 (24.49-53.57) | 3188 (2015-4737) | 28.91 (18.42-43.06) | -0.85 (-0.88 to -0.82) | < 0.001 |
| Cameroon | 1915 (1262-2736) | 41.69 (27.86-59.20) | 5951 (3937-8901) | 43.80 (28.90-64.97) | 0.16 (0.06 to 0.25) | 0.002 |
| Canada | 1742 (1051-2723) | 5.41 (3.28-8.46) | 2773 (1628-4436) | 3.77 (2.21-6.12) | -1.16 (-1.24 to -1.08) | < 0.001 |
| Central African Republic | 564 (372-820) | 49.30 (32.37-70.35) | 1084 (702-1597) | 46.99 (30.71-69.42) | -0.15 (-0.19 to -0.11) | < 0.001 |
| Chad | 1068 (706-1521) | 36.31 (24.12-51.52) | 2342 (1539-3512) | 34.72 (23.05-51.36) | -0.14 (-0.17 to -0.10) | < 0.001 |
| Chile | 822 (511-1228) | 9.29 (5.91-13.51) | 1689 (1056-2594) | 6.49 (4.06-9.99) | -1.17 (-1.21 to -1.13) | < 0.001 |
| China | 169970 (112856-240346) | 23.89 (15.99-34.01) | 191114 (124978-274103) | 10.39 (6.79-14.81) | -2.64 (-2.70 to -2.59) | < 0.001 |
| Colombia | 2479 (1600-3661) | 14.27 (9.26-21.14) | 4793 (2911-7234) | 8.56 (5.16-12.96) | -1.64 (-1.72 to -1.57) | < 0.001 |
| Comoros | 43 (27-63) | 19.21 (12.61-27.58) | 75 (47-114) | 15.34 (9.81-22.99) | -0.72 (-0.77 to -0.67) | < 0.001 |
| Congo | 595 (391-845) | 54.08 (35.44-76.24) | 1288 (833-1926) | 44.22 (28.18-65.22) | -0.64 (-0.70 to -0.59) | < 0.001 |
| Cook Islands | 3 (2-4) | 27.34 (17.40-39.63) | 6 (4-9) | 24.07 (15.06-35.79) | -0.41 (-0.48 to -0.35) | < 0.001 |
| Costa Rica | 307 (193-460) | 17.07 (10.74-25.31) | 674 (414-1032) | 12.34 (7.63-18.90) | -1.03 (-1.12 to -0.95) | < 0.001 |
| Coted'Ivoire | 1883 (1265-2720) | 38.47 (25.38-54.85) | 4268 (2792-6192) | 33.76 (22.31-48.93) | -0.42 (-0.45 to -0.39) | < 0.001 |
| Croatia | 676 (437-1028) | 12.64 (8.15-19.34) | 876 (560-1314) | 10.33 (6.57-15.38) | -0.65 (-0.70 to -0.59) | < 0.001 |
| Cuba | 1147 (698-1787) | 12.08 (7.44-18.86) | 1951 (1120-3099) | 9.92 (5.87-15.47) | -0.63 (-0.67 to -0.59) | < 0.001 |
| Cyprus | 76 (46-118) | 11.98 (7.56-17.94) | 141 (85-217) | 7.36 (4.43-11.22) | -1.56 (-1.65 to -1.47) | < 0.001 |
| Czechia | 1611 (1028-2443) | 13.25 (8.43-19.95) | 1954 (1192-3074) | 9.56 (5.87-14.98) | -1.04 (-1.09 to -0.99) | < 0.001 |
| Democratic People's Republic of Korea | 3307 (2121-4892) | 23.55 (15.26-34.45) | 5584 (3635-8097) | 18.97 (12.22-27.68) | -0.70 (-0.72 to -0.67) | < 0.001 |
| Democratic Republic of the Congo | 10436 (6909-14994) | 61.90 (41.08-88.01) | 18713 (12016-27358) | 47.95 (31.11-70.16) | -0.82 (-0.86 to -0.79) | < 0.001 |
| Denmark | 705 (429-1124) | 8.37 (5.13-13.22) | 872 (516-1372) | 6.55 (3.87-10.38) | -0.79 (-0.84 to -0.74) | < 0.001 |
| Djibouti | 28 (18-43) | 16.56 (10.80-24.78) | 92 (57-138) | 13.91 (8.76-20.57) | -0.55 (-0.59 to -0.52) | < 0.001 |
| Dominica | 12 (8-18) | 21.25 (13.62-31.57) | 16 (9-23) | 19.92 (12.20-29.69) | -0.21 (-0.24 to -0.19) | < 0.001 |
| Dominican Republic | 716 (458-1082) | 19.16 (12.31-28.72) | 1567 (991-2425) | 15.63 (9.98-24.24) | -0.66 (-0.70 to -0.61) | < 0.001 |
| Ecuador | 457 (284-707) | 8.31 (5.10-12.90) | 960 (600-1529) | 6.02 (3.79-9.53) | -1.02 (-1.08 to -0.96) | < 0.001 |
| Egypt | 8020 (5186-11815) | 28.56 (18.68-40.92) | 13741 (8857-19803) | 22.84 (14.99-33.07) | -0.71 (-0.75 to -0.67) | < 0.001 |
| El Salvador | 367 (237-541) | 12.04 (7.80-17.96) | 592 (377-921) | 9.10 (5.80-14.20) | -0.89 (-0.95 to -0.83) | < 0.001 |
| Equatorial Guinea | 111 (73-159) | 55.69 (36.95-80.23) | 231 (148-343) | 38.63 (25.08-56.94) | -1.19 (-1.24 to -1.13) | < 0.001 |
| Eritrea | 275 (174-410) | 20.03 (12.75-29.62) | 490 (314-739) | 16.42 (10.51-23.98) | -0.63 (-0.67 to -0.59) | < 0.001 |
| Estonia | 378 (240-560) | 19.81 (12.73-29.55) | 450 (270-697) | 16.31 (10.06-24.89) | -0.61 (-0.65 to -0.57) | < 0.001 |
| Eswatini | 109 (70-158) | 35.77 (23.40-51.26) | 192 (123-280) | 34.10 (21.92-49.6) | -0.11 (-0.17 to -0.05) | < 0.001 |
| Ethiopia | 3702 (2466-5386) | 17.53 (11.60-25.51) | 5037 (3290-7348) | 11.05 (7.15-16.01) | -1.48 (-1.51 to -1.45) | < 0.001 |
| Fiji | 114 (73-168) | 34.01 (22.00-49.28) | 261 (166-396) | 39.19 (25.3-59.28) | 0.46 (0.42 to 0.49) | < 0.001 |
| Finland | 492 (317-743) | 7.18 (4.62-10.82) | 793 (478-1268) | 5.13 (3.16-8.20) | -1.08 (-1.14 to -1.01) | < 0.001 |
| France | 5119 (3067-7861) | 6.01 (3.57-9.25) | 9078 (5359-14494) | 4.86 (2.90-7.70) | -0.70 (-0.76 to -0.64) | < 0.001 |
| Gabon | 320 (215-452) | 55.45 (37.54-77.80) | 528 (351-763) | 47.36 (31.35-68.54) | -0.51 (-0.54 to -0.49) | < 0.001 |
| Gambia | 135 (90-194) | 33.82 (22.86-48.16) | 356 (234-523) | 32.05 (20.79-46.85) | -0.16 (-0.19 to -0.14) | < 0.001 |
| Georgia | 2131 (1353-3179) | 36.07 (23.02-53.54) | 2039 (1268-3055) | 34.49 (21.58-51.59) | -0.15 (-0.17 to -0.12) | < 0.001 |
| Germany | 11426 (7101-17198) | 9.12 (5.73-13.98) | 15538 (9323-24271) | 6.80 (4.14-10.70) | -0.93 (-0.97 to -0.90) | < 0.001 |
| Ghana | 1562 (1029-2250) | 22.84 (15.04-32.33) | 3834 (2445-5665) | 21.02 (13.38-30.65) | -0.26 (-0.28 to -0.23) | < 0.001 |
| Greece | 1320 (831-1966) | 9.54 (6.01-14.24) | 2388 (1487-3805) | 7.94 (4.91-12.39) | -0.58 (-0.63 to -0.53) | < 0.001 |
| Greenland | 3 (2-4) | 9.98 (6.16-15.48) | 4 (3-7) | 7.74 (4.73-11.78) | -0.82 (-0.91 to -0.73) | < 0.001 |
| Grenada | 16 (10-24) | 20.91 (13.34-31.22) | 19 (12-29) | 17.63 (10.96-27.01) | -0.56 (-0.60 to -0.52) | < 0.001 |
| Guam | 16 (10-25) | 24.21 (15.35-37.23) | 59 (37-90) | 26.69 (16.77-40.89) | 0.31 (0.21 to 0.41) | < 0.001 |
| Guatemala | 882 (578-1272) | 24.04 (15.74-34.73) | 2289 (1463-3430) | 20.23 (12.82-30.59) | -0.56 (-0.58 to -0.53) | < 0.001 |
| Guinea | 1277 (855-1819) | 38.08 (25.57-53.75) | 2207 (1448-3213) | 35.90 (23.5-51.53) | -0.18 (-0.22 to -0.15) | < 0.001 |
| Guinea-Bissau | 189 (128-276) | 42.23 (28.12-62.48) | 323 (207-477) | 37.27 (23.83-54.40) | -0.40 (-0.43 to -0.37) | < 0.001 |
| Guyana | 115 (74-170) | 28.02 (18.30-40.72) | 157 (99-243) | 25.13 (16.10-38.28) | -0.36 (-0.40 to -0.32) | < 0.001 |
| Haiti | 1085 (726-1551) | 33.51 (22.61-48.51) | 2202 (1438-3243) | 30.42 (20.27-44.60) | -0.31 (-0.33 to -0.30) | < 0.001 |
| Honduras | 450 (294-656) | 21.58 (14.05-31.17) | 1127 (721-1619) | 18.77 (12.14-26.91) | -0.45 (-0.47 to -0.42) | < 0.001 |
| Hungary | 1530 (996-2252) | 11.67 (7.53-17.34) | 1673 (1069-2477) | 9.17 (5.87-13.60) | -0.77 (-0.81 to -0.73) | < 0.001 |
| Iceland | 17 (10-27) | 5.56 (3.40-8.73) | 27 (16-44) | 3.95 (2.33-6.24) | -1.06 (-1.17 to -0.95) | < 0.001 |
| India | 275432 (187432-381724) | 61.31 (41.90-84.69) | 522879 (355089-728557) | 46.36 (31.64-64.59) | -0.89 (-0.92 to -0.86) | < 0.001 |
| Indonesia | 33795 (21596-49070) | 37.41 (23.92-54.00) | 62090 (39041-93535) | 30.25 (19.36-44.62) | -0.65 (-0.73 to -0.56) | < 0.001 |
| Iran (Islamic Republic of) | 8796 (5679-12712) | 33.06 (21.27-46.93) | 17953 (11538-26031) | 23.87 (15.32-34.47) | -1.03 (-1.09 to -0.97) | < 0.001 |
| Iraq | 2177 (1447-3219) | 22.58 (14.86-32.98) | 4088 (2615-6088) | 15.99 (10.22-23.97) | -1.11 (-1.17 to -1.06) | < 0.001 |
| Ireland | 432 (263-650) | 11.36 (6.98-17.02) | 553 (327-859) | 6.65 (3.97-10.38) | -1.7 (-1.76 to -1.64) | < 0.001 |
| Israel | 549 (326-842) | 12.39 (7.5-19.02) | 1204 (738-1886) | 8.89 (5.43-14.04) | -1.06 (-1.09 to -1.03) | < 0.001 |
| Italy | 6272 (3765-9547) | 7.50 (4.58-11.27) | 8630 (5357-13264) | 4.82 (2.98-7.41) | -1.41 (-1.44 to -1.38) | < 0.001 |
| Jamaica | 316 (195-486) | 16.94 (10.52-26.23) | 527 (326-788) | 15.38 (9.31-23.12) | -0.31 (-0.33 to -0.29) | < 0.001 |
| Japan | 20728 (12505-32285) | 13.58 (8.33-20.93) | 56784 (34786-84861) | 10.95 (6.54-16.92) | -0.69 (-0.75 to -0.64) | < 0.001 |
| Jordan | 380 (252-554) | 23.02 (15.08-33.12) | 1410 (924-2118) | 17.22 (11.26-25.93) | -0.93 (-0.97 to -0.88) | < 0.001 |
| Kazakhstan | 6377 (4193-9382) | 49.75 (32.77-72.34) | 6729 (4307-9896) | 38.54 (24.71-56.24) | -0.82 (-0.88 to -0.76) | < 0.001 |
| Kenya | 1128 (745-1620) | 13.84 (9.14-19.64) | 2666 (1757-3862) | 12.55 (8.30-18.03) | -0.31 (-0.33 to -0.30) | < 0.001 |
| Kiribati | 16 (11-24) | 44.49 (29.11-65.45) | 30 (19-44) | 43.92 (28.21-64.21) | -0.05 (-0.07 to -0.03) | < 0.001 |
| Kuwait | 119 (75-178) | 13.58 (8.64-20.61) | 348 (216-519) | 10.14 (6.33-15.04) | -0.94 (-1.04 to -0.84) | < 0.001 |
| Kyrgyzstan | 1567 (1025-2242) | 50.64 (33.26-72.35) | 1944 (1270-2883) | 38.84 (25.4-57.49) | -0.85 (-0.89 to -0.82) | < 0.001 |
| Lao People's Democratic Republic | 870 (586-1250) | 44.01 (29.64-63.32) | 1509 (960-2222) | 34.84 (22.36-51.54) | -0.75 (-0.77 to -0.73) | < 0.001 |
| Latvia | 643 (412-956) | 19.06 (12.29-28.29) | 676 (403-1023) | 16.95 (10.33-25.72) | -0.38 (-0.39 to -0.37) | < 0.001 |
| Lebanon | 400 (260-573) | 18.50 (12.03-26.56) | 787 (504-1147) | 12.80 (8.19-18.71) | -1.18 (-1.20 to -1.16) | < 0.001 |
| Lesotho | 281 (182-416) | 33.61 (21.57-50.44) | 354 (227-514) | 34.07 (21.55-49.57) | 0.10 (0.04 to 0.16) | 0.001 |
| Liberia | 492 (322-698) | 39.32 (25.91-55.52) | 946 (622-1348) | 35.34 (22.94-49.42) | -0.35 (-0.39 to -0.30) | < 0.001 |
| Libya | 390 (244-583) | 17.57 (11.10-25.57) | 846 (545-1280) | 15.41 (9.89-22.91) | -0.43 (-0.51 to -0.35) | < 0.001 |
| Lithuania | 829 (503-1301) | 19.12 (11.60-29.72) | 944 (587-1448) | 16.28 (10.11-24.82) | -0.51 (-0.54 to -0.49) | < 0.001 |
| Luxembourg | 46 (28-71) | 9.02 (5.45-13.99) | 71 (42-111) | 5.92 (3.51-9.28) | -1.35 (-1.40 to -1.31) | < 0.001 |
| Madagascar | 1024 (680-1463) | 18.68 (12.46-26.74) | 1932 (1269-2897) | 16.25 (10.74-23.83) | -0.45 (-0.48 to -0.42) | < 0.001 |
| Malawi | 1081 (723-1503) | 23.78 (15.77-33.69) | 1614 (1005-2408) | 18.66 (11.92-27.30) | -0.77 (-0.83 to -0.72) | < 0.001 |
| Malaysia | 2838 (1892-4234) | 30.54 (20.42-45.61) | 7572 (4767-11272) | 29.04 (18.28-42.80) | -0.15 (-0.20 to -0.09) | < 0.001 |
| Maldives | 34 (22-49) | 38.17 (24.51-54.20) | 83 (55-121) | 24.97 (16.19-36.20) | -1.36 (-1.40 to -1.32) | < 0.001 |
| Mali | 1955 (1308-2801) | 44.87 (30.08-64.37) | 4439 (3010-6197) | 42.15 (28.15-59.64) | -0.20 (-0.21 to -0.18) | < 0.001 |
| Malta | 40 (25-62) | 10.67 (6.70-16.21) | 81 (50-121) | 7.41 (4.57-11.08) | -1.17 (-1.22 to -1.12) | < 0.001 |
| Marshall Islands | 6 (4-9) | 36.96 (24.27-52.22) | 11 (7-17) | 37.77 (24.67-55.39) | 0.07 (0.05 to 0.10) | < 0.001 |
| Mauritania | 407 (264-586) | 38.12 (25.33-54.93) | 680 (439-1015) | 29.22 (18.97-43.59) | -0.85 (-0.87 to -0.83) | < 0.001 |
| Mauritius | 237 (154-356) | 34.71 (22.70-52.41) | 608 (383-921) | 35.18 (22.45-53.61) | 0.06 (0.02 to 0.09) | 0.001 |
| Mexico | 6037 (3946-8930) | 14.87 (9.75-21.80) | 16352 (10459-24204) | 13.63 (8.76-20.06) | -0.28 (-0.32 to -0.25) | < 0.001 |
| Micronesia (Federated States of) | 21 (14-30) | 42.21 (27.25-60.79) | 28 (18-42) | 42.99 (27.53-62.95) | 0.06 (0.01 to 0.10) | 0.009 |
| Monaco | 4 (2-6) | 5.27 (3.07-8.38) | 5 (3-9) | 4.46 (2.68-7.32) | -0.54 (-0.59 to -0.49) | < 0.001 |
| Mongolia | 595 (403-838) | 52.06 (34.96-74.01) | 828 (542-1193) | 36.25 (23.93-52.49) | -1.17 (-1.28 to -1.07) | < 0.001 |
| Montenegro | 84 (55-126) | 13.99 (9.10-20.91) | 107 (69-164) | 13.00 (8.40-20.07) | -0.24 (-0.28 to -0.20) | < 0.001 |
| Morocco | 2718 (1755-4007) | 17.08 (11.10-25.01) | 4365 (2825-6308) | 13.04 (8.40-18.84) | -0.86 (-0.92 to -0.81) | < 0.001 |
| Mozambique | 1260 (854-1783) | 19.49 (13.17-27.95) | 2373 (1596-3379) | 17.99 (12.07-25.67) | -0.25 (-0.30 to -0.20) | < 0.001 |
| Myanmar | 8904 (5995-12888) | 40.01 (26.95-58.03) | 14718 (9749-21618) | 32.79 (21.92-48.05) | -0.62 (-0.66 to -0.57) | < 0.001 |
| Namibia | 272 (170-395) | 39.75 (25.04-58.45) | 378 (237-562) | 27.93 (17.58-40.69) | -1.11 (-1.2 to -1.02) | < 0.001 |
| Nauru | 2 (1-3) | 39.45 (25.35-59.38) | 2 (1-3) | 40.52 (25.79-59.72) | 0.09 (0.04 to 0.13) | < 0.001 |
| Nepal | 9232 (6260-13063) | 91.19 (61.78-127.13) | 19588 (12474-28743) | 84.28 (54.64-123.86) | -0.25 (-0.35 to -0.15) | < 0.001 |
| Netherlands | 1512 (919-2316) | 7.63 (4.67-11.64) | 2238 (1340-3479) | 5.83 (3.54-9.00) | -0.86 (-0.90 to -0.83) | < 0.001 |
| New Zealand | 336 (206-504) | 9.24 (5.79-13.93) | 658 (389-1024) | 7.24 (4.31-11.29) | -0.79 (-0.84 to -0.74) | < 0.001 |
| Nicaragua | 399 (257-597) | 24.79 (16.08-37.36) | 1106 (696-1634) | 23.39 (14.70-34.43) | -0.19 (-0.24 to -0.14) | < 0.001 |
| Niger | 1191 (793-1753) | 37.28 (24.55-53.76) | 2960 (1957-4430) | 33.43 (21.89-49.37) | -0.35 (-0.36 to -0.33) | < 0.001 |
| Nigeria | 28633 (19190-40985) | 60.78 (40.58-86.83) | 59367 (39111-85442) | 56.22 (36.82-80.30) | -0.25 (-0.27 to -0.24) | < 0.001 |
| Niue | 1 (1-1) | 35.2 (22.27-51.62) | 1 (0-1) | 34.73 (22.29-50.50) | -0.05 (-0.08 to -0.03) | < 0.001 |
| North Macedonia | 412 (272-620) | 22.72 (15.13-33.85) | 519 (335-764) | 18.87 (12.13-27.29) | -0.59 (-0.64 to -0.54) | < 0.001 |
| Northern Mariana Islands | 6 (4-9) | 32.55 (19.81-49.28) | 14 (9-23) | 33.14 (20.75-51.44) | 0.06 (0.03 to 0.10) | 0.001 |
| Norway | 435 (262-680) | 5.92 (3.59-9.04) | 549 (323-870) | 4.63 (2.71-7.33) | -0.79 (-0.83 to -0.74) | < 0.001 |
| Oman | 182 (114-272) | 22.36 (13.96-33.37) | 335 (207-503) | 15.30 (9.36-23.59) | -1.21 (-1.25 to -1.17) | < 0.001 |
| Pakistan | 28381 (19153-39585) | 48.39 (32.40-67.41) | 66500 (44813-94204) | 50.51 (34.21-72.53) | 0.10 (0.06 to 0.14) | < 0.001 |
| Palau | 3 (2-5) | 35.70 (22.61-51.83) | 7 (4-10) | 35.37 (22.57-53.36) | -0.04 (-0.08 to 0.00) | 0.04 |
| Palestine | 232 (151-339) | 23.27 (15.04-33.38) | 441 (281-666) | 16.00 (10.16-24.07) | -1.2 (-1.23 to -1.16) | < 0.001 |
| Panama | 251 (158-374) | 16.69 (10.55-24.58) | 616 (381-943) | 13.70 (8.49-21.06) | -0.64 (-0.70 to -0.58) | < 0.001 |
| Papua New Guinea | 543 (348-800) | 31.89 (21.41-45.45) | 1385 (896-2144) | 29.83 (19.47-44.90) | -0.22 (-0.24 to -0.19) | < 0.001 |
| Paraguay | 430 (272-647) | 19.06 (12.15-28.53) | 899 (560-1399) | 15.71 (9.76-24.3) | -0.61 (-0.65 to -0.57) | < 0.001 |
| Peru | 2043 (1266-3003) | 14.70 (9.21-21.40) | 2691 (1707-3983) | 7.85 (4.96-11.65) | -2.00 (-2.06 to -1.94) | < 0.001 |
| Philippines | 9260 (6011-13649) | 33.37 (21.96-48.13) | 19974 (13065-28396) | 26.71 (17.62-37.60) | -0.71 (-0.74 to -0.69) | < 0.001 |
| Poland | 7463 (4671-10870) | 18.91 (12.14-27.43) | 8485 (5294-12438) | 12.39 (7.69-18.16) | -1.36 (-1.41 to -1.30) | < 0.001 |
| Portugal | 1043 (617-1594) | 8.82 (5.31-13.30) | 1580 (942-2463) | 5.34 (3.19-8.41) | -1.61 (-1.67 to -1.56) | < 0.001 |
| Puerto Rico | 447 (269-700) | 13.25 (8.14-20.47) | 754 (435-1153) | 9.67 (5.71-15.00) | -1.02 (-1.05 to -0.98) | < 0.001 |
| Qatar | 33 (20-50) | 22.18 (14.14-33.27) | 157 (97-240) | 15.84 (9.78-25.06) | -1.07 (-1.20 to -0.94) | < 0.001 |
| Republic of Korea | 3291 (1990-5192) | 14.59 (8.99-22.59) | 5202 (3146-8391) | 5.87 (3.52-9.54) | -2.89 (-2.95 to -2.83) | < 0.001 |
| Republic of Moldova | 1353 (857-1989) | 33.20 (20.98-48.22) | 1747 (1089-2686) | 29.76 (18.75-45.72) | -0.36 (-0.39 to -0.33) | < 0.001 |
| Romania | 4576 (2883-6708) | 19.15 (12.25-28.04) | 5338 (3382-8034) | 15.40 (9.81-22.90) | -0.7.00 (-0.79 to -0.62) | < 0.001 |
| Russian Federation | 28827 (18069-43359) | 18.04 (11.39-26.99) | 30952 (19086-45822) | 13.54 (8.48-19.92) | -0.92 (-0.98 to -0.86) | < 0.001 |
| Rwanda | 553 (358-813) | 19.05 (12.49-27.66) | 780 (492-1184) | 13.24 (8.34-19.69) | -1.16 (-1.22 to -1.09) | < 0.001 |
| Saint Kitts and Nevis | 8 (5-13) | 24.09 (15.5-35.67) | 10 (6-16) | 16.20 (9.84-25.75) | -1.27 (-1.31 to -1.23) | < 0.001 |
| Saint Lucia | 18 (11-28) | 20.88 (13.41-31.93) | 39 (24-59) | 16.95 (10.72-25.90) | -0.67 (-0.70 to -0.65) | < 0.001 |
| Saint Vincent and the Grenadines | 14 (9-21) | 19.47 (12.1-28.84) | 24 (15-36) | 18.07 (11.34-27.30) | -0.24 (-0.27 to -0.21) | < 0.001 |
| Samoa | 26 (17-40) | 33.42 (21.77-49.55) | 42 (27-63) | 32.37 (20.58-48.02) | -0.10 (-0.14 to -0.07) | < 0.001 |
| San Marino | 2 (1-3) | 6.02 (3.63-9.35) | 5 (3-9) | 5.32 (3.18-8.36) | -0.39 (-0.42 to -0.36) | < 0.001 |
| Sao Tome and Principe | 26 (17-38) | 38.10 (25.18-54.89) | 42 (27-61) | 33.48 (21.40-49.10) | -0.42 (-0.46 to -0.37) | < 0.001 |
| Saudi Arabia | 1743 (1119-2559) | 26.60 (17.01-40.13) | 4393 (2756-6683) | 21.04 (13.17-32.66) | -0.74 (-0.79 to -0.70) | < 0.001 |
| Senegal | 1207 (809-1710) | 32.67 (22.00-46.76) | 2298 (1519-3292) | 26.36 (17.59-37.74) | -0.69 (-0.73 to -0.65) | < 0.001 |
| Serbia | 1081 (668-1619) | 11.24 (7.02-16.60) | 1308 (826-1958) | 8.25 (5.20-12.49) | -0.98 (-1.02 to -0.94) | < 0.001 |
| Seychelles | 19 (12-27) | 32.23 (20.91-47.89) | 31 (20-47) | 29.33 (18.80-44.31) | -0.28 (-0.34 to -0.23) | < 0.001 |
| Sierra Leone | 824 (555-1212) | 38.08 (25.28-55.19) | 1435 (945-2114) | 34.77 (23.06-51.02) | -0.29 (-0.32 to -0.27) | < 0.001 |
| Singapore | 264 (153-435) | 13.53 (8.13-21.64) | 567 (334-973) | 6.87 (4.12-11.72) | -2.14 (-2.19 to -2.10) | < 0.001 |
| Slovakia | 828 (530-1242) | 15.26 (9.73-22.73) | 1006 (622-1534) | 11.52 (7.17-17.59) | -0.90 (-0.94 to -0.86) | < 0.001 |
| Slovenia | 263 (166-393) | 11.54 (7.28-17.28) | 378 (227-589) | 8.25 (5.04-12.69) | -1.08 (-1.13 to -1.03) | < 0.001 |
| Solomon Islands | 56 (37-79) | 41.08 (27.39-59.08) | 131 (83-192) | 38.65 (24.74-55.71) | -0.20 (-0.22 to -0.17) | < 0.001 |
| Somalia | 706 (467-1026) | 21.64 (14.18-31.27) | 1496 (942-2231) | 19.30 (12.28-28.18) | -0.36 (-0.40 to -0.32) | < 0.001 |
| South Africa | 7093 (4650-10393) | 32.02 (21.02-46.89) | 13326 (8655-19348) | 28.77 (18.69-41.87) | -0.34 (-0.39 to -0.28) | < 0.001 |
| South Sudan | 506 (333-731) | 17.97 (11.86-26.05) | 697 (440-1041) | 16.01 (10.2-23.97) | -0.38 (-0.42 to -0.33) | < 0.001 |
| Spain | 4979 (3139-7648) | 9.61 (6.07-14.74) | 7337 (4383-11404) | 5.91 (3.60-9.29) | -1.54 (-1.62 to -1.47) | < 0.001 |
| Sri Lanka | 3536 (2323-5291) | 33.97 (22.19-50.21) | 6398 (3952-9722) | 25.73 (16.14-38.93) | -0.89 (-0.93 to -0.86) | < 0.001 |
| Sudan | 2926 (1936-4197) | 29.51 (19.22-42.13) | 5103 (3295-7536) | 24.32 (15.55-35.42) | -0.62 (-0.66 to -0.58) | < 0.001 |
| Suriname | 61 (39-91) | 22.68 (14.35-34.01) | 126 (80-192) | 20.69 (13.22-31.16) | -0.30 (-0.34 to -0.27) | < 0.001 |
| Sweden | 993 (613-1518) | 6.21 (3.83-9.52) | 1270 (760-2027) | 4.74 (2.87-7.52) | -0.86 (-0.89 to -0.83) | < 0.001 |
| Switzerland | 929 (586-1472) | 8.61 (5.33-13.63) | 1471 (887-2345) | 6.75 (4.12-10.59) | -0.75 (-0.79 to -0.71) | < 0.001 |
| Syrian Arab Republic | 1770 (1152-2645) | 28.65 (18.79-42.50) | 2496 (1577-3751) | 20.51 (13.27-30.67) | -1.07 (-1.16 to -0.98) | < 0.001 |
| Taiwan (Province of China) | 2787 (1774-4177) | 21.39 (13.54-32.09) | 6217 (3840-9403) | 14.29 (8.93-21.70) | -1.29 (-1.42 to -1.17) | < 0.001 |
| Tajikistan | 1187 (792-1691) | 41.37 (27.98-59.09) | 2415 (1575-3453) | 40.65 (26.55-58.44) | -0.06 (-0.10 to -0.02) | 0.002 |
| Thailand | 12072 (7805-17674) | 38.24 (24.78-56.48) | 37253 (23542-55485) | 34.72 (21.89-51.58) | -0.31 (-0.35 to -0.26) | < 0.001 |
| Timor-Leste | 91 (59-133) | 34.25 (22.29-48.95) | 226 (144-334) | 28.98 (18.74-43.22) | -0.53 (-0.57 to -0.49) | < 0.001 |
| Togo | 558 (360-815) | 38.47 (24.89-55.81) | 1611 (1051-2283) | 38.21 (24.74-54.50) | -0.01 (-0.04 to 0.01) | 0.262 |
| Tokelau | 0 (0-1) | 36.26 (23.04-53.04) | 0 (0-1) | 32.78 (20.67-46.78) | -0.32 (-0.34 to -0.31) | < 0.001 |
| Tonga | 18 (11-26) | 34.00 (21.8-49.44) | 26 (17-39) | 33.41 (21.66-49.87) | -0.06 (-0.09 to -0.03) | < 0.001 |
| Trinidad and Tobago | 154 (96-233) | 19.05 (11.83-28.73) | 284 (179-449) | 15.75 (9.86-24.73) | -0.62 (-0.65 to -0.59) | < 0.001 |
| Tunisia | 890 (595-1270) | 17.97 (12.11-25.36) | 1901 (1237-2761) | 15.24 (9.87-22.18) | -0.52 (-0.55 to -0.48) | < 0.001 |
| Turkey | 8599 (5708-12565) | 23.27 (15.37-33.52) | 13910 (9039-20337) | 15.59 (10.15-22.92) | -1.26 (-1.34 to -1.17) | < 0.001 |
| Turkmenistan | 925 (612-1338) | 44.91 (29.77-65.39) | 1580 (1021-2294) | 38.46 (24.75-55.73) | -0.50 (-0.56 to -0.45) | < 0.001 |
| Tuvalu | 2 (2-4) | 40.85 (26.35-59.42) | 3 (2-5) | 36.74 (23.03-55.38) | -0.34 (-0.36 to -0.32) | < 0.001 |
| Uganda | 1518 (1000-2169) | 21.99 (14.53-31.11) | 2911 (1899-4333) | 17.84 (11.53-26.28) | -0.67 (-0.70 to -0.64) | < 0.001 |
| Ukraine | 11032 (6985-16351) | 17.33 (11.16-25.66) | 12458 (7814-18667) | 16.68 (10.45-24.99) | -0.12 (-0.16 to -0.07) | < 0.001 |
| United Arab Emirates | 204 (122-307) | 27.81 (16.97-42.68) | 1114 (677-1787) | 19.45 (12.07-30.03) | -1.14 (-1.17 to -1.11) | < 0.001 |
| United Kingdom | 8408 (5207-12581) | 9.41 (5.86-14.38) | 9957 (5868-15531) | 6.92 (4.11-10.85) | -0.99 (-1.01 to -0.96) | < 0.001 |
| United Republic of Tanzania | 2488 (1655-3540) | 19.22 (12.69-27.77) | 4508 (2865-6653) | 15.13 (9.83-22.21) | -0.79 (-0.85 to -0.74) | < 0.001 |
| United States of America | 33706 (21399-49574) | 10.23 (6.50-15.12) | 70394 (44252-107910) | 11.75 (7.37-17.92) | 0.45 (0.39 to 0.52) | < 0.001 |
| United States Virgin Islands | 11 (7-17) | 14.16 (8.43-21.84) | 20 (12-31) | 12.33 (7.58-19.46) | -0.43 (-0.48 to -0.39) | < 0.001 |
| Uruguay | 364 (224-555) | 9.81 (6.08-15.08) | 484 (297-734) | 7.50 (4.60-11.49) | -0.88 (-0.95 to -0.81) | < 0.001 |
| Uzbekistan | 8062 (5295-11853) | 62.49 (40.87-92.31) | 15245 (9673-23180) | 54.75 (34.88-82.32) | -0.44 (-0.48 to -0.40) | < 0.001 |
| Vanuatu | 22 (14-32) | 36.76 (23.88-53.14) | 65 (41-96) | 40.04 (25.48-56.83) | 0.28 (0.24 to 0.31) | < 0.001 |
| Venezuela (Bolivarian Republic of) | 1692 (1062-2559) | 17.71 (11.26-26.76) | 4424 (2861-6671) | 15.64 (10.10-23.58) | -0.38 (-0.45 to -0.32) | < 0.001 |
| Viet Nam | 8374 (5336-12523) | 21.37 (13.69-31.65) | 12762 (8139-19083) | 14.63 (9.38-21.84) | -1.19 (-1.28 to -1.11) | < 0.001 |
| Yemen | 1506 (1030-2129) | 29.08 (19.66-41.35) | 4099 (2857-5856) | 26.61 (18.03-38.09) | -0.28 (-0.31 to -0.25) | < 0.001 |
| Zambia | 769 (521-1106) | 22.76 (15.28-32.93) | 1782 (1157-2668) | 21.30 (13.90-31.26) | -0.20 (-0.24 to -0.17) | < 0.001 |
| Zimbabwe | 1630 (1054-2372) | 37.16 (24.37-54.66) | 2677 (1752-3849) | 37.58 (25.31-53.97) | 0.07 (-0.02 to 0.15) | 0.133 |

Abbreviations: YLDs, years lived with disability.
